# Modifications of NU-9, a potent protein aggregation inhibitor. Properties and activity in a cellular model of amyotrophic lateral sclerosis

**DOI:** 10.1016/j.bioorg.2025.109190

**Published:** 2025-11-02

**Authors:** Mohamed F. Elmansy, Pedro Soares, Jose Ricardo D. dos Remedios, Raghad Nowar, Susan G. Fox, Anan Yu, William L. Klein, Richard I. Morimoto, Richard B. Silverman

**Affiliations:** a Department of Chemistry, Chemistry of Life Processes Institute, and Center for Developmental Therapeutics, Northwestern University, Evanston, IL 60208, USA; b Organometallic and Organometalloid Chemistry Department, National Research Centre, 12622 Cairo, Egypt; c Department of Molecular Biosciences, Northwestern University, Evanston, IL 60208, USA; d Department of Neurobiology, Northwestern University, Evanston, IL 60208, USA; e Daniel F. and Ada L. Rice Institute for Biomedical Research, Northwestern University, Evanston, IL 60208, USA; f Department of Neurology, Northwestern University, Chicago, IL 60611, USA; g Department of Pharmacology, Feinberg School of Medicine, Northwestern University, Chicago, IL 60611, USA

**Keywords:** Cyclohexane-1,3-diones, Small-molecules, Amyotrophic lateral sclerosis, SOD-1, Protein aggregation

## Abstract

Amyotrophic lateral sclerosis (ALS) is a fast-progressing disease characterized by the loss of voluntary movements and death due to respiratory failure. The presence of protein aggregates is a major hallmark of the disease. Hence, targeting the pathological protein aggregation may provide more efficient therapeutics for ALS.

Recently, we reported a cyclohexane-1,3-dione (**NU-9**) with *in vitro* anti-aggregation capacity and promising *in vivo* efficacy in ALS animal models, which validated our approach toward the development of novel and potentially more effective ALS therapeutics. Herein, we report the design and synthesis of a new series of small-molecule derivatives of **NU-9** and the evaluation of their *in vitro* anti-aggregation activity in a PC12 cellular model containing an SOD1^G85R^ familial ALS mutation. The most promising compound (**20**) presented an *in vitro* anti-aggregation potency comparable to that of **NU-9**. Moreover, the better *in vitro* BBB permeability, microsomal stability, and toxicity profile of **20** also suggests a potentially higher efficacy *in vivo*.

## 1. Introduction

Amyotrophic lateral sclerosis (ALS) is a fast progressing disease characterized by the progressive degeneration of upper and lower motor neurons, leading to an increasing loss of voluntary movements and ultimately death due to respiratory failure in 2–5 years after diagnosis [1–3]. It has an incidence of 0.6–3.8 people per 100,000 and a prevalence of 4.1–8.4 people per 100,000 in Europe and the United States [4]. About 90 % of ALS cases are sporadic and 10 % familial with the onset of the disease generally observed in people between 40 and 60 years of age; patients with familial ALS generally experience an earlier onset of the disease [5,6].

There is currently no cure for the disease, and the approved therapies are only capable of providing minimal symptomatic relief to the patients [7]. The only FDA-approved drugs for ALS are the repurposed drugs riluzole (glutamate antagonist) and edaravone (radical oxygen scavenger), and an antisense oligonucleotide for SOD1 familial ALS, tofersen, that have only a minor effect in delaying the progression of the symptoms in a subset of patients [8,9].

In ALS, ageing and the presence of genetic mutations are two prominent factors that compromise the efficiency of the proteostasis network and lead to protein aggregation [10–13]. Furthermore, proteins associated with ALS pathology, such as SOD1 and TDP-43, exhibit prion-like properties and can propagate protein misfolding and aggregation, leading to continuous pathology progression in motor neurons and other neuronal cells [14–19]. When compared with other cells in the CNS, motor neurons are particularly vulnerable to proteotoxic stress due to proteostasis imbalance and stress induced by protein aggregates accumulated through ageing [20–22]. Consequently, the discovery of therapeutic approaches capable of modulating the biological mechanisms contributing to pathological protein aggregation may lead to novel and potentially more efficient therapeutics for ALS [23].

**Fig. 1.**
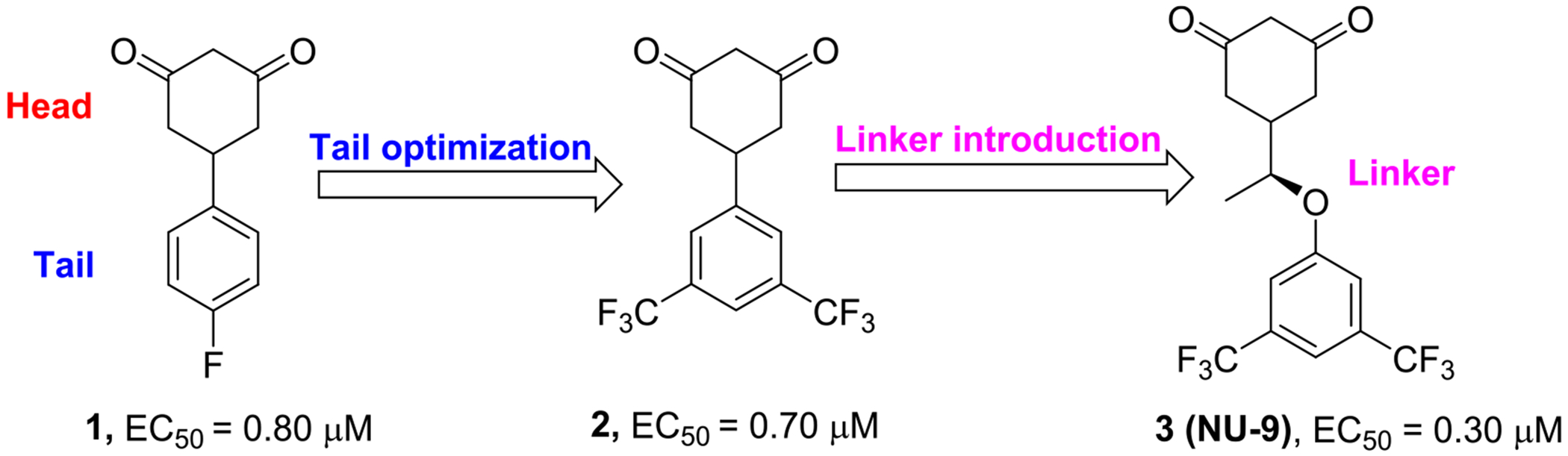
Evolution of CHD series and the linker introduction.

The pathological role of the abnormal formation of protein aggregates on ALS pathology motivated the Silverman group and collaborators to conduct an initial high-throughput phenotypic screen (HTPS) aiming to discover small-molecule hits with anti-aggregation capacity in cells containing SOD1 mutations associated with ALS [24]. The initial HTPS led to the discovery of cyclohexane-1,3-dione^2^(CHD) **1** (Fig. 1) as one of the promising hits, which was considered for further optimization [24]. The initial structure-activity relationship studies around the structure of CHD hit **1** led to the observation that meta-substituents on the tail fragment were crucial for enhancing the pharmacodynamic properties of the compounds, and 3,5-bis(trifluoromethyl)cyclohexane-1,3-dione **(2)** was identified but was inactive in cortical neuron protection assays due to lack of penetration into neurons [25]. To overcome this problem, a linker was introduced between the head and tail units, leading to the discovery of **NU-9**, a compound with enhanced anti-aggregation activity *in vitro* and promising *in vivo* activity in ALS animal models [26]. Furthermore, **NU-9** was also capable of decreasing upper motor neuron degeneration and improving the health of mitochondria, endothelium reticulum (ER), and overall cytoskeletal integrity in the neurons of the ALS animal models [27].

These promising results of **NU-9** encouraged us to systematically explore modifications on the linker and head parts of **NU-9**. The 3,5-bis (trifluoromethyl)phenyl- was previously identified as the most potent tail moiety of our compounds and was retained. We explored the optimization of the linker and the head of **NU-9**. In this paper, we present the synthesis and *in vitro* SAR studies of the newly synthesized **NU-9** analogues against mutant superoxide dismutase 1 (SOD1) aggregation and the *in vitro* pharmacological studies that led us to identify **20,** a compound with promising anti-aggregation activity, BBB permeability, and a good pharmacological profile, as a candidate for further ALS therapeutic validation.

## 2. Results and discussion

### 2.1. Chemistry

**Fig. 2.**
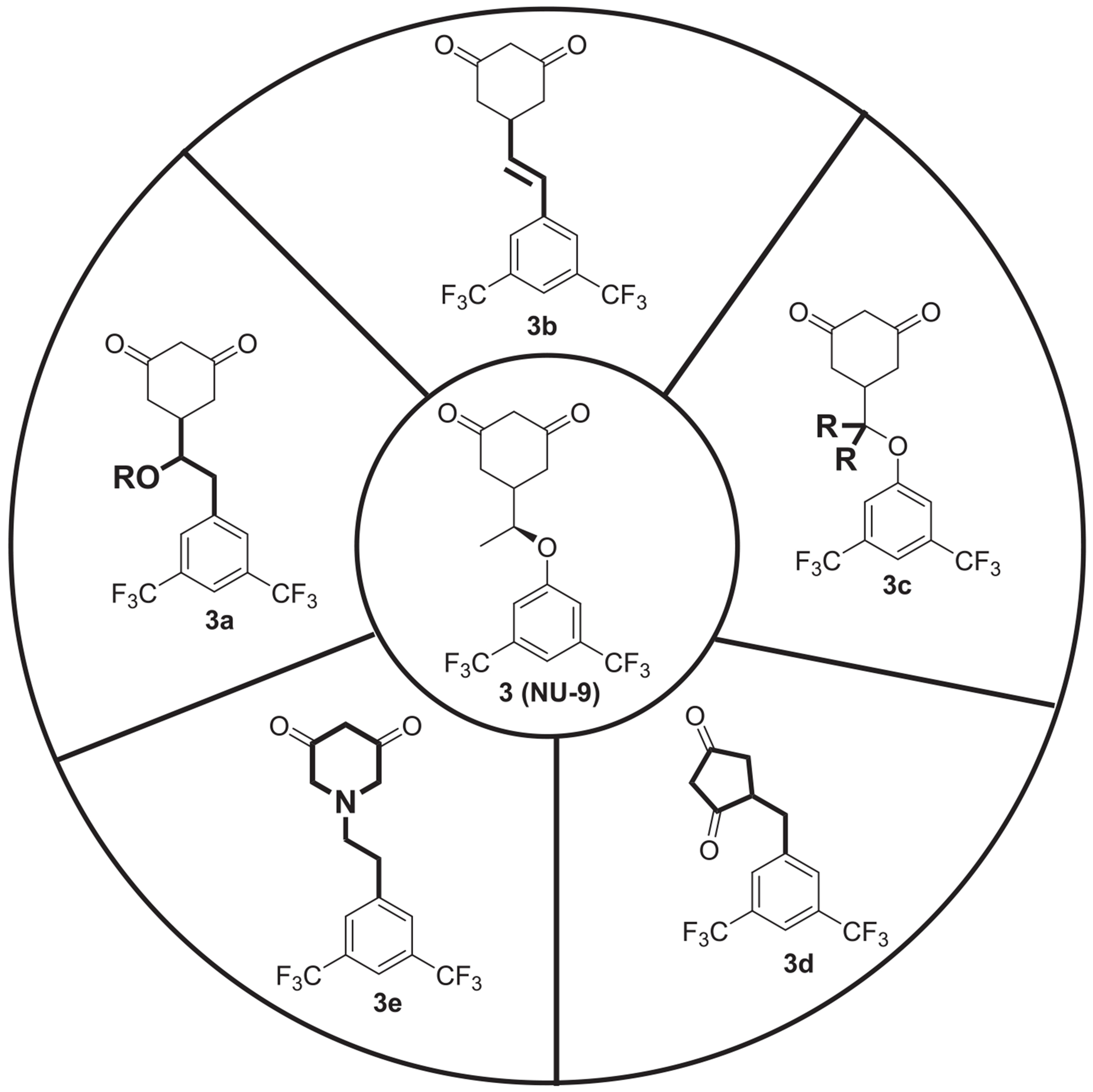
Designed **NU-9** analogues to explore head and linker optimization.

Compound **NU-9** was synthesized following a previously described protocol [25,26]. We then expanded our SAR, obtaining a library of compounds based on the chemical structure of **NU-9**, retaining the 3,5-bis(trifluoromethyl)benzene, previously identified as the most potent tail moiety. In our work, we designed new **NU-9** analogues with chemical modifications that, as exemplified in Fig. 2, target the linker (**3a-3c**) and head (**3d-3e**) of the compounds. Modification of the linker backbone from ether to carbon and the introduction of a hydroxyl group to the linker allowed us to study the impact of adding a hydrogen bond on the compounds potency through **NU-9** structural isomer **3a**. Lowering the number of rotatable bonds and limiting the flexibility of small molecules is reported to impact the pharmacokinetic and pharmacodynamic profile of drugs and drug candidates [28–30]. Hence, we aimed to introduce an alkene linker in the compounds (**3b**) to restrict linker flexibility. Additionally, with the structural modifications represented on **3c**, we aimed to study the linker steric hindrance by introducing different substituents to induce conformational constraints on the compounds. We also added modifications to the head of the compounds, with the modification highlighted in **3d** we aimed to study the effect of decreasing the ring size, while with derivatives represented on **3e**, through the addition of an *N* to the cyclohexane-1,3-dione ring we aimed to evaluate the change of the electronics on the activity of the compound and improve the pharmacokinetic properties (Fig. 2).

**Scheme 1.**
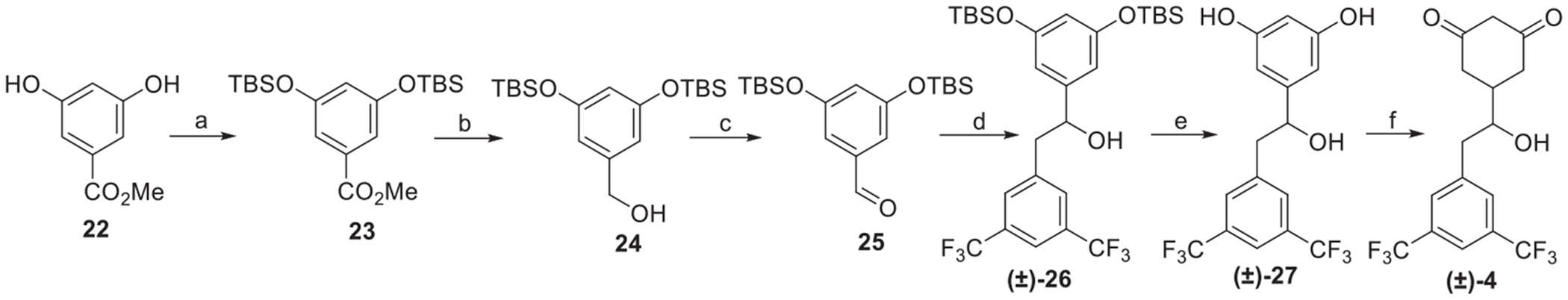
Synthesis of hydroxy-substituted CHD linker **(±)-4**^a^ ^a^ Reagents and conditions: (a) TBSCl, DMAP, imidazole, DCM, rt.; (b) DIBAl-H, DCM, 0 °C; c) PCC, NaOAc, DCM; (d) 3,5-bis(trifluoromethyl)benzyl magnesium bromide, Et_2_O, rt.; (e) HCl (conc.), MeOH, rt.; (f) NaOH, H_2_ (balloon), Rh/Al_2_O_3_ (10 %), H_2_O, 90 °C.

The hydroxy-substituted CHD linker compound **(±)-4** was synthesized starting with the di-TBS protection of 3,5-dihdroxymethylbenzoate **(22)** to obtain compound **23** followed by DIBAL-H reduction to give alcohol **24**, which was then oxidized to the corresponding aldehyde **25**. The Grignard reaction between the aldehyde and 3,5-bis(trifluoromethyl)benzyl magnesium bromide proceeded smoothly providing the secondary alcohol **(±)-26**, which was cleanly deprotected to the corresponding diphenol **(±)-27**. The alkaline Rh/Al_2_O_3_ catalyzed hydrogenation provided the desired final diketone **(±)-4** (Scheme 1).

**Fig. 3.**
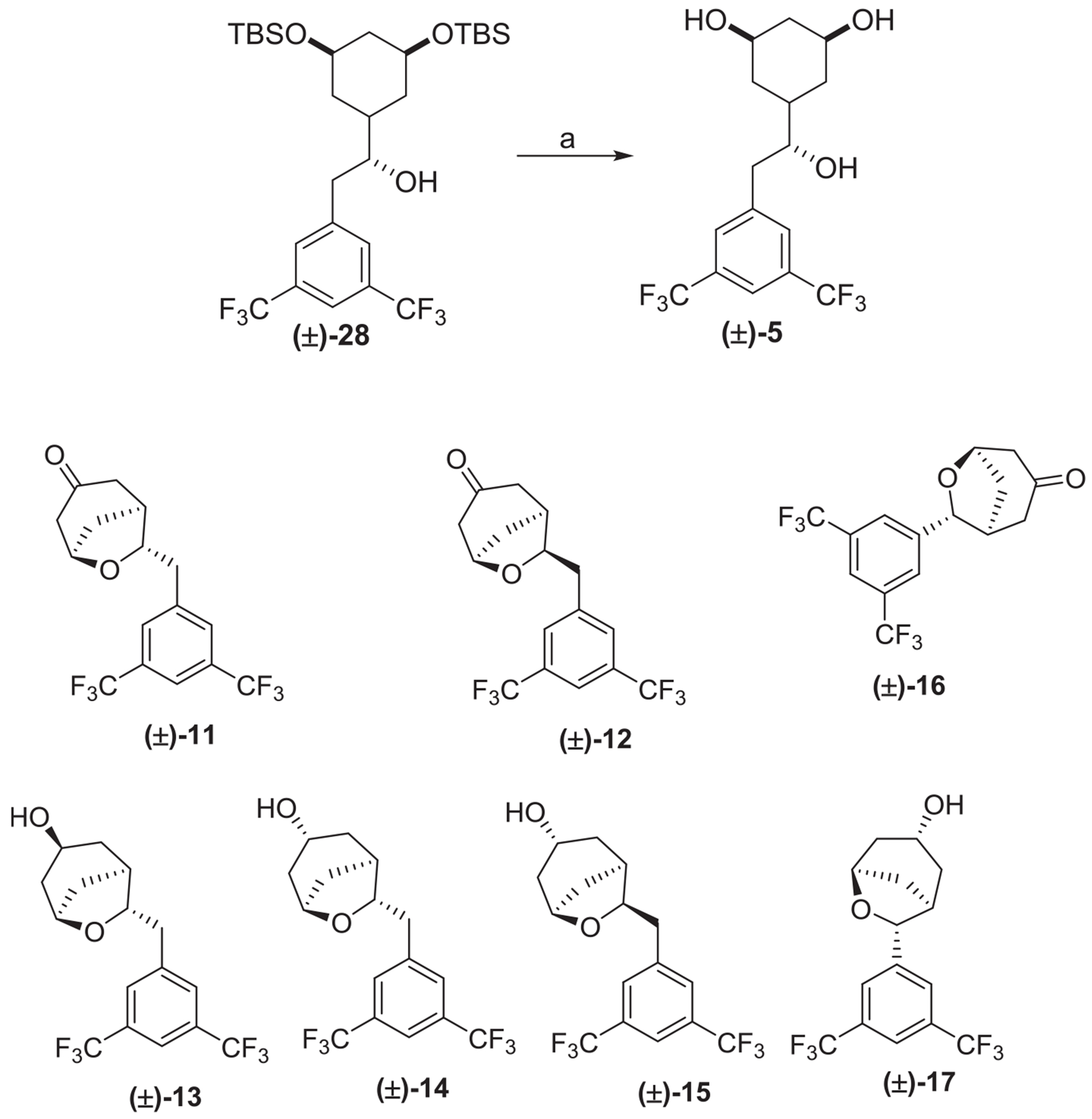
Bicyclic analogues of **NU-9.**

**Scheme 2.**
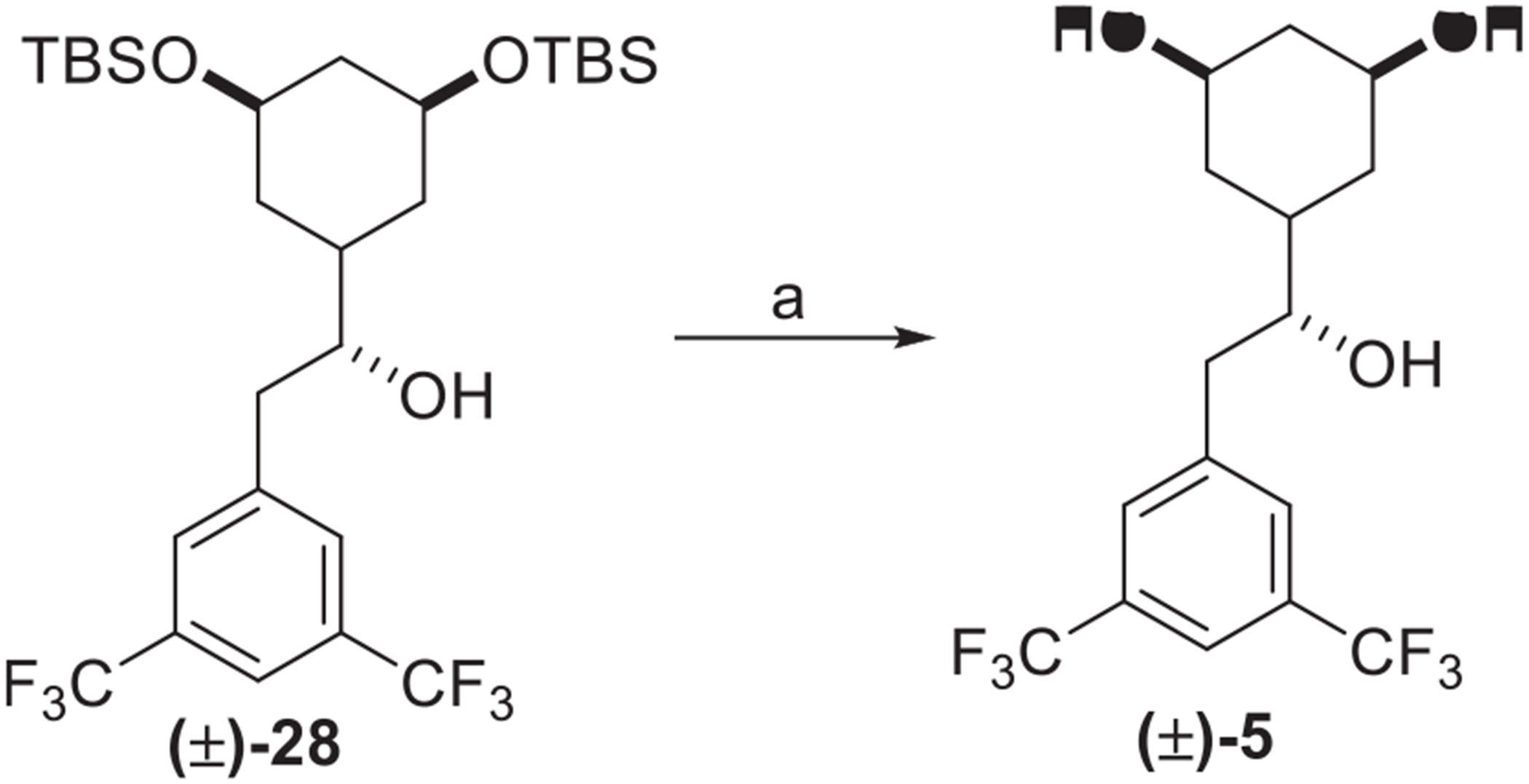
Synthesis compound **(±)-5**^a^ ^a^ Reagents and conditions: (a) HCl, MeOH, rt.

Starting from the recently reported compound **(±)-28,** the cleavage of the TBS-protecting group under acidic conditions provided the triol **(±)-5** (Scheme 2). An initial attempt was made to access the alkene-based linker on our compounds by Burgess reagent-mediated elimination of secondary alcohol on **(±)-28** followed by silyl deprotection. However, our approach led to the synthesis of bicyclic alcohols **(±)-13** to **(±)-15** and **(±)-17** *via* unprecedented Burgess reagent-mediated cyclodehydration of δ-diols under acidic conditions [31]. These bicyclic alcohols were oxidized to provide the corresponding ketones **(±)-11**, **(±)-12** and **(±)-16** (Fig. 3) [31].

**Scheme 3.**
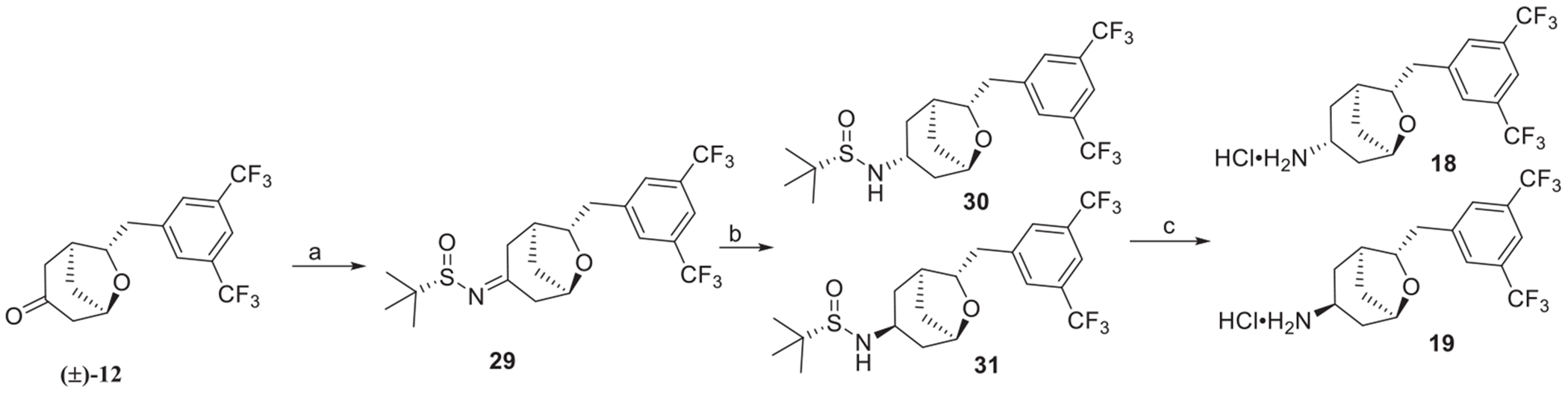
Synthesis of chiral bicyclic amines **18** and **19** ^a^ Reagents and conditions: (a) (*S*)-tert-butanesulfinamide, Ti(O-iPr)_4_, THF, reflux; (b) NaBH_4_, THF, rt.; (c) HCl, MeOH, rt.

Derivatization of ketone **(±)-12** using Ellman’s^1^ chiral *(S)*-tertbutanesulfinamide provided an inseparable mixture of imines **29** that, upon reduction, gave two separable sulfonamides **30** and **31** respectively. Sulfinamide deprotection using methanolic hydrochloric acid provided chiral amines **18** and **19** (Scheme 3).

**Scheme 4.**
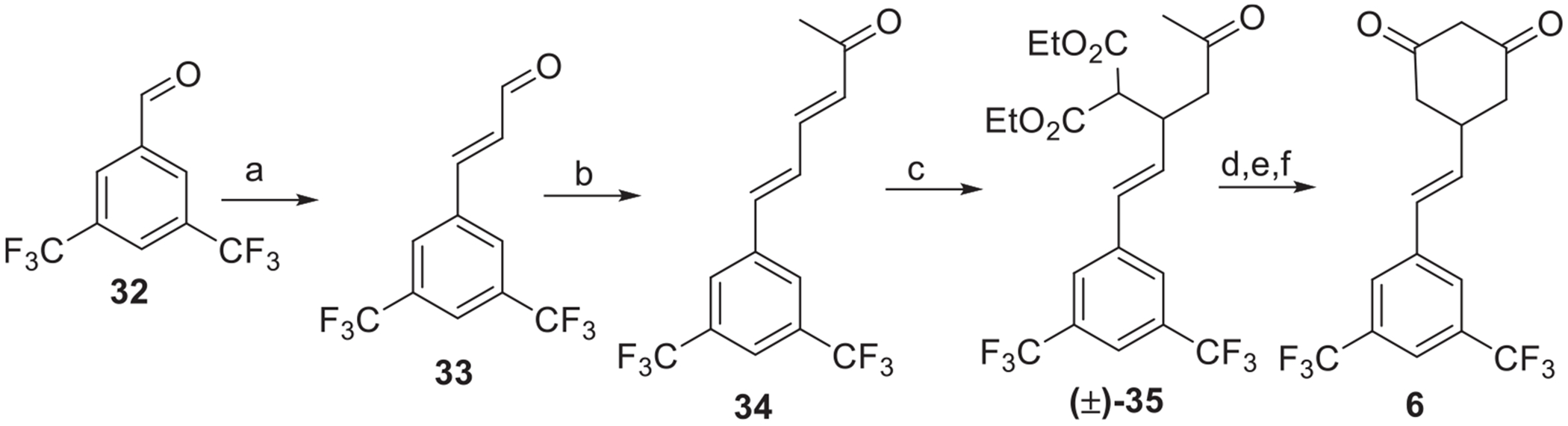
Synthesis of trans alkene linker-based cyclohexane-1,3-dione **6**^a^ ^a^Reagents and conditions: (a) (triphenylphosphoranylidene)acetaldehyde, THF, rt.; (b) 1-(triphenylphosphoranylidene)-2-propanone, THF, rt.; (c) diethylmalonate, Et_3_N, LiI, toluene, rt.; (d) NaOEt, EtOH, rt. (e) 2 N NaOH, rt. (aq); (f) 2 N HCl, 100 °C.

We envisioned that the synthesis of the alkene-based linker CHD **6** could be achieved by revisiting the synthetic route developed by our group [25] with some modifications. Successive Wittig reactions [32] of 3,5-bis(trifluoromethyl)benzaldehyde **(32)** led to the corresponding dienone (**34**). Selective 1,4-addition of diethylmalonate, in the presence of a catalytic amount of lithium iodide, yielded **(±)-34**. The malonate derivative **(±)-35**, was cyclized upon treatment with sodium ethoxide solution, followed by ester hydrolysis and then decarboxylation to provide the target alkene linker CHD **6** (Scheme 4).

**Scheme 5.**
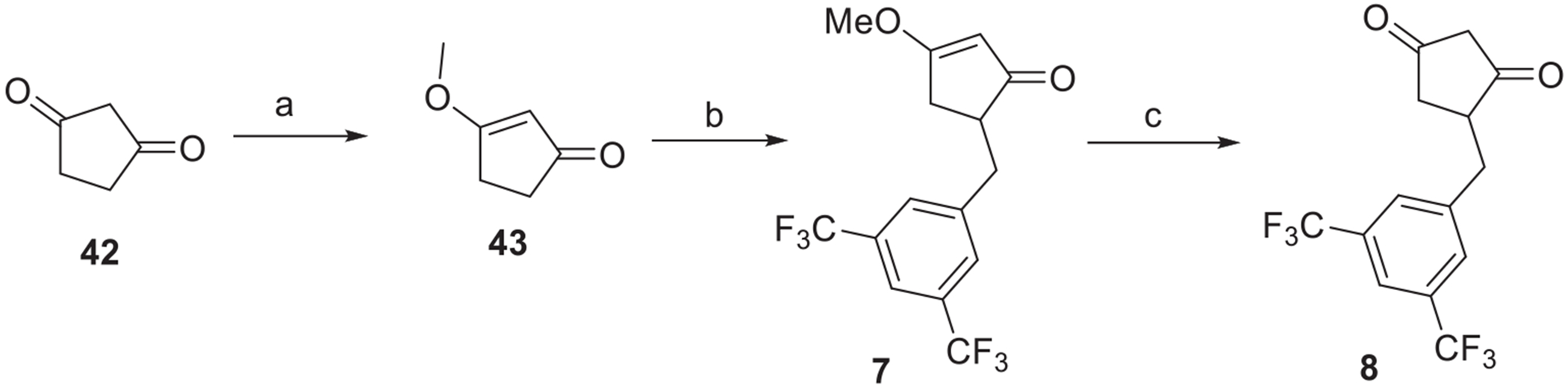
Synthesis of 3-methoxycyclopent-3-en-1-one **7** and 1,3-cyclopentanedione **8** ^a^ ^a^ Reagents and conditions: (a) I_2_ (6 mol%), MeOH, rt.; (b) 3,5-bis(trifluoromethyl)benzyl bromide, LDA, THF, −78 °C; c) HCl, THF, 70 °C.

To explore the head moiety, we aimed to synthesize the corresponding 1,3-cyclopentanedione analogue by protection of commercially available 1,3-cyclopentanedione (**42**) according to the procedure reported by Kagan [33]. Treatment of methyl enol ether **43** with 3,5-bis(trifluoromethyl)benzyl bromide in the presence of lithium diisopropylamide gave compound **7**. Deprotection of **7** was achieved using HCl in THF to provide the corresponding 1,3-cyclopentanedione **8** (Scheme 5).

**Scheme 6.**
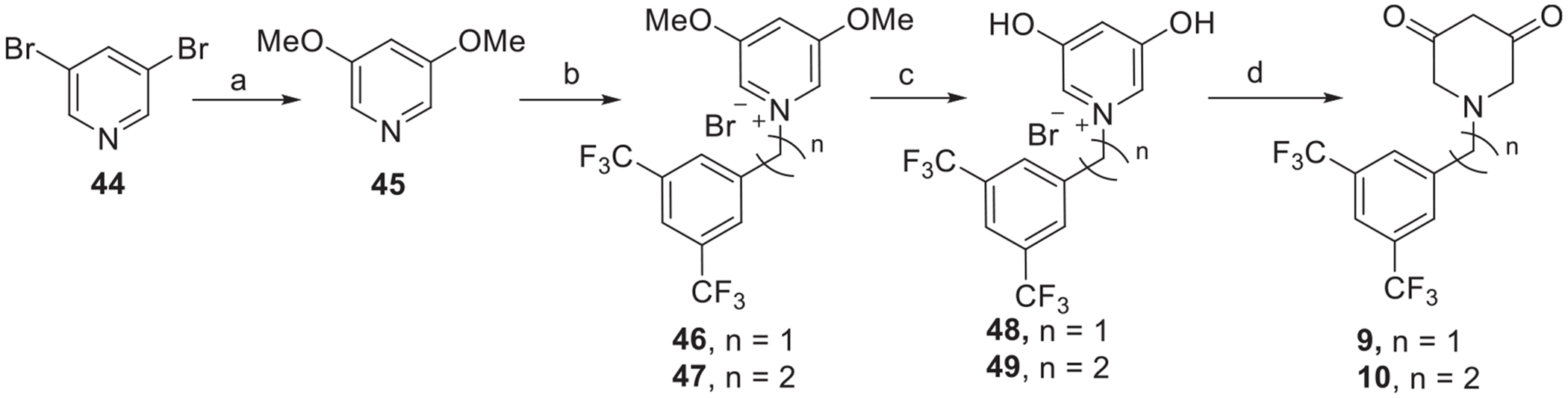
Synthesis of 3,5-piperidinedione derivatives **9** and **10**^a^ ^a^ Reagents and conditions: (a) MeONa, CuBr, DMF, 90 °C; (b) 3,5-bis(trifluoromethyl)benzyl bromide or 1-(2-bromoethyl)-3,5-bis(trifluoromethyl)benzene, DCM, 70 °C; c) HBr, 120 °C; (d) H_2_ (balloon), NaOH, Rh/Al_2_O_3_, H_2_O, 75 °C.

To obtain 3,5-piperidinedione derivatives **9** and **10** we started with 3,5-dibromopyridine (**44**), which resisted alkylation. Switching the bromides to the corresponding dimethoxy groups [34] (**45**) allowed the alkylation to occur and to give the quaternary ammonium salts (**46** and **47**). Methoxy deprotection was carried out using 48 % HBr solution to afford the diphenols (**48** and **49**), which were converted to the corresponding final 3,5-piperidinedione derivatives **9** and **10** by hydrogenation (Scheme 6).

**Scheme 7.**
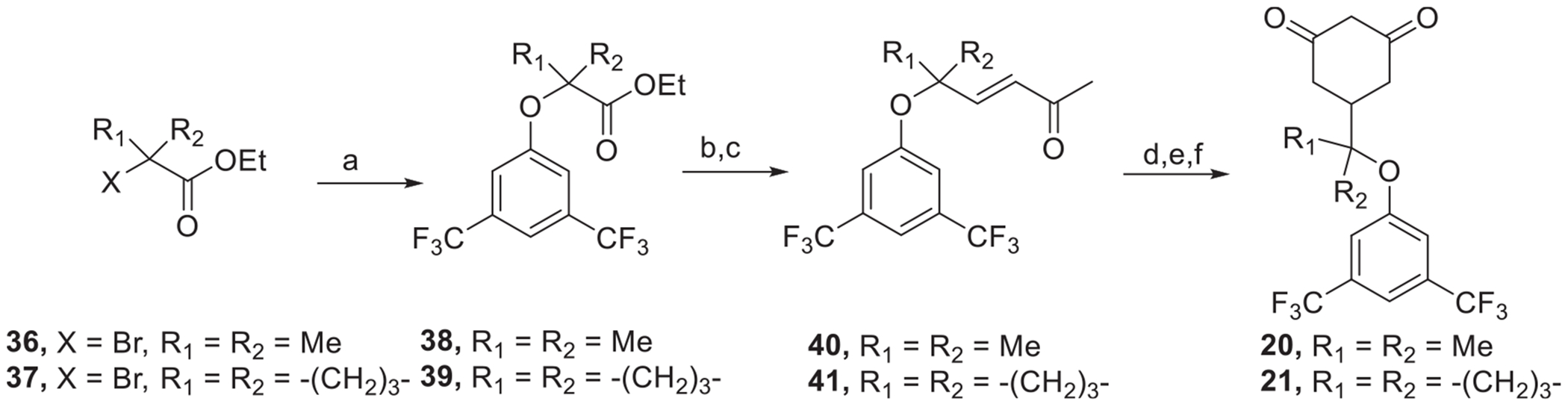
Synthesis of different sterically hindered cyclohexane-1,3-diones **20-21**^a^ ^a^ Reagents and conditions: (a) 3,5-bis(trifluoromethyl)phenol, (CH_3_)_3_COK, KI, DMF, 85 °C; (b) DIBAL-H, DCM, −78 °C; c) 1-(triphenylphosphoranylidene)-2-propanone, THF, rt.; (d) diethylmalonate, EtONa, EtOH, rt.; (e) 2 N NaOH (aq), rt.; (f) 2 N HCl, 100 °C.

To access the sterically hindered cyclohexane-1,3-dione derivatives **20** and **21**, we started from the nucleophilic substitution of α-bromoesters (**36** and **37**) with 3,5-bis(trifluoromethyl)phenolate, giving the corresponding α-oxy esters (**38** and **39**). Controlled reduction of the ester to an aldehyde with DIBAL-H at −78 °C directly followed by a Wittig olefination furnished the corresponding enones (**40** and **41**). The final cyclohexane-1,3-diones **20** and **21** were obtained in a one-pot, four-step sequence starting with Michael addition of diethylmalonate, followed by intramolecular cyclization, hydrolysis, and decarboxylation upon heating (Scheme 7).

**Fig. 4.**
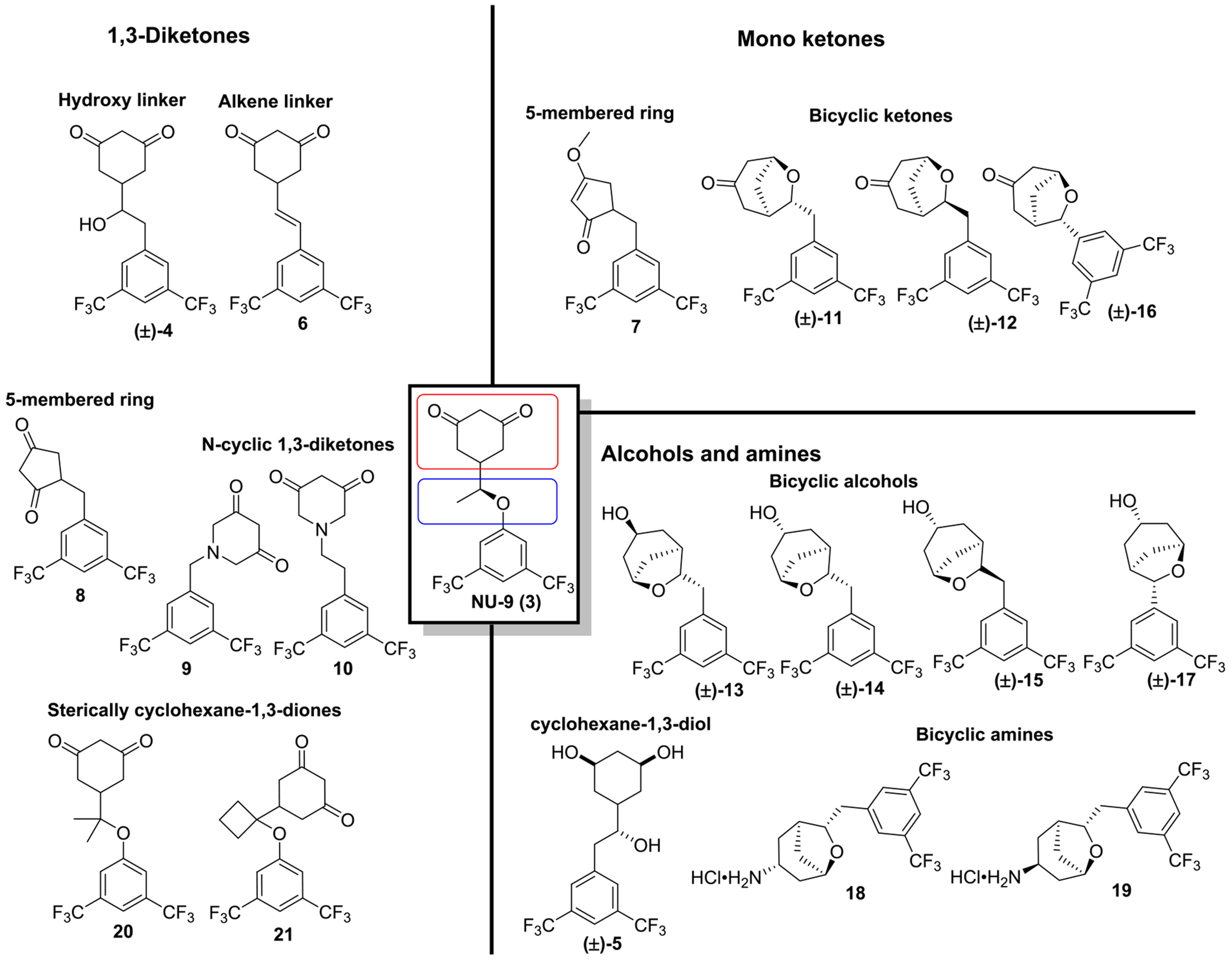
Chemical structures of NU-9 and its derivatives evaluated for their anti-aggregation capacity in the ALS cellular model.

In summary, through our synthetic efforts, a series of new 1,3-diketones, cyclohexane-1,3-diol and mono-ketone derivatives were obtained. Additionally, novel oxabicyclo[3.2.1]octan-3-one scaffolds were also accessed *via* unprecedented Burgess reagent-mediated cyclodehydration of δ-diols under acidic conditions. Hence, our efforts allowed us to assemble a library with carefully designed derivatives which were evaluated for their capacity to decrease mut-SOD1-induced aggregation in an ALS *in vitro* cellular model (Fig. 4).

### 2.2. In vitro compound evaluation of their protective capacity against Mut-SOD1-induced aggregation

**Fig. 5.**
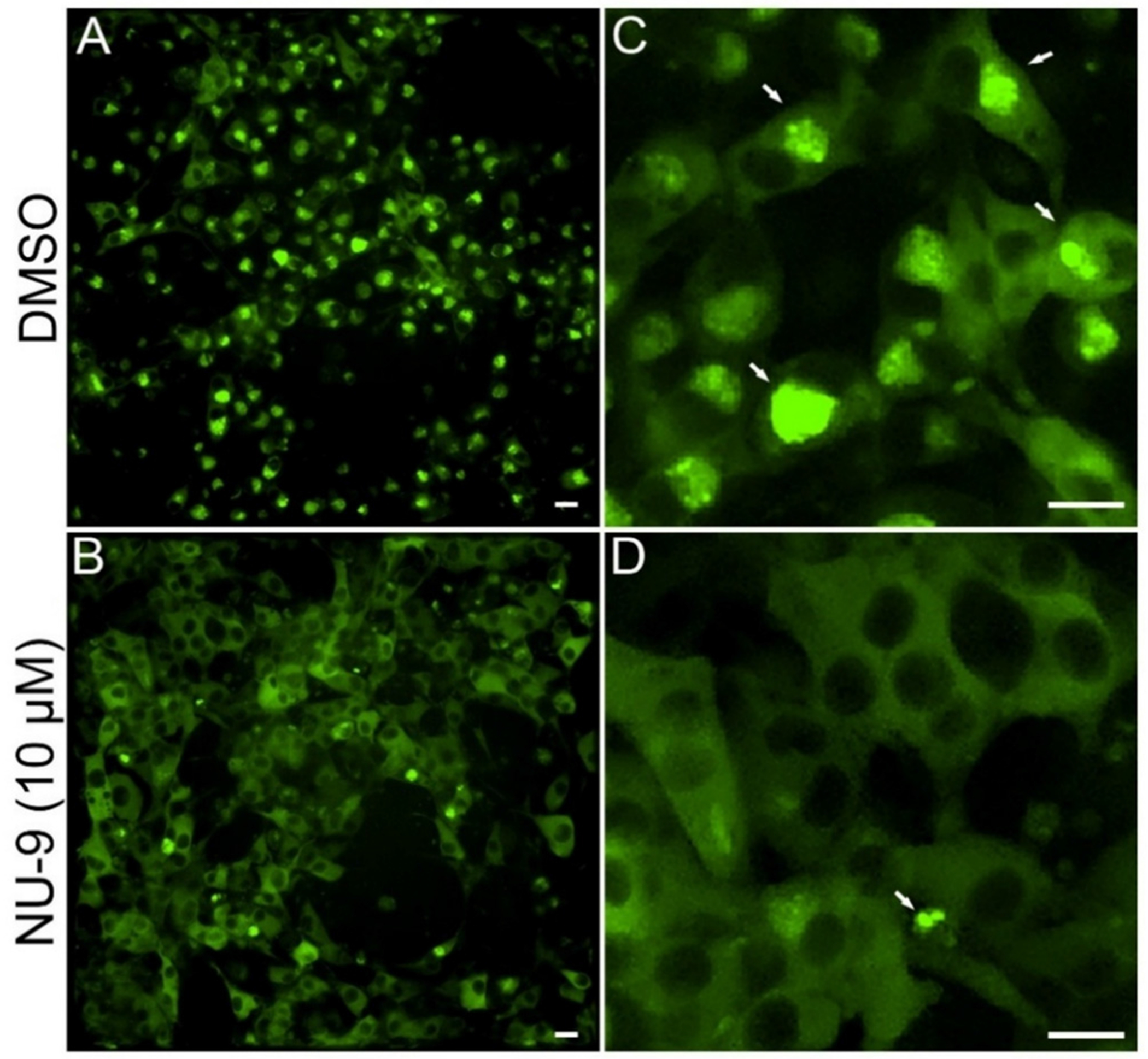
Fluorescence microscopy images of PC12-Tet-Off SOD1^G85R^ YFP cells 72 h after 0.7 μM MG132 and co-treatment with (A) DMSO or (B) NU-9 (10 μM). Arrows in enlarged panels (C) and (D) point to aggregates. The measurement bars on the right lower corner of the figures have a scale of 100 μm.

Ubiquitination is a crucial step to mark misfolded proteins for proteolytic degradation through the UPS, which is the primary system involved in this process [35,36]. Ubiquitinated protein aggregates have been observed in both ALS patients and models of the disease, suggesting defective UPS activity [35,37]. Additionally, proteasome inhibition by MG132 enhances intracellular aggregation of TDP-43 and SOD1 in ALS cellular models [24,38,39]. Based on these observations, we screened the *in vitro* anti-aggregation capacity of a library of compounds derived from **NU-9** using a PC12 pheochromocytoma Tet-Off cell line expressing a SOD1-YFP protein containing a G85R mutation that is associated with familial ALS pathology (PC12-SOD1^G85R^ YFP) by fluorescence microscopy (Table 1 and SI Figs. 1 and 2) [40]. Treatment of this cell line with MG132 led to a notable increase in mutant SOD1 intracellular aggregates after 72 h of treatment. However, co-treatment of the cellular model with **NU-9** led to a marked decrease in the observed intracellular aggregates (Fig. 5).

Our initial compound, **NU-9**, yielded an EC_50_ of 2.52 μM. Conversion of the ether linker between the cyclohexane-1,3-dione and the phenyl ring of **NU-9** into a hydroxyethylene linker (**4**) resulted in a 3-fold loss of potency. Additional reduction of the cyclohexane-1,3-dione to a 1,3-cyclohexanediol resulted in an inactive compound **5**. However, conversion of the ether linker into a more conformationally restricted ethylene linker (**6**) increased the anti-aggregation capacity of the compounds.

Contraction of the cyclohexane-1,3-dione into a 1,3-cyclopentadiene (**8**) promoted a significant loss in activity, and mono-methylation of this moiety into a 3-methoxycyclopent-3-en-1-one (**7**) depleted the activity of the compound. The conversion of cyclohexane-1,3-dione into a piperidine-3,5-dione (**9** and **10**) also induced a considerable loss of anti-aggregation activity. The less conservative conversion of the cyclohexane-1,3-dione into an expanded oxabicyclo[3.2.1]octan-3-one moiety and its derivatives (**11–19**) mostly provided inactive derivatives, with only compound **16** presenting mild anti-aggregation activity at 10 μM (SI Fig. 1). These results indicated that the cyclohexane-1,3-dione moiety was a crucial pharmacophore of our molecules, and any modifications to this moiety led to a great loss of potency. Following this rationale, and the observation that restricting the rotation of the linker between the cyclohexane-1,3-dione and the phenyl ring (**6**) improved the potency, we evaluated **NU-9** spiro derivatives **20** and **21**. The functionalization of the α-carbon to the ether group with two methyl groups (**20**) provided a compound with a potency comparable to **NU-9**; however, expansion of the substitution to a cyclobutyl moiety (**21**) led to more than a 2-fold loss in potency.

Compounds **6** and **20** were the most promising compounds in our screening, having, respectively, slightly improved or comparable anti-aggregation activity relative to **NU-9**. Furthermore, the cyclohexane-1,3-dione head group was essential to retain activity, and changes to this moiety resulted in a considerable loss of anti-aggregation activity. We also observed that hydrophobicity, a parameter known to impact small-molecule intracellular permeability, was not directly correlated with an increase in compound potency: **21** presented more than a 2-fold loss in potency compared with **NU-9** despite having greater lipophilicity. Furthermore, restriction of the linker rotation appears to contribute to an increase in potency.

### 2.3. In vitro evaluation of blood-brain barrier (BBB) permeability and pharmacokinetic and cellular cytotoxicity profile of the most promising cyclohexane-1,3-dione derivatives

The efficacy of the drugs developed for neurodegenerative diseases is critically dependent on their ability to permeate the BBB and accumulate in the brain at concentrations that allow them to engage their targets and promote a therapeutic effect in the central nervous system (CNS) [41]. The BBB is predominantly formed by endothelial cells with tight junctions between them acting as a gatekeeper, restricting the access of toxic and therapeutic agents into the brain [41,42]. Passive diffusion of drugs through biological membranes, such as the BBB, is the most common and energetically efficient process of permeation [41]. One of the most common methods to evaluate small-molecule BBB permeability is the parallel artificial membrane permeability assay (PAMPA-BBB), in which the passive diffusion of small molecules through a membrane that mimics the physicochemical environment of the BBB is evaluated [43]. Consequently, the PAMPA-BBB assay was used to predict the passive permeability of the compounds presenting the highest cellular potency (**4**, **6**, **20**, and **21**), and the results were compared against **NU-9** and two ALS-approved drugs, edaravone and riluzole, which are known for their capacity to permeate the BBB (Table 2).

Analysis of our data showed that **NU-9** and derivatives **4**, **6**, **20**, and **21** had lower BBB passive permeability than that of edaravone, riluzole, and verapamil, another drug known for its high BBB permeation. When comparing **NU-9** and its derivatives in their enol tautomeric form, we observed a correlation between the increase in lipophilicity and compound passive permeability (Table 2). To expand our analysis, we calculated their topological polar surface area (TPSA), molecular weight (MW), number of hydrogen bond donors (NHBD), and number of rotatable bonds (NRB), which are physicochemical parameters that, together with lipophilicity, are known to impact the passive permeability of small molecules through physiological membranes such as the BBB [44,45]. We observed that increasing the TPSA together with the NHBD led to a marked loss of permeability; compound **4**, the only cyclohexane-1,3-dione having 2 HBD groups and the highest TPSA, was the only derivative with a permeability as low as theophylline, the negative control used in the assay, suggesting that this compound may not be able to permeate the BBB. These results suggest that, in addition to an increase in lipophilicity, the control of the TPSA and NHBD during subsequent rounds of optimization will be crucial for improving BBB passive permeation.

PAMPA-BBB is routinely used as a first-line assay to predict small-molecule permeability through the BBB. However, despite its extensive use, active transport and the effect of efflux pumps, such as P-glycoprotein (also known as multidrug resistance protein 1 (P-gp/MDR1)) and breast cancer resistance protein (BCRP), on BBB permeability are not assessed in this assay [46]. The metabolic stability is a parameter that critically impacts the pharmacokinetic-pharmacodynamic (PKPD) profile of small-molecules, playing a key role in the amount of bioactive molecules capable of engaging physiological tissues such as the BBB and, therefore, impacting their efficacy [47]. Consequently, the determination of both the effect of efflux pumps on the permeation and the metabolic stability of small molecules are crucial for validating *in vivo* BBB permeability capacity of drugs and efficient brain tissue engagement. Therefore, based on their *in vitro* anti-aggregation activity and passive BBB permeability profile, we selected **6** and **20** for evaluation of their microsomal metabolic stability and the potential effect of P-gp and BCRP efflux pumps on their permeability.

The bidirectional apparent permeability (P_app_) of **6** and **20** with two of the most highly expressed ATP binding cassette (ABC) efflux transporters present on the BBB luminal side, P-gp and BCRP, was assessed using monolayers of MDCK-MRD1 or MDCKII-BCRP (MDR1 knockout and BCRP knock in) cell lines in the presence or absence of zosuquidar (a P-gp inhibitor) or Ko143 (a BCRP inhibitor) (Table 3) [48,49].

Analysis of the efflux data indicates that both **6** and **20** are substrates for both the P-gp and BCRP transporters. Based on the efflux ratio, both compounds have mild substrate specificity for P-gp transporter, but both are more active substrates for the BCRP transporter, with **6** presenting substrate specificity comparable to that of the highly active BCRP substrate dantrolene. This is expected to severely restrict BBB permeability of **6,** despite its good passive permeability potential as assessed by PAMPA-BBB. Efflux of **20** by the MDCKII-BCRP transporter is much less than for **6**, suggesting better BBB permeability for **20**.

We then evaluated the metabolic stability of **6** and **20** in the presence of human and mouse liver microsomes. The microsomal *in vitro* half-life (t_1/2_) of the compounds was determined in the assay and then used to obtain their intrinsic clearance (CL_int_) (Table 4). Compound **20** had a half-life slightly shorter than that of **NU-9** with mouse microsomes but had a much longer half-life with human microsomes. **NU-9** is known to have good bioavailability, BBB permeability, and efficacy *in vivo* mouse models [27]. The better *in vitro* BBB permeability and the greater human microsome stability of **20** than **NU-9** (Tables 2–4) suggest potential higher BBB permeability of **20** *in vivo*, which should translate to higher brain concentration and improved efficacy.

These *in vitro* results also suggest that **6** is expected to have lower bioavailability than **NU-9** and **20** in both species, and the poor *in vitro* BBB permeability profile of **6**, mostly because of its high BCRP efflux, suggests that it may also have lower BBB permeability *in vivo*, which should decrease its *in vivo* efficacy [52,53]. The alkene linker in **6** might be responsible for the considerable loss of metabolic stability of **6** in both models. The cytotoxicity profiles of **NU-9**, **6**, and **20** were evaluated in HepG2 and HEK293 cell lines, two common models to assess small-molecule *in vitro* liver and kidney toxicity, respectively (Table 5 and SI Figs. 3–6) [54–57]. The CellTiter-Glo^®^ luminescent cell viability assay, which measures the amount of ATP produced by viable cells, was used to evaluate the dose-dependent cellular toxicity of the test compounds. All the compounds exhibited only marginal cytotoxic effects in both cell lines at the highest concentration screened in our assay (30 μM), a loss of viability of <10 % in HepG2 and of <18 % in HEK293 cell lines. Additionally, we also observed that all compounds were also protecting the PC12-SOD1^G85R^ cells against MG132-induced toxicity with single-digit micromolar EC_50_s (Table 5 and SI Fig. 7). A comparison of the anti-aggregation and MG132 protection assays results and the cellular cytotoxicity results suggests a good safety window between the active dose and the dose needed to induce liver and kidney toxicity, which are two of the main reasons for drug safety concerns [58,59].

**Fig. 7.**
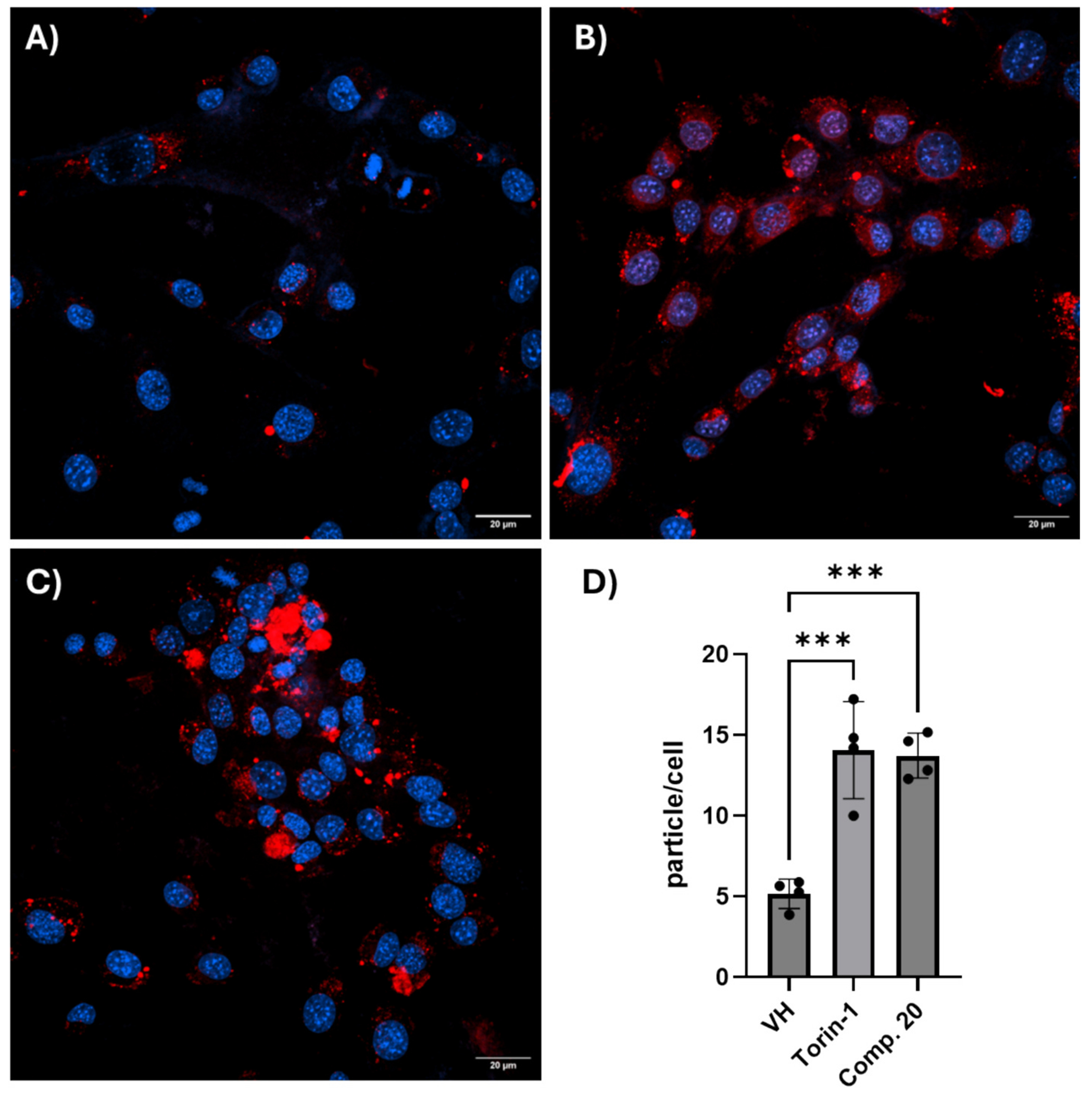
Live cell imaging of HT22 hippocampal cells stained with DAPRed autophagy fluorescence marker (red – representing autophagosome formation) and Hoescht 33,342 nuclear staining after 5 h treatment; (A) DMSO (0.1 % *v*/v), (B) Torin-1 (autophagy inducer, 0.2 μM) and (C) **20** (3 μM). Bafilomycin (0.1 μM) was added in the last 3 h of treatment to block autolysosome formation and promote the accumulation and detection of mature autophagosome formation. Images were acquired using a Nikon SORA. The measurement bars on the right lower corner of the figures have a scale of 20 μm. Graph D represent the mean ± sd of the analysis of 4 images, and the results are presented as the number of DAPRed particles/cell (stained autophagosomes/cell). One-way ANOVA followed by Sidak multiple comparison tests was performed to calculate the *p* values, and the levels of significance are denoted as follows: ns 0.1234, *0.0332, **0.0021, ***0.0002, **** < 0.0001. (For interpretation of the references to colour in this figure legend, the reader is referred to the web version of this article.)

In a recent study, we demonstrated that **NU-9** was capable of reducing Amyloid-beta oligomer build-up tested in primary hippocampal neuronal cells. This suggests that the therapeutic potential of our CHD compounds could be expanded for Alzheimer’s disease and potentially other proteinopathies [60]. In the same work, super resolution microscopy, using an anti-LC3II-GFP antibody, demonstrated that **NU-9** was capable of increasing LC3II puncta in primary hippocampal neurons. These data demonstrated the ability of NU9 to modulate the autophagic flux [60]. In line with our reported findings for **NU-9**, we tested compound 20 in HT22 hippocampal cells for its ability to induce the autophagic flux using the autophagy DAPRed fluorescent dye, a marker of autophagosomes/autolysosomes, and the mTOR inhibitor Torin-1 as a positive control for the assay. We observed that compound **20** treatment was capable of increasing DAPRed fluorescence signal when compared to vehicle, suggesting an increase in the autophagy flux (Fig. 7). Collectively, these observations show that the proposed NU-9 mechanism is extended to the entire CHD compound family, indicating a shared ability to promote autophagy, which may contribute to the compounds’ therapeutic potential in ALS and potentially other proteinopathies. However, further studies are being conducted to validate our initial observations.

## 3. Conclusion

ALS is a fast-progressing and very debilitating disease which was reported by the French neurologist Jean-Martin Charcot more than 100 years ago. However, today the therapeutic approaches available for patients are still limited and only provide marginal symptomatic relief to the patients. Hence, with the continuous increase in worldwide disease incidence, there is great social pressure for the development of more efficient therapies that could decrease the social and economic burdens associated with ALS. Here, we evaluated a series of compounds derived from **NU-9**, a compound with reported anti-aggregation activity *in vitro* and excellent efficacy in the transgenic SOD1^G93A^ and the TDP43^A315T^ mouse models of the disease [27], for their capacity to inhibit mutant SOD1 intracellular aggregation. Additionally, recently we also demonstrated the therapeutic potential of **NU-9** in an *in vitro* model of Alzheimer’s disease and preliminary data suggested the compound’s capacity to modulate the autophagy efflux [60]. Of the compounds tested, **20** was the most promising, which will be further validated in more relevant ALS models, such as patient-derived cells and animal models of the disease. This compound has an *in vitro* cellular potency in the same range of **NU-9**, has a better pharmacological profile, better *in vitro* BBB permeability potential, similar or better metabolic stability in mouse and human microsomes, and a good safety window in the initial cellular cytotoxicity screening, all of which suggest potentially higher *in vivo* efficacy than **NU-9**. Furthermore, our initial screening suggested, in line with **NU-9’s** previous observations, the potential capacity of compound **20** to modulate the autophagy response, which further suggests a common biological pathway for the compound series. Nevertheless, studies are currently being performed by our group to further validate the mode of action of our series of compounds. Additionally, the results of this work provide a basis for the structural attributes that could impact potency and pharmacological properties, which should translate to higher *in vivo* efficacy.

## 4. Experimental section

### 4.1. General chemistry materials and methods

The solvents used in the reactions were analytical grade and obtained from Millipore Sigma and Fisher Scientific. The commercially available reagents and starting materials were purchased from TCI, Ambeed, Millipore Sigma, Fisher Scientific, or Alfa Aesar and used without any purification. The reactions were performed using magnetic stirring plates and monitored by thin-layer chromatography (TLC) using precoated silica gel plates (silica gel 60 F_254_) and UV light (254 nm) for detection. The reaction progression was also monitored by liquid chromatography–mass spectrometry (LC-MS) on a Waters Acquity H Plus UPLC connected to a diode array and a Waters Xevo TQ-S micro triple quadrupole mass spectrometer. The crude compounds were purified by flash column chromatography with prepacked silica gel cartridges (230–400 mesh, 40–63 mm, RediSep Silver – Teledyne ISCO) and a Teledyne ISCO Combiflash RF+ or Combiflash Nextgen 300+ using the solvent mixtures stated for each synthesis as mobile phase.

The ^1^H and ^13^C nuclear magnetic resonance (NMR) spectra were acquired at room temperature on a Bruker Avance III 500 spectrometer (^1^H at 500.1 MHz and ^13^C at 125.8 MHz) equipped with a DCH Cryo-Probe or on a Bruker Avance III HD system (^1^H at 500.1 MHz and ^13^C at 125.8 MHz) equipped with a BBO Prodigy probe. The chemical shifts are expressed in *δ* (ppm) values using residual deuterated solvents as the internal reference (^1^H NMR: DMSO-*d*_6_, *δ* 2.50 ppm; CD_3_OD, *δ* 3.31 ppm; D_2_O, *δ* 4.79 ppm; CDCl_3,_ *δ* 7.26 ppm; ^13^C NMR: DMSO-*d*_6_, *δ* 39.52 ppm; CD_3_OD, *δ* 49.00 ppm; CDCl_3,_ *δ* 77.16 ppm). The signal splitting patterns are described as singlet (s), doublet (d), triplet (t), quartet (q), pentate (p), multiplet (m) or a combination thereof. The coupling constants (*J*) are quoted to the nearest 0.1 Hz. The purity of the final compounds was determined by liquid chromatography–mass spectrometry (LC-MS) on a Waters Acquity H Plus UPLC connected to a diode array and a Waters Xevo TQ-S micro triple quadrupole mass spectrometer using a flow rate of 0.4 mL/min and a gradient elution of acetonitrile (0.1 % formic acid) in water (0.1 % formic acid) over 7 min as follows: 10 % acetonitrile over 30 s, acetonitrile gradient from 10 to 100 % from 30 s to 5 min, acetonitrile 100 % from 5 to 6 min, acetonitrile gradient from 100 to 10 % from 6 to 6.2 min and then 10 % acetonitrile was maintained until the end of the program. The purity of all final compounds was >95 %. The melting points of compounds obtained as a solid were measured using a Buchi Melting Point B-540 instrument. The high-resolution mass spectrometry (HRMS) data of the newly synthesized final compounds were obtained using an Agilent 6545 Q-TOF LC/MS instrument coupled to an Agilent 1290 Infinity II UHPLC system with methanol as solvent and acquired in the positive or negative mode as specified in the protocol. **NU-9**, intermediate compound **(±)-28** and compounds **(±)-11-(±)-17** were synthesized as described elsewhere [25,26,31].

#### 4.1.1. Methyl 3,5-bis((tert-butyldimethylsilyl)oxy)benzoate (23)

To a stirring solution of 3,5-dihydroxymethyl benzoate **22** (1.7 g, 10.0 mmol) in DCM (20 mL) in an ice bath was added imidazole (2.7 g, 40.0 mmol), followed by DMAP (244 mg, 2 mmol). After 10 min, TBSCl (4.5 g, 30 mmol) was added, and the reaction mixture was allowed to stir at rt. for 4 h. The reaction was quenched with a solution (5 mL) of 1 *N* HCl with 3 % LiCl, and the product was extracted with DCM (10 mL, 3×). The organic phase was dried using Na_2_SO_4_, concentrated *in vacuo*, and the crude compound was purified by silica gel flash column chromatography using hexane/EtOAc with a gradient elution from 0 to 100 % EtOAc. The desired product was eluted with 0–10 % EtOAc, to give **23** (3.7 g, 9.3 mmol, 93 %) as a colourless oil. ^1^H NMR (500 MHz, CDCl_3_): *δ* 7.12 (d, *J* = 2.2 Hz, 2H), 6.52 (s, 1H), 3.88 (s, 3H), 0.98 (s, 18H), 0.20 (s, 12H). ^13^C NMR (126 MHz, CDCl_3_): *δ* 166.81, 156.53, 131.83, 116.83, 114.58, 52.16, 25.65, 18.21, −4.43.

#### 4.1.2. (3,5-Bis((tert-butyldimethylsilyl)oxy)phenyl)methanol (24)

To a stirring solution of **23** (3.68 g, 9.3 mmol) in DCM (30 mL) at 0 °C was added a solution of DIBAL-H (20 mL, 20 mmol, 1 M in DCM). After 3 h, the reaction was quenched with Rochelle salt (10 mL) and extracted with DCM (10 mL, 3×). The organic phase was dried using Na_2_SO_4_, concentrated *in vacuo*, and the crude compound was purified by silica gel flash column chromatography using hexane/EtOAc with a gradient elution from 0 to 100 % EtOAc. The desired product was eluted with 10 % EtOAc, to give **24** (3.1 g, 8.4 mmol, 91 %) as a colourless oil. ^1^H NMR (500 MHz, CDCl_3_): *δ* 6.47 (d, *J* = 2.2 Hz, 2H), 6.26 (s, 1H), 4.57 (s, 2H), 0.98 (s, 18H), 0.19 (s, 12H). ^13^C NMR (126 MHz, CDCl_3_): *δ* 156.73, 143.09, 111.77, 111.20, 65.16, 25.69, 18.21, −4.38.

#### 4.1.3. 3,5-Bis((tert-butyldimethylsilyl)oxy)benzaldehyde (25)

To a stirring solution of **24** (1.6 g, 4.4 mmol) in DCM (40 mL) at rt. was added NaOAc (0.1 g, 1.2 mmol), followed by PCC (1.4 g, 6.5 mmol). After 1 h, the reaction was filtered through a plug of silica gel and washed with EtOAc. The combined filtrates were concentrated *in vacuo*, and the crude compound was purified by silica gel flash column chromatography using hexane/EtOAc with a gradient elution from 0 to 100 % EtOAc. The desired product was eluted with 5 % EtOAc, to give aldehyde **25** (1.38 g, 3.8 mmol, 87 %) as a colourless oil. ^1^H NMR (500 MHz, CDCl_3_): *δ* 9.86 (s, 1H), 6.95 (d, *J* = 2.3 Hz, 2H), 6.59 (s, 1H), 0.99 (s, 18H), 0.22 (s, 12H). ^13^C NMR (126 MHz, CDCl_3_): *δ* 191.83, 191.82, 157.27, 138.35, 118.42, 114.39, 25.62, 18.21, −4.42.

#### 4.1.4. 1-(3,5-Bis((tert-butyldimethylsilyl)oxy)phenyl)-2-(3,5-bis(trifluoromethyl)phenyl)ethan-1-ol [(±)-26]

To a stirring solution of **25** (366 mg, 1.0 mmol) in diethyl ether (10 mL) at 0 °C was added dropwise a solution of the Grignard reagent prepared as follows: in an oven-dried 2-neck RBF charged with Mg turnings (48 mg, 2.0 mmol) in diethyl ether (10 mL) at 40 °C was added 3,5-bis(trifluoromethyl)benzyl bromide (0.28 mL, 1.5 mmol) in two portions, the second after the reaction mixture became turbid, and allowed to reflux until most of the Mg was consumed. After the addition of the Grignard reagent was complete, the reaction mixture was allowed to warm to rt. and stirred overnight. The reaction was quenched with sat. aq. NH_4_Cl (5 mL) and 3 *N* HCl (5 mL), and extracted with diethyl ether (10 mL, 3×). The organic phase was dried (NaSO_4_) and concentrated *in vacuo*. The crude compound was purified by silica gel flash column chromatography using hexane/EtOAc with a gradient elution from 0 to 100 % EtOAc. The desired product was eluted with 10 % EtOAc, to give **(±)-26** (853 mg, 0.98 mmol, 98 %) as a colourless oil. ^1^H NMR (500 MHz, CDCl_3_): *δ* 7.72 (s, 1H), 7.63–7.57 (m, 2H), 6.38 (d, *J* = 2.2 Hz, 2H), 6.25 (s, 1H), 4.82 (dd, *J* = 7.6, 5.2 Hz, 1H), 3.19–3.02 (m, 2H), 0.96 (s, 18H), 0.15 (d, *J* = 1.1 Hz, 12H). ^13^C NMR (126 MHz, CDCl_3_): *δ* 156.83, 145.18, 140.57, 131.32 (q, *J*_CF_ = 32.8 Hz), 129.80, 123.37 (q, *J*_CF_ = 273.4 Hz), 120.47, 111.64, 110.79, 74.54, 44.89, 25.63, 18.19, −4.49.

#### 4.1.5. 5-(2-(3,5-Bis(trifluoromethyl)phenyl)-1-hydroxyethyl)benzene-1,3-diol [(±)-27]

To a stirring solution of **(±)-26** (500 mg, 0.84 mmol) in MeOH (10 mL) was added HCl (0.2 mL, 2.5 mmol, 12.8 M). After 12 h, the solvent was concentrated *in vacuo*, and the residue was purified by silica gel flash column chromatography using hexane/EtOAc with a gradient elution from 0 to 100 % EtOAc. The desired product was eluted with 60–70 % EtOAc, to give **(±)-27** (271 mg, 0.74 mmol, 88 %) as a white solid, mp: 206–207 °C. ^1^H NMR (500 MHz, DMSO): *δ* 9.11 (s, 2H), 7.89 (s, 3H), 6.24 (d, *J* = 2.2 Hz, 2H), 6.09 (t, *J* = 2.2 Hz, 1H), 5.27 (d, *J* = 4.6 Hz, 1H), 4.62 (dt, *J* = 8.7, 4.3 Hz, 1H), 3.05 (dd, *J* = 13.6, 4.0 Hz, 1H), 2.94 (dd, *J* = 13.6, 8.8 Hz, 1H). ^13^C NMR (126 MHz, DMSO): *δ* 158.55, 147.87, 143.35, 130.88, 130.85, 129.97 (q, *J*_CF_ = 32.8 Hz), 124.01 (q, *J*_CF_ = 273.4 Hz), 119.99 (p, *J*_CF_ = 3.8 Hz), 104.37, 101.48, 73.10, 44.86.

#### 4.1.6. 5-(2-(3,5-Bis(trifluoromethyl)phenyl)-1-hydroxyethyl)cyclohexane-1,3-dione [(±)-4]

To a stirring solution of NaOH (26 mg, 0.66 mmol) in water (5 mL) was added **(±)-27** (80 mg, 0.22 mmol), followed by a Rh/Al_2_O_3_ catalyst (10 mg). The reaction mixture was stirred until a clear solution was obtained, and then the reaction was heated at 90 °C under a hydrogen balloon for 3 days. The reaction was cooled and acidified with 3 N HCl (1 mL) and extracted with EtOAc (10 mL, 3×). The dried (Na_2_SO_4_) extract was concentrated *in vacuo*, and the crude compound was purified by silica gel flash column chromatography using hexane/EtOAc with a gradient elution from 0 to 100 % EtOAc. The desired product was eluted with 10 % EtOAc, to give **(±)-4** (77 mg, 0.21 mmol, 96 %) as a colourless solid; mp: 151–152 °C. ^1^H NMR (500 MHz, DMSO): *δ* 11.09 (s, 1H), 7.95 (d, *J* = 1.8 Hz, 2H), 7.89 (s, 1H), 5.21 (s, 1H), 4.87 (s, 1H), 3.66–3.56 (m, 1H), 2.99 (dd, *J* = 13.8, 2.8 Hz, 1H), 2.74 (dd, *J* = 13.7, 9.9 Hz, 1H), 2.44–2.13 (m, 4H), 2.13–1.99 (m, 1H). ^13^C NMR (126 MHz, DMSO): *δ* 144.19, 130.68 (q, *J*_CF_ = 3.8 Hz), 130.18 (q, *J*_CF_ = 32.8 Hz), 124.01 (q, *J*_CF_ = 273.4 Hz), 119.95 (p, *J*_CF_ = 3.8 Hz), 103.87, 73.24. HRMS (ESI-TOF) *m*/*z*: calculated for C_16_H_14_F_6_O_3_ [M - H]^−^ 367.0774, found 367.0787.

#### 4.1.7. (1R,3S)-5-((R)-2-(3,5-Bis(trifluoromethyl)phenyl)-1-hydroxyethyl)cyclohexane-1,3-diol [(±)-5]

To a stirring solution of compound **(±)-28** (525 mg, 0.88 mmol) in MeOH (10 mL) at 0 °C was added HCl (0.2 mL, 2.53 mmol, 12.8 M). After 6 h, the reaction was concentrated *in vacuo*, and the residue was washed with diethyl ether to give a colourless solid of **(±)-5** (271 mg, 0.73 mmol, 83 %); mp: 184–186 °C. ^1^H NMR (500 MHz, CD_3_OD): *δ* 7.86 (s, 2H), 7.78 (s, 1H), 3.59 (dtd, *J* = 11.3, 7.6, 4.0 Hz, 3H), 3.03 (dd, *J* = 13.9, 3.0 Hz, 1H), 2.80 (dd, *J* = 13.9, 9.9 Hz, 1H), 2.24 (ddt, *J* = 11.0, 4.4, 2.3 Hz, 1H), 2.10 (d, *J* = 12.2 Hz, 1H), 1.94 (dd, *J* = 12.1, 4.2 Hz, 1H), 1.50 (ddt, *J* = 12.5, 5.2, 3.3 Hz, 1H), 1.25–1.12 (m, 2H), 1.12–1.00 (m, 1H). ^13^C NMR (126 MHz, CD_3_OD): *δ* 143.38, 130.94 (q, *J*_CF_ = 34.0 Hz), 129.67, 123.63 (q, *J*_CF_ = 272.2 Hz), 119.38 (p, *J*_CF_ = 3.8 Hz), 74.87, 67.81, 67.75, 44.06, 39.94, 39.13, 37.28, 35.57. HRMS (ESI-TOF) m/z: calculated for C_16_H_18_F_6_O_3_ [M − H]^−^ 317.1087, found 371.1104; calculated for C_16_H_18_F_6_O_3_ [M + Cl]^−^ 407.0854, found 407.0870; calculated for C_16_H_18_F_6_O_3_ [M + HCOO]^−^ 417.1142, found 417.1161.

#### 4.1.8. N-((1R,3R,5R,7S)-7-(3,5-Bis(trifluoromethyl)benzyl)-6-oxabicyclo[3.2.1]octan-3-yl)-2-methylpropane-2-sulfinamide (30) and N-((1R,3S,5R,7S)-7-(3,5-Bis(trifluoromethyl)benzyl)-6-oxabicyclo[3.2.1]octan-3-yl)-2-methylpropane-2-sulfinamide (31)

To a stirring solution of ketone **(±)-12** (100 mg, 0.284 mmol) in THF (5 mL) was added *(S)-tert*-butanesulfinamide (41 mg, 0.341 mmol), followed by Ti(*i*-OPr)_4_ (1 mL). After refluxing for 12 h, the reaction was cooled to rt. and quenched with sat. brine, passed through a celite plug and washed with ethyl acetate (20 mL). The organic layer was separated, and the aqueous one was extracted with ethyl acetate (10 mL, 3×). The combined organic layers were dried (Na_2_SO_4_), and the solvent was concentrated *in vacuo*. The residue was purified by silica gel chromatography, eluting with 50 % EtOAc / hexanes to give a partially separable diastereomeric mixture of the desired product **29** (61 mg, 0.134 mmol, 47 %), which was used in the next step without further purification. To a stirring solution of **29** (50 mg, 0.11 mmol) in THF was added NaBH_4_ (50 mg, 1.32 mmol). After stirring for 2 h at rt., the reaction was filtered through a celite plug and the filtrate was concentrated *in vacuo*. The residue was purified by silica gel flash column chromatography using hexane/EtOAc with a gradient elution from 0 to 100 % EtOAc. The desired products were eluted with 50 % EtOAc, to give **30** (17 mg, 0.037 mmol, 34 %) followed by **31** (21 mg, 0.046 mmol, 42 %), both as colourless oils.

**30**; ^1^H NMR (500 MHz, CDCl_3_) *δ* 7.69 (s, 3H), 4.78 (dd, *J* = 9.7, 3.9 Hz, 1H), 4.55–4.47 (m, 1H), 4.30 (d, *J* = 9.6 Hz, 1H), 3.63 (dt, *J* = 11.1, 6.0 Hz, 1H), 2.84 (dd, *J* = 14.3, 3.9 Hz, 1H), 2.75 (dd, *J* = 14.2, 9.7 Hz, 1H), 2.46 (ddt, *J* = 14.8, 4.0, 1.9 Hz, 1H), 2.30 (q, *J* = 3.7 Hz, 1H), 2.10 (dtd, *J* = 11.3, 4.6, 2.3 Hz, 1H), 2.03–1.94 (m, 1H), 1.91 (ddd, *J* = 14.7, 5.8, 2.9 Hz, 1H), 1.78 (dd, *J* = 14.5, 6.8 Hz, 1H), 1.55 (d, *J* = 11.6 Hz, 1H), 1.13 (d, *J* = 3.2 Hz, 9H). ^13^C NMR (126 MHz, CDCl_3_) *δ* 141.56, 131.39 (q, *J*_CF_ = 32.8 Hz), 123.44 (q, *J*_CF_ = 272.6 Hz), 120.23 (p, *J*_CF_ = 3.8 Hz), 81.58, 76.30, 55.71, 50.92, 42.01, 39.09, 38.28, 37.21, 35.07, 22.42.

**31**; ^1^H NMR (500 MHz, CDCl_3_): *δ* 7.73 (s, 1H), 7.65 (d, *J* = 1.6 Hz, 2H), 4.45 (t, *J* = 5.4 Hz, 1H), 4.14 (ddd, *J* = 12.0, 8.0, 5.5 Hz, 1H), 3.68–3.54 (m, 1H), 2.96 (dd, *J* = 21.1, 7.3 Hz, 1H), 2.85–2.71 (m, 2H), 2.29 (tt, *J* = 4.6, 2.2 Hz, 1H), 2.25–2.16 (m, 1H), 2.07 (dddt, *J* = 15.1, 6.6, 4.5, 2.3 Hz, 1H), 2.00–1.92 (m, 1H), 1.53 (d, *J* = 11.6 Hz, 1H), 1.49–1.36 (m, 1H), 1.35–1.22 (m, 1H), 1.16 (d, *J* = 3.2 Hz, 9H). ^13^C NMR (126 MHz, CDCl_3_): *δ* 142.09, 141.03, 141.01, 131.52 (q, *J*_CF_ = 32.8 Hz), 129.49, 123.39 (q, *J*_CF_ = 272.2 Hz), 120.47 (p, *J*_CF_ = 3.8 Hz), 82.41, 82.31, 75.85, 82.31, 82.41, 75.85, 55.55, 50.94, 50.23, 42.43, 41.29, 40.64, 39.97, 39.02, 38.79, 35.79, 22.51, 22.48.

#### 4.1.9. General protocol for the synthesis of amine salts 18 and 19

To a stirring solution of the respective sulfinamide derivative in MeOH (5 mL) was added conc. HCl (0.1 mL) at rt. After 6 h stirring, the solvent was evaporated, and the obtained residue was washed with ether (10 mL) to give the corresponding final compounds as amine salts.

##### 4.1.9.1. (1R,3R,5R,7S)-7-(3,5-Bis(trifluoromethyl)benzyl)-6-oxabicyclo[3.2.1]octan-3-aminium chloride (18).

Following the general protocol for the synthesis of amine salts **18** and **19,** and using sulfinamide **30** (17 mg, 0.037 mmol). The final compound **18** (12 mg, 0.031 mmol, 86 %) was obtained as a colourless solid; mp: 158–161 °C. ^1^H NMR (500 MHz, CD_3_OD): *δ* 7.88 (d, *J* = 1.8 Hz, 2H), 7.82 (s, 1H), 4.58 (ddd, *J* = 5.9, 4.2, 1.5 Hz, 1H), 4.23 (dd, *J* = 7.8, 5.7 Hz, 1H), 3.53 (t, *J* = 6.8 Hz, 1H), 3.00–2.85 (m, 2H), 2.37 (q, *J* = 3.7 Hz, 1H), 2.29 (dtd, *J* = 11.6, 4.6, 2.3 Hz, 1H), 2.22 (ddd, *J* = 15.6, 7.2, 3.7 Hz, 1H), 2.02–1.85 (m, 3H), 1.70 (d, *J* = 12.0 Hz, 1H). ^13^C NMR (126 MHz, CD_3_OD): *δ* 142.02, 131.09 (q, *J*_CF_ = 32.8 Hz), 129.86, 123.57 (q, *J*_CF_ = 272.2 Hz), 119.71 (p, *J*_CF_ = 3.8 Hz), 83.40, 74.98, 44.75, 41.17, 37.43, 34.57, 33.04, 32.58. HRMS (ESI-TOF) *m*/*z*: calculated for C_16_H_18_ClF_6_NO [M-HCl + H]^+^ 353.1314, found 354.1352.

##### 4.1.9.2. (1R,3S,5R,7S)-7-(3,5-Bis(trifluoromethyl)benzyl)-6-oxabicyclo[3.2.1]octan-3-aminium chloride (19).

Following the general protocol for the synthesis of amine salts **18** and **19,** and using sulfinamide **31** (21 mg, 0.046 mmol). The final compound **19** (14 mg, 0.036 mmol, 78 %) was obtained as a colourless solid; mp: 171–173 °C. ^1^H NMR (500 MHz, CD_3_OD): *δ* 7.84 (d, *J* = 25.8 Hz, 3H), 4.58–4.45 (m, 1H), 4.19 (d, *J* = 6.7 Hz, 1H), 3.46 (s, 1H), 2.90 (d, *J* = 6.7 Hz, 2H), 2.44 (s, 1H), 2.24–2.06 (m, 3H), 1.72–1.53 (m, 2H), 1.43 (t, *J* = 12.1 Hz, 1H). ^13^C NMR (126 MHz, CD_3_OD): *δ* 142.13, 129.70, 119.66, 82.48, 74.98, 45.33, 41.23, 38.36, 36.13, 34.50, 34.12. HRMS (ESI-TOF) m/z: calculated for C_16_H_18_ClF_6_NO [M -HCl + H]^+^ 353.1314, found 354.1344.

#### 4.1.10. (E)-3-(3,5-Bis(trifluoromethyl)phenyl)acrylaldehyde (33)

To a stirring solution of 3,5-bis(trifluoromethyl)benzaldehyde **32** (1.2 g, 5.0 mmol, 0.8 mL) in THF (10 mL) at rt. was added the (triphenylphosphoranylidene)acetaldehyde (1.8 g, 5.9 mmol). After 3 h, the reaction mixture was concentrated *in vacuo* and the crude compound was purified by silica gel flash column chromatography using hexane/EtOAc with a gradient elution from 0 to 100 % EtOAc. The desired product was eluted with 10–15 % EtOAc, to give **33** (1.01 g, 4.0 mmol, 81 %) as a white solid; mp 83–86 °C. ^1^H NMR (500 MHz, CDCl_3_): *δ* 9.78 (d, *J* = 7.3 Hz, 1H), 8.04–7.96 (m, 2H), 7.97–7.89 (m, 1H), 7.53 (d, *J* = 16.1 Hz, 1H), 6.83 (dd, *J* = 16.1, 7.3 Hz, 1H). ^13^C NMR (126 MHz, CDCl_3_): *δ* 192.44, 147.93, 136.08, 132.77 (q, *J*_CF_ = 34.0 Hz), 131.46, 127.94 (q, *J*_CF_ = 3.8 Hz), 124.21 (p, *J*_CF_ = 3.8 Hz), 122.87 (q, *J*_CF_ = 273.4 Hz).

#### 4.1.11. (3E,5E)-6-(3,5-Bis(trifluoromethyl)phenyl)hexa-3,5-dien-2-one (34)

To a stirring solution of enal **33** (1.0 g, 3.7 mmol) in THF (10 mL) at rt. was added 1-(triphenylphosphoranylidene)-2-propanone (1.9 g, 6.0 mmol). After 12 h, the reaction mixture was concentrated *in vacuo* and the crude compound was purified by silica gel flash column chromatography using hexane/EtOAc with a gradient elution from 0 to 100 % EtOAc. The desired product was eluted with 10–20 % EtOAc, to give **34** (1.01 g, 3.5 mmol, 94 %) as a white solid; mp 98.2–99.1 °C. ^1^H NMR (500 MHz, CDCl_3_): *δ* 8.15 (s, 2H), 8.07 (s, 1H), 7.62–7.46 (m, 1H), 7.36–7.17 (m, 2H), 6.65 (d, *J* = 15.5 Hz, 1H), 2.62 (s, 3H). ^13^C NMR (126 MHz, CDCl_3_): *δ* 198.00, 141.38, 138.02, 137.09, 132.62, 132.33 (q, *J*_CF_ = 34.0 Hz), 130.24, 126.73 (d, *J*_CF_ = 3.8 Hz), 123.02 (q, *J*_CF_ = 248.2 Hz), 122.15 (p, *J*_CF_ = 3.8 Hz), 27.76.

#### 4.1.12. Diethyl (E)-2-(1-(3,5-bis(trifluoromethyl)phenyl)-5-oxohex-1-en-3-yl)malonate [(±)-35]

To a stirring solution of dienone **34** (320 mg, 1.04 mmol) in toluene (10 mL) at rt. was added sequentially LiI (26 mg, 0.2 mmol), diethylmalonate (0.2 mL, 1.3 mmol), and Et_3_N (0.2 mL, 1.4 mmol). After 3 h, the reaction was quenched with water (10 mL) and extracted with EtOAc (10 mL, 3×). The organic phase was dried (NaSO_4_), concentrated *in vacuo*, and the crude compound was purified by silica gel flash column chromatography using hexane/EtOAc with a gradient elution from 0 to 100 % EtOAc. The desired product was eluted with 40–50 % EtOAc, to give **(±)-35** (441 mg, 0.94 mmol, 94 %) as a colourless oil. ^1^H NMR (500 MHz, CDCl_3_): *δ* 7.71 (s, 3H), 6.56 (d, *J* = 15.9 Hz, 1H), 6.37 (dd, *J* = 15.9, 8.9 Hz, 1H), 4.26–4.13 (m, 4H), 3.64 (d, *J* = 7.1 Hz, 1H), 3.58–3.49 (m, 1H), 2.88–2.74 (m, 2H), 2.15 (s, 3H), 1.24 (dt, *J* = 16.9, 7.1 Hz, 6H). ^13^C NMR (126 MHz, CDCl_3_): *δ* 205.84, 167.96, 138.83, 132.86, 131.9 (q, *J*_CF_ = 32.8 Hz), 130.04, 126.14 (d, *J*_CF_ = 3.8 Hz), 123.26 (q, *J*_CF_ = 273.4 Hz), 120.96 (p, *J*_CF_ = 3.8 Hz), 61.69, 61.58, 55.00, 45.58, 37.85, 30.40, 14.07.

#### 4.1.13. (E)-5-(3,5-Bis(trifluoromethyl)styryl)cyclohexane-1,3-dione (6)

To a stirring solution of ketone **(±)-35** (330 mg, 0.7 mmol) in ethanol (4 mL) at rt. was added NaOEt [32 mg Na metal, 1.4 mmol, in ethanol (3 mL)]. After 4 h, a solution of 2 *N* NaOH (5 mL) was added with stirring. Then, after 12 h stirring, the reaction mixture was concentrated *in vacuo*, the obtained residue was solubilized in water (10 mL), and the solution was acidified with 3 *N* HCl. The resulting mixture was refluxed for 3 h and then concentrated *in vacuo*. The crude compound was purified by silica gel flash column chromatography using hexane/EtOAc with a gradient elution from 0 to 100 % EtOAc. The desired product was eluted with 75–100 % EtOAc, to give the alkene-containing CHD **6** (197 mg, 0.6 mmol, 80 %) as a white solid; mp 191–192 °C. ^1^H NMR (500 MHz, DMSO): *δ* 11.20 (s, 1H), 8.11 (d, *J* = 1.7 Hz, 2H), 7.90 (s, 1H), 6.74 (dd, *J* = 16.1, 6.2 Hz, 1H), 6.67 (d, *J* = 16.2 Hz, 1H), 5.25 (s, 1H), 2.95 (tq, *J* = 10.7, 5.3 Hz, 1H), 2.39 (td, *J* = 17.0, 7.6 Hz, 4H). ^13^C NMR (126 MHz, DMSO): *δ* 140.28,137.82, 131.07 (q, *J*_CF_ = 32.8 Hz), 127.08, 126.97, 126.93, 126.76, 123.83 (q, *J*_CF_ = 273.4 Hz), 120.66 (p, *J*_CF_ = 3.8 Hz), 120.57, 36.86. HRMS (ESI-TOF) *m*/*z*: calculated for C_16_H_12_F_6_O_2_ [M - H]^−^ 349.0669, found 349.0686.

#### 4.1.14. 3-Methoxycyclopent-2-en-1-one (43)

To a stirring solution of cyclopentanedione **42** (961 mg, 10.0 mmol) in MeOH (20 mL) was added a catalytic amount of iodine (152 mg, 0.6 mmol). The reaction mixture was stirred at rt. for 2 days, concentrated *in vacuo*, and the residue was purified by silica gel flash column chromatography using hexane/EtOAc with a gradient elution from 0 to 100 % EtOAc. The desired product was eluted with 50 % EtOAc, to give **43** (783 mg, 7.0 mmol, 71 %) as a colourless solid. mp: 61–62 °C. The NMR data were consistent with the reported literature data.[61] ^1^H NMR (500 MHz, CDCl_3_): *δ* 5.28 (t, *J* = 1.2 Hz, 1H), 3.80 (s, 3H), 2.62–2.53 (m, 2H), 2.45–2.37 (m, 2H). ^13^C NMR (126 MHz, CDCl_3_) *δ* 205.90, 191.23, 104.52, 58.77, 34.23, 28.29.

#### 4.1.15. 5-(3,5-Bis(trifluoromethyl)benzyl)-3-methoxycyclopent-2-en-1-one (7)

To a stirring solution of lithium diisopropylamide (4.3 mL, 4.4 mmol, 1 M) in THF (15 mL) at −78 °C was added a solution of **43** (0.40 g, 4.3 mmol) in THF (10 mL) dropwise over 10 min. The reaction mixture was stirred at −78 ° C for 30 min, followed by the dropwise addition of a solution of 3,5-bis(trifluoromethyl)benzyl bromide (1.2 g, 0.7 mL, 3.9 mmol) in THF (10 mL). The reaction mixture was stirred at −78 °C for an additional 1 h and then allowed to reach room temperature gradually. The reaction was quenched with sat. NH_4_Cl solution (10 mL), and the product was extracted with ethyl acetate (30 mL, 3×). The organic layers were combined, dried (NaSO_4_), concentrated *in vacuo*, and purified by silica gel flash column chromatography using hexane/EtOAc with a gradient elution from 0 to 100 % EtOAc. The desired product was eluted with 50 % EtOAc to give **7** (457 mg, 1.4 mmol, 38 %) as a white solid; mp: 100–101 °C. ^1^H NMR (500 MHz, CDCl_3_): *δ* 7.74 (s, 1H), 7.68–7.63 (m, 2H), 5.32 (s, 1H), 3.83 (s, 3H), 3.38–3.28 (m, 1H), 2.84 (dddd, *J* = 10.0, 6.9, 4.0, 3.0 Hz, 1H), 2.77 (dd, *J* = 13.8, 9.9 Hz, 1H), 2.67 (ddd, *J* = 17.7, 7.2, 1.2 Hz, 1H), 2.27 (ddd, *J* = 17.7, 3.1, 1.1 Hz, 1H). ^13^C NMR (126 MHz, CDCl_3_): *δ* 205.54, 189.72, 141.96, 131.76 (q, *J*_CF_ = 34.0 Hz), 129.05 (q, *J*_CF_ = 5.0 Hz), 123.28 (q, *J*_CF_ = 272.2 Hz), 120.61 (p, *J*_CF_ = 3.8 Hz), 103.68, 58.86, 46.22, 36.68, 33.79. HRMS (ESI-TOF) *m*/*z*: calculated for C_15_H_12_F_6_O_2_ [M + H]^+^ 339.0814, found 339.0829; calculated for C_15_H_12_F_6_O_2_ [M + Na]^+^ 361.0634, found 361.0648.

#### 4.1.16. 4-(3,5-Bis(trifluoromethyl)benzyl)cyclopentane-1,3-dione (8)

To a stirring solution of **7** (150 mg, 0.11 mmol) in THF (10 mL) at rt. was added conc. HCl (0.2 mL, 2.31 mmol, 12.1 M). The reaction mixture was heated at 70 °C for 1 h, after which time the reaction mixture was allowed to cool to rt., diluted with water (5 mL), and neutralized with sat. NaHCO_3_, and the product was extracted with diethyl ether (10 mL, 3×). The organic layer was dried (NaSO_4_) and concentrated. The resulting residue was washed with hexanes and recrystallized from diethyl ether to give **8** (102 mg, 0.31 mmol, 75 %) as a white solid; mp: 159–161 °C. ^1^H NMR (500 MHz, CD_3_OD): *δ* 7.86 (d, *J* = 1.7 Hz, 2H), 7.78 (s, 1H), 3.26 (dd, *J* = 13.4, 3.8 Hz, 1H), 2.94–2.63 (m, 2H), 2.37 (dd, *J* = 17.2, 7.1 Hz, 1H), 1.99 (dd, *J* = 17.3, 2.7 Hz, 1H). ^13^C NMR (126 MHz, CD_3_OD): *δ* 206.40, 204.70, 143.46, 131.04 (q, *J*_CF_ = 32.8 Hz), 129.44, 123.56 (q, *J*_CF_ = 272.2 Hz), 119.50 (p, *J*_CF_ = 3.8 Hz), 37.90, 36.92. HRMS (ESI-TOF) m/z: calculated for C_14_H_10_F_6_O_2_ [M - H]^−^ 323.0512, found 323.0528.

#### 4.1.17. 3,5-Dimethoxypyridine (45)

To an oven-dried round-bottom flask was added methanol (20 mL), which was allowed to cool in an ice bath, then small pieces of sodium metal (2.9 g, 126.58 mmol) were added portion-wise. After the sodium completely reacted, the solvent was removed under vacuum, and 3,5-dibromopyridine **44** (5.0 g, 21.10 mmol) was added, followed by CuBr (0.9 g, 6.33 mmol) and DMF (10 mL). The reaction mixture was heated at 90 °C and stirred for 12 h. The reaction was allowed to cool to room temperature, filtered, and washed with ethyl acetate (30 mL). Saturated NaHCO_3_ was added to the filtrate, and the mixture was extracted with ethyl acetate (30 mL, 3×). The organic phase was dried using Na_2_SO_4_, concentrated *in vacuo*, and the crude compound was purified by silica gel flash column chromatography using hexane/EtOAc with a gradient elution from 0 to 100 % EtOAc. The desired product was eluted with 40–50 % EtOAc, to give **45** (3.77 g, 19.93 mmol, 94 %) as a colourless oil. The NMR data were consistent with the literature-reported data [62]. ^1^H NMR (500 MHz, CDCl_3_): *δ* 7.94 (dd, *J* = 2.5, 1.3 Hz, 2H), 6.79–6.66 (m, 1H), 3.84 (d, *J* = 1.4 Hz, 6H). ^13^C NMR (126 MHz, CDCl_3_): *δ* 156.50, 129.58, 106.69, 55.71.

#### 4.1.18. General protocol for the synthesis of compounds 46 and 47

To a stirring solution of 3,5-dimethoxypyridine **45** in DCM was added 3,5-bis(trifluoromethyl)benzyl bromide or 1-(2-bromoethyl)-3,5-bis(trifluoromethyl)benzene (2.0 equiv.) in a sealed tube, and the mixture was heated at 70 °C over 24 h. The reaction mixture was cooled to room temperature, the solvent was evaporated, and the residue was redissolved in MeOH (1 mL). The final product was precipitated by the addition of diethyl ether as a white precipitate that was collected and washed with diethyl ether to provide compounds **46** and **47**.

##### 4.1.18.1. 1-(3,5-Bis(trifluoromethyl)benzyl)-3,5-dimethoxypyridin-1-ium bromide (**46**).

Following the general methodology for the synthesis of compounds **46** and **47,** and using 3,5-dimethoxypyridine **45** (140 mg, 1.0 mmol) in DCM (1 mL) and 3,5-bis(trifluoromethyl)benzyl bromide (0.37 mL, 2.0 mmol). The desired Compound **46** (371 mg, 0.8 mmol, 83 %) was obtained as a white solid; mp: 164–165 °C. ^1^H NMR (500 MHz, D_2_O): *δ* 8.26 (d, *J* = 2.2 Hz, 2H), 8.17 (s, 1H), 8.02 (s, 2H), 7.67 (d, *J* = 2.1 Hz, 1H), 5.85 (s, 2H), 3.97 (s, 6H). ^13^C NMR (126 MHz, D_2_O): *δ* 159.87, 159.85, 159.79, 134.87, 131.84 (q, *J*_CF_ = 34.0 Hz), 129.22, 125.55, 124.14, 122.94 (q, *J*_CF_ = 272.2 Hz), 115.14, 115.08, 65.98, 63.62, 63.55, 57.24, 14.02.

##### 4.1.18.2. 1-(3,5-Bis(trifluoromethyl)phenethyl)-3,5-dimethoxypyridin-1-ium bromide (47).

Following the general methodology for the synthesis of compounds **46** and **47,** and using 3,5-dimethoxypyridine **45** (0.50 g, 3.7 mmol) in DCM (5 mL) and 1-(2-bromoethyl)-3,5-bis(trifluoromethyl)benzene (1.78 mL, 5.5 mmol). The desired Compound **47** (1.4 g, 2.9 mmol, 79 %) was obtained as a white solid; mp: 187–188 °C. ^1^H NMR (500 MHz, D^2^O): *δ* 7.97 (s, 1H), 7.90 (d, *J* = 2.3 Hz, 2H), 7.58 (d, *J* = 14.8 Hz, 3H), 3.99 (s, 1H), 3.82 (s, 6H), 3.42 (s, 2H). ^13^C NMR (126 MHz, D_2_O): *δ* 160.36, 159.32, 138.14, 131.26 (q, *J*_CF_ = 34.0 Hz), 129.35, 125.69, 125.05, 123.09 (q, *J*_CF_ = 272.2 Hz), 121.71, 117.35, 114.98, 62.78, 57.60, 57.11, 35.98.

#### 4.1.19. 1-(3,5-Bis(trifluoromethyl)benzyl)-3,5-dihydroxypyridin-1-ium bromide (48)

In a high-pressure tube was added **46** (222 mg, 0.5 mmol) in HBr (1.5 mL, 48 %) at rt., the tube was sealed, and the reaction mixture was heated at 120 °C over 24 h. The reaction was allowed to gradually reach rt., the formed crystals were collected and washed with diethyl ether to afford diphenol **48** (173 mg, 0.4 mmol, 83 %) as pale-yellow crystals; mp: 203–205 °C. ^1^H NMR (500 MHz, D_2_O): *δ* 8.16 (s, 1H), 8.04–7.94 (m, 4H), 7.35 (t, *J* = 2.2 Hz, 1H), 5.75 (s, 2H). ^13^C NMR (126 MHz, D_2_O): *δ* 157.72, 135.02, 131.80 (q, *J*_CF_ = 34.0 Hz), 129.25, 125.34, 124.04, 121.88, 118.02, 63.19.

#### 4.1.20. 1-(3,5-bis(trifluoromethyl)phenethyl)-3,5-dihydroxypyridin-1-ium bromide (49)

In a high-pressure tube was added **47** (459 mg, 1.0 mmol) in HBr (3 mL, 62 %) at rt. The tube was sealed, and the reaction mixture was heated at 120 °C over 48 h. The reaction was gradually allowed to cool to rt. The solvent was evaporated using a stream of N_2_ gas, and the residue was washed with ether and filtered to afford diphenol **49** (402 mg, 0.9 mmol, 93 %) as an orange ppt; mp: 195–197 °C. ^1^H NMR (500 MHz, D_2_O): *δ* 7.95 (s, 1H), 7.62–7.54 (m, 4H), 7.24 (d, *J* = 2.3 Hz, 1H), 4.65 (t, *J* = 6.5 Hz, 2H), 3.37 (t, *J* = 6.5 Hz, 2H). ^13^C NMR (126 MHz, D_2_O): *δ* 158.63, 157.11, 138.21, 131.21 (q, *J*_CF_ = 32.8 Hz), 129.25, 125.09, 124.98, 123.13 (q, *J*_CF_ = 272.2 Hz), 121.69, 120.63, 117.39, 62.40, 35.91.

#### 4.1.21. General protocol for the synthesis of piperidine-3,5-diones 9 and 10

To a stirring solution of sodium hydroxide in water was added the respective diphenol intermediate (500 mg, 1.2 mmol) and the mixture was stirred until the reaction became clear. Rh/Al_2_O_3_ catalyst was added to the reaction mixture and heated at 75 °C under a hydrogen balloon for 12 h. The reaction mixture was cooled to rt., filtered through a plug of Celite, and washed with water (2 mL). The aqueous filtrate was transferred to a separatory funnel, ethyl acetate (20 mL) was added, then a 2 *N* aq. HCl solution was also added dropwise while extracting the free amine with ethyl acetate (30 mL, 3×) until the pH was acidic. The organic layers were combined, dried (NaSO_4_) and concentrated *in vacuo*. The crude compound was purified by silica gel flash column chromatography using hexane/EtOAc with a gradient elution from 0 to 100 % EtOAc.

##### 4.1.21.1. 1-(3,5-Bis(trifluoromethyl)benzyl)piperidine-3,5-dione (9).

Following the general protocol for the synthesis of piperidine-3,5-diones derivatives and using sodium hydroxide (240 mg, 6.0 mmol) in water (5 mL), diphenol **48** (500 mg, 1.2 mmol) and Rh/Al_2_O_3_ catalyst (50 mg). The desired product was eluted with 40 % EtOAc, to give **9** (341 mg, 1.0 mmol, 84 %) as a white solid; mp: 85–86 °C. ^1^H NMR (500 MHz, CD_3_OD): *δ* 7.99 (d, *J* = 1.7 Hz, 2H), 7.91 (s, 1H), 3.88 (s, 2H), 3.22 (s, 4H), 0.00 (s, 2H). ^13^C NMR (126 MHz, CD_3_OD) *δ* 140.69, 131.50 (q, *J*_CF_ = 32.8 Hz), 129.17, 123.44 (q, *J*_CF_ = 273.4 Hz), 121.09 (p, *J*_CF_ = 3.8 Hz), 59.08, 56.09. HRMS (ESI-TOF) *m*/*z*: calculated for C_14_H_11_F_6_NO_2_ [M − H]^−^ 338.0621, found 338.0637.

##### 4.1.21.2. 1-(3,5-Bis(trifluoromethyl)phenethyl)piperidine-3,5-dione (10).

Following the general methodology for the synthesis of piperidine-3,5-diones derivatives and using sodium hydroxide (278 mg, 7.0 mmol) in water (5 mL), diphenol **49** (600 mg, 1.4 mmol) and Rh/Al_2_O_3_ catalyst (60 mg). The desired product was eluted with 40 % EtOAc, to give **10** (172 mg, 0.5 mmol, 35 %) as a white solid; mp: 121–122 °C. ^1^H NMR (500 MHz, CD_3_OD): *δ* 7.93 (d, *J* = 1.7 Hz, 2H), 7.84 (s, 1H), 3.44 (s, 4H), 3.11 (ddd, *J* = 8.5, 6.2, 2.1 Hz, 2H), 3.05 (ddd, *J* = 9.7, 6.0, 2.0 Hz, 2H). ^13^C NMR (126 MHz, CD_3_OD): *δ* 142.03, 131.4 (q, *J*_CF_ = 32.8 Hz), 129.19, 123.5 (q, *J*_CF_ = 272.2 Hz), 120.01 (p, *J*_CF_ = 3.8 Hz), 56.82, 55.84, 31.13. HRMS (ESI-TOF) m/z: calculated for C_15_H_13_F_6_NO_2_ [M - H]^−^ 352.0778, found 352.0797.

#### 4.1.22. General protocol for the synthesis of esters 38 and 39

To a suspension of (CH_3_)_3_COK and KI in dry DMF (8 mL) was added 3,5-bis(trifluoromethyl)phenol. The resulting mixture was then warmed to 85 °C and stirred for 90 min. The corresponding ethyl bromo ester was added to the mixture, and the reaction was stirred at 85 °C for an additional 10 h. After cooling to room temperature, H_2_O (20 mL) was added, and the resulting mixture was extracted with ethyl acetate (20 mL, 3×). The organic layers were combined and washed with 1 M NaOH (20 mL) and brine (20 mL). The organic layer was dried (Na_2_SO_4_) and evaporated to afford the crude compound, which was purified by silica gel flash column chromatography using a gradient elution of 0 % to 30 % diethyl ether in heptane to afford the final compounds as oils.

##### 4.1.22.1. Ethyl 2-(3,5-bis(trifluoromethyl)phenoxy)-2-methylpropanoate (38).

Following the general protocol for the synthesis of esters **38** and **39** and using (CH_3_)_3_COK (0.980 g, 8.69 mmol), KI (0.360 g, 2.17 mmol), 3,5-bis(trifluoromethyl)phenol (1.0 g, 0.65 mL, 4.34 mmol) and **36** (1.69 g, 1.28 mL, 8.69 mmol). The ester compound **38** (0.73 g, 2.13 mmol, 49 %) was obtained as a pale green oil. ^1^H NMR (500.1 MHz, CDCl_3_): *δ* 7.48 (s, 1H), 7.24 (s, 2H), 4.24 (q, *J* = 7.1 Hz, 2H), 1.65 (s, 6H), 1.24 (t, *J* = 7.1 Hz, 3H). ^13^C NMR (125.8 MHz, CDCl_3_): *δ* 173.03, 156.31, 132.56 (q, *J*_CF_ = 33.4 Hz), 123.04 (q, *J*_CF_ = 272.7 Hz), 118.69 (d, *J*_CF_ = 4.1 Hz), 115.29 (p, *J*_CF_ = 3.9 Hz), 80.30, 61.95, 25.27, 13.95.

##### 4.1.22.2. Ethyl 1-(3,5-bis(trifluoromethyl)phenoxy)cyclobutane-1-carboxylate (39).

Following the general protocol for the synthesis of esters **38** and **39** and using (CH_3_)_3_COK (0.980 g, 8.69 mmol), KI (0.360 g, 2.17 mmol), 3,5-bis(trifluoromethyl)phenol (1.0 g, 0.65 mL, 4.34 mmol) and **37** (1.80 g, 1.45 mL, 8.69 mmol). The ester compound **39** (0.603 g, 1.69 mmol, 39 %) was obtained as a pale-yellow oil. ^1^H NMR (500.1 MHz, CDCl_3_): *δ* 7.38 (s, 1H), 7.01 (s, 2H), 4.16 (q, *J* = 7.1 Hz, 2H), 2.77–2.68 (m, 2H), 2.44–2.37 (m, 2H), 2.08–1.91 (m, 2H), 1.11 (t, *J* = 7.1 Hz, 3H). ^13^C NMR (125.8 MHz, CDCl_3_): *δ* 171.73, 156.36, 132.77 (q, *J*_CF_ = 33.5 Hz), 123.03 (q, *J*_CF_ = 272.7 Hz), 115.91 (d, *J*_CF_ = 4.1 Hz), 114.66 (p, *J*_CF_ = 3.9 Hz), 80.36, 61.83, 32.18, 13.93, 13.71.

#### 4.1.23. (E)-5-(3,5-Bis(trifluoromethyl)phenoxy)-5-methylhex-3-en-2-one (40)

To a dried three-neck round-bottom flask backfilled with N_2_ was added **38** (0.98 g, 2.90 mmol) and dry DCM (20 mL). After cooling the resulting solution to −78 °C, a solution of DIBAL-H in toluene (1 M, 2.90 mmol, 2.90 mL) was added dropwise, and the resulting mixture was stirred for 1 h. A saturated solution of NH_4_Cl was added, and the reaction mixture was left to warm to room temperature. An additional volume of DCM (20 mL) was added, and the organic and aqueous layers were separated. The organic layer was then washed with brine, dried over anhydrous Na_2_SO_4_, and evaporated to afford the intermediate aldehyde compound (quantitative), which was immediately used in the following reaction. To a solution of the intermediate aldehyde solubilized in THF (20 mL) was added 1-(triphenylphosphoranylidene)propan-2-one (1.11 g, 3.48 mmol), and the solution was stirred for 17 h at room temperature. The solvent was evaporated under reduced pressure to obtain the crude compound, which was purified by silica gel flash column chromatography using a gradient elution of 0 % to 30 % ethyl acetate in heptane to afford the final compound **40** (0.533 g, 1.57 mmol, 54 %) as a pale green oil. ^1^H NMR (500.1 MHz, CDCl_3_): *δ* 7.43 (s, 1H), 7.24 (s, 2H), 6.85 (d, *J* = 16.3 Hz, 1H), 6.15 (d, *J* = 16.3 Hz, 1H), 2.21 (s, 3H), 1.48 (s, 6H). ^13^C NMR (125.8 MHz, CDCl_3_): *δ* 197.68, 156.26, 149.44, 132.57 (q, *J*_CF_ = 33.4 Hz), 129.26, 123.00 (q, *J*_CF_ = 272.7 Hz), 120.69 (d, *J*_CF_ = 3.9 Hz), 116.03–115.80 (m), 80.2, 27.7, 26.9.

#### 4.1.24. (E)-4-(1-(3,5-bis(trifluoromethyl)phenoxy)cyclobutyl)but-3-en-2-one (41)

To a dried three-neck round-bottom flask backfilled with N_2_ was added **39** (1.02 g, 2.86 mmol) and dry DCM (20 mL). After cooling the resulting solution to −78 °C, a solution of DIBAL-H in toluene (1 M, 2.86 mmol, 2.90 mL) was added dropwise, and the resulting mixture was stirred for 1 h. A saturated solution of NH_4_Cl was added, and the reaction was left to warm to room temperature. An additional volume of DCM (20 mL) was added, and the organic and aqueous layers were separated. The organic layer was washed with brine (20 mL), dried over anhydrous Na_2_SO_4_, and evaporated to afford the intermediate aldehyde compound (quantitative), which was immediately used in the following reaction. To a solution of the intermediate aldehyde dissolved in THF was added 1-(triphenylphosphoranylidene)propan-2-one (1.09 g, 3.42 mmol), and the solution was stirred for 17 h at room temperature. The solvent was evaporated under reduced pressure to obtain the crude compound, which was purified by silica gel flash column chromatography using a gradient elution of 0 % to 30 % ethyl acetate in heptane to afford the final compound **41** (0.594 g, 1.69 mmol, 59 %) as a pale-yellow oil. ^1^H NMR (500.1 MHz, CDCl_3_): *δ* 7.37 (s, 1H), 7.02 (s, 2H), 6.97 (d, *J* = 16.1 Hz, 1H), 6.17 (d, *J* = 16.1 Hz, 1H), 2.54–2.36 (m, 4H), 2.19 (s, 3H), 2.02–1.79 (m, 2H). ^13^C NMR (125.8 MHz, CDCl_3_): *δ* 197.50, 155.92, 145.56, 132.74 (q, *J*_CF_ = 33.4 Hz), 128.58, 123.02 (q, *J*_CF_ = 272.7 Hz), 116.78 (d, *J*_CF_ = 3.1 Hz), 116.1–111.1 (m), 80.88, 34.09, 28.07, 12.87.

#### 4.1.25. General protocol for the synthesis of sterically hindered cyclohexane-1,3-diones 20 and 21

To a solution of the respective enone intermediate (1 equiv) and diethyl malonate (1 equiv) in absolute ethanol (4 mL) was added a solution of NaOEt in ethanol (2 equiv). The resulting mixture was then stirred for 16 h at room temperature. An aqueous solution of NaOH (3 equiv) was added to the solution, and the reaction was left stirring at room temperature for 5 h. A solution of 2 N HCl was added to acidify the reaction media to a pH of around 1, and the resulting mixture was heated to 100 °C and stirred for 2 h. An excess of water (25 mL) was added to stop the reaction, and the resulting mixture was extracted with ethyl acetate (3 × 20 mL). The organic layers were combined, washed with brine (20 mL), and dried over anhydrous Na_2_SO_4_. The organic solvent was evaporated to afford the crude compound, which was purified by silica gel flash column chromatography using a gradient elution of 0 % to 10 % MeOH in DCM to afford the compound. The product was dissolved in the minimum amount of DCM and crystallized overnight after the slow addition of hexane, to obtain the final compounds.

##### 4.1.25.1. 5-(2-(3,5-bis(trifluoromethyl)phenoxy)propan-2-yl)cyclohexane-1,3-dione (20).

Following the general synthetic protocol for the synthesis of the sterically hindered cyclohexane-1,3-diones and using **40** (0.465 g, 1.37 mmol), diethyl malonate (0.219 g, 0.210 mL, 1.37 mmol) in absolute ethanol (4 mL) and a solution of NaOEt in ethanol (21 wt%, 0.186 g, 2.73 mmol, 1.02 mL). The final compound **20** (0.252 g, 0.67 mmol, 49 %) was obtained as a white solid; m.p. 152.8–153.4 °C. ^1^H NMR (500 MHz, DMSO-*d*_6_): *δ* 11.16 (s, 1H), 7.80 (s, 1H), 7.67 (s, 2H), 5.26 (s, 1H), 2.43–2.36 (m, 5H), 1.30 (s, 6H). ^13^C NMR (126 MHz, DMSO-*d*_6_): *δ* 156.66, 131.70 (q, *J*_CF_ = 32.9 Hz), 124.60, 123.52 (q, *J*_CF_ = 272.8 Hz), 117.07, 103.75, 83.86, 43.79, 23.89. HRMS (ESI-TOF) *m*/*z*: calculated for C_17_H_16_F_6_O_3_ [M + H]^+^ 383.1082, found 383.1090.

##### 4.1.25.2. 5-(1-(3,5-bis(trifluoromethyl)phenoxy)cyclobutyl)cyclohexane-1,3-dione (21).

Following the general synthetic protocol for the synthesis of the sterically hindered cyclohexane-1,3-diones and using **41** (0.572 g, 1.62 mmol), diethyl malonate (0.260 g, 0.25 mL, 1.62 mmol) in absolute ethanol (4 mL) and a solution of NaOEt in ethanol (21 wt%, 0.221 g, 3.25 mmol, 1.05 mL). The impure final compound was obtained as a pale yellow solid (0.268 g, 0.68 mmol) and then purified by preparative HPLC equipped with Luna C18 (2) column (Phenomenex, CA, USA), 2500 × 21.2 mm, 5 μm and using a gradient elution of 10 % to 100 % ACN (0.1 % acetic acid) in water (0.1 % acetic acid) over 20 min to afford the final compound **21** (0.150 g, 0.40 mmol, 25 %) as a white solid; m.p. 72.1–72.9 °C. ^1^H NMR (500 MHz, DMSO-*d*_6_): *δ* 11.16 (s, 1H), 7.64 (s, 1H), 7.43 (s, 2H), 5.20 (s, 1H), 2.84–2.63 (m, 1H), 2.64–2.53 (m, 2H), 2.42–2.22 (m, 6H), 1.88–1.64 (m, 1H), 1.68–1.49 (m, 1H). ^13^C NMR (126 MHz, DMSO-*d*_6_): *δ* 156.29, 132.00 (q, *J*_CF_ = 32.9 Hz), 123.49 (q, *J*_CF_ = 273.0 Hz), 119.04, 114.76, 103.84, 85.11, 36.66, 30.00, 12.98. HRMS (ESI-TOF) m/z: calculated for C_18_H_16_F_6_O_3_ [M + H]^+^ 395.1081, found 395.1098.

### 4.2. Evaluation of small molecule protection against induced mut-SOD1 intracellular aggregation in PC12 Tet-Off -SOD1^G85R^ YFP cells

The PC12 Tet-Off-SOD1^G85R^ YFP cells were cultured in DMEM media supplemented with 10 % horse serum, 5 % Tet-approved FBS, 1 % Penstrep, 1× l-glutamine, 200 μg/mL hygromycin, 100 μg/mL G418, with 1 μg/mL Doxycycline and were incubated at 37 °C in humidified air with 5 % CO until 90–95 % confluency. To induce the expression of SOD1^G85R^ YFP, the Doxycycline was removed at least 4 days before the experiment. To remove the Doxycycline, the media was removed, and the cells were washed 3 times with 1xPBS. The cells were then cultured in standard culture media without Doxycycline. To set up the assay, the cells were then seeded into 96-well cell culture plates (Greiner Bio-One Catalog #655090) at a concentration of 50,000 cells/well with a final volume of 100 μL/well and incubated in the CO_2_ incubator at 37 °C for 24 h. The media were removed from the cells and replaced with 100 μL of media containing 0.1 % DMSO or compounds, and incubated in the CO_2_ incubator at 37 °C for 6 h. The media were then replaced with fresh 100 μL of media containing the diluted compounds with 0.7 μM (−)-MG-132, and the plates were incubated for 72 h under culture conditions. After 72 h, the cells were stained with Hoescht 33,342 (55 μg/mL, 10 μL/well) and imaged (l_ex_ = 358, 513 nm) using a Molecular Devices ImageXpress Micro Confocal High-Content Imaging System equipped with a 40× objective. The image was set to 2. Hoescht 33,342 fluorescence Image binning was set to 2. DAPI fluorescence was captured using an exposure time of 300 ms, and FITC fluorescence was captured using an exposure time of 400 ms. A 13-plane z-series was acquired for each field of view with a step size of 3 μm. For each condition, 2 sets of 9 images were generated in a 3 × 3 grid to separately image the SOD1^G85R^ YFP proteins and the nuclei stained with Hoescht 33,342. Image analysis was performed with MetaXpress software (Molecular Devices) using the Transfluor module. Parameters for puncta detection (aggregates) were set with a minimum pit width of 2 μm and a maximum pit width of 5 μm. Signal intensity thresholds were defined as 700 grayscale units above the local background. The analysis provided the average area of each cell that is covered by puncta (pit/cell), which are indicated by bright green spots on the SOD1^G85R^YFP image set.

The assay was performed in triplicate for each condition tested, and wells containing the media with vehicle (0.1 % DMSO) or (−)-MG-132 alone were used as controls representing the minimum and maximum aggregate formation. An initial single-point screen at 10 μM was performed, and the compounds presenting a decrease in aggregate formation >80 % were selected, and their EC_50_ values were obtained from a dose-dependent response assay performed with 12 concentrations between 15 and 0.2 μM. The data for EC_50_ determination was treated with Prism and fitted using a semi-logarithmic curve for determination of the EC_50_ values of **NU9** and compounds **4**, **6**, **20** and **21**.

### 4.3. Parallel artificial membrane permeability assay – blood brain barrier (PAMPA-BBB)

A solution of 1 × PBS (pH 7.5) was initially prepared by the dilution of a commercially available 10 × PBS solution. The initial 10 mM stock solutions of the tested compounds and the controls verapamil (high permeability) and theophylline (low permeability) were prepared in DMSO and then diluted in 1 × PBS to obtain the final solution of the compounds at 250 μM (2 % DMSO *v*/v). A solution of porcine brain lipid (PBL) (20 mg/mL in dodecane) was prepared, and 4 μL of this solution was used to coat the filters on the bottom of the PAMPA donor plates (Sigma Aldrich #MAIPNTR10). After air drying for 20 min, the donor plate wells were charged with 250 μL of the solutions of the test compounds or the controls. The donor plate was then placed over the acceptor plate (Sigma Aldrich #MATRNPS50), in which wells were previously filled with 1 × PBS (2 % DMSO *v*/v) solution. The PAMPA sandwich was closed with parafilm and incubated at 25 °C for 18 h in a humidity-saturated atmosphere with orbital agitation. After incubation, 150 μL of the donor and acceptor wells of each compound was collected and transferred to a new plate with low UV absorption at 240 nm (UV star plates – Greiner #655801). The UV spectra (250–400 nm) of each well were acquired on a BioTek Synergy H1 MD plate reader equipped with a monochromator at room temperature. The passive permeability (P_e_) of the compounds and the controls was determined using the equation:

$$\text{Pe}(\text{cm}/\text{s})=\frac{2.303}{\text{A}\times (t-\tau \text{ss})}\times \frac{\text{VA}\times \text{VD}}{\left(\text{VA}+\text{VD}\right)}\text{Log}[1-\left(\frac{\text{VA}+\text{VD}}{(1-R)\times \text{VD}}\right)\times \left(\frac{\text{CA}(\text{t})}{\text{CD}(0)}\right)]$$

where A is the area of the filter of the donor wells (0.21 cm^2^); t is the incubation time (64,800 s); τss is the time to reach the steady state (usually neglectable because it is very short compared to the total incubation time); V_A_ and V_D_ are the volumes of the acceptor and donor wells respectively (0.25 cm^3^); R is the membrane retention factor and is calculated using the equation:

$$\text{R}=\left[1-\left(\frac{\text{CD}(\text{t})}{\text{CD}(0)}\right)-\left(\frac{\text{VA}}{\text{VD}}\times \frac{\text{CA}(\text{t})}{\text{CD}(0)}\right)\times \left(\frac{\text{CA}(\text{t})}{\text{CD}(0)}\right)\right]$$

${\text{C}}_{\text{A}}(t)$ is the concentration of the compounds at the final time of incubation on the acceptor well; ${\text{C}}_{\text{D}}(0)$ and ${\text{C}}_{\text{D}}(t)$ are the concentration of the compounds at the initial time of incubation and at the final time of incubation on the donor well, respectively. The permeability of the compounds was considered valid when the recovery rate of the compounds was >75 %, and the P_e_ results are presented as an average of at least three independent experiments, each performed in triplicate.

### 4.4. In vitro efflux pump studies

#### 4.4.1. MDCK-MDR1 assay

The assay was carried out at Sai Life Sciences. Stock solutions of **6**, **20**, assay controls loperamide, atenolol, and propanolol (4 and 20 mM), and P-gp inhibitor zosuquidar (10 mM) were prepared in DMSO. Then the stock solutions from each test compound were diluted in HBSS buffer containing 10 mM HEPES to obtain the assay working solutions without the P-gp inhibitor at 2 and 10 μM (0.1 % DMSO v/v). Into working solutions containing zosuquidar (5 μM) were individually diluted **6**, **20**, and loperamide to obtain working solutions containing the test compounds at 2 or 10 μM and zosuquidar at 5 μM.

The MDCK-MDR1 (Madin Darby canine kidney - Multidrug Resistance gene-1) cells were cultured in DMEM media supplemented with 10 % FBS and antibiotics. For transport studies, cells were seeded into PET trans filter membranes (Millipore #PSRP010R5; 1.0 μm pore size) at a density of 1.2 × 10^5^ cells/well and colchicine was added to the flasks and plates according to their volume. After 24 h, the media were replaced by fresh media and then changed every 2 days over 8 days. The media were removed from the wells of the basal and apical compartments of the plates, and a solution of HBSS with or without zosuquidar (5 μM) was added to the apical (400 μL) and basal (800 μL) sides of the plates. The plates were pre-incubated at 37 °C for 30 min in humidified air with 5 % CO_2_. The TTER (Transepithelial-Transendothelial Electrical Resistance) value for each well was recorded (assay TEER >83 Ω.cm^2^). The buffer was removed from both compartments, and for the apical to basal direction transport determination, the apical compartment was loaded with 400 μL of the working solutions of the tested compounds or controls (2 or 10 μM) in the presence or absence of zosuquidar (5 μM), and the basal compartment was loaded with 800 μL of HBSS (0.1 % DMSO v/v). For the basal to apical direction transport determination, the compartments were loaded in their reverse order. The assay was performed in duplicate for the two transport directions [apical to basolateral (A → B) and basolateral to apical (B → A)]. After 120 min, 100 μL samples from both the apical and basal sides of the plate were transferred to new 96-deep well plates, quenched with acetonitrile containing an internal standard, vortexed for 5 min, and then centrifuged at 4000 rpm for 10 min. The same procedure was used to prepare a 96-deep well plate containing non-assayed working solutions (*t* = 0 concentrations). After centrifugation, 100 μL of the supernatant of each well was transferred to a new 96-well plate, and the samples were analyzed by LC-MS/MS.

#### 4.4.2. MDCKII-BCRP assay

The assay was carried out at Sai Life Sciences with MDCKII-BCRP (Madin Darby Canine Kidney, MDR1 knock out and transfected with Breast Cancer Resistance Protein) cells following the same protocol described for the assay with MDCK-MDR1 cells, with minor modifications, namely, the use of the BCRP inhibitor Ko-143 instead of the P-gp inhibitor zosuquidar.

For each experiment, the apparent permeability (P_app_) for each direction and the efflux ratio (ER) were determined by the equations:

$$\text{Papp}(\text{cm}/\text{s})=\frac{\left(\text{dQ}/\text{dt}\right)}{\text{A}\times \text{Co}}$$

where dQ/dt is the rate of permeation of the tested compounds across the (0 and 120 min) after correcting for dilution; A is the area of the filter (0.7 cm^2^); Co is the initial concentration of the compounds on the donor compartment.


$$\text{ER}=\frac{\text{P}(\text{B}\to \text{A})}{\text{P}(\text{A}\to \text{B})}$$

Where P(B→A) is the apparent permeability of the compounds from the basal to the apical direction and P(A→B) is the apparent permeability from the apical to basal direction. Lucifer Yellow was used as a control of the assay to evaluate monolayer integrity after incubation with the working solutions of the compounds, and the results were validated if the permeability of Lucifer Yellow from the apical to the basal side after 1-h incubation was kept <2 %. The results are presented as an average of two independent experiments.

### 4.5. Microsomal metabolic stability studies

The assay was performed at Jubilant Biosys. Vials containing human or mouse microsomes were collected from the −80 °C freezer and thawed on ice. Then 27.5 μL of the initial microsome solutions (20 mg/mL) were diluted in 971.5 μL of a 67 mM potassium phosphate buffer solution (pH 7.4). The testing compounds were diluted in the buffered microsomal solution to obtain a final concentration of 1.1 μM. Then four different aliquots, each containing 180 μL of the assay solution, representing the samples at *T* = 0, *T* = 5, *T* = 15, and *T* = 30 min. The samples were pre-incubated at 37 °C for 5 min with gentle shaking, and then 20 μL of a NADPH solution (10 mM) was added to the different solutions of the test compounds. The reactions were stopped by the addition of 200 μL acetonitrile containing an internal standard (Loperamide, Tolbutamide or Warfarine) at the specified times of incubation, and then the samples were centrifuged at 3220 x*g* over 20 min. An aliquot of the supernatant (200 μL) was collected from each reaction sample and analyzed by LC-MS/MS. The Ln values of the peak area of the compounds to the internal standard over time (min.) were graphed using Excel, and the elimination rates of the compounds (*k*) were determined from the slopes obtained from the graphs. The *k* values were used to calculate the compounds half-life using the equation:

$$\text{t}1/2=\frac{\text{Ln}\;2}{k}$$

The intrinsic clearance (CL_int_) of each compound in the presence of mouse and human microsomes was calculated using the equation:

$$\text{CLint}=\frac{0.693}{\text{t}1/2}\times \frac{\text{V}}{\text{microsomal protein (mg)}}$$

Where ${\text{t}}_{1/2}$ is the compound half-life determined on each technical replicate; V is the total volume of microsomal incubation solution for each compound (1000 μL); microsomal protein is the total concentration of microsomal protein used in the assay (0.5 mg/mL).

Verapamil and Ketoconazole were used as controls to evaluate the assay performance. The results are presented as an average of two technical replicates.

### 4.6. In vitro cytotoxicity profile studies

The HepG2 and HEK293 cells were cultured in DMEM media supplemented with 10 % FBS (heat-inactivated), 1 % Pen-strep, and l-glutamine and incubated at 37 °C in humidified air with 5 % CO_2_ until 85–90 % confluency. The PC12 Tet-Off-SOD1^G85R^ YFP cells were maintained as described in 4.2. On the day before the experiments, the cells were transferred to white bottom 96 well plates (Greiner cat. 655,083) at a concentration of 50,000 cells/100 μL of media in each well and incubated overnight at 37 °C. Stock solutions of the compounds (30 mM) in DMSO were prepared and then diluted in media to obtain the six final working solutions starting at 30 μM, and serial dilutions were obtained with the lowest concentration at 0.25 μM. The media were removed and fresh media containing the compounds or the vehicle DMSO (0.1 % *v*/v) were transferred to the plates containing HepG2 and HEK293 cells and the plates were incubated for 24 or 48 h at 37 °C. The PC12 Tet-Off-SOD1^G85R^ YFP cells compound treatment was performed as described in section 4.2 and using a 9-point 2-fold serial dilution of the compounds starting at 15 μM and the lowest concentration at 0.05 μM. After incubation, the plates were cooled to room temperature and 25 μL of CellTiter-Glo^®^ solution was added to each well. The plates were loaded into a BioTek Synergy H1 MD plate reader and shaken for 2 min, stabilized for 10 min without shaking, and then the luminescence values were read for each well (luminescence integration step 1 ms). Wells containing media with the vehicle DMSO with or without cells were used as controls to represent, respectively, the maximum viability and the lowest viability (background signal) for the cytotoxicity studies with HepG2 and HEK293 cell lines. The viability values for the assay performed with the PC12 Tet-Off-SOD1^G85R^ YFP cells were normalised for the vehicle (0.1 % DMSO) and (−)-MG-132 alone treatments, which represented respectively the minimum (0 %) and maximum (100 %) viability for the assay. The EC_50_ values for **NU9** and compounds **6** and **20** on the viability screening with PC12 Tet-Off-SOD1^G85R^ YFP cells were determined with Prism by fitting the data for the dose-dependent response to a non-linear variable slope (four-parameter) curve.

The results are presented as an average of three independent experiments, each performed in triplicate, for the cytotoxicity experiments performed with HepG2 and HEK293 cells. The results for the protective screening with the PC12 Tet-Off-SOD1^G85R^ YFP cells are presented as an average of four replicates.

### 4.7. High-resolution microscopy studies with DAPRed autophagy dye in neuronal HT22 hippocampus cells

HT22 immortalized mouse hippocampal neuronal cell line was maintained in DMEM supplemented with 10% FBS and 1% penicillin-streptomycin under standard conditions (37 °C, 5% CO_2_). On the day before the experiment, HT22 cells were transferred to a sterile poly-d-lysine-coated Nunc^™^ Lab-Tek^™^ II Chambered Coverglass (8 wells; Thermo Fisher Sci. cat. 155,409) at a concentration of 40,000 cells/300 μL of media in each well and incubated overnight at 37 °C. Stock solutions of Torin-1 (10 mM) and **20** (10 mM) were prepared in DMSO and then diluted in media to obtain the final treatment solutions at 0.2 and 3 μM, respectively. The media was removed, the cells were quickly washed with 1×PBS solution, and fresh media containing the compounds or the DMSO (0.1 % v/v) were transferred to the wells. The plates were incubated for 2 h. The media was removed, and after washing once with 1×PBS, fresh media containing Bafilomycin (0.1 μM), DAPRed dye (0.25 μM) and Hoescht 33,342 (55 μg/mL) was added to the wells and the cells were incubated for 3 h at 37 °C. Bafilomycin blocked autolysosome formation and allowed the increase of mature autophagosome formation, enhancing DAPRed intensity. The media was removed, the cells were washed once with 1×PBS, and fresh warm media was added before the microscopy data was acquired. The cells were imaged with super-resolution microscopy (Nikon-SORA) at 60×. The images were processed in ImageJ by propagating equal intensity of the DAPRed channel. Puncta structures >0.2 μm were then identified using Otsu thresholding and the results represented as particles per cell for each set of images analyzed. Statistical analyses and graph representation were performed using Prism 10 software (Graphpad Software). The results are presented as an average of *n* = 4 independent sets of images ± sd for each condition. The D’Agostino and Pearson normality test was performed for all datasets. The statistical differences between two or more groups were determined by One-way ANOVA followed by Sidak post-hoc multiple-comparison test. Statistically significant differences were taken at *p* < 0.04.

## Supplementary Material

SI

## Figures and Tables

**Fig. 6.**
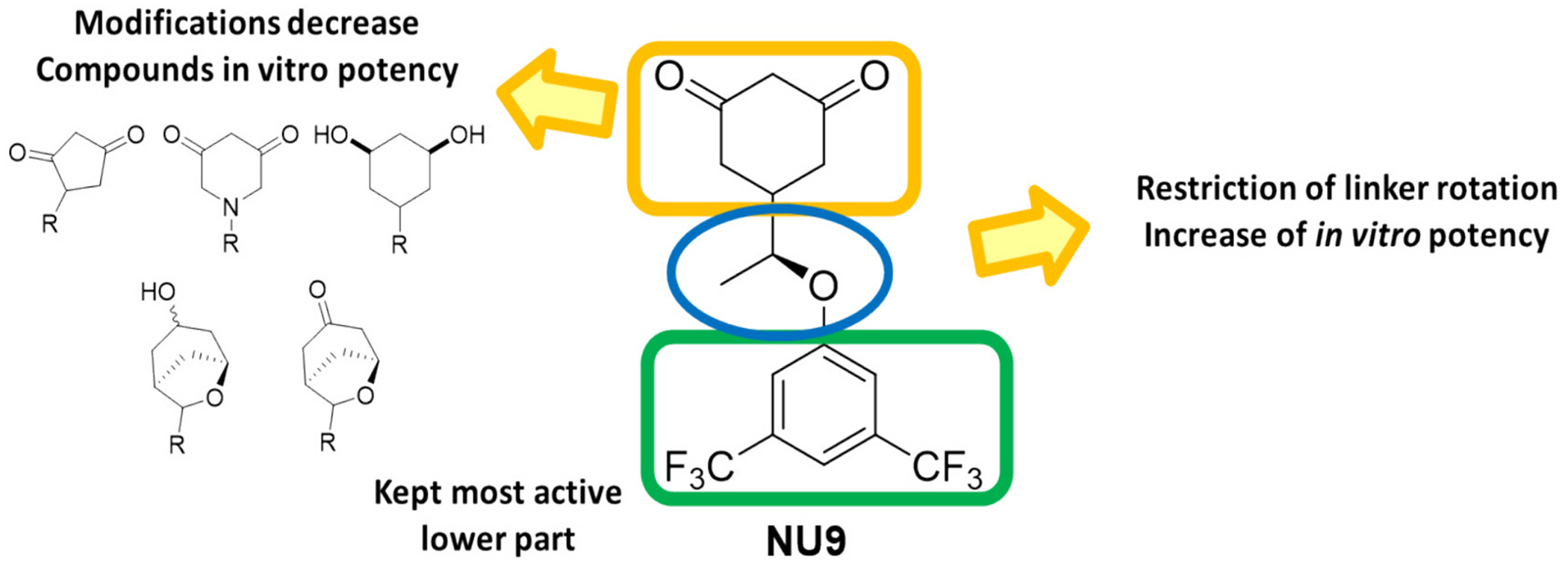
Summary of the *in vitro* SAR of the CHD compounds anti-aggregation activity in PC12-Tet-Off SOD1^G85R^ YFP cells.

**Table 1:** EC_50_ values (**NU-9** and compounds **4–21)** against MG132-induced mutant SOD1 intracellular aggregation in PC12-SOD1^G85R^ YFP cells and calculated lipophilicity (cLogP)^a^ of **NU-9** and compounds **4, 6, 20, 21.**

Compound	EC_50_ (μM)^a^	cLogP^c^
NU-9	2.52 ± 0.38	4.39
4	7.89 ± 1.88	3.90
5	Inactive	–
6	2.06 ± 0.24	4.58
7	Inactive	–
8	>10	–
9	ND^b^	–
10	>10	–
11	Inactive	–
12	Inactive	–
13	Inactive	–
14	Inactive	–
15	Inactive	–
16	>10	–
17	Inactive	–
18	Inactive	–
19	Inactive	–
20	2.63 ± 0.27	4.61
21	5.37 ± 1.03	4.77

a EC_50_ values were obtained for the compounds that, similarly to **NU-9,** decreased the MG132-induced SOD-1^G85R^ aggregate formation in >80 % at 10 μM; Inactive compounds did not decrease MG132-induced SOD-1^G85R^ aggregates formation at 10 μM when compared with the vehicle treatment [DMSO 0.1 % (*v*/v)].

b EC_50_ of compound **9** was not defined (ND); at 10 μM the compound presented a decrease in MG132-induced SOD-1^G85R^ aggregate formation of 61.30 %, which was below the threshold of 80 %.

c Calculated using SwissADME software (http://www.swissadme.ch/index.php). cLogP values were calculated for the more abundant enol form of the compounds.

**Table 2:** PAMPA-BBB passive permeability (P_e_) for compounds **4, 6, 20, 21**, and **NU-9.** Controls include verapamil (high permeability), theophylline (low permeability), and the ALS-approved drugs riluzole and edaravone. Calculated lipophilicity (cLog P), topological polar surface area (TPSA), molecular weight (MW), number of hydrogen bond donor groups (NHBD), and number of rotatable bonds (NRB) of the evaluated compounds are given.

Compound	P_e_ (10^−6^ cm/s)	Log P_e_^a^	cLog P^b^	TPSA (Ǻ^2^)^b^	MW (g/Mol)^b^	NHBD^b^	NRB^b^
NU-9	6.98 ± 0.14	−5.16	4.39	46.53	368.27	1	5
4	0.25 ± 0.01	−6.61	3.90	57.53	368.27	2	5
6	8.46 ± 0.10	−5.07	4.58	37.30	350.26	1	4
20	11.54 ± 0.66	−4.94	4.61	46.53	382.30	1	5
21	16.83 ± 0.54	−4.77	4.77	46.53	394.31	1	5
Edaravone	17.43 ± 3.20	−4.76	1.64	32.67	174.20	0	1
Riluzole	33.68 ± 0.96	−4.47	2.89	67.15	234.20	1	1
Verapamil	25.41 ± 1.87	−4.60	–	–	–	–	
Theophylline	0.22 ± 0.02	−6.66	–	–	–	–	

a Prediction of BBB passive permeability scale. No permeability LogP_e_ < −6.14; Low permeability −6.14 < LogP_e_ < −5.66; Medium permeability −5.66 < LogPe<−5.33; High permeability LogPe > −5.33.

b Values were calculated using the Swiss ADME website (http://www.swissadme.ch/).

**Table 3:** Bidirectional permeability and efflux ratio of compounds **6, 20,** P-gp substrate loperamide (Lop.), and BCRP substrate dantrolene (Dan.) in MDCK-MRD1 and MDCKII-BCRP cells in the presence (+) or absence (−) of P-gp inhibitor zosuquidar (Zosuq.) or BCRP inhibitor Ko143.

Treatment	MDCK-MRD1			MDCKII-BCRP		
	P_app_ (A → B)^a^ (10^−6^ cm/s)	P_app_ (B → A)^a^ (10^−6^ cm/s)	Efflux Ratio^b^	P_app_ (A → B)^a^ (10^−6^ cm/s)	P_app_ (B → A) ^a^ (10^−6^ cm/s)	Efflux Ratio^b^
6 − Zosuq	10.60 ± 1.28	24.31 ± 0.18	2.29	–	–	–
6 + Zosuq	13.55 ± 2.30	14.14 ± 1.66	1.04	–	–	–
6 − Ko143	–	–	–	0.66 ± 0.18	31.49 ± 2.73	47.38
6 + Ko143	–	–	–	20.43 ± 2.62	18.44 ± 1.15	0.90
20 − Zosuq	5.82 ± 0.06	14.73 ± 0.78	2.53	–	–	–
20 + Zosuq	11.72 ± 0.39	9.40 ± 0.86	0.80	–	–	–
20 − Ko143	–	–	–	3.62 ± 0.22	19.23 ± 0.88	5.32
20 + Ko143	–	–	–	10.68 ± 0.72	8.95 ± 0.46	0.84
Lop. − Zosuq	0.20 ± 0.06	25.70 ± 0.35	127.85	–	–	–
Lop. + Zosuq	9.01 ± 0.49	5.32 ± 0.81	0.59	–	–	–
Dan. − Ko143	–	–	–	0.52 ± 0.10	29.72 ± 3.21	56.85
Dan. + Ko143	–	–	–	24.97 ± 0.16	16.47 ± 1.28	0.66

a $P_{\text{app}}(A\to B)$ apparent permeability apical to basal direction; $P_{\text{app}}(B\to A)$ apparent permeability basal to apical direction.

b Efflux ratio < 2.0 - Not a substrate for efflux transport pathway. Efflux ratio > 2.0 - Substrate for efflux transport pathway.

**Table 4:** *In vitro* human and mouse microsomal stability of compounds **6** and **20**.

Compound	CL_int_ (human) (μL/min/mg)	t_1/2_ (human) (min)^a^	CL_int_ (mouse) (μL/min/mg)	t_1/2_ (mouse) (min)^b^
NU-9	–	74^27^	–	52^27^
6	25.8 ± 1.6	53.9 ± 3.3	67.3 ± 0.9	20.7 ± 0.3
20	10.4 ± 3.1	>120	35.7 ± 4.7	40.7 ± 3.8

a Human microsomal stability classification: High stability t_1/2_ > 30 min.; intermediate stability 30 min > t_1/2_ > 10 min; Low stability <10 min.

b Mouse microsomal stability classification: High stability t_1/2_ > 60 min.; low stability <30 min [50,51].

**Table 5:** *In vitro* cytotoxicity profile of **NU-9, 6,** and **20** on HepG2 and HEK293 cell lines.

Compound	HepG2 EC_50_ (μM)	HEK293 EC_50_ (μM)	PC12-SOD1^G85R^ EC_50_ (μM)	EC_50_ Fold difference
NU-9 (24 h)	>30 (93.54 ± 1.44)^a^	>30 (85.73 ± 0.79)^a^	2.52 ± 0.38^b^	>12
NU-9 (48 h)	>30 (96.27 ± 1.84)^a^	>30 (89.62 ± 3.55)^a^	3.37 ± 0.26^c^	
6 (24 h)	>30 (91.91 ± 3.28)^a^	>30 (82.59 ± 1.23)^a^	2.06 ± 0.24^b^	>14
6 (48 h)	>30 (94.05 ± 4.37)^a^	>30 (89.59 ± 2.76)^a^	1.98 ± 0.25^c^	
20 (24 h)	>30 (96.04 ± 3.39)^a^	>30 (90.04 ± 1.72)^a^	2.63 ± 0.27^b^	>11
20 (48 h)	>30 (92.30 ± 1.24)^a^	>30 (91.61 ± 0.83)^a^	2.47 ± 0.15^c^	

a Percentage of viable cells observed at 30 μM treatment for both HepG2 and HEK293 cells; viability expressed relative to untreated cells.

b EC_50_ from the *in vitro* anti-aggregation screening with PC12-SOD1^G85R^ YFP cells reported in Table 1.

c EC_50_ from the *in vitro* viability screening with PC12-SOD1^G85R^ YFP cells co-treated with MG132 and the compounds.

## Data Availability

Data will be made available on request.

## Footnotes

2 Cyclohexane-1,3-dione has been shown in many cases to favor the tautomeric form, 3-hydroxycyclohex-2-en-1-one, but it is depicted and written as cyclohexane-1,3-dione throughout.

## Appendix A. Supplementary data

Supplementary data to this article can be found online at https://doi.org/10.1016/j.bioorg.2025.109190.

## Abbreviations:

ABC: ATP binding cassette efflux transporters
ALS: Amyotrophic lateral sclerosis
ATP: Adenosine triphosphate
BBB: Blood-brain barrier
BCRP: Breast cancer resistance protein
CHD: Cyclohexane-1,3-dione
CL_int_: Microsomal intrinsic clearance
CNS: Central nervous system
DCM: Dichloromethane
DIBAL-H: Diisobutylaluminium hydride
DMAP: 4-Dimethylaminopyridine
DMF: Dimethylformamide
DMSO: Dimethyl sulfoxide
EC_50_: Half maximal effective concentration
Et_3_N: Triethyl amine
Et_2_O: Diethyl ether
EtOH: Ethanol
EtONa: Sodium ethoxide
ER: Endothelium reticulum
HCl: Hydrochloric acid
H_2_O: Water
HTPS: High-throughput phenotypic screen
K_2_CO_3_: Potassium carbonate
LDA: Lithium diisopropylamide
LiI: Lithium iodide
MDCKII-BCR: Madin Darby canine kidney
MDR1: Knock out and breast Cancer resistance protein knock-in cells
MDCK-MDR1: Madin Darby canine kidney multidrug resistance gene-1 cells
MeOH: Methanol
MW: Molecular weight
NaBH_4_: Sodium borohydride
NHBD: Number of hydrogen bond donors
NaOAc: Sodium acetate
NaOH: Sodium hydroxide
NRB: Number of rotatable bonds
P_app_: Apparent permeability
PAMPA: Parallel artificial membrane permeability assay
PCC: Pyridinium chlorochromate
P_e_: Passive permeability
P-gp/MDR1: P-glycoprotein/ multidrug resistance protein 1
PK-PD: Pharmacokinetic-pharmacodynamic profile
Rh/Al_2_O_3_: Rhodium on alumina catalyst
SAR: Structure activity relationship studies
SOD1: Superoxide dismutase 1
t_1/2_: Half-life
TBS: Tert-butyl(dimethyl)silyl protecting group
TDP-43: TAR DNA-binding protein 43
THF: Tetrahydrofuran
Ti(O-iPr)_4_: Titanium Isopropoxide
TPSA: Topological polar surface area
UPS: Ubiquitin-proteasome
YFP: Yellow fluorescent protein
