# Supplementary material for: Modifications of NU-9, a potent protein aggregation inhibitor. Properties and activity in a cellular model of amyotrophic lateral sclerosis
Source: Bioorg Chem. Author manuscript; Available in PMC 2025 Nov 27. (PMC12648443; doi:10.1016/j.bioorg.2025.109190)
Supplement: SI [file NIHMS2123515-supplement-SI.pdf]

Supporting Information

Modifications of NU-9, a potent protein aggregation inhibitor. Properties and activity in a cellular model of amyotrophic lateral sclerosis

Mohamed F. Elmansy,[†,⊥,1] Pedro Soares,[†,§,1] Jose Ricardo D. dos Remedios,[†,1] Raghad Nowar,[γ] Susan G. Fox,[§#] Anan Yu,[§,#] William L Klein,[†,γ,Δ] Richard I. Morimoto,[§,#] Richard B. Silverman*[†,§,∥]

[†] *Department of Chemistry, Chemistry of Life Processes Institute, and Center for Developmental Therapeutics, Northwestern University, Evanston, Illinois 60208, U.S.A.*

[⊥] *Organometallic and Organometalloid Chemistry Department, National Research Centre, 12622 Cairo, Egypt*

[§] *Department of Molecular Biosciences, Northwestern University, Evanston, Illinois, 60208, U.S.A.*

[γ] *Department of Neurobiology, Northwestern University, Evanston, Illinois 60208, U.S.A.*

# Daniel F. and Ada L. Rice Institute for Biomedical Research, Northwestern University, Evanston, Illinois 60208, U.S.A.

[Δ] *Department of Neurology, Northwestern University, Chicago, Illinois 60611, U.S.A.*

[∥] *Department of Pharmacology, Feinberg School of Medicine, Northwestern University, Chicago, Illinois 60611, U.S.A.*

[1] These authors contributed equally to this work.

*Corresponding author: Tel: 847-491-5663. Fax: 847-491-7713. *r-silverman@northwestern.edu*

Table of Contents

1. ^{1}H and ^{13}C NMR spectra……………………………………………………………S3-S64

2. HRMS spectra of compounds **4-10** and **18-20**……………………………………S65-S84

3. HPLC chromatograms of **NU-9** and compounds **4-21**……………………………S85-S94

4. Supplemental Figures 1 and 2: Screening results of the anti-aggregation assay of **NU-9** and compounds **4, 6, 8, 9, 10, 16, 20** and **21**……………………………S95-S96
5. Supplemental Figures 3-7: Cellular cytotoxicity data of **NU-9** and compounds **6** and **20** on HEK298 and HepG2 and PC12 Tet-Off-SOD1^{G85R} YFP cell lines…S97-S101

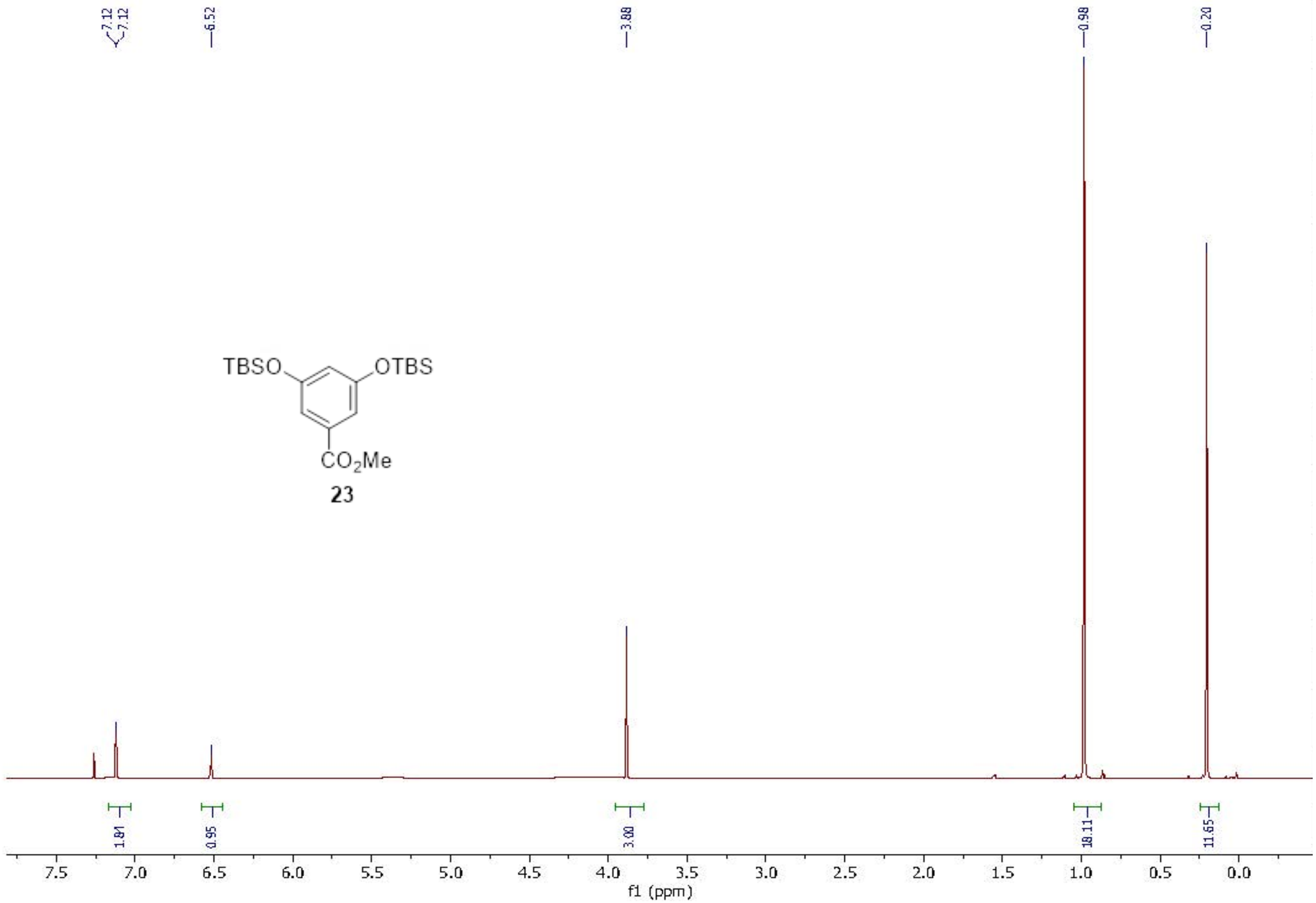
7.12
7.12
6.52
3.88
0.98
0.20
TBSO
OTBS
CO2Me
23
1.81
0.95
3.00
18.11
11.65
7.5
7.0
6.5
6.0
5.5
5.0
4.5
4.0
3.5
3.0
2.5
2.0
1.5
1.0
0.5
0.0
f1 (ppm)

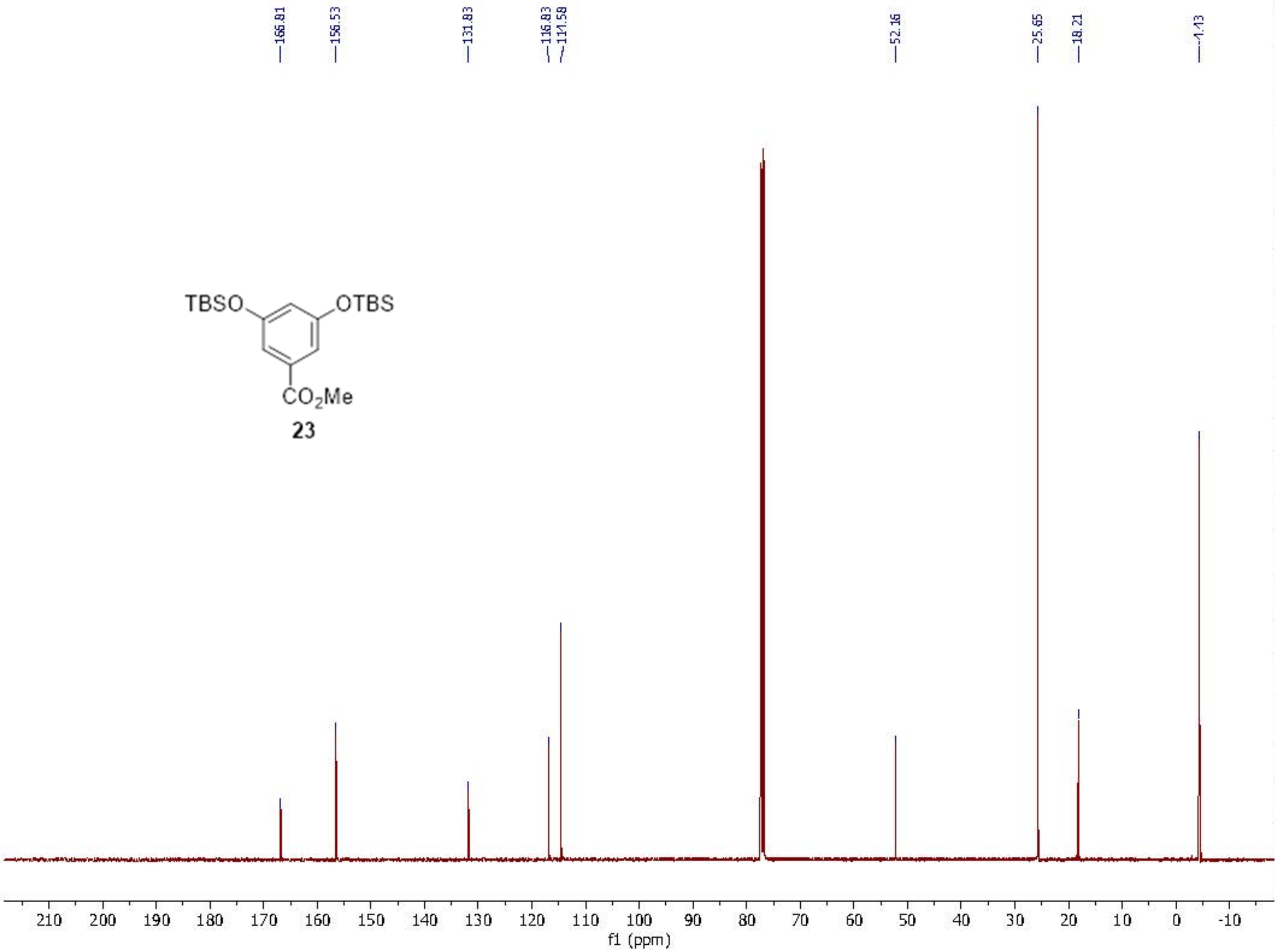
166.81
156.53
131.83
116.83
114.58
52.16
25.65
18.21
TBSO
OTBS
CO_2Me
210 200 190 180 170 160 150 140 130 120 110 100 90 80 70 60 50 40 30 20 10 0 -10
f1 (ppm)

23

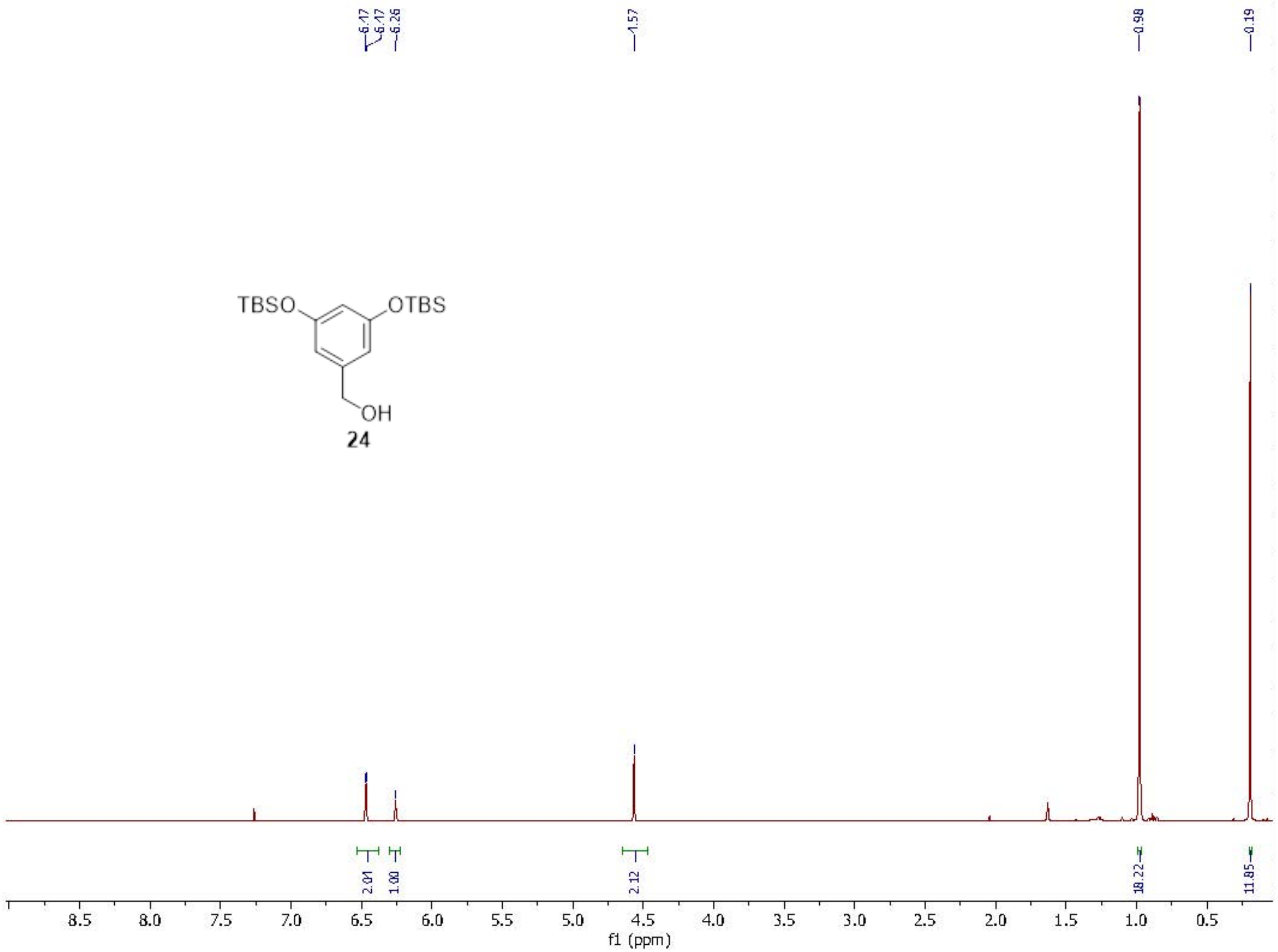
6.47
6.47
6.26
4.57
0.98
0.19
TBSO
OTBS
OH
24
2.01
1.00
2.12
18.22
11.85
8.5
8.0
7.5
7.0
6.5
6.0
5.5
5.0
4.5
4.0
3.5
3.0
2.5
2.0
1.5
1.0
0.5
f1 (ppm)

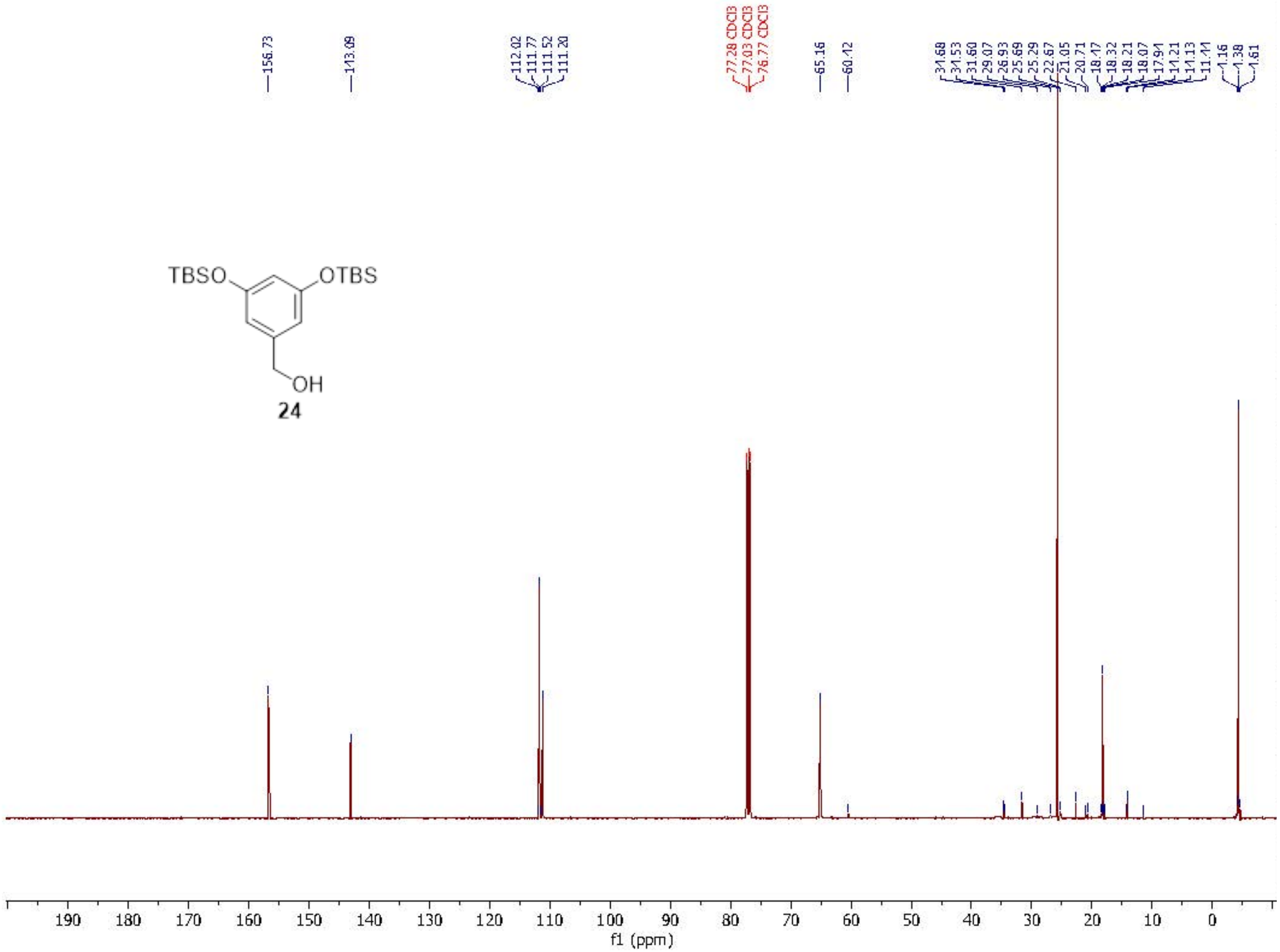
156.73
143.09
112.02
111.77
111.52
111.20
77.28 CDCl3
77.03 CDCl3
76.77 CDCl3
65.16
60.42
34.68
34.53
31.60
29.07
26.93
25.69
25.29
22.67
21.05
20.71
18.47
18.32
18.21
18.07
17.94
14.21
14.13
11.44
-4.16
-4.38
-4.61
f1 (ppm)

24

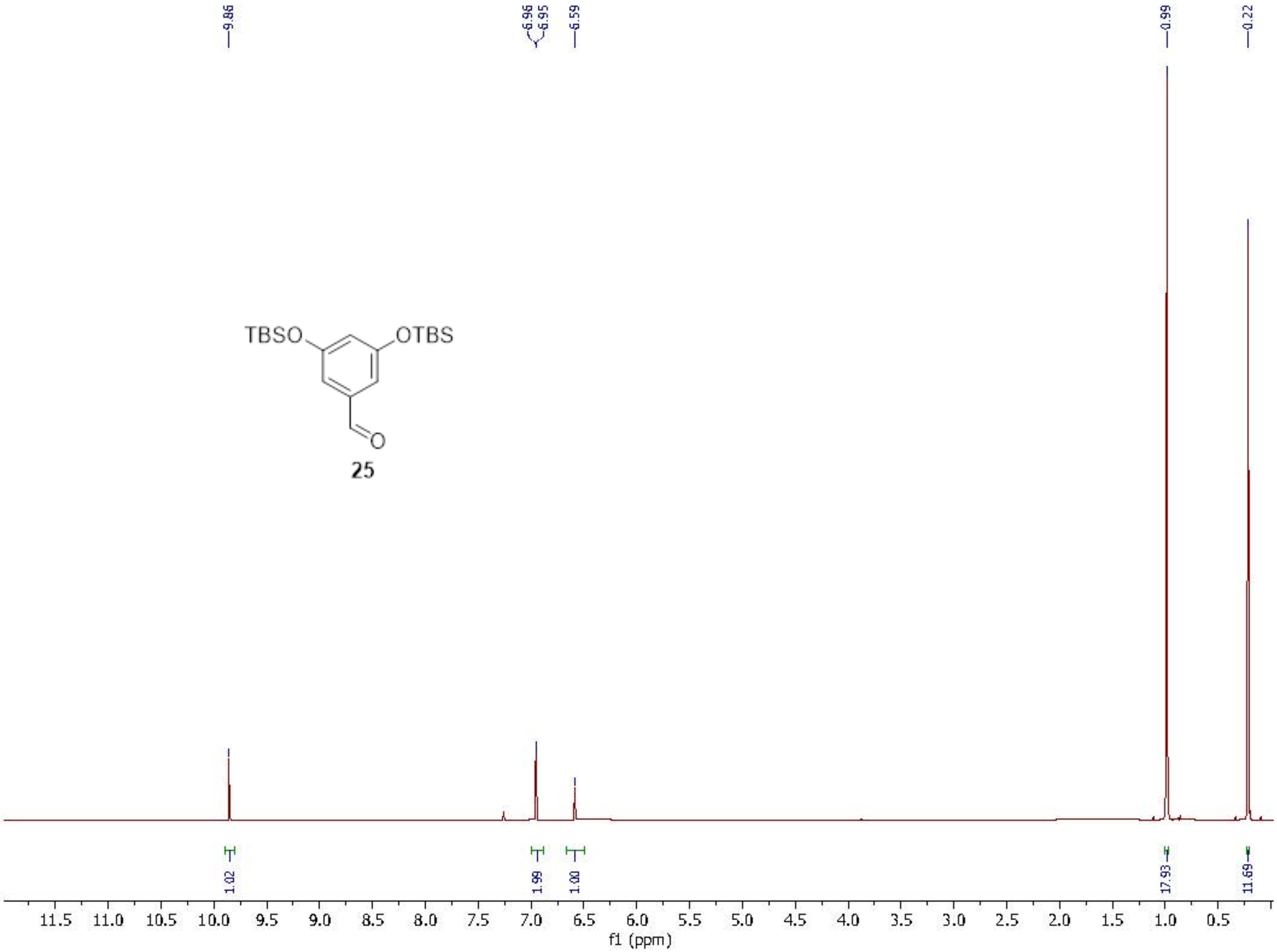
9.86
6.96
6.95
6.59
0.99
0.22
TBSO
OTBS
O
1.02
1.99
1.00
17.93
11.69
11.5
11.0
10.5
10.0
9.5
9.0
8.5
8.0
7.5
7.0
6.5
6.0
5.5
5.0
4.5
4.0
3.5
3.0
2.5
2.0
1.5
1.0
0.5
f1 (ppm)

25

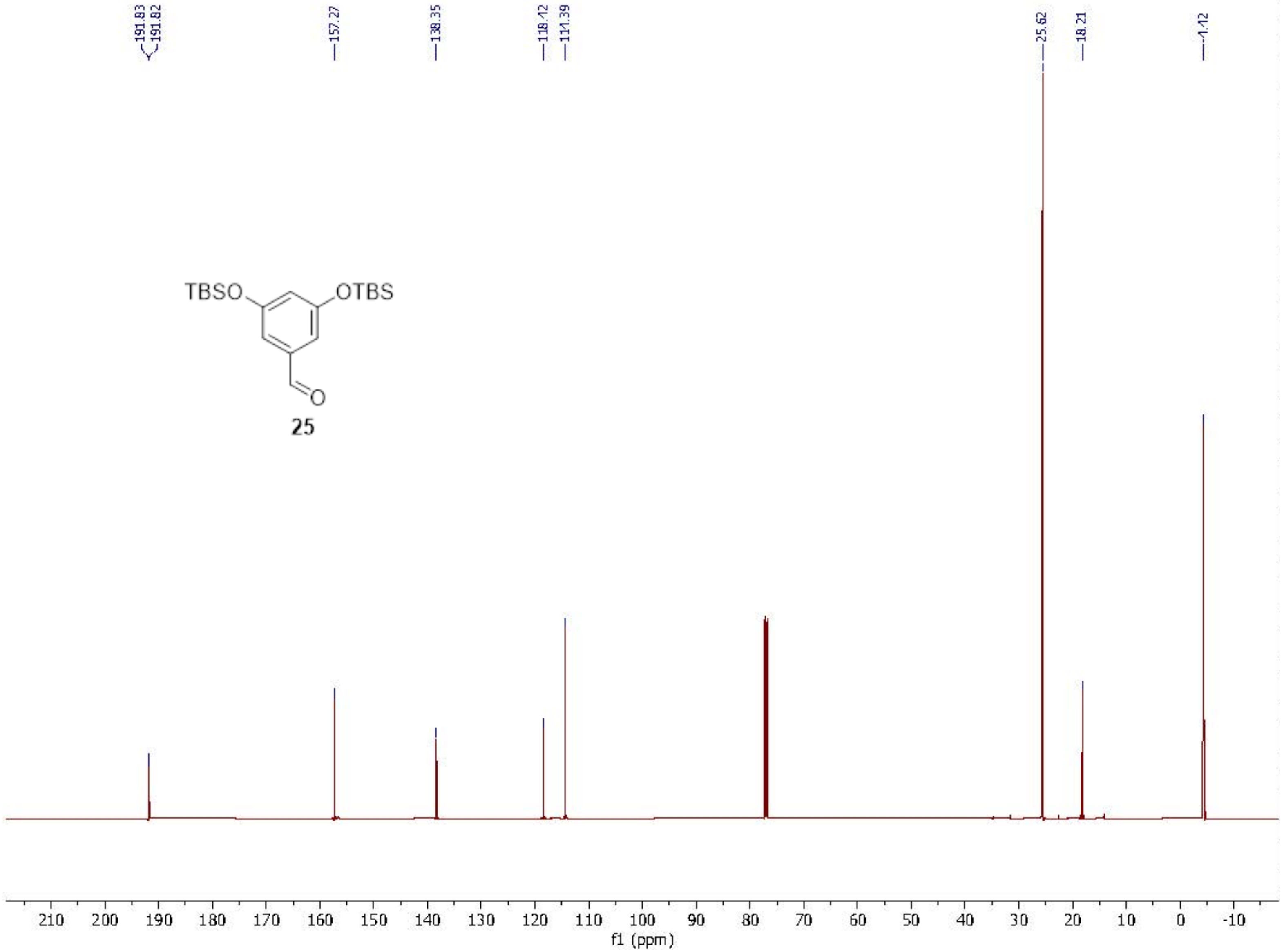
191.83
191.82
157.27
138.35
118.12
114.39
25.62
18.21
-4.12
TBSO
OTBS
O
25
210 200 190 180 170 160 150 140 130 120 110 100 90 80 70 60 50 40 30 20 10 0 -10
f1 (ppm)

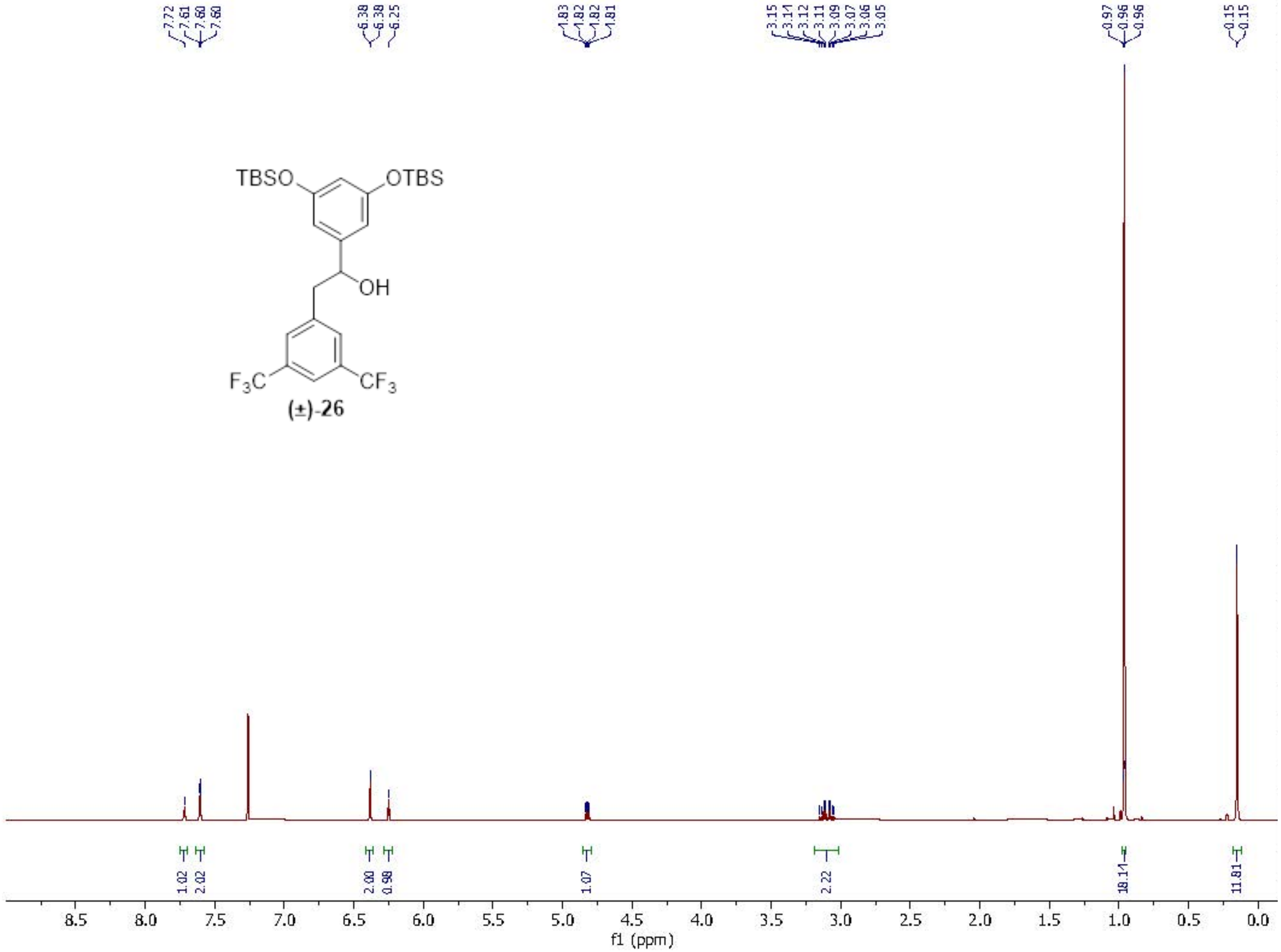
7.72
7.61
7.60
7.60
6.38
6.38
6.25
4.83
4.82
4.82
4.81
3.15
3.14
3.12
3.11
3.09
3.07
3.06
3.05
0.97
0.96
0.96
0.15
0.15
TBSO
OTBS
OH
F3C
CF3
(±)-26
1.02
2.02
2.00
0.98
1.07
2.22
18.14
11.81
8.5
8.0
7.5
7.0
6.5
6.0
5.5
5.0
4.5
4.0
3.5
3.0
2.5
2.0
1.5
1.0
0.5
0.0
f1 (ppm)

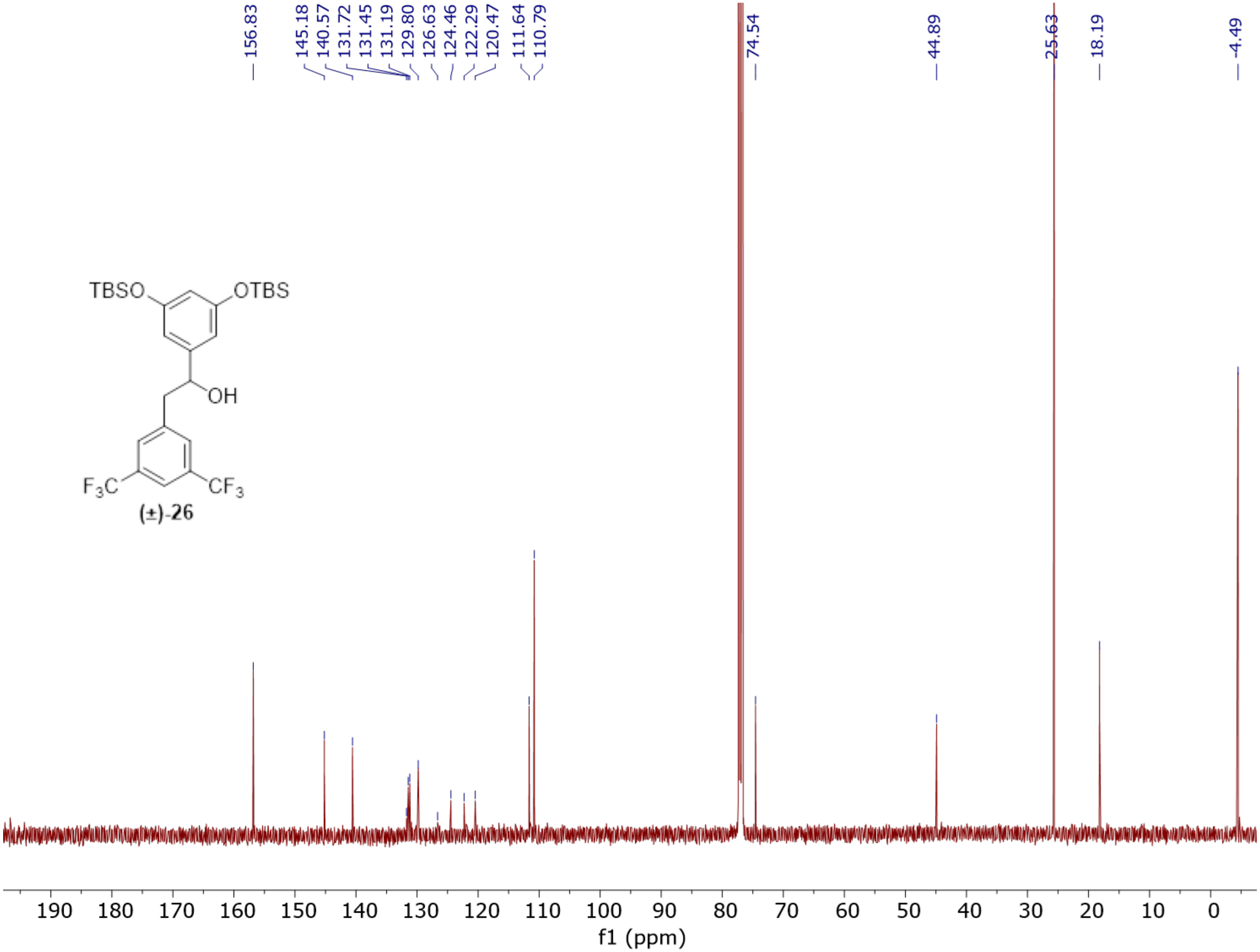
156.83
145.18
140.57
131.72
131.45
131.19
129.80
126.63
124.46
122.29
120.47
111.64
110.79
74.54
44.89
25.63
18.19
-4.49
TBSO
OTBS
OH
F3C
CF3
(±)-26
190 180 170 160 150 140 130 120 110 100 90 80 70 60 50 40 30 20 10 0
f1 (ppm)

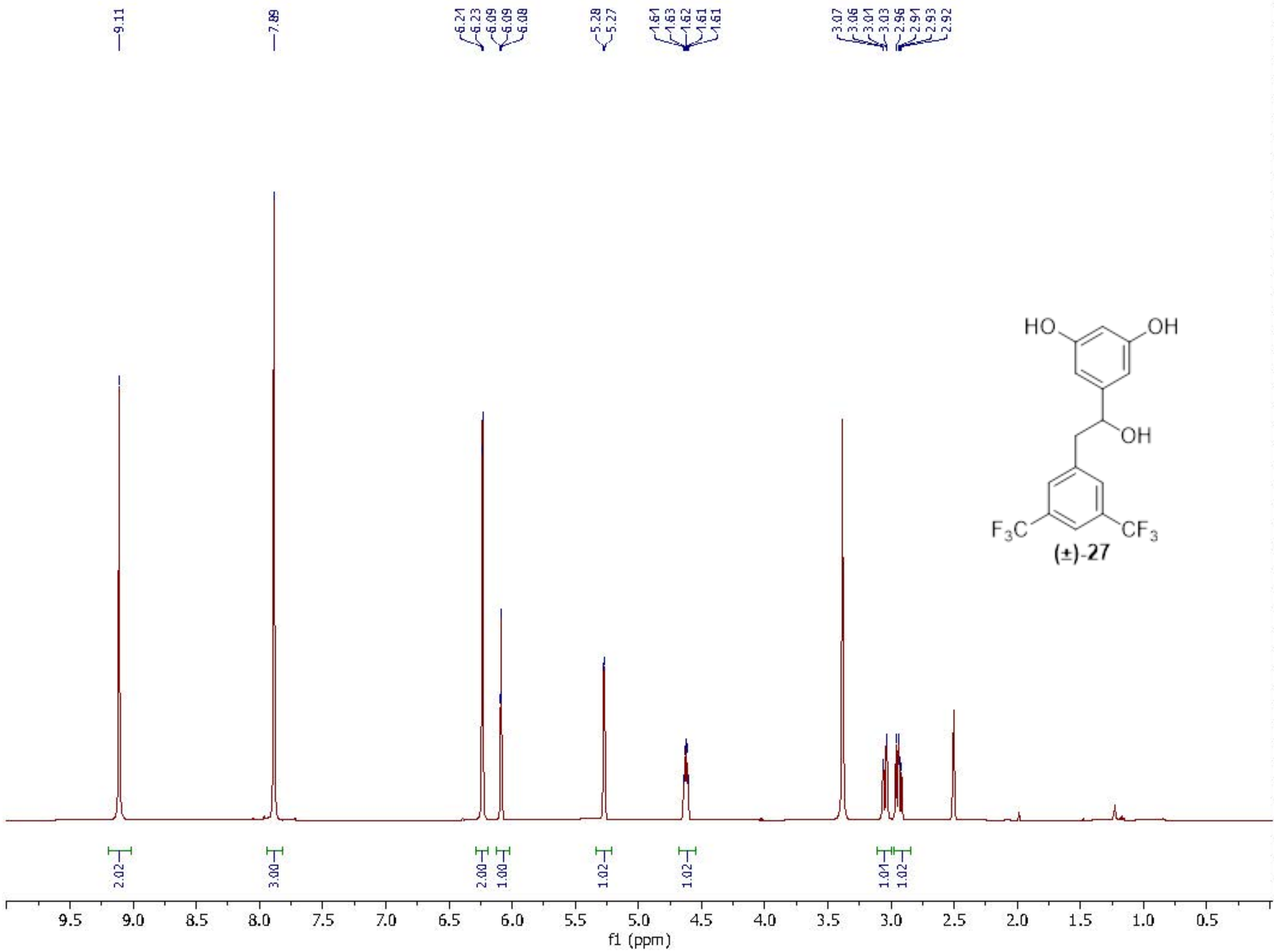
9.11
7.89
6.24
6.23
6.09
6.09
6.08
5.28
5.27
4.64
4.63
4.62
4.61
4.61
3.07
3.06
3.04
3.03
2.96
2.94
2.93
2.92
HO
OH
OH
F_3C
CF_3
(±)-27
2.02
3.00
2.00
1.00
1.02
1.02
1.04
1.02
9.5
9.0
8.5
8.0
7.5
7.0
6.5
6.0
5.5
5.0
4.5
4.0
3.5
3.0
2.5
2.0
1.5
1.0
0.5
f1 (ppm)

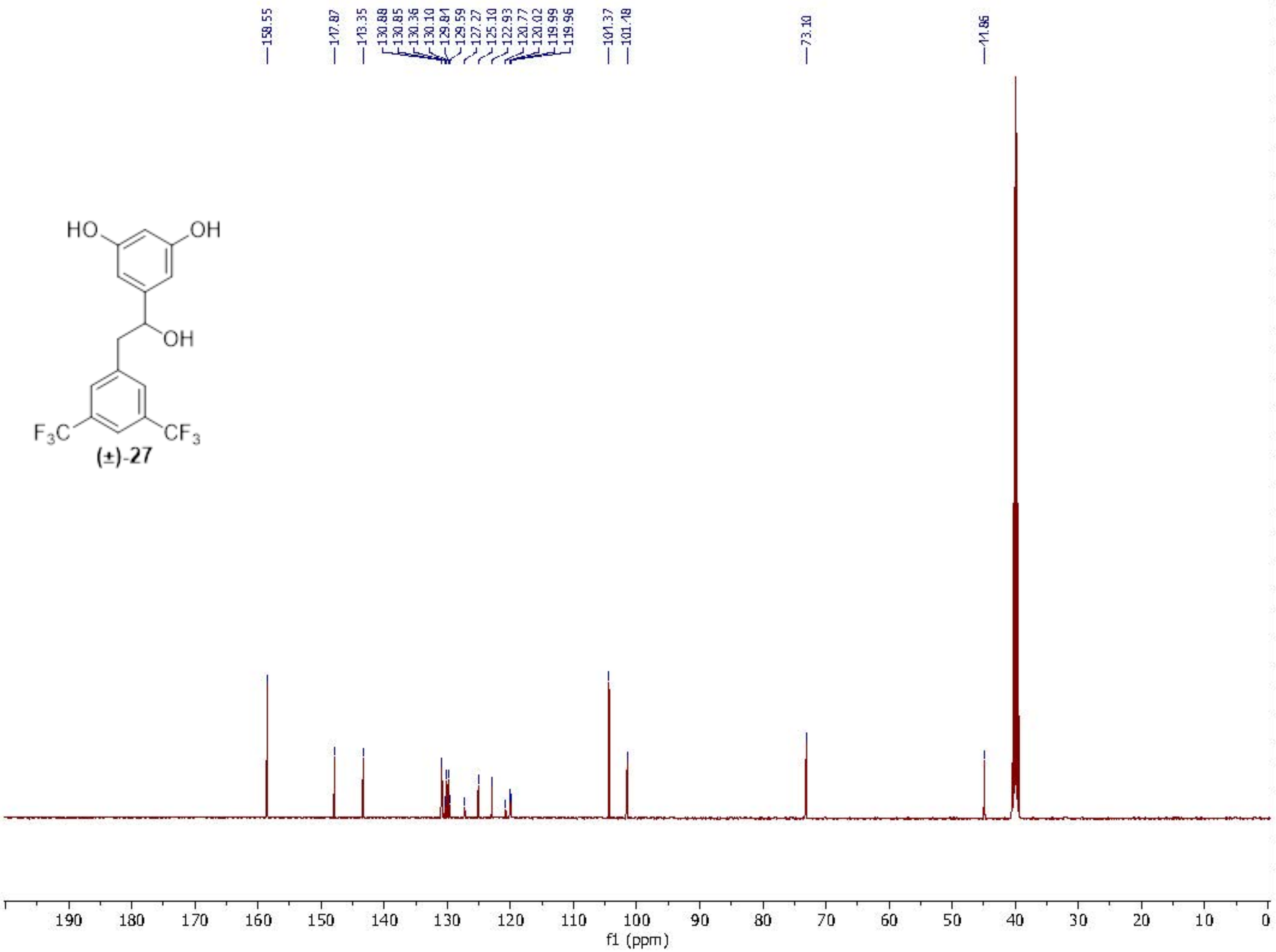

130.85
130.36
130.10
129.59
127.27
125.10
122.93
120.77
120.02
119.99
119.96
73.10
190 180 170 160 150 140 130 120 110 100 90 80 70 60 50 40 30 20 10 0
f1 (ppm)

(±)-27

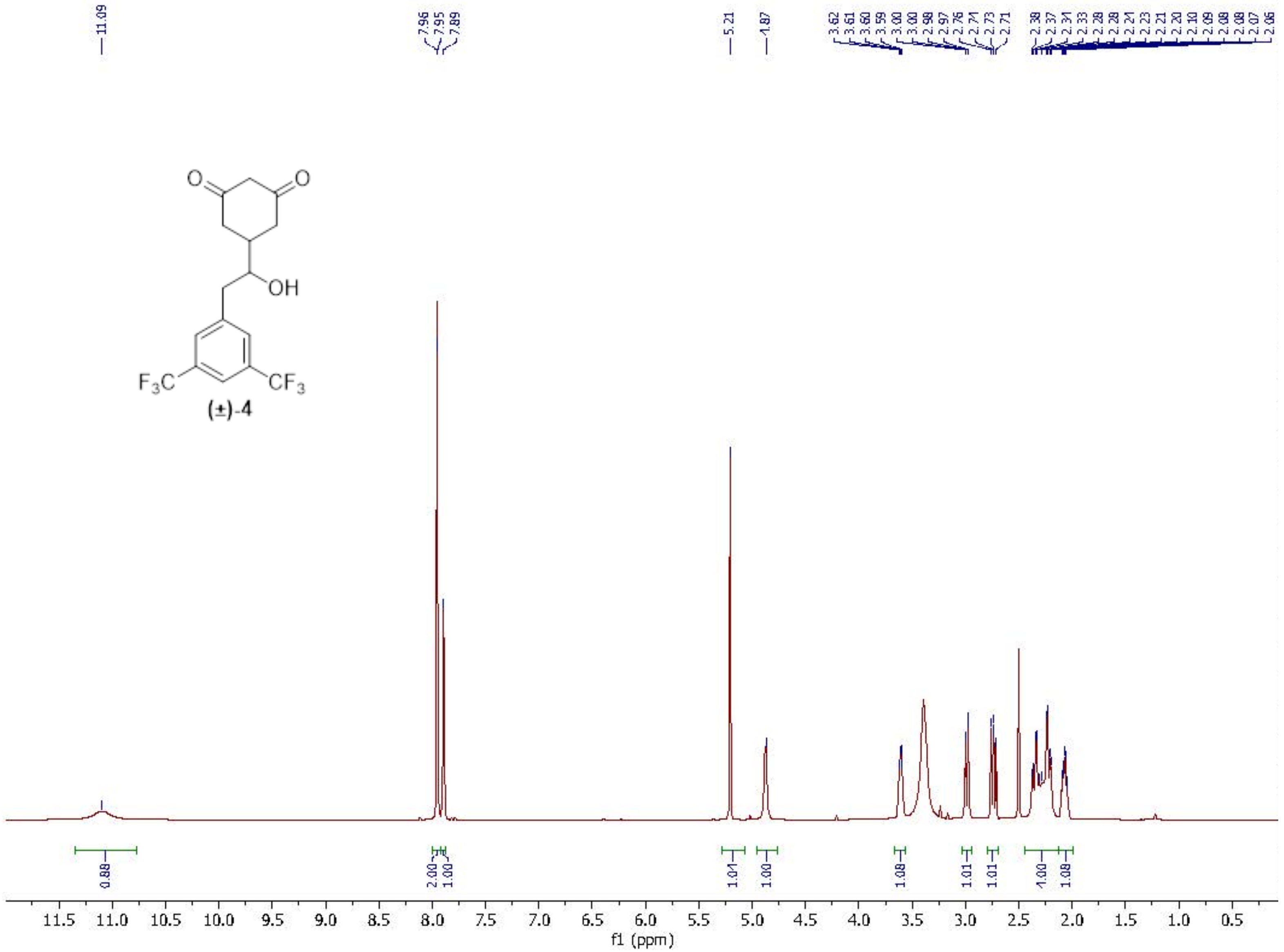

(±)-4
f1 (ppm)

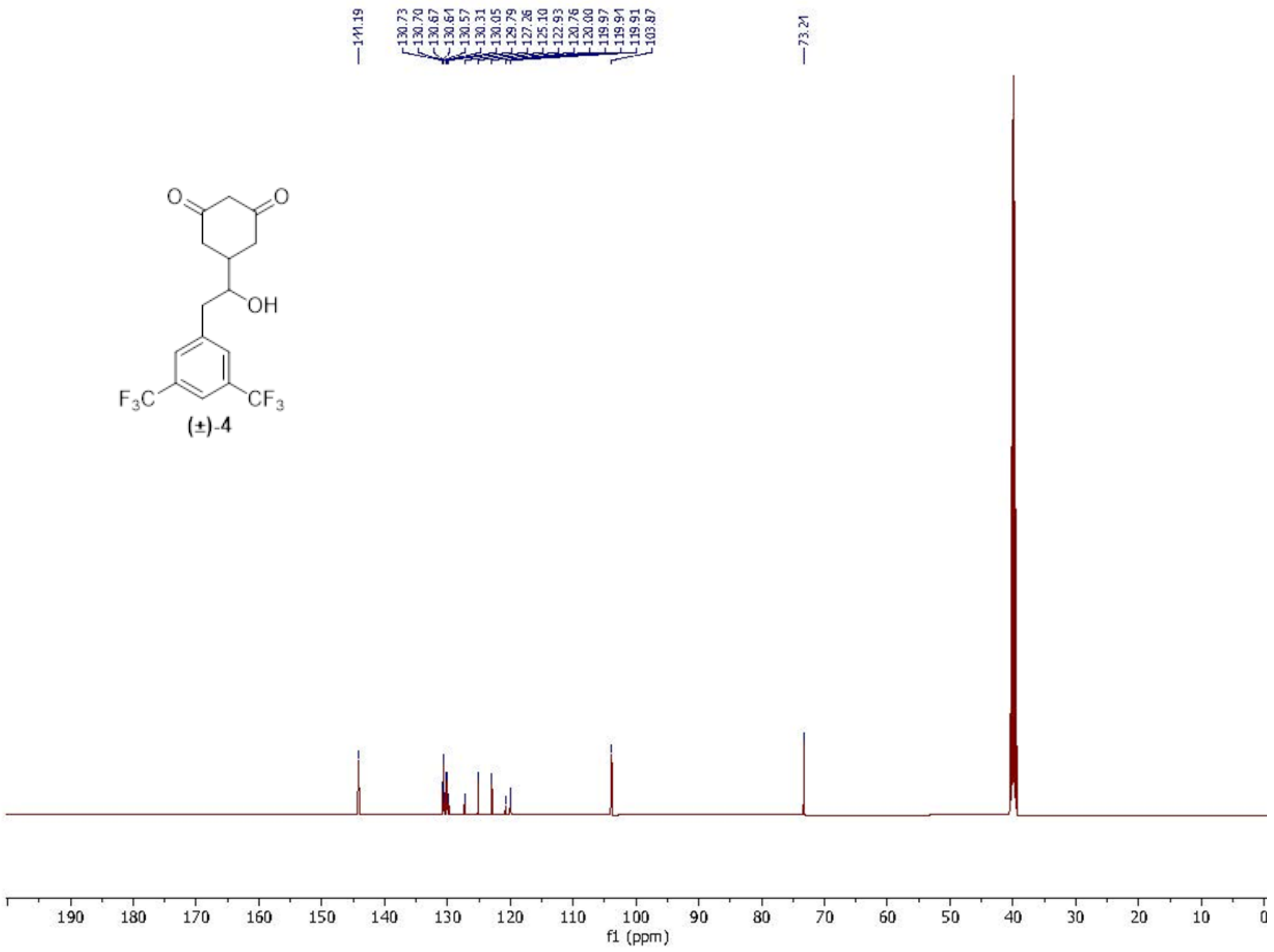

144.19
130.73
130.70
130.67
130.64
130.57
130.31
130.05
129.79
127.26
125.10
122.93
120.76
120.00
119.97
119.94
119.91
103.87
73.24
OH
F3C
CF3
(±)-4
f1 (ppm)

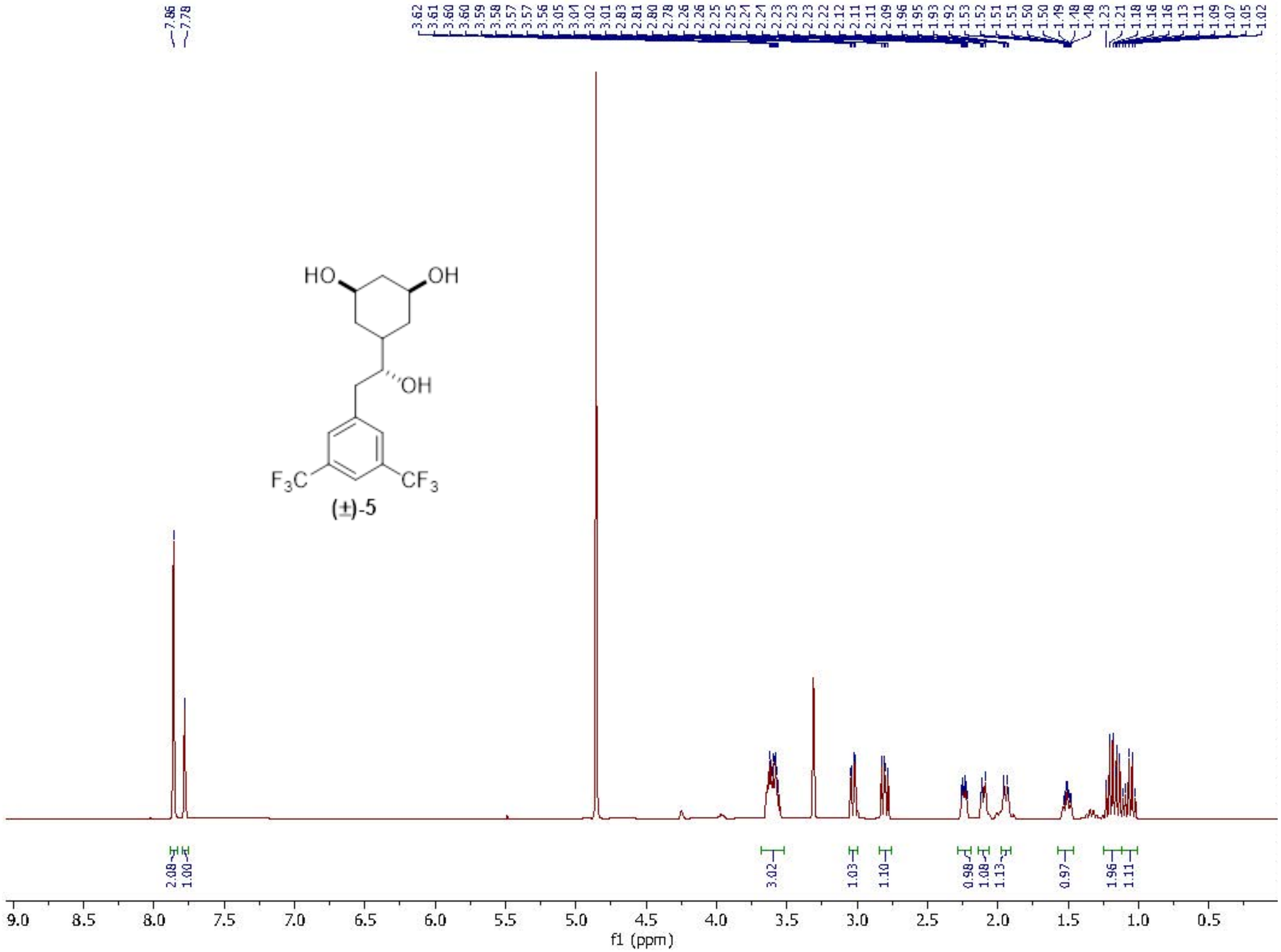
HO
OH
OH
F3C
CF3
(±)-5
f1 (ppm)

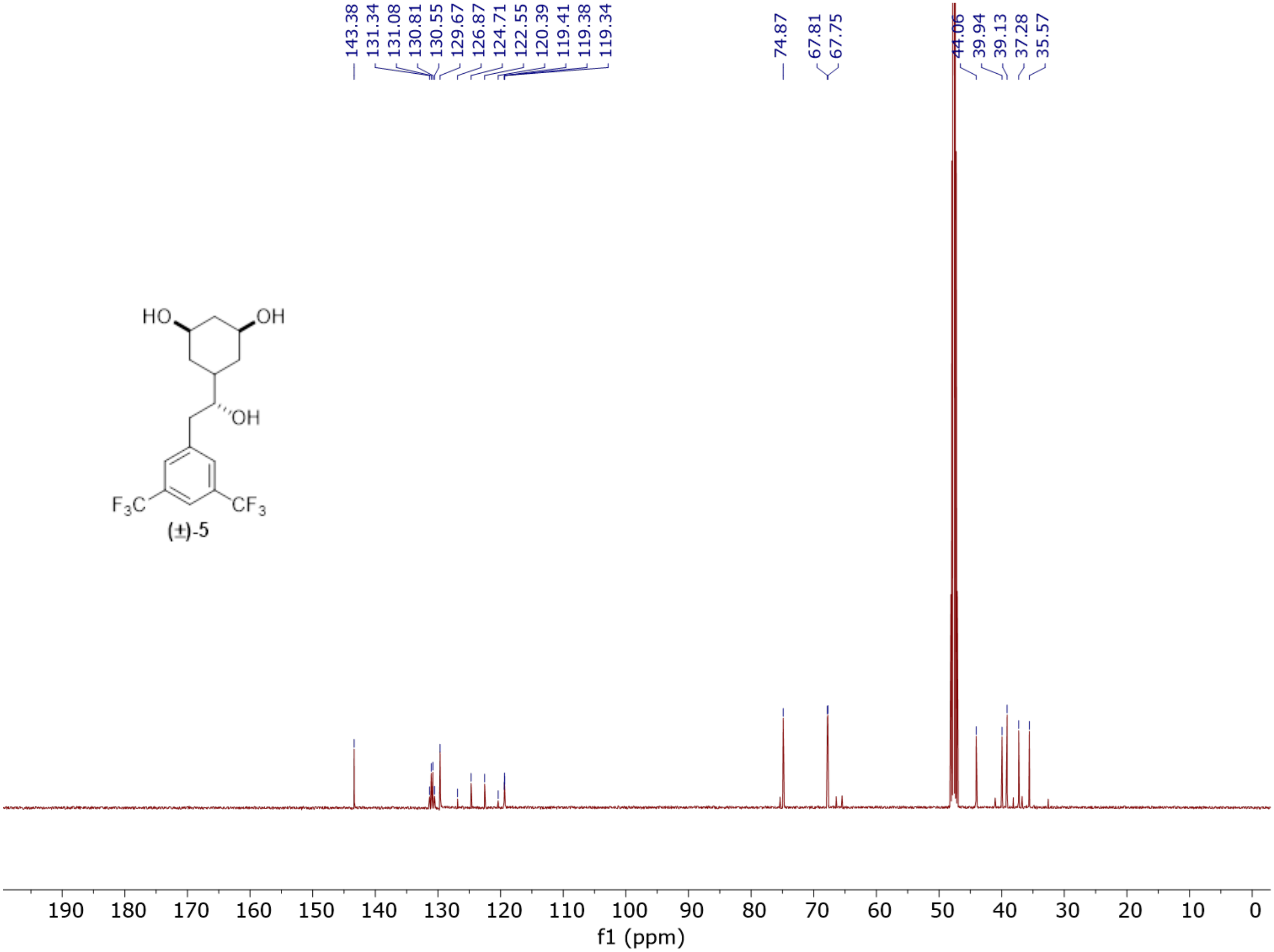

143.38
131.34
131.08
130.81
130.55
129.67
126.87
124.71
122.55
120.39
119.41
119.38
119.34
74.87
67.81
67.75
44.06
39.94
39.13
37.28
35.57
HO
OH
OH
F_3C
CF_3
(±)-5
190 180 170 160 150 140 130 120 110 100 90 80 70 60 50 40 30 20 10 0
f1 (ppm)

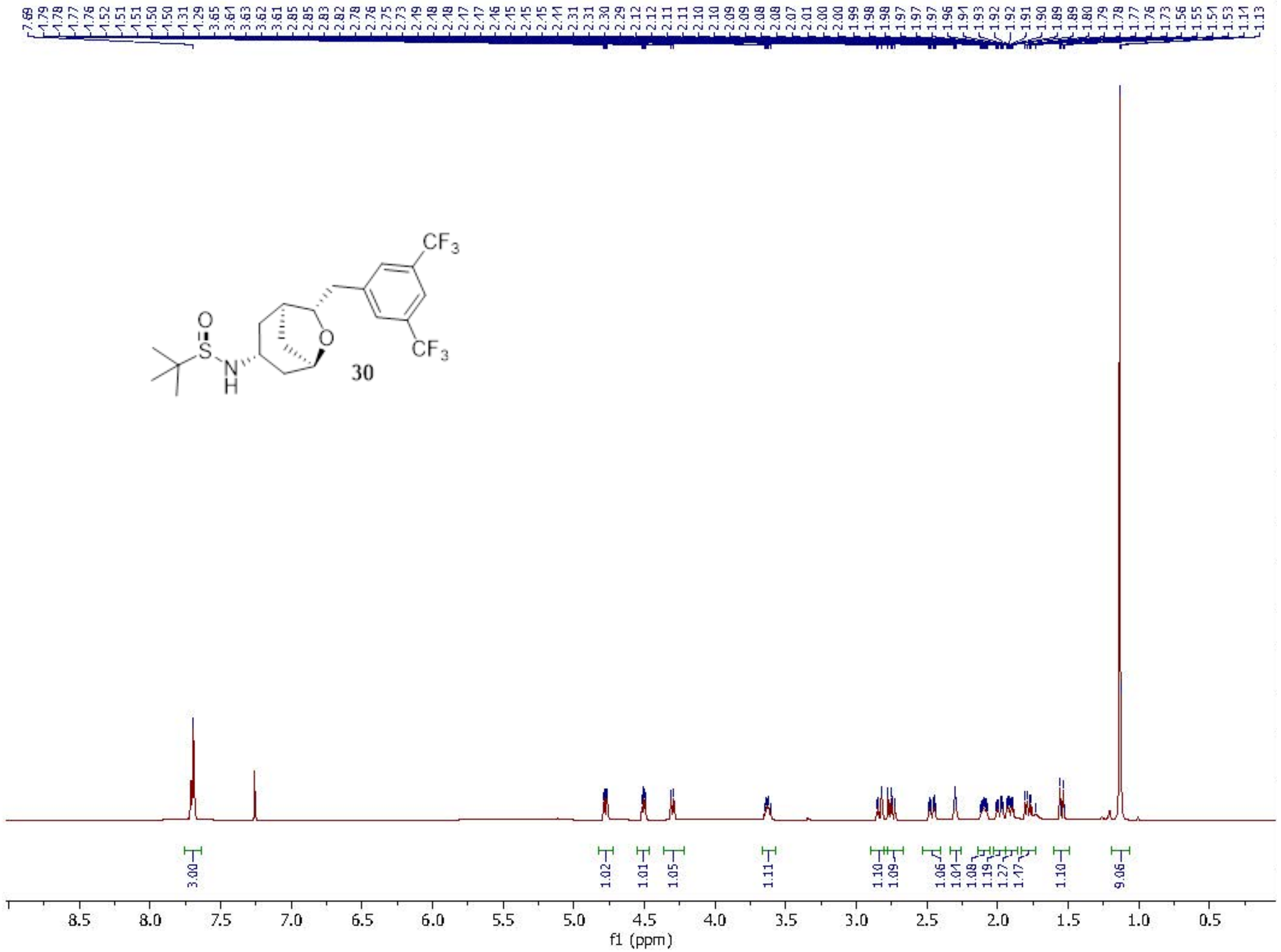
CF3
CF3
O
S
N
H
O
30
3.00
1.02
1.01
1.05
1.11
1.10
1.09
1.06
1.04
1.08
1.19
1.27
1.47
1.10
9.06
8.5
8.0
7.5
7.0
6.5
6.0
5.5
5.0
4.5
4.0
3.5
3.0
2.5
2.0
1.5
1.0
0.5
f1 (ppm)

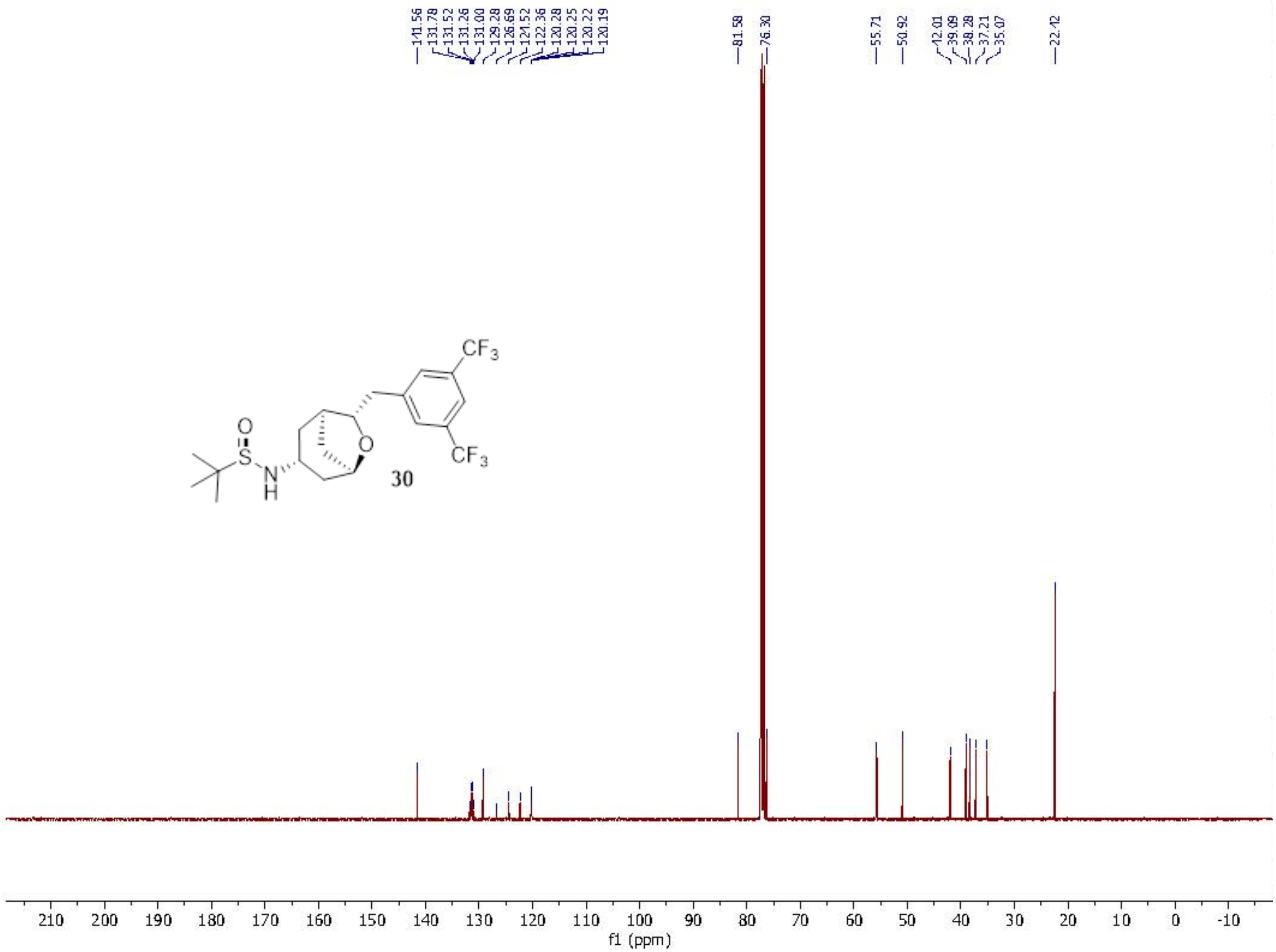

141.56
131.78
131.52
131.26
131.00
129.28
126.69
124.52
122.36
120.28
120.25
120.22
120.19
81.58
76.30
55.71
50.92
42.01
39.09
38.28
37.21
35.07
22.42
CF3
O
S
N
H
O
CF3
30
210 200 190 180 170 160 150 140 130 120 110 100 90 80 70 60 50 40 30 20 10 0 -10
f1 (ppm)

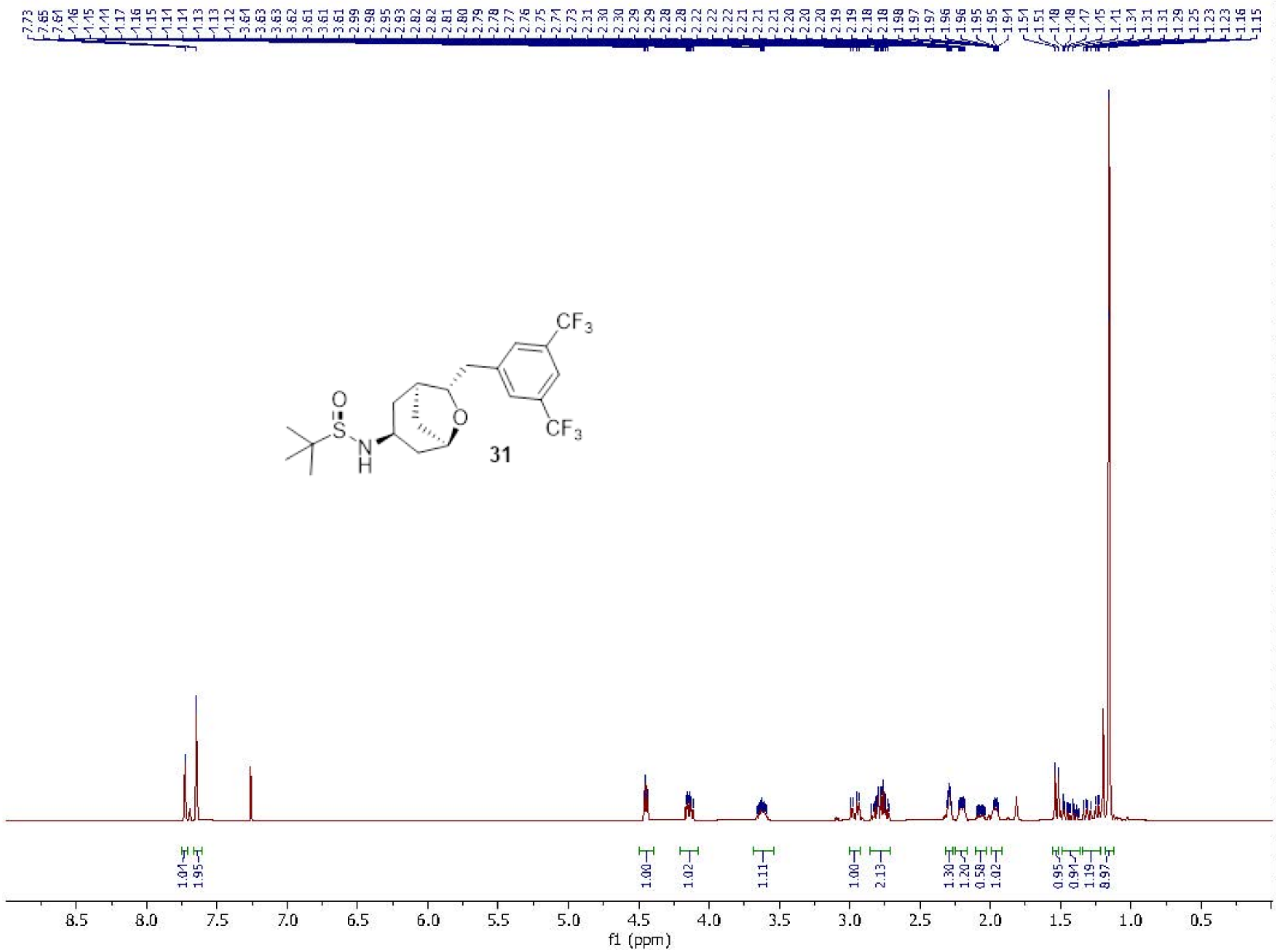

CF3
CF3
O
S
N
H
O
31
8.5 8.0 7.5 7.0 6.5 6.0 5.5 5.0 4.5 4.0 3.5 3.0 2.5 2.0 1.5 1.0 0.5
f1 (ppm)

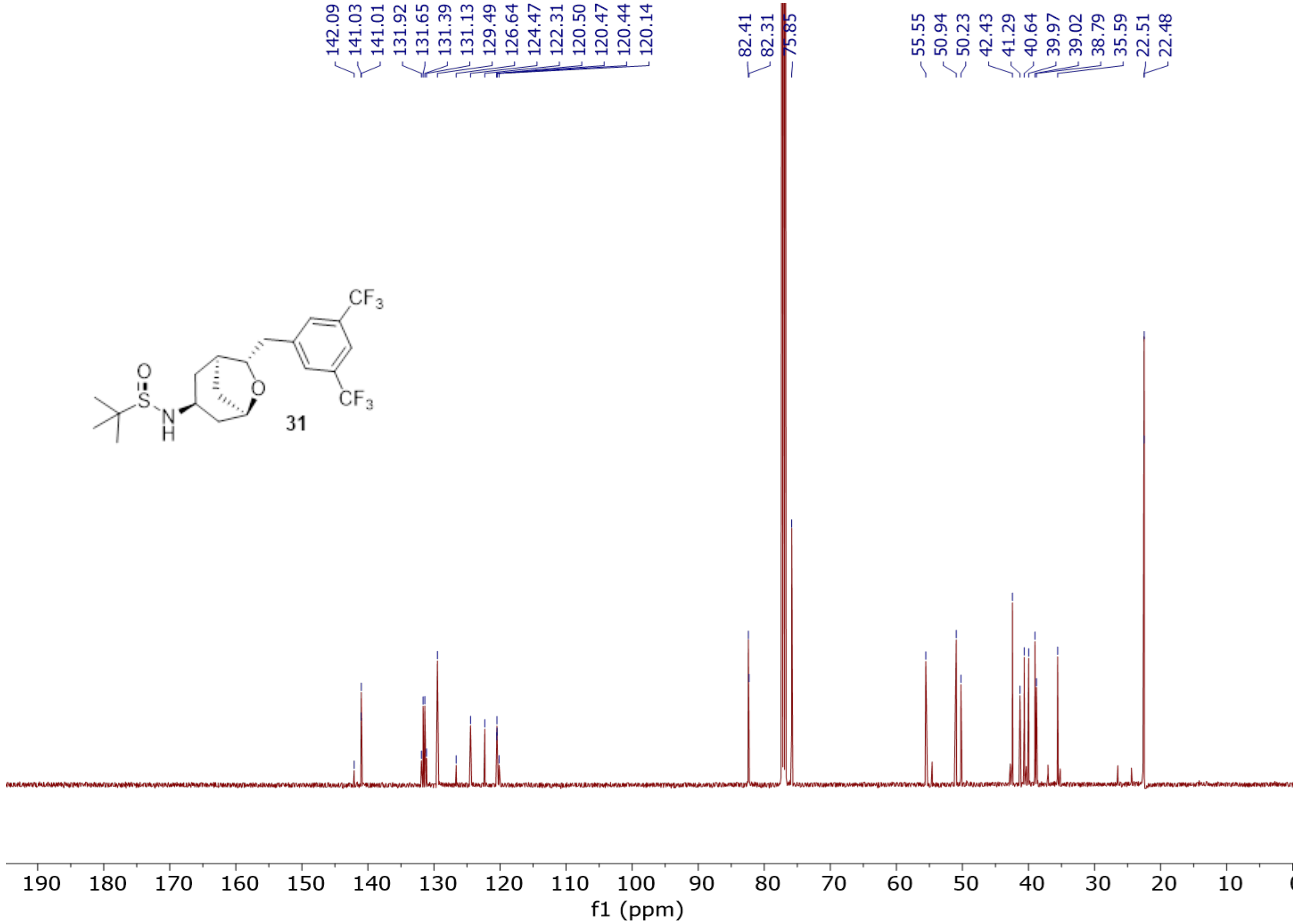
142.09
141.03
141.01
131.92
131.65
131.39
131.13
129.49
126.64
124.47
122.31
120.50
120.47
120.44
120.14
82.41
82.31
75.85
55.55
50.94
50.23
42.43
41.29
40.64
39.97
39.02
38.79
35.59
22.51
22.48
CF3
CF3
O
S
N
H
O
31
190 180 170 160 150 140 130 120 110 100 90 80 70 60 50 40 30 20 10
f1 (ppm)

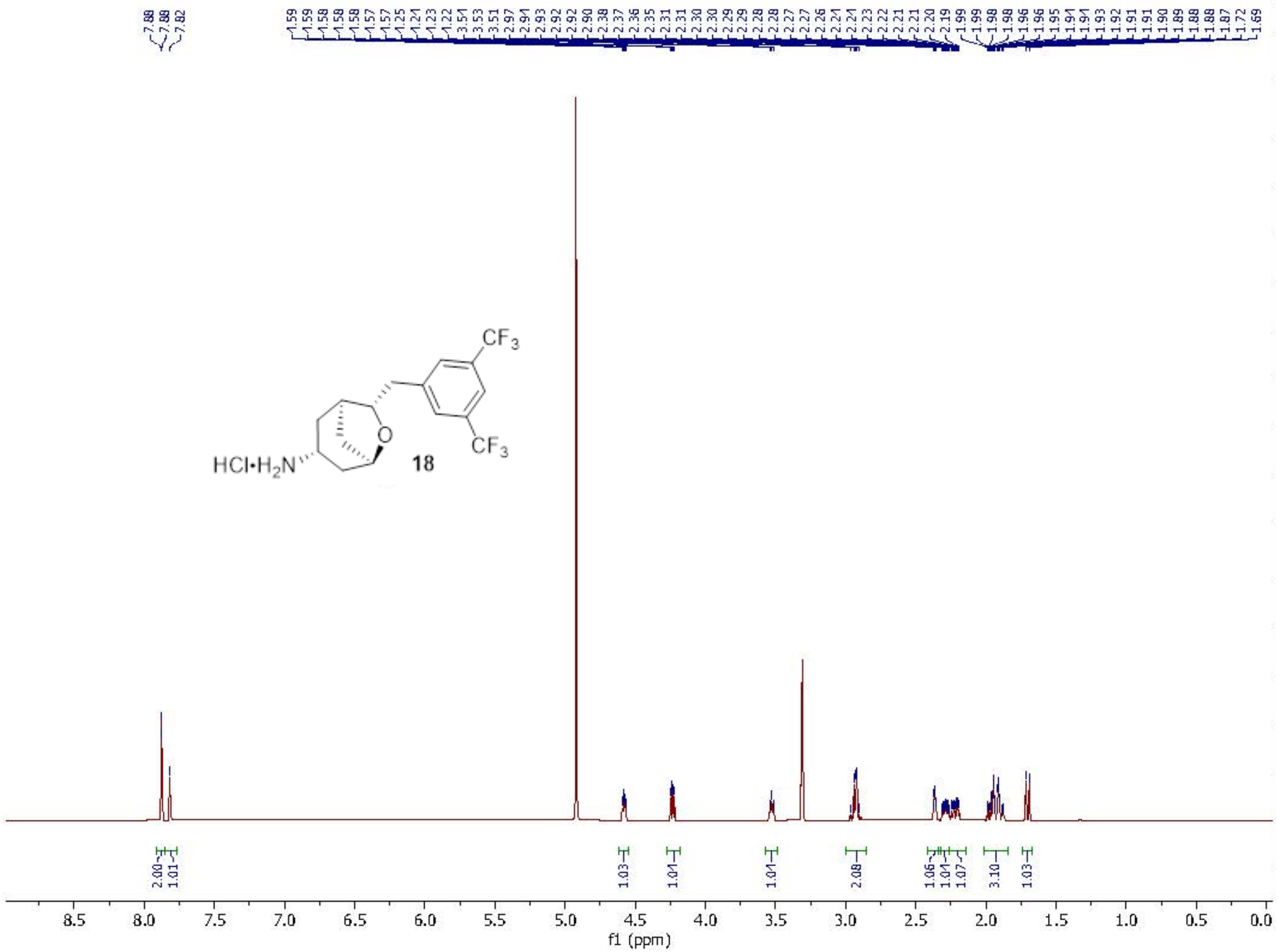

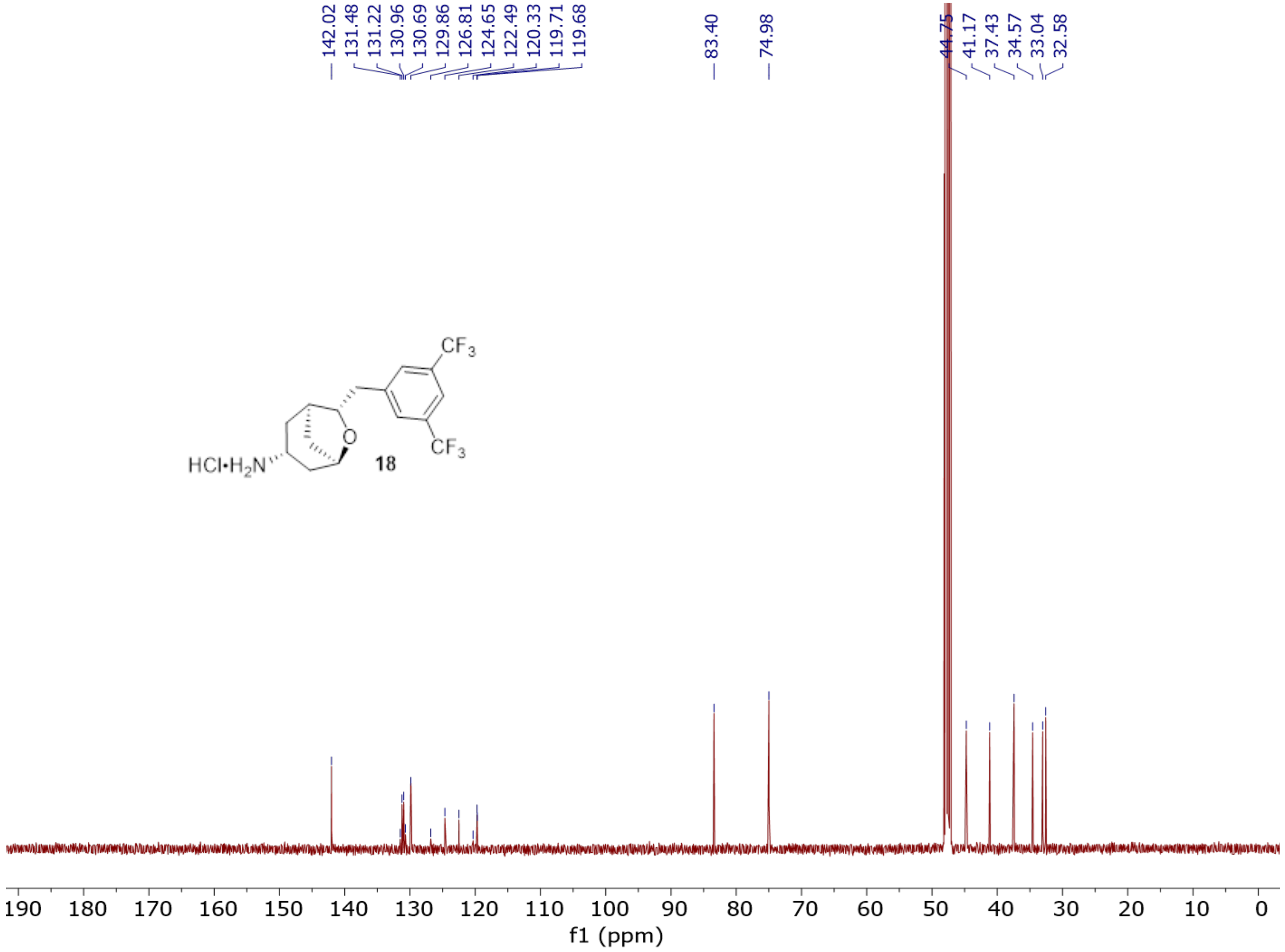
142.02
131.48
131.22
130.96
130.69
129.86
126.81
124.65
122.49
120.33
119.71
119.68
83.40
74.98
44.75
41.17
37.43
34.57
33.04
32.58
CF3
CF3
HCl•H2N
O
18
190 180 170 160 150 140 130 120 110 100 90 80 70 60 50 40 30 20 10 0
f1 (ppm)

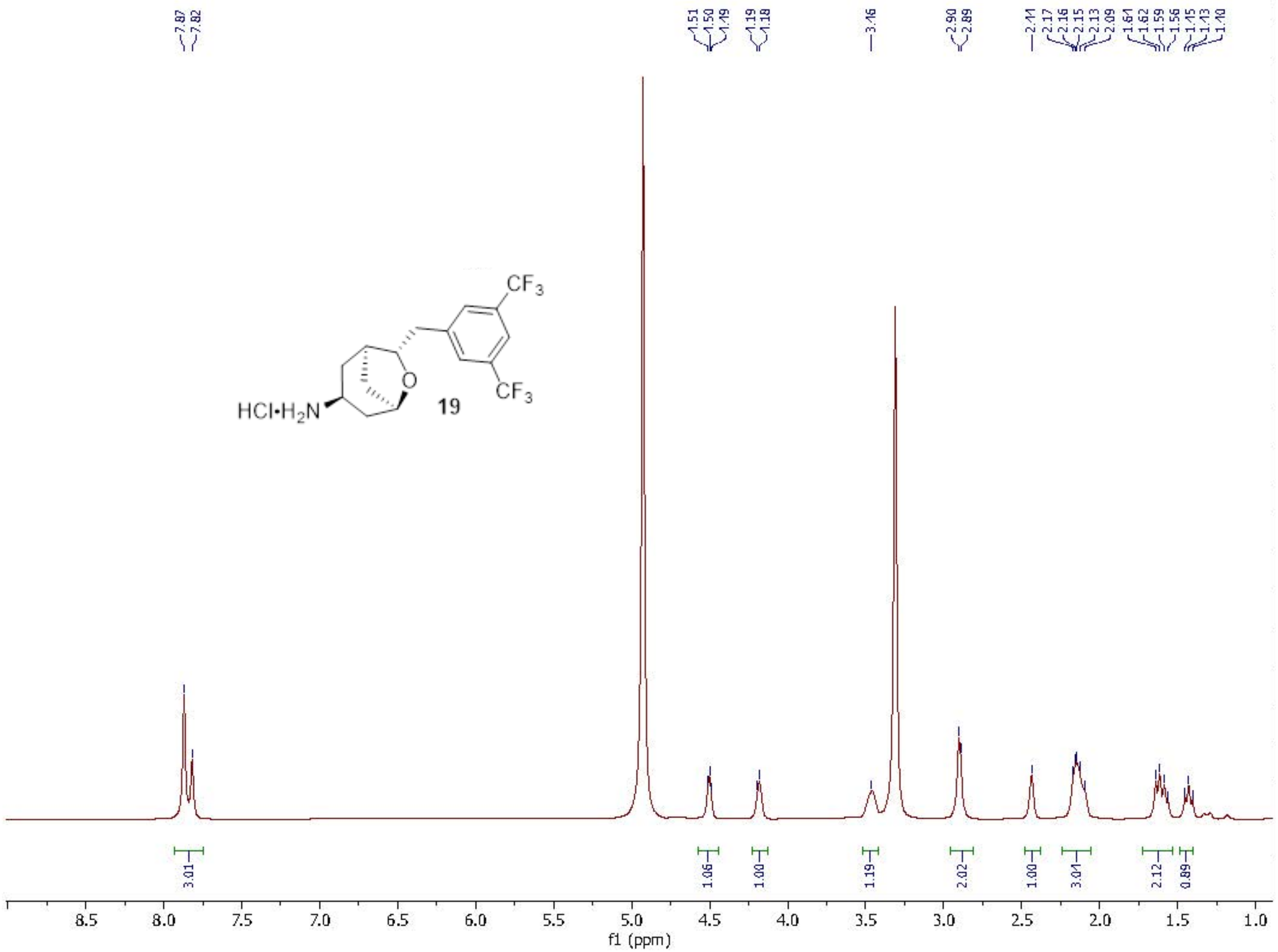

CF3
CF3
HCl•H2N
O
19
3.01
1.06
1.00
1.19
2.02
1.00
3.01
2.12
0.89
8.5
8.0
7.5
7.0
6.5
6.0
5.5
5.0
4.5
4.0
3.5
3.0
2.5
2.0
1.5
1.0
f1 (ppm)

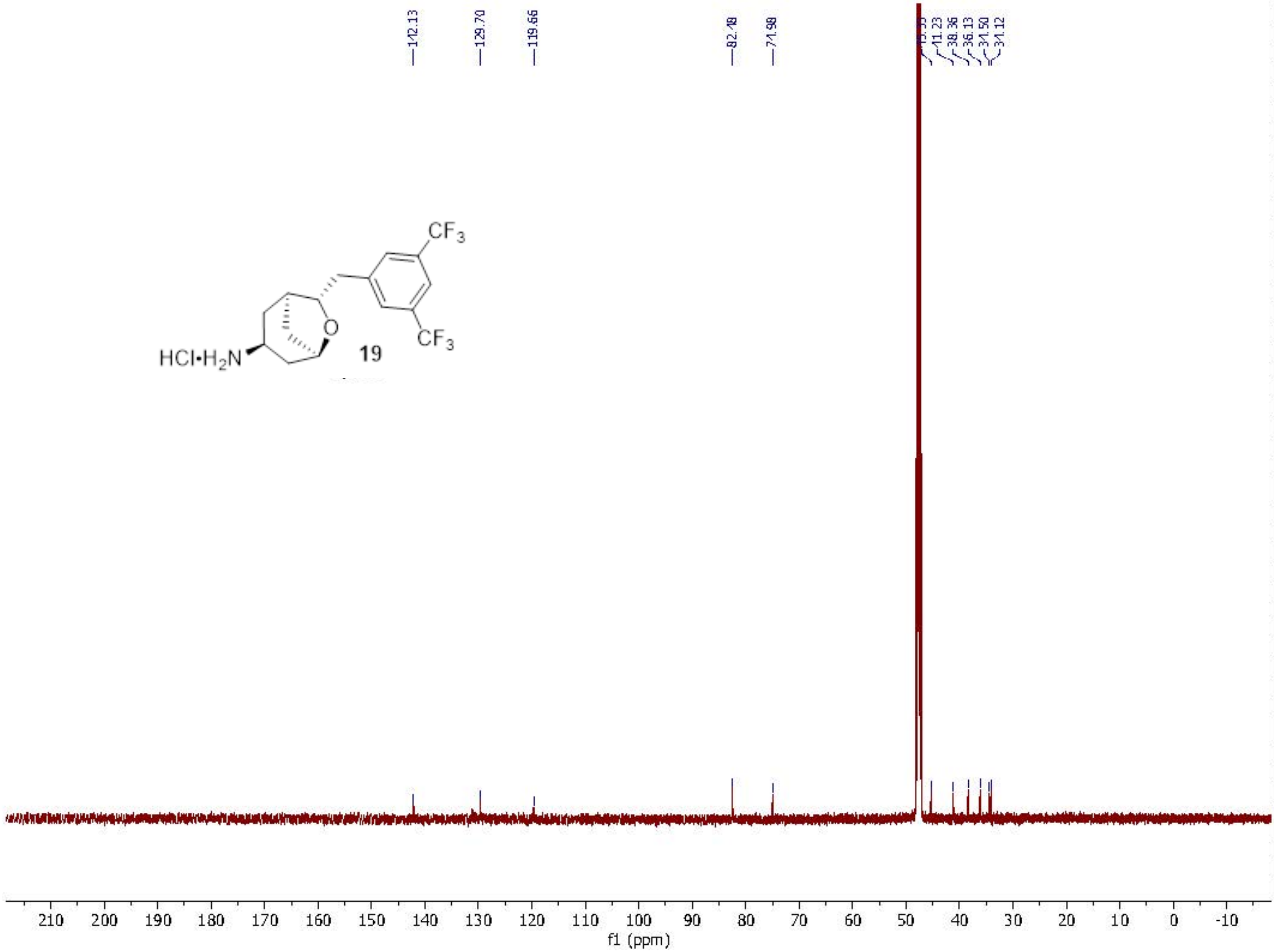
142.13
129.70
119.66
82.48
74.98
41.23
38.36
36.13
34.50
34.12
CF3
CF3
O
HCl•H2N
19
210 200 190 180 170 160 150 140 130 120 110 100 90 80 70 60 50 40 30 20 10 0 -10
f1 (ppm)

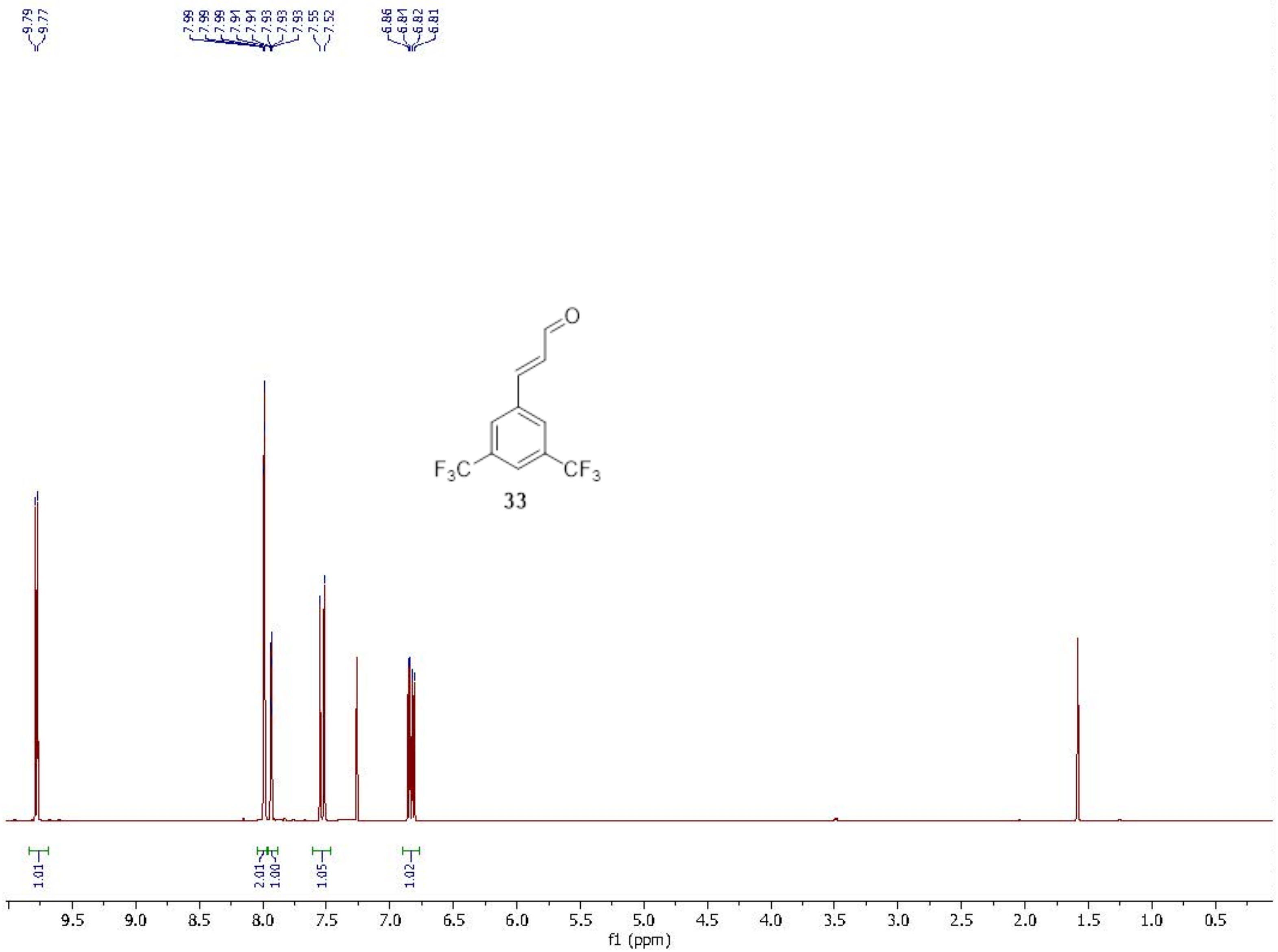
9.79
9.77
7.99
7.99
7.99
7.99
7.91
7.91
7.93
7.93
7.93
7.55
7.52
6.86
6.84
6.82
6.81
O
F3C
CF3
33
1.01
2.01
1.00
1.05
1.02
9.5 9.0 8.5 8.0 7.5 7.0 6.5 6.0 5.5 5.0 4.5 4.0 3.5 3.0 2.5 2.0 1.5 1.0 0.5
f1 (ppm)

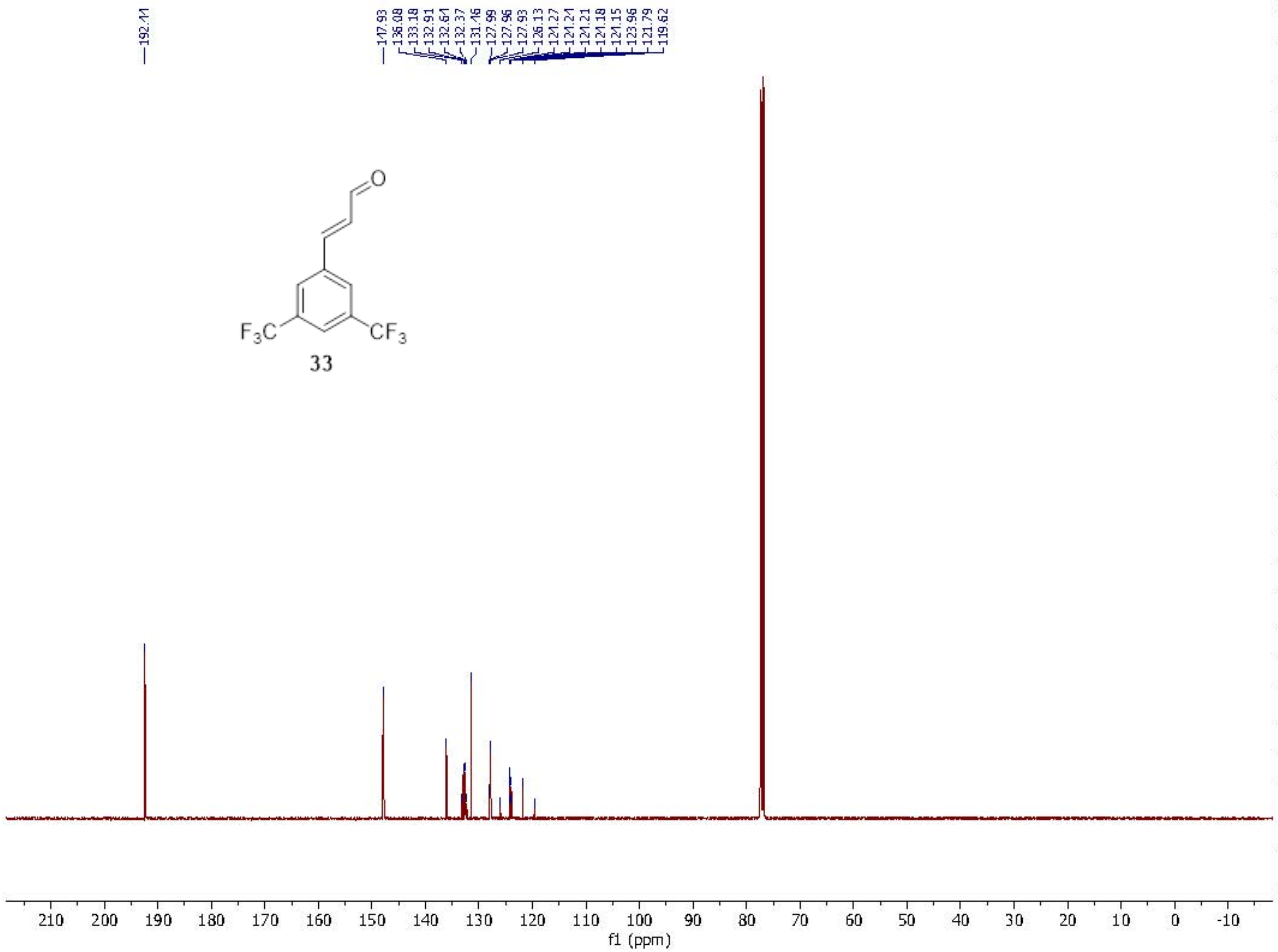

192.11
147.93
136.08
133.18
132.91
132.64
132.37
131.46
127.99
127.96
127.93
126.13
124.27
124.24
124.21
124.18
124.15
123.96
121.79
119.62
O
F3C
CF3
33
210 200 190 180 170 160 150 140 130 120 110 100 90 80 70 60 50 40 30 20 10 0 -10
f1 (ppm)

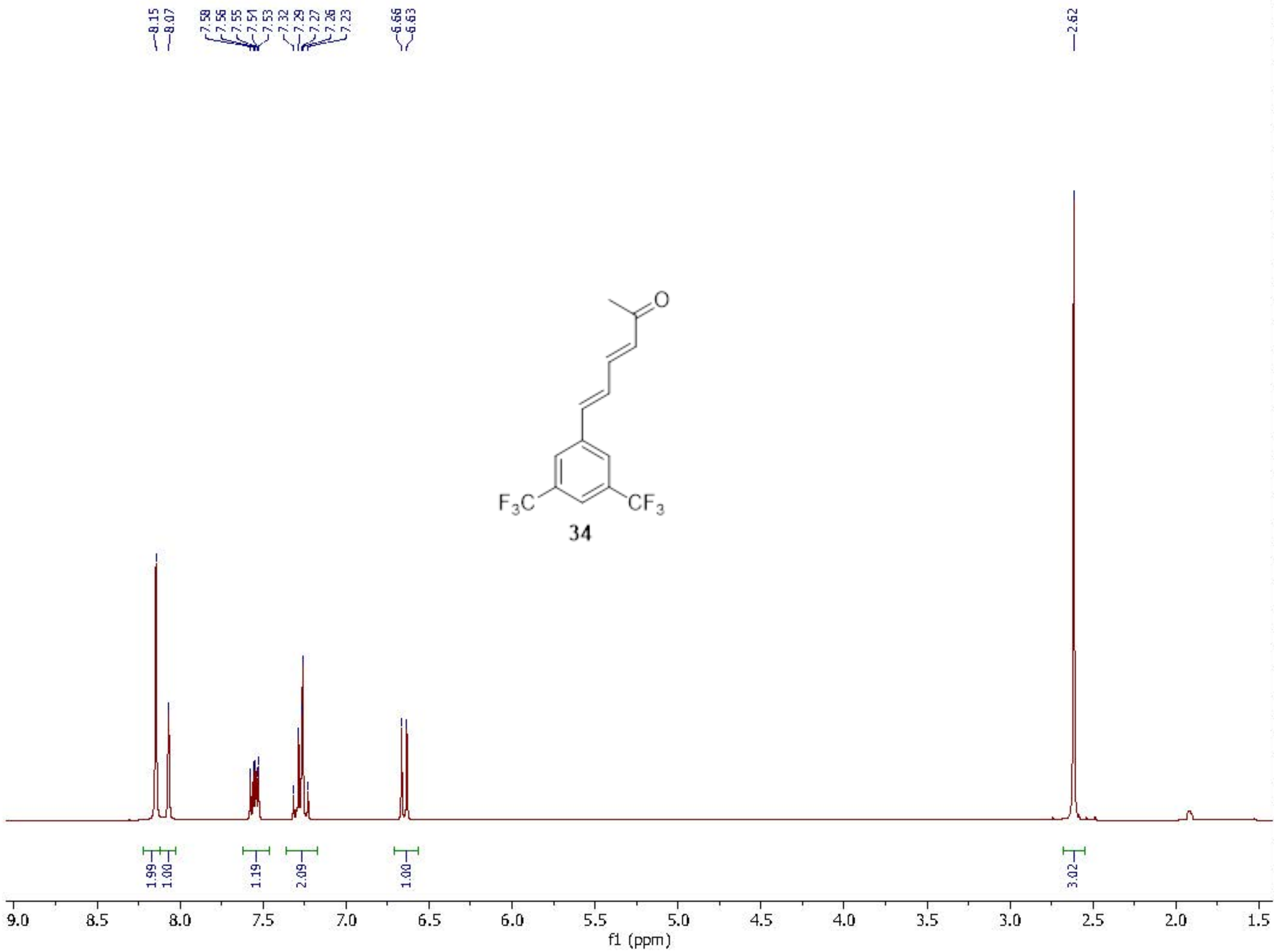
8.15
8.07
7.58
7.56
7.55
7.54
7.53
7.32
7.29
7.27
7.26
7.23
6.66
6.63
2.62
O
F3C
CF3
34
1.99
1.00
1.19
2.09
1.00
3.02
9.0
8.5
8.0
7.5
7.0
6.5
6.0
5.5
5.0
4.5
4.0
3.5
3.0
2.5
2.0
1.5
f1 (ppm)

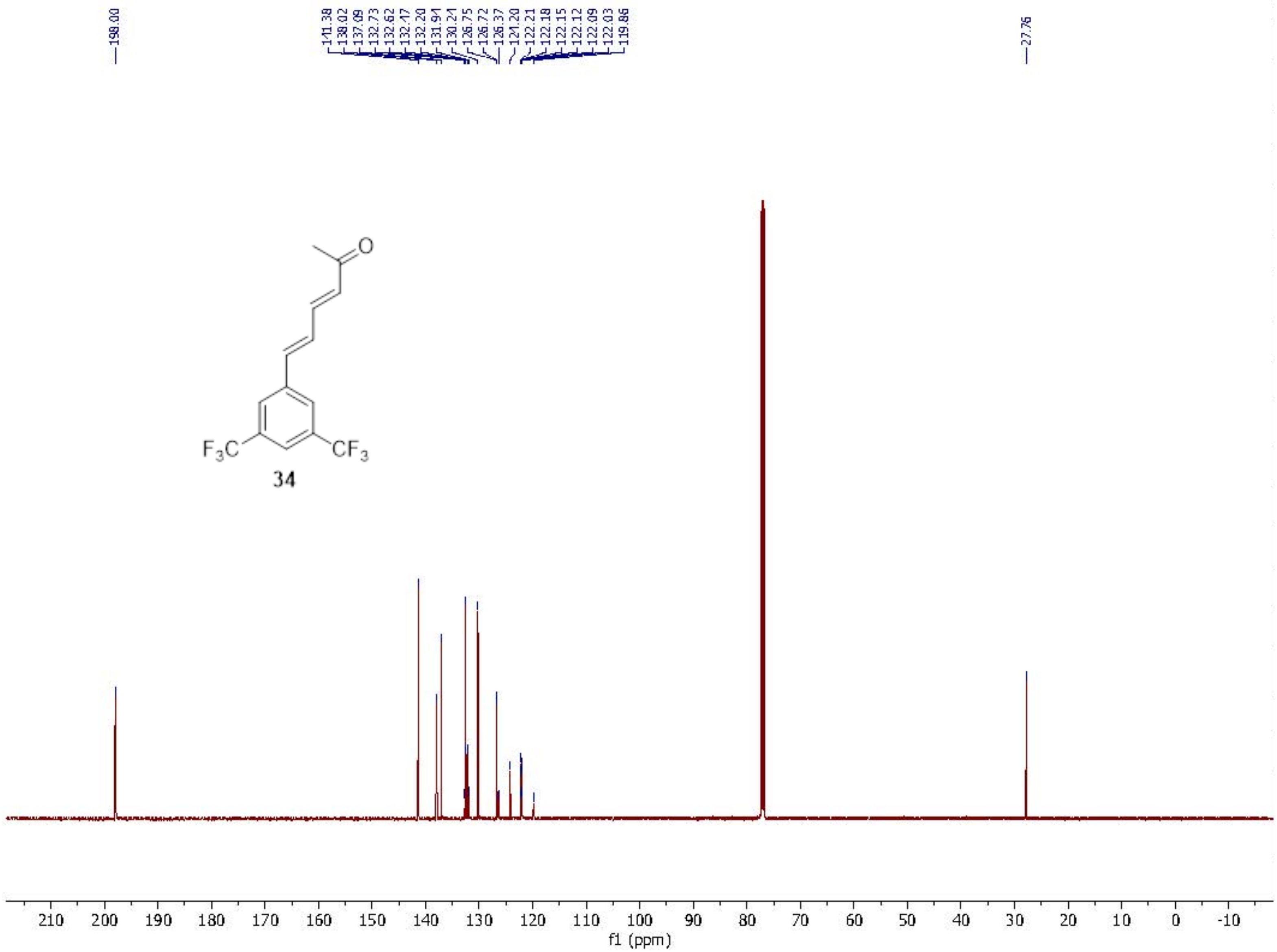
198.00
141.38
138.02
137.09
132.73
132.62
132.47
132.20
131.94
130.24
126.75
126.72
126.37
124.20
122.21
122.18
122.15
122.12
122.09
122.03
119.86
27.76
F3C
CF3
34
210 200 190 180 170 160 150 140 130 120 110 100 90 80 70 60 50 40 30 20 10 0 -10
f1 (ppm)

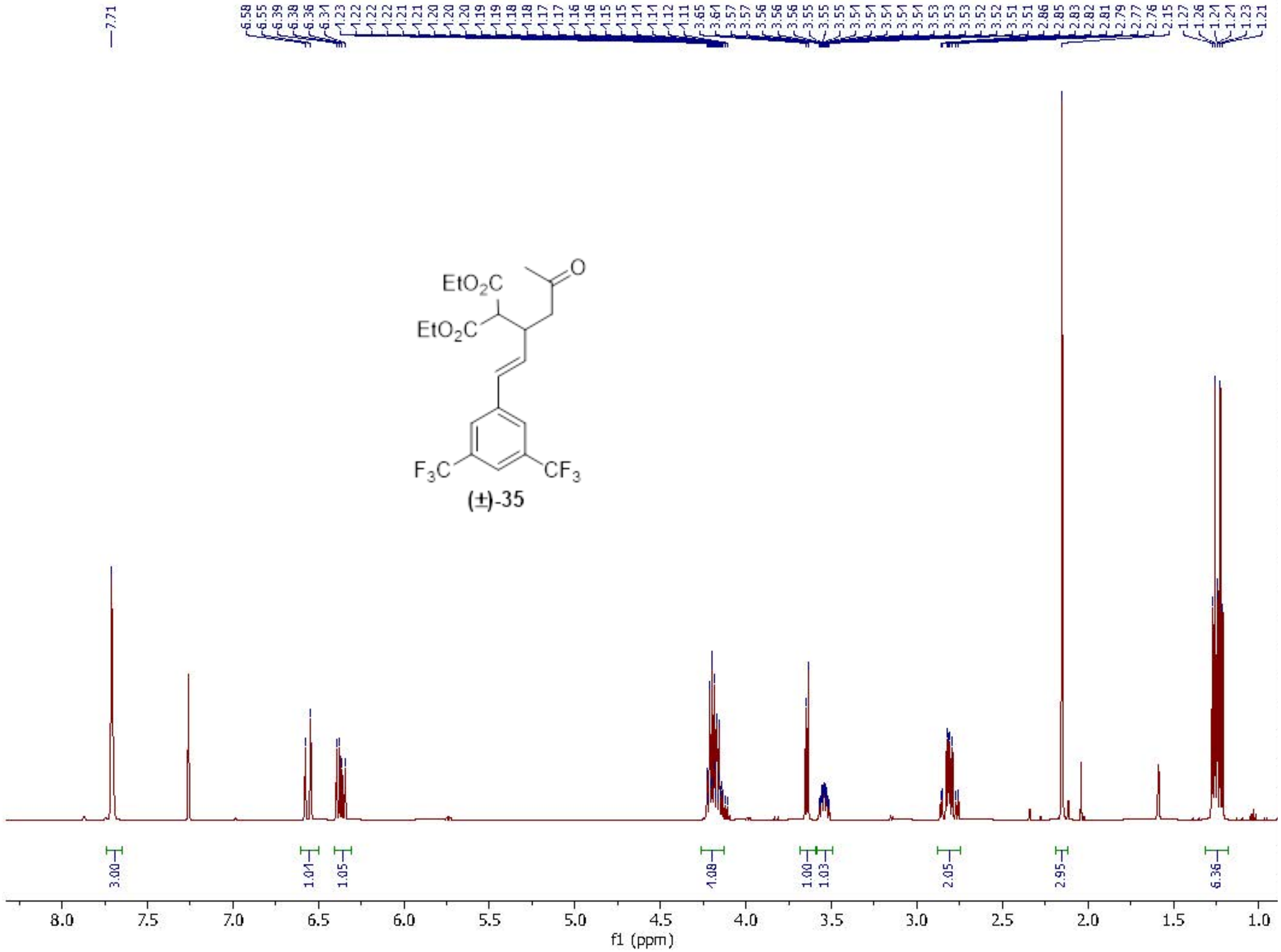
EtO2C
EtO2C
O
F3C
CF3
(±)-35
8.0 7.5 7.0 6.5 6.0 5.5 5.0 4.5 4.0 3.5 3.0 2.5 2.0 1.5 1.0
f1 (ppm)

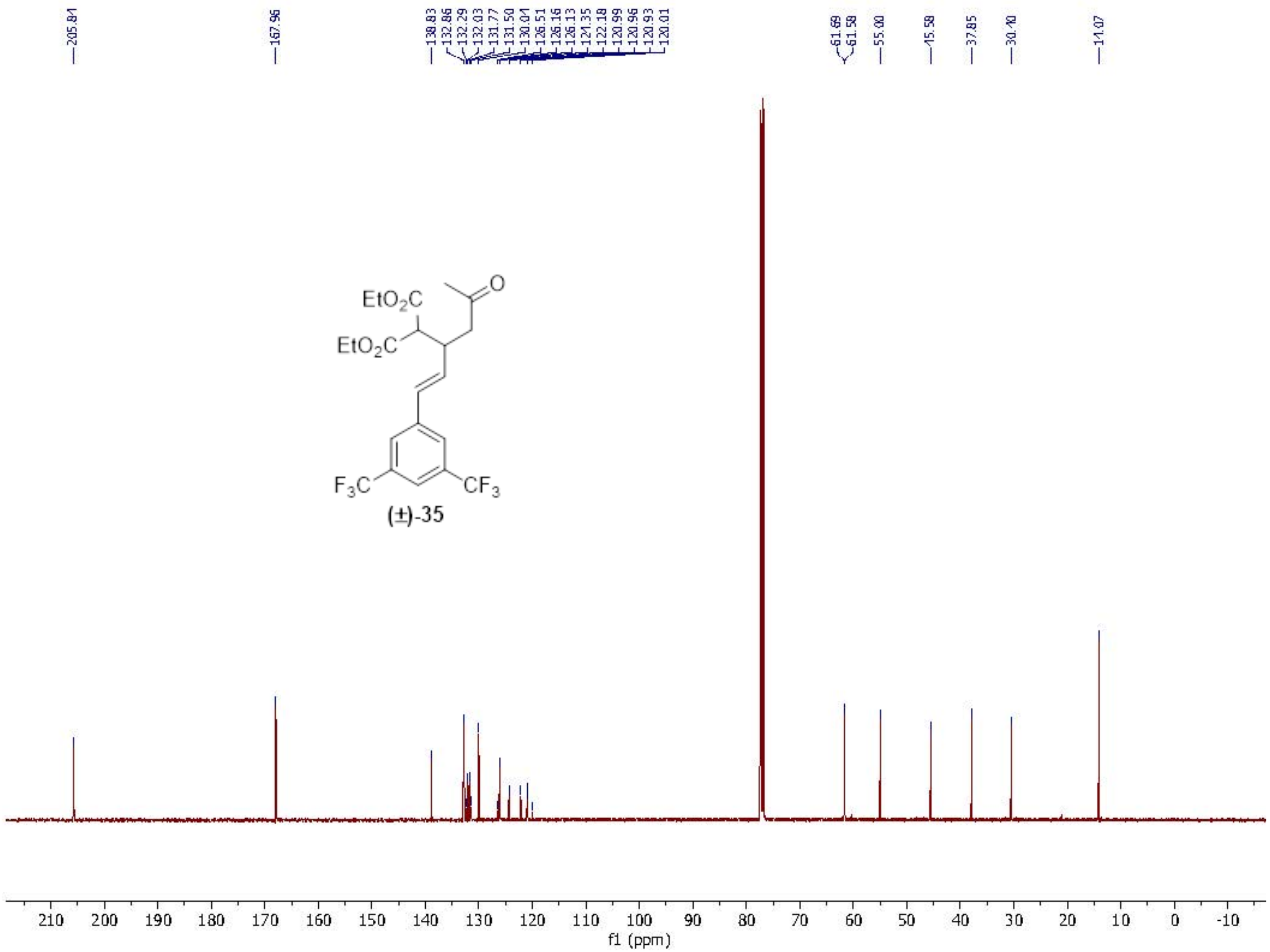
205.84
167.96
138.83
132.86
132.29
132.03
131.77
131.50
130.04
126.51
126.16
126.13
124.35
122.18
120.99
120.96
120.93
120.01
61.69
61.58
55.00
45.58
37.85
30.40
14.07
EtO2C
EtO2C
O
F3C
CF3
(±)-35
210 200 190 180 170 160 150 140 130 120 110 100 90 80 70 60 50 40 30 20 10 0 -10
f1 (ppm)

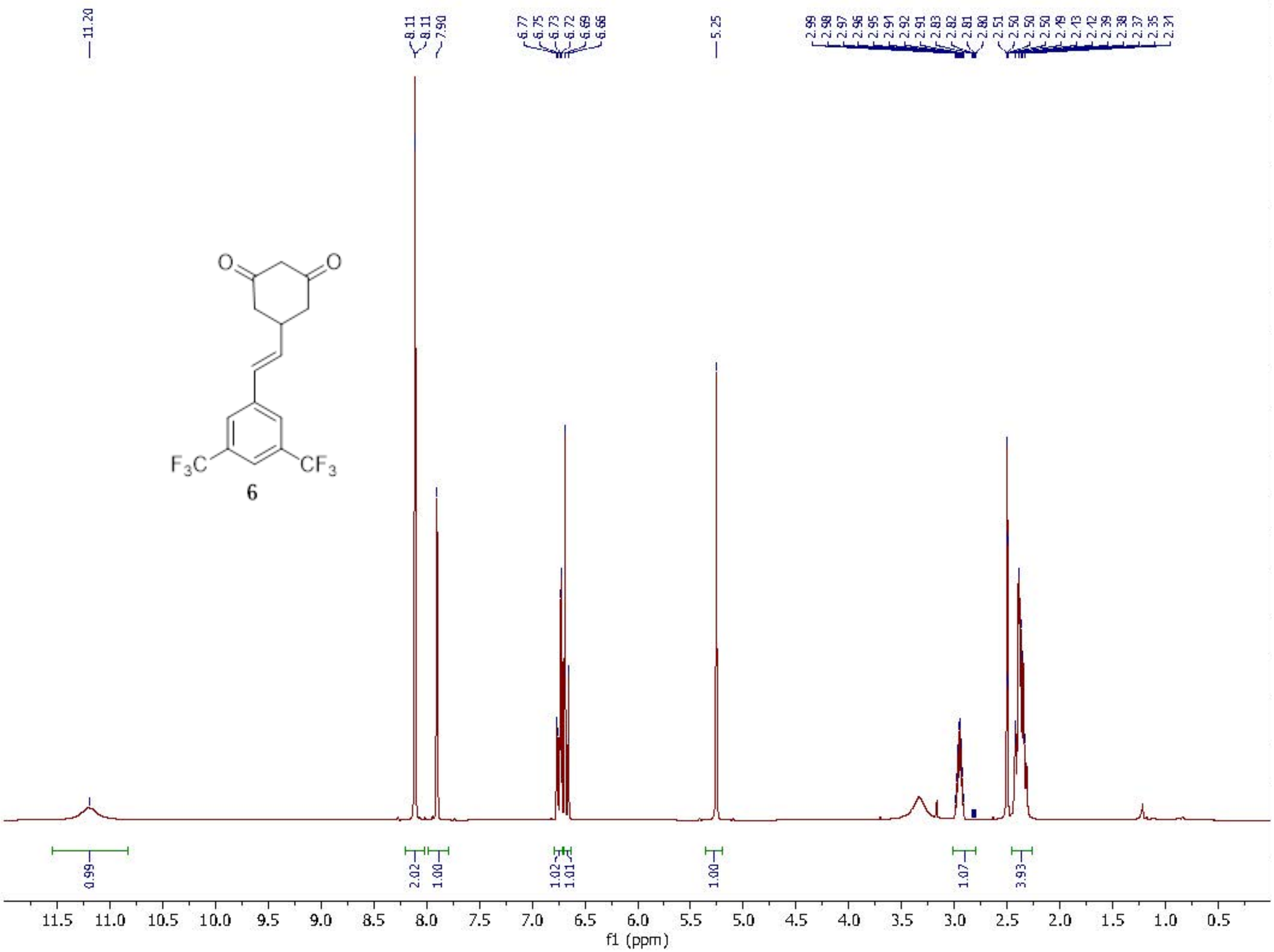

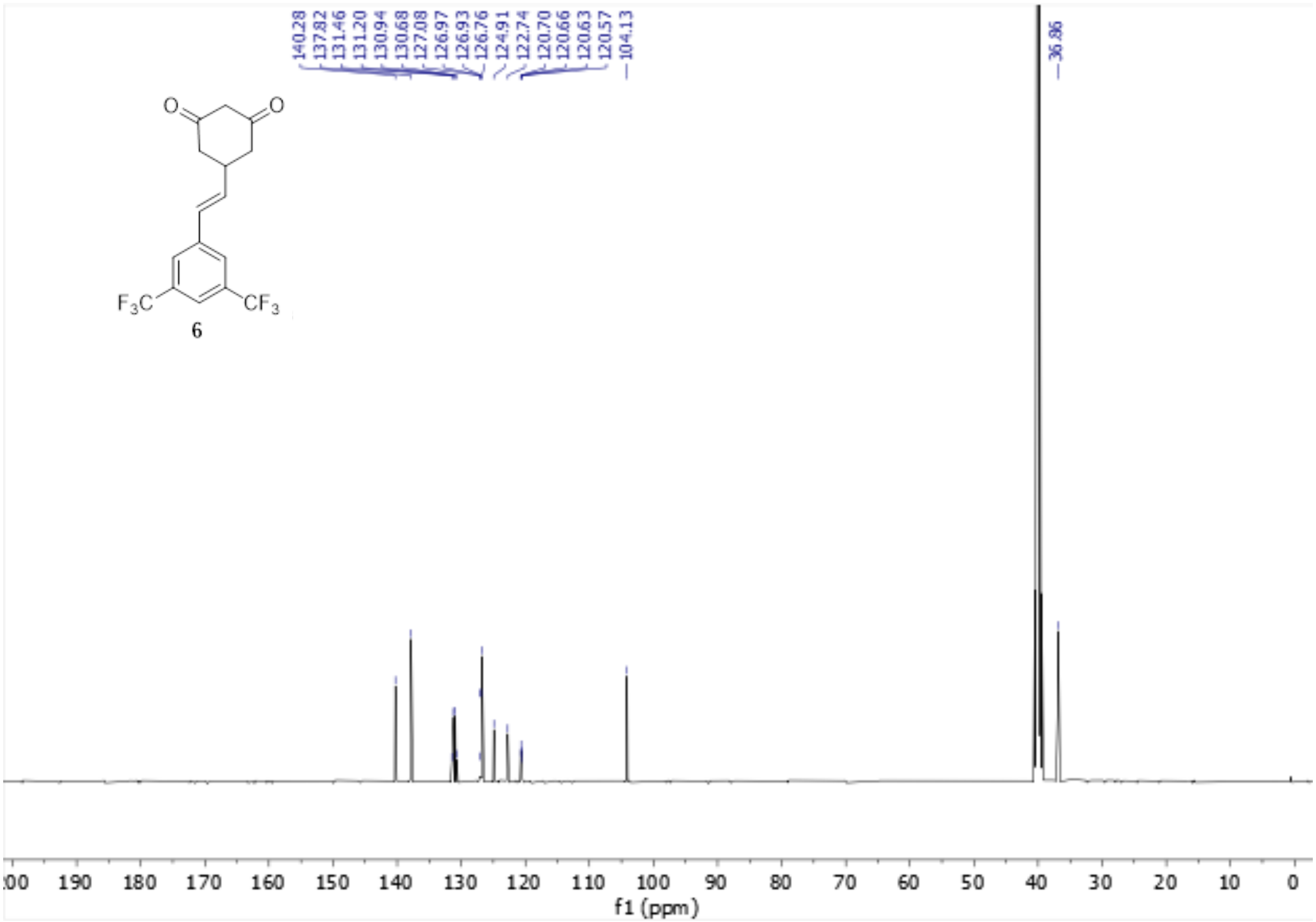
140.28
137.82
131.46
131.20
130.94
130.68
127.08
126.97
126.93
126.76
124.91
122.74
120.70
120.66
120.63
120.57
104.13
36.86
O
O
F3C
CF3
6
190 180 170 160 150 140 130 120 110 100 90 80 70 60 50 40 30 20 10 0
f1 (ppm)

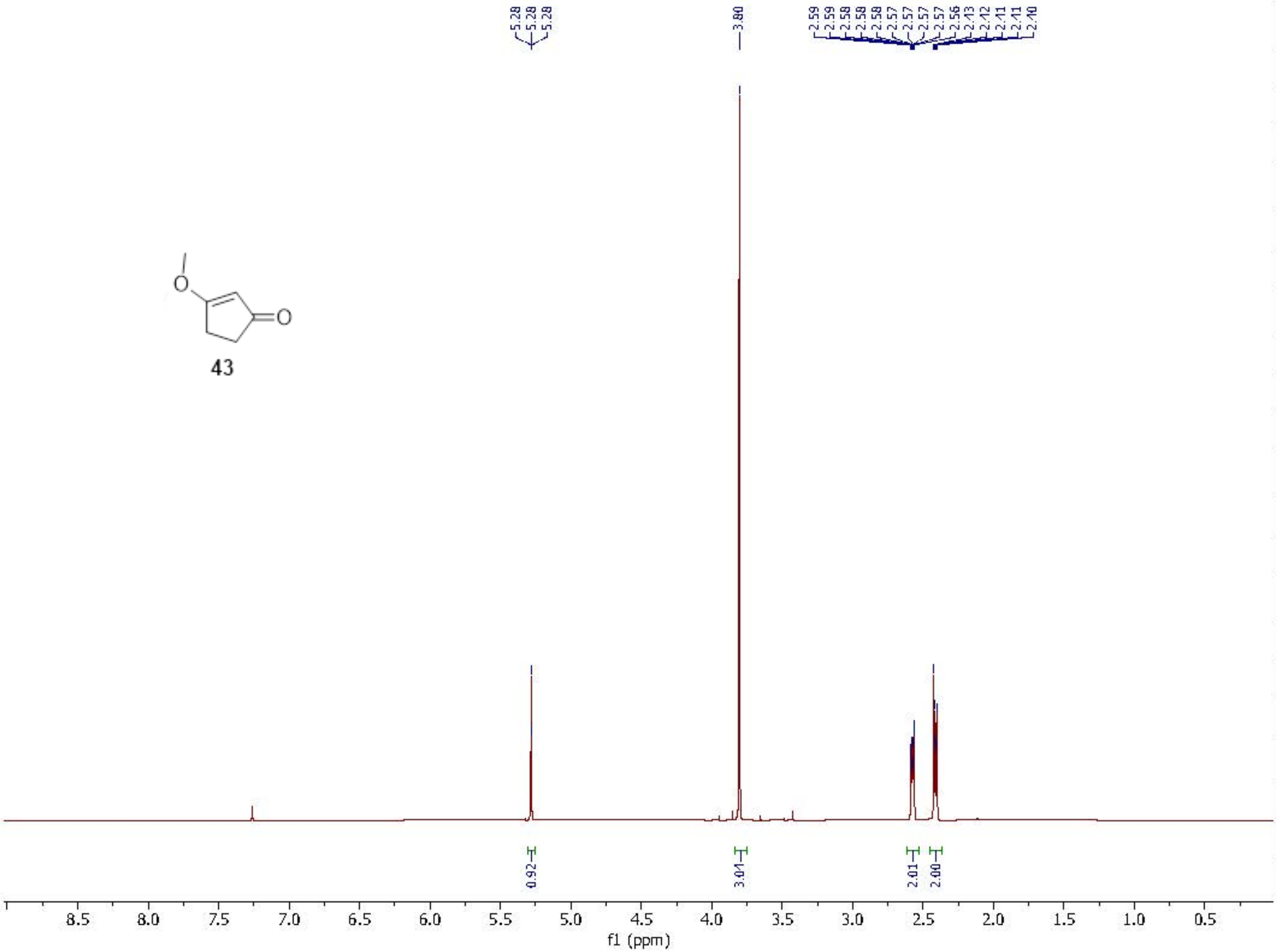
O
O
43
5.28
5.28
5.28
3.80
2.59
2.59
2.58
2.58
2.58
2.57
2.57
2.57
2.57
2.56
2.13
2.12
2.11
2.11
2.10
0.92
3.04
2.01
2.00
8.5
8.0
7.5
7.0
6.5
6.0
5.5
5.0
4.5
4.0
3.5
3.0
2.5
2.0
1.5
1.0
0.5
f1 (ppm)

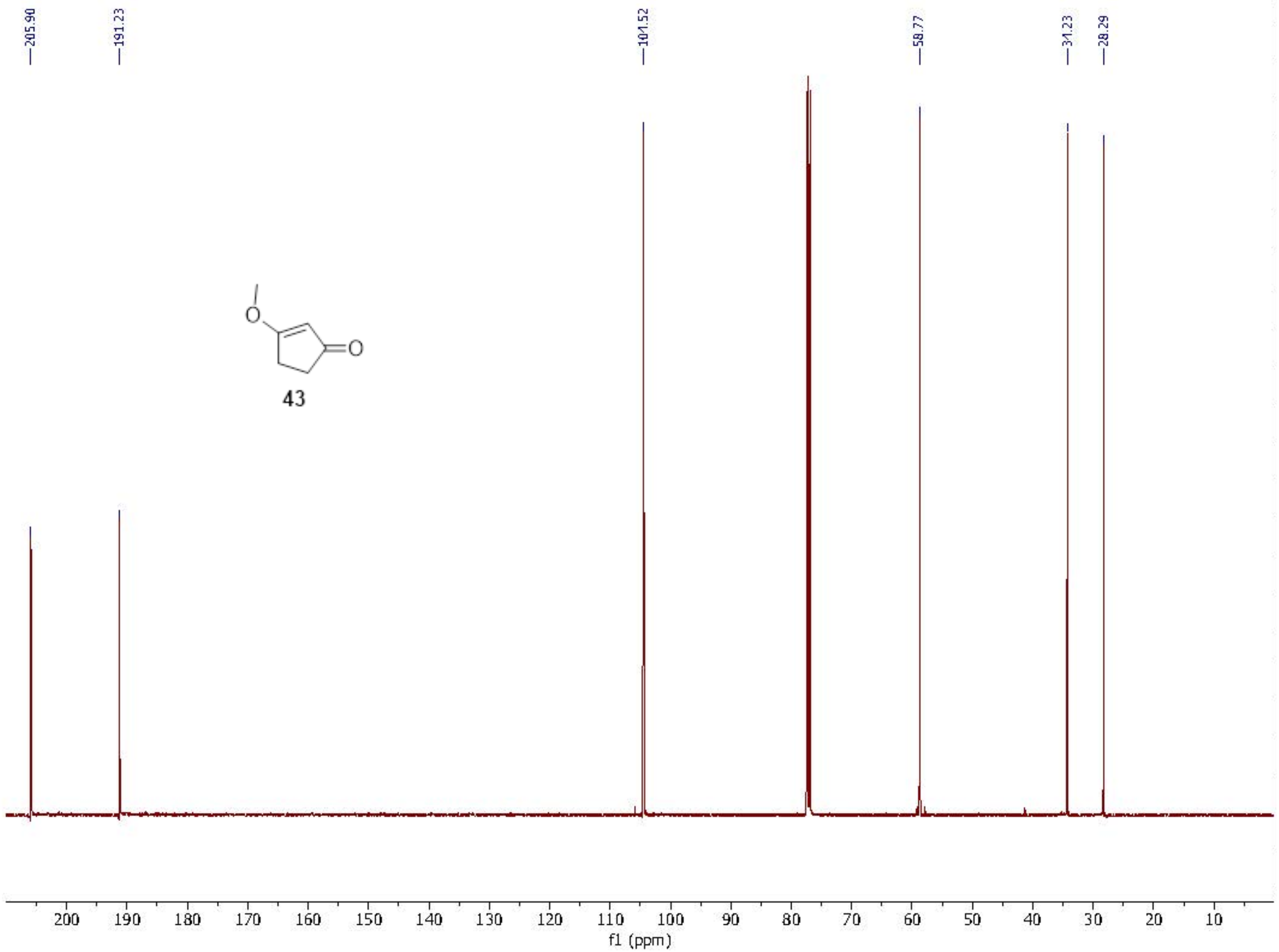
205.90
191.23
104.52
58.77
34.23
28.29
43
200
190
180
170
160
150
140
130
120
110
100
90
80
70
60
50
40
30
20
10
f1 (ppm)

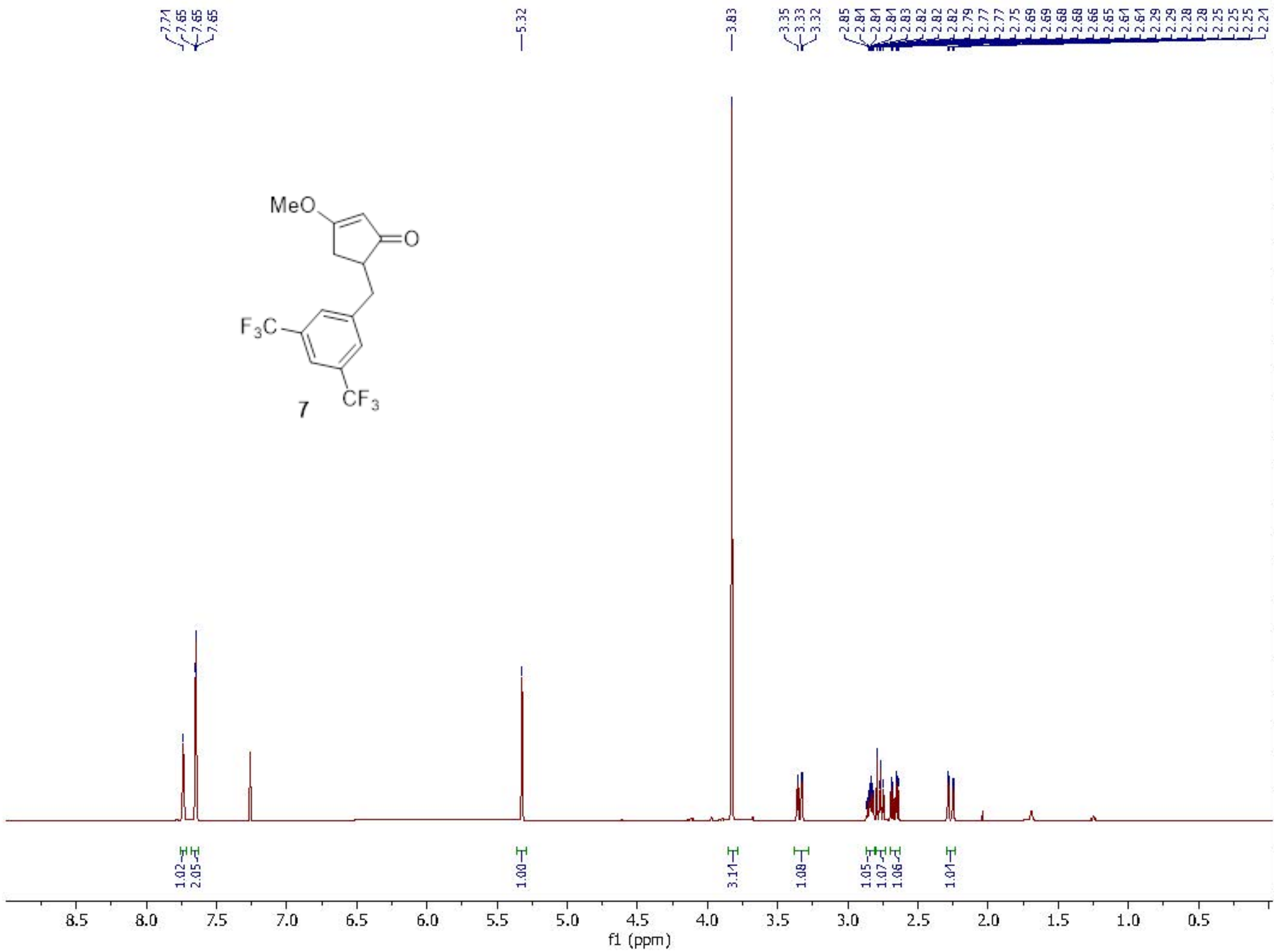

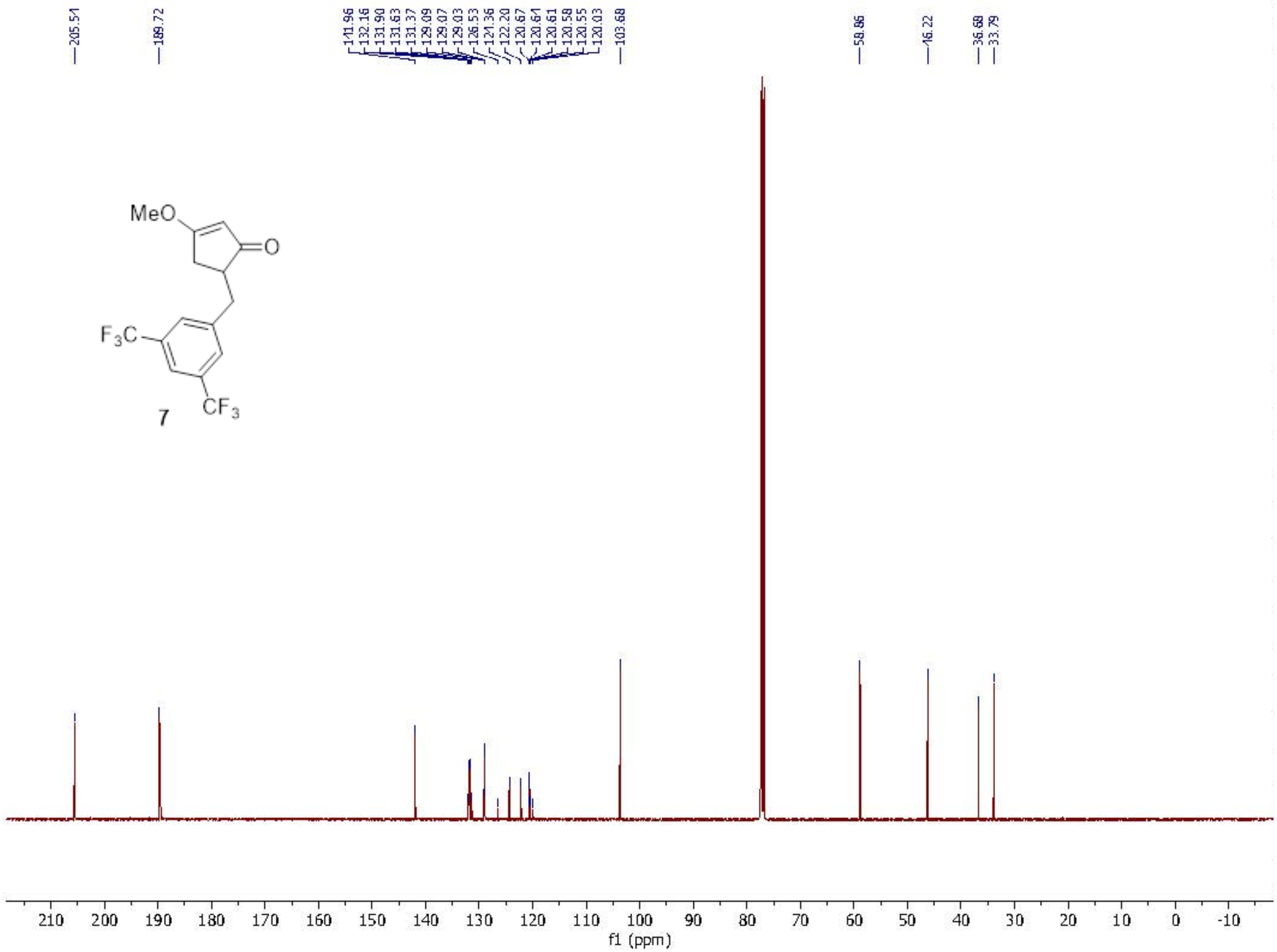

205.51
189.72
141.96
132.16
131.90
131.63
131.37
129.09
129.07
129.03
126.53
124.36
122.20
120.67
120.64
120.61
120.58
120.55
120.03
103.68
58.86
46.22
36.68
33.79
MeO
O
F3C
CF3
7
f1 (ppm)

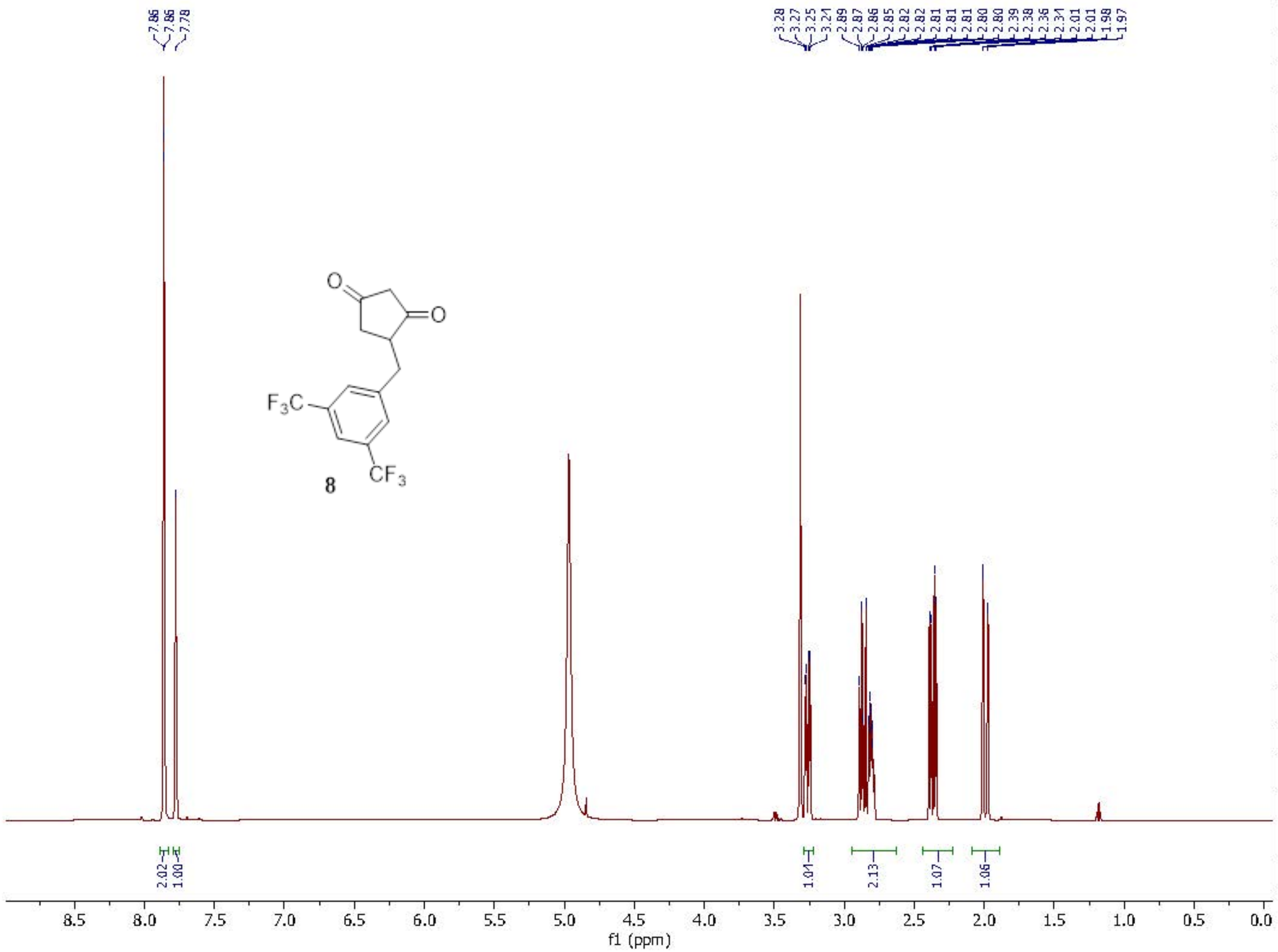
7.86
7.86
7.78
3.28
3.27
3.25
3.24
2.89
2.87
2.86
2.85
2.82
2.82
2.81
2.81
2.81
2.80
2.80
2.39
2.38
2.36
2.34
2.01
2.01
1.98
1.97
2.02
1.00
1.04
2.13
1.07
1.06
8.5
8.0
7.5
7.0
6.5
6.0
5.5
5.0
4.5
4.0
3.5
3.0
2.5
2.0
1.5
1.0
0.5
0.0
f1 (ppm)
O
O
F3C
CF3
8

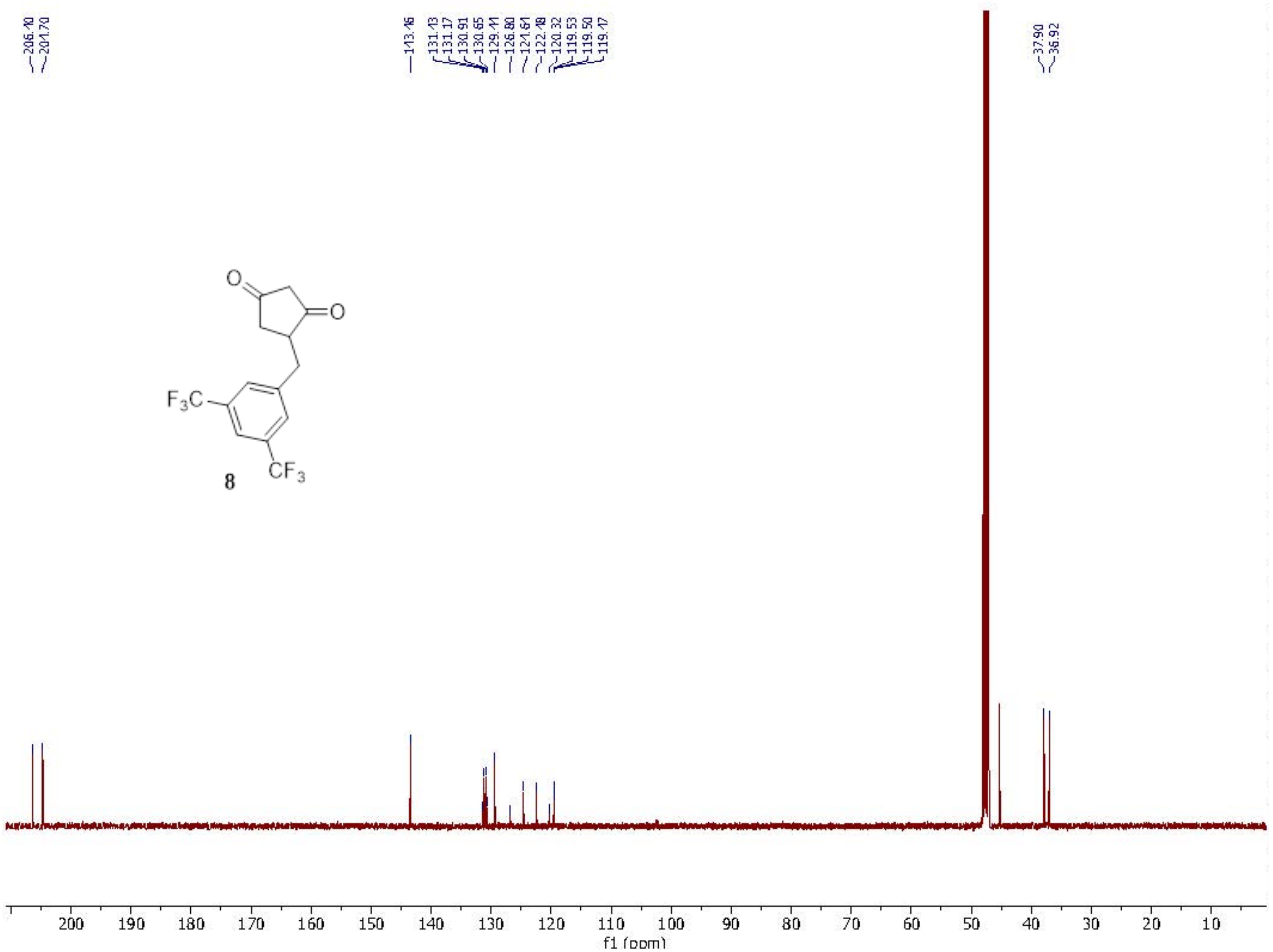
206.40
204.70
143.46
131.43
131.17
130.91
130.65
129.41
126.80
124.61
122.48
120.32
119.53
119.50
119.47
37.90
36.92
O
O
F3C
CF3
8
200 190 180 170 160 150 140 130 120 110 100 90 80 70 60 50 40 30 20 10
f1 (ppm)

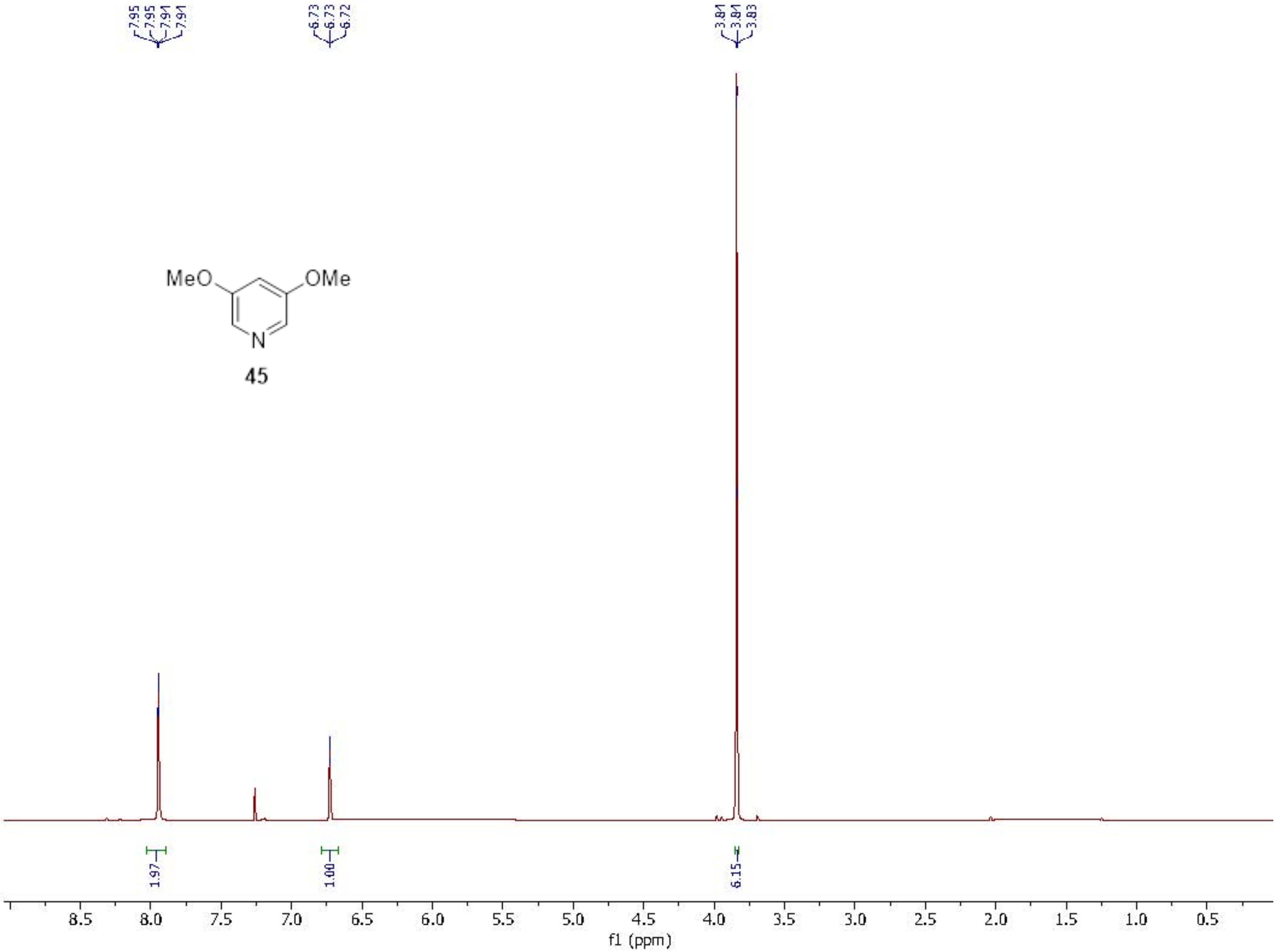

7.95
7.95
7.94
7.94
6.73
6.73
6.72
3.84
3.84
3.83
MeO
OMe
N
1.97
1.00
6.15
8.5
8.0
7.5
7.0
6.5
6.0
5.5
5.0
4.5
4.0
3.5
3.0
2.5
2.0
1.5
1.0
0.5
f1 (ppm)

45

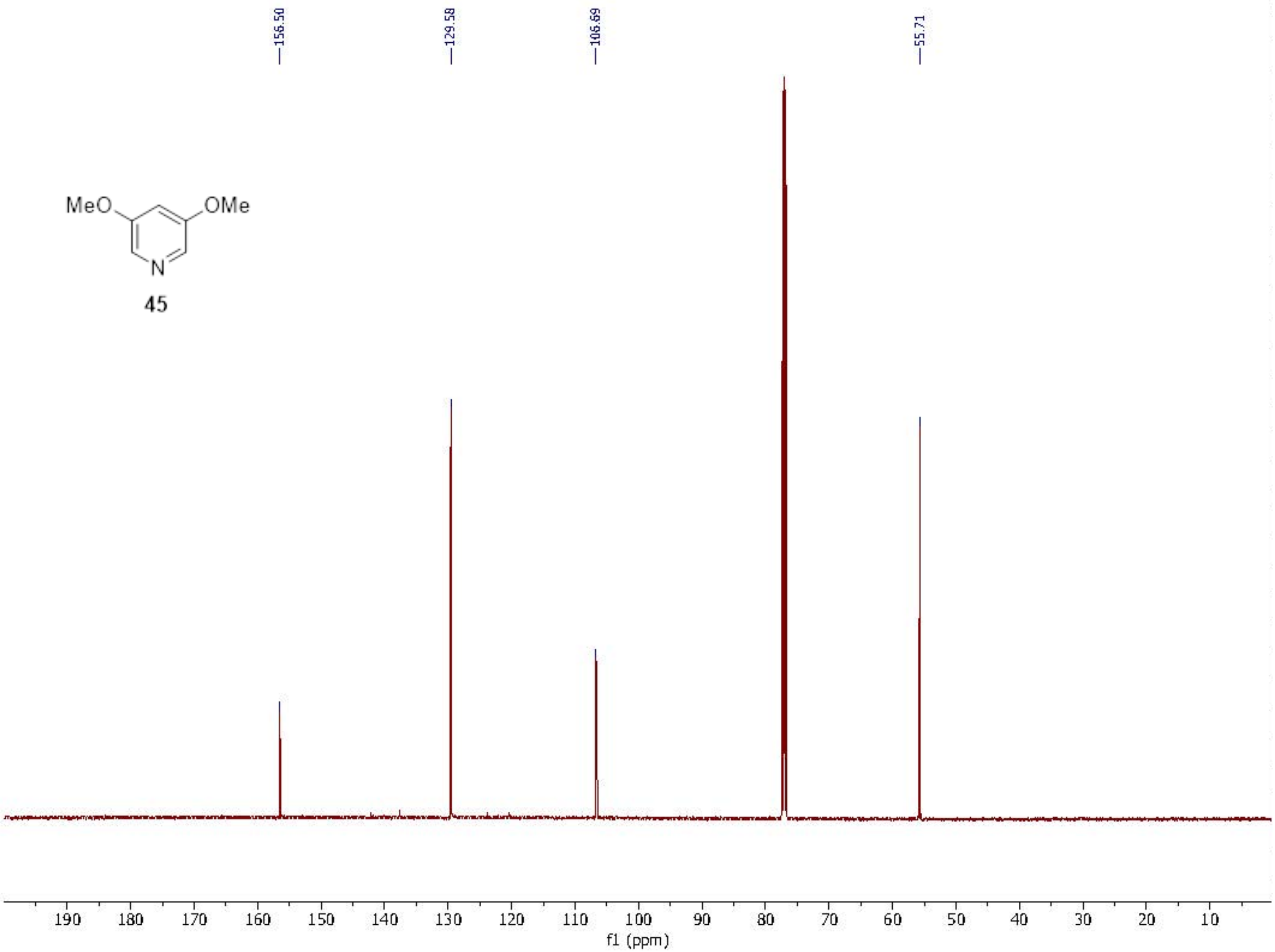
156.50
129.58
106.69
55.71
MeO
OMe
N
45
190 180 170 160 150 140 130 120 110 100 90 80 70 60 50 40 30 20 10
f1 (ppm)

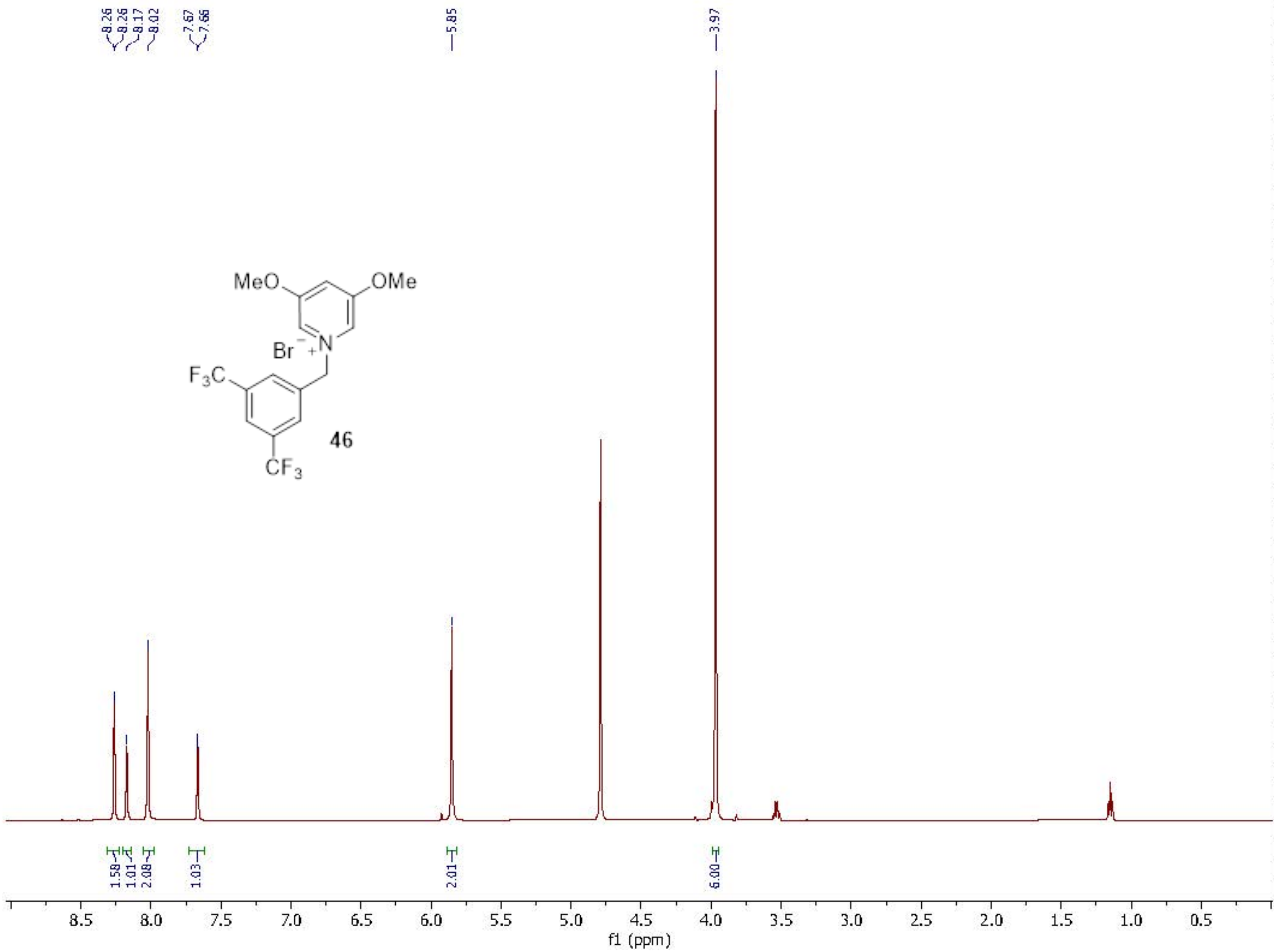
8.26
8.26
8.17
8.02
7.67
7.66
5.85
3.97
MeO
OMe
Br
N
F3C
CF3
46
1.58
1.01
2.08
1.03
2.01
6.00
8.5
8.0
7.5
7.0
6.5
6.0
5.5
5.0
4.5
4.0
3.5
3.0
2.5
2.0
1.5
1.0
0.5
f1 (ppm)

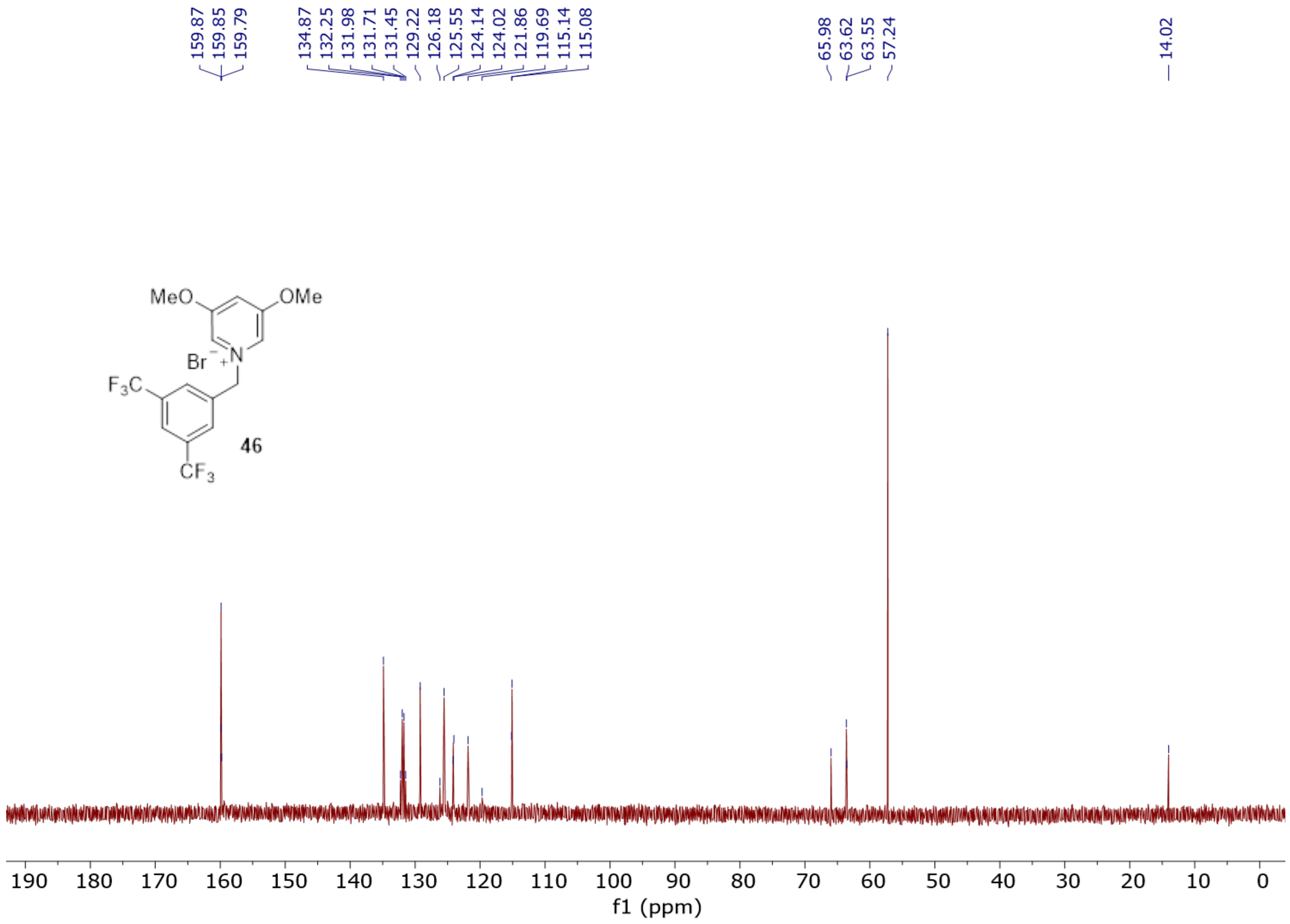
159.87
159.85
159.79
134.87
132.25
131.98
131.71
131.45
129.22
126.18
125.55
124.14
124.02
121.86
119.69
115.14
115.08
65.98
63.62
63.55
57.24
14.02
MeO
OMe
Br
N
F3C
CF3
46
190 180 170 160 150 140 130 120 110 100 90 80 70 60 50 40 30 20 10 0
f1 (ppm)

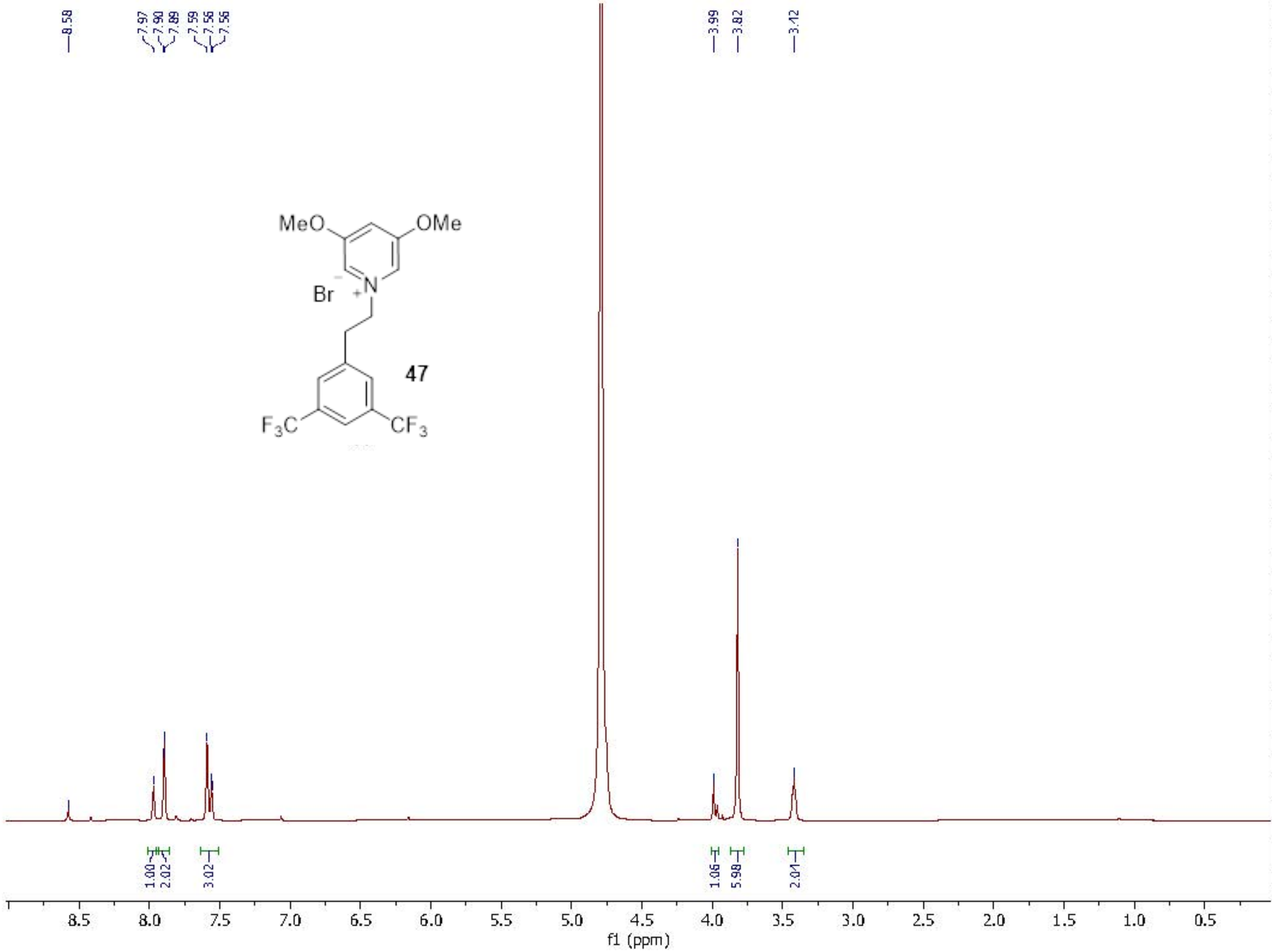
8.58
7.97
7.90
7.89
7.59
7.58
7.56
3.99
3.82
3.42
MeO
OMe
Br
N
47
F3C
CF3
1.00
2.02
3.02
1.06
5.98
2.01
8.5
8.0
7.5
7.0
6.5
6.0
5.5
5.0
4.5
4.0
3.5
3.0
2.5
2.0
1.5
1.0
0.5
f1 (ppm)

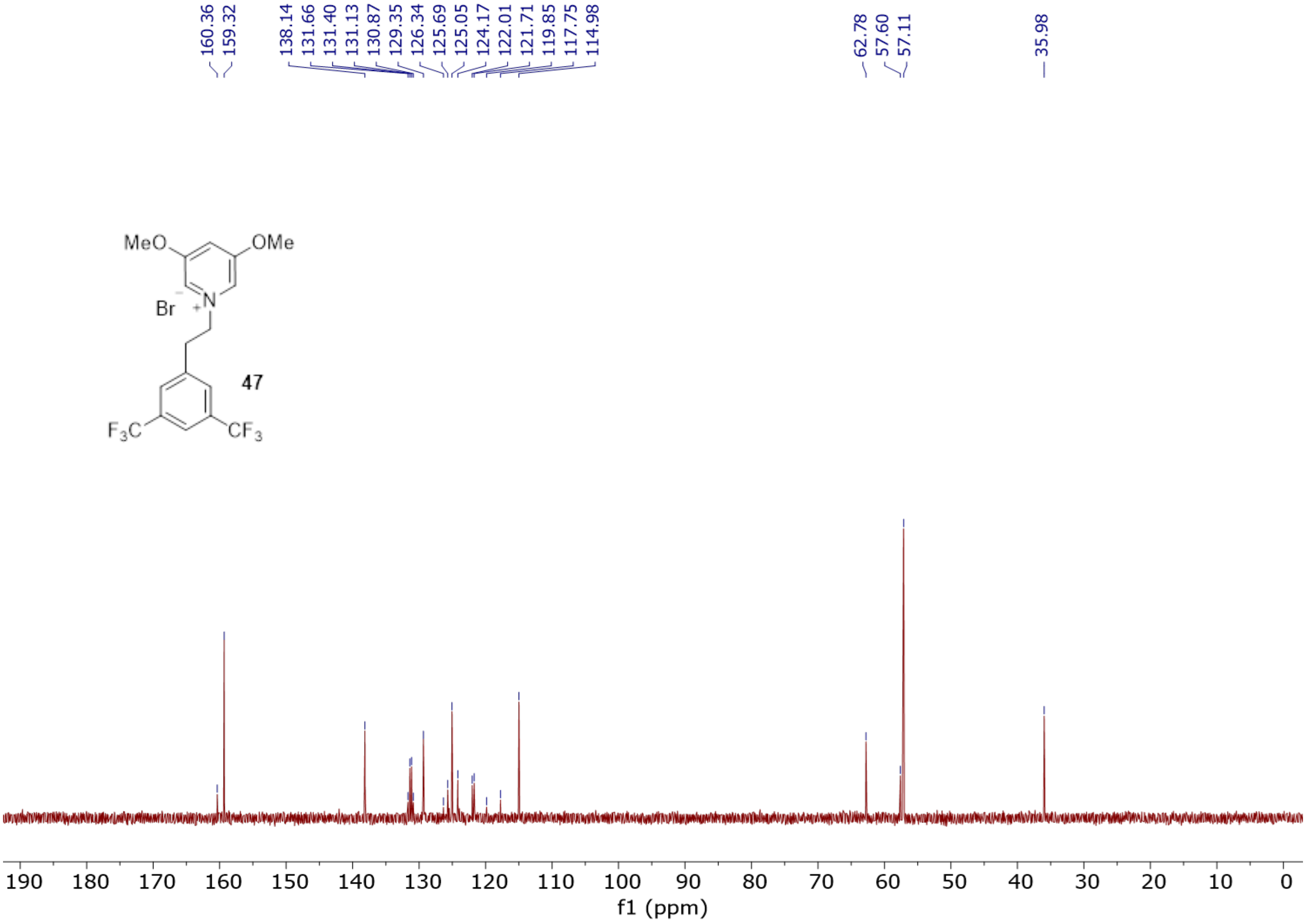
160.36
159.32
138.14
131.66
131.40
131.13
130.87
129.35
126.34
125.69
125.05
124.17
122.01
121.71
119.85
117.75
114.98
62.78
57.60
57.11
35.98
MeO
OMe
Br
N
F3C
CF3
190 180 170 160 150 140 130 120 110 100 90 80 70 60 50 40 30 20 10 0
f1 (ppm)

47

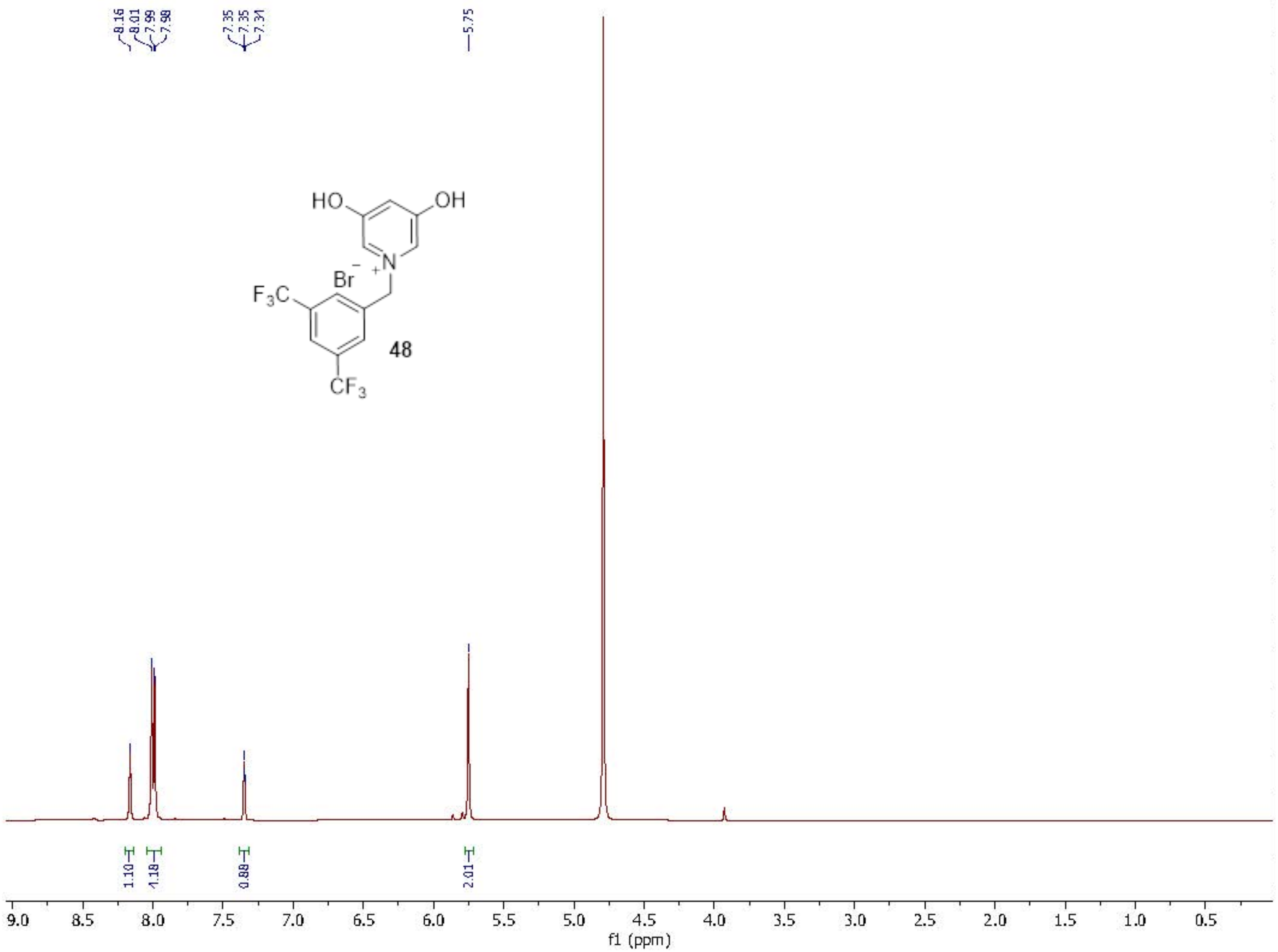
8.16
8.01
7.99
7.98
7.35
7.35
7.34
5.75
HO
OH
N
Br
F3C
CF3
48
1.10
1.18
0.88
2.01
9.0 8.5 8.0 7.5 7.0 6.5 6.0 5.5 5.0 4.5 4.0 3.5 3.0 2.5 2.0 1.5 1.0 0.5
f1 (ppm)

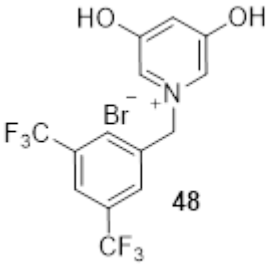
HO
OH
Br
N
F3C
CF3

48

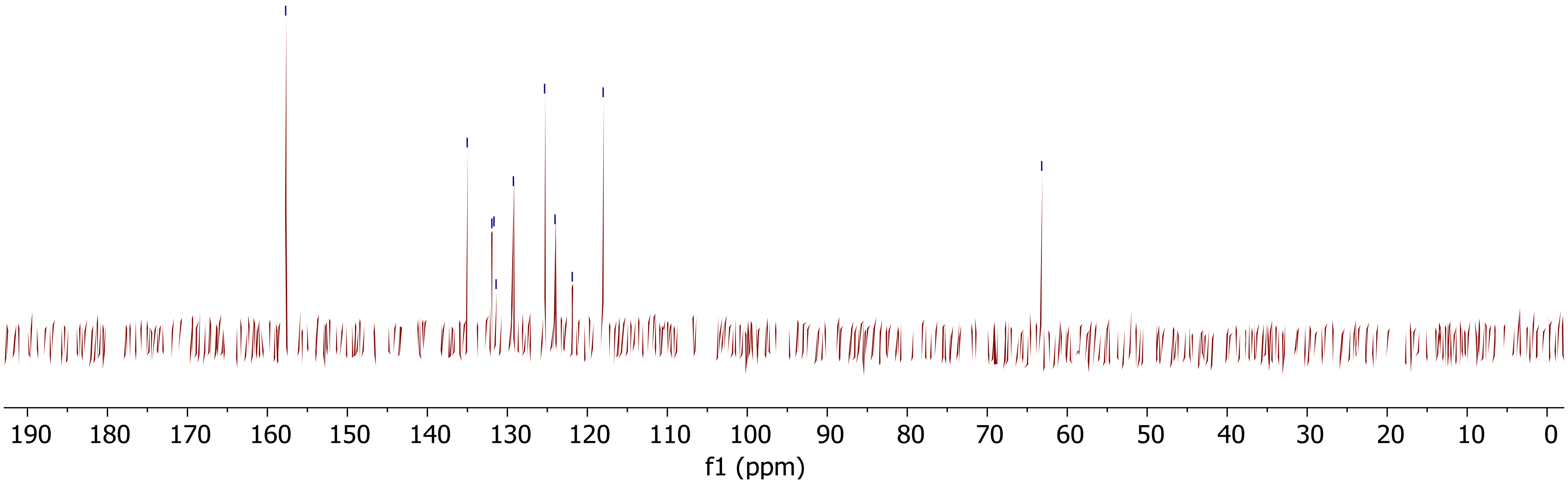
190 180 170 160 150 140 130 120 110 100 90 80 70 60 50 40 30 20 10 0
f1 (ppm)

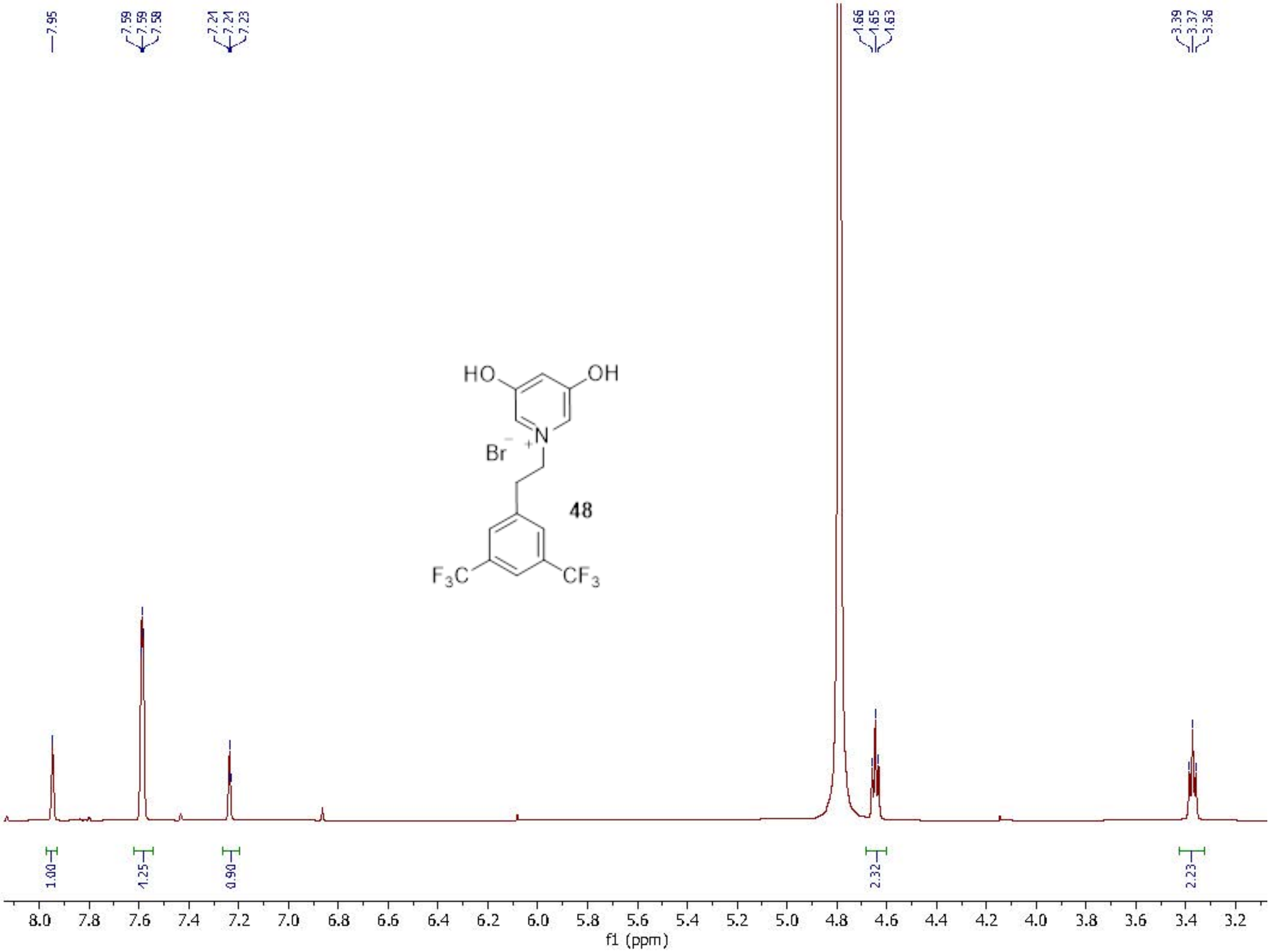

7.95
7.59
7.59
7.58
7.24
7.24
7.23
4.66
4.65
4.63
3.39
3.37
3.36
HO
OH
Br
N
48
F3C
CF3
1.00
1.25
0.90
2.32
2.23
8.0 7.8 7.6 7.4 7.2 7.0 6.8 6.6 6.4 6.2 6.0 5.8 5.6 5.4 5.2 5.0 4.8 4.6 4.4 4.2 4.0 3.8 3.6 3.4 3.2
f1 (ppm)

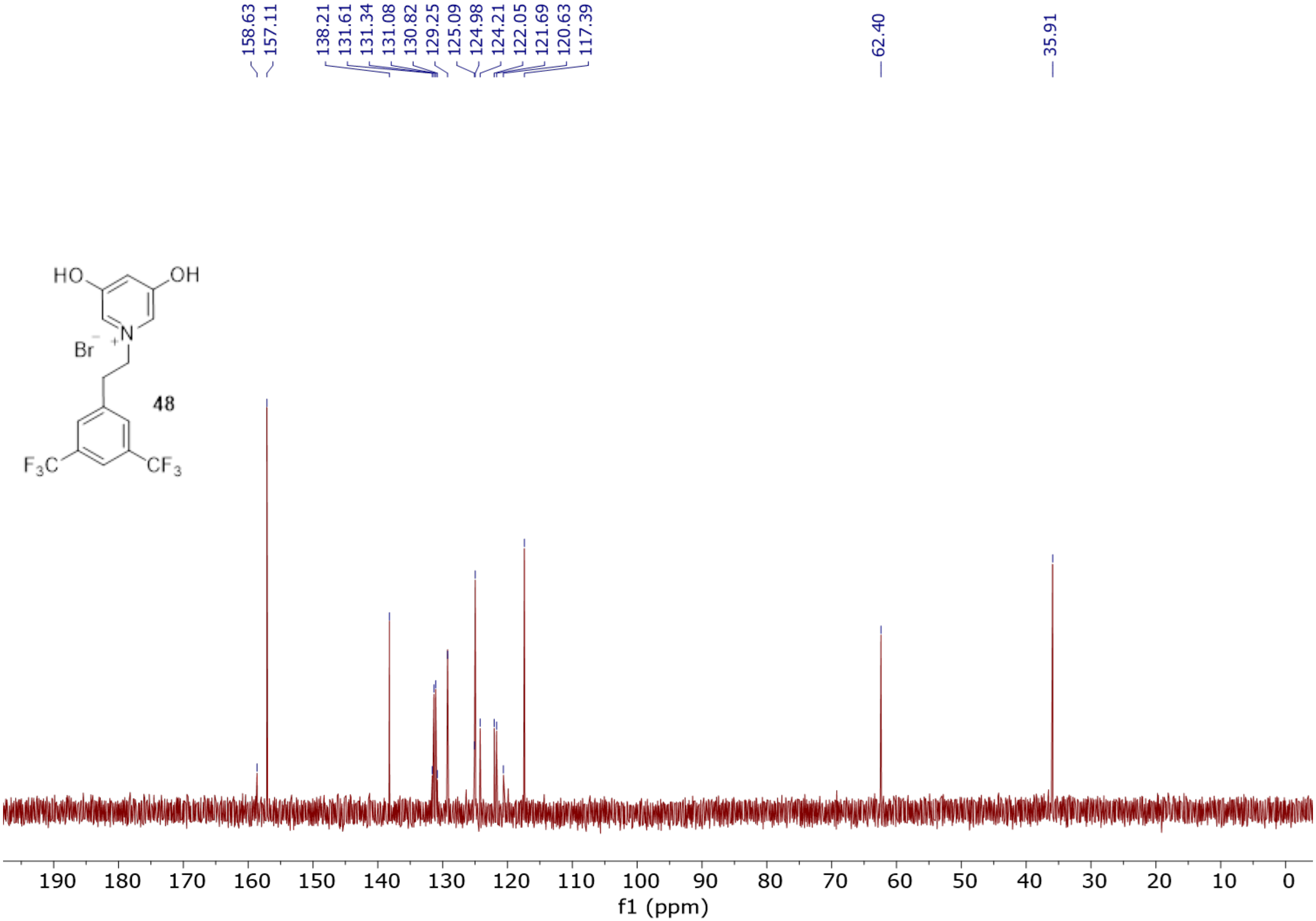
158.63
157.11
138.21
131.61
131.34
131.08
130.82
129.25
125.09
124.98
124.21
122.05
121.69
120.63
117.39
62.40
35.91
HO
OH
Br
N
48
F3C
CF3
190 180 170 160 150 140 130 120 110 100 90 80 70 60 50 40 30 20 10 0
f1 (ppm)

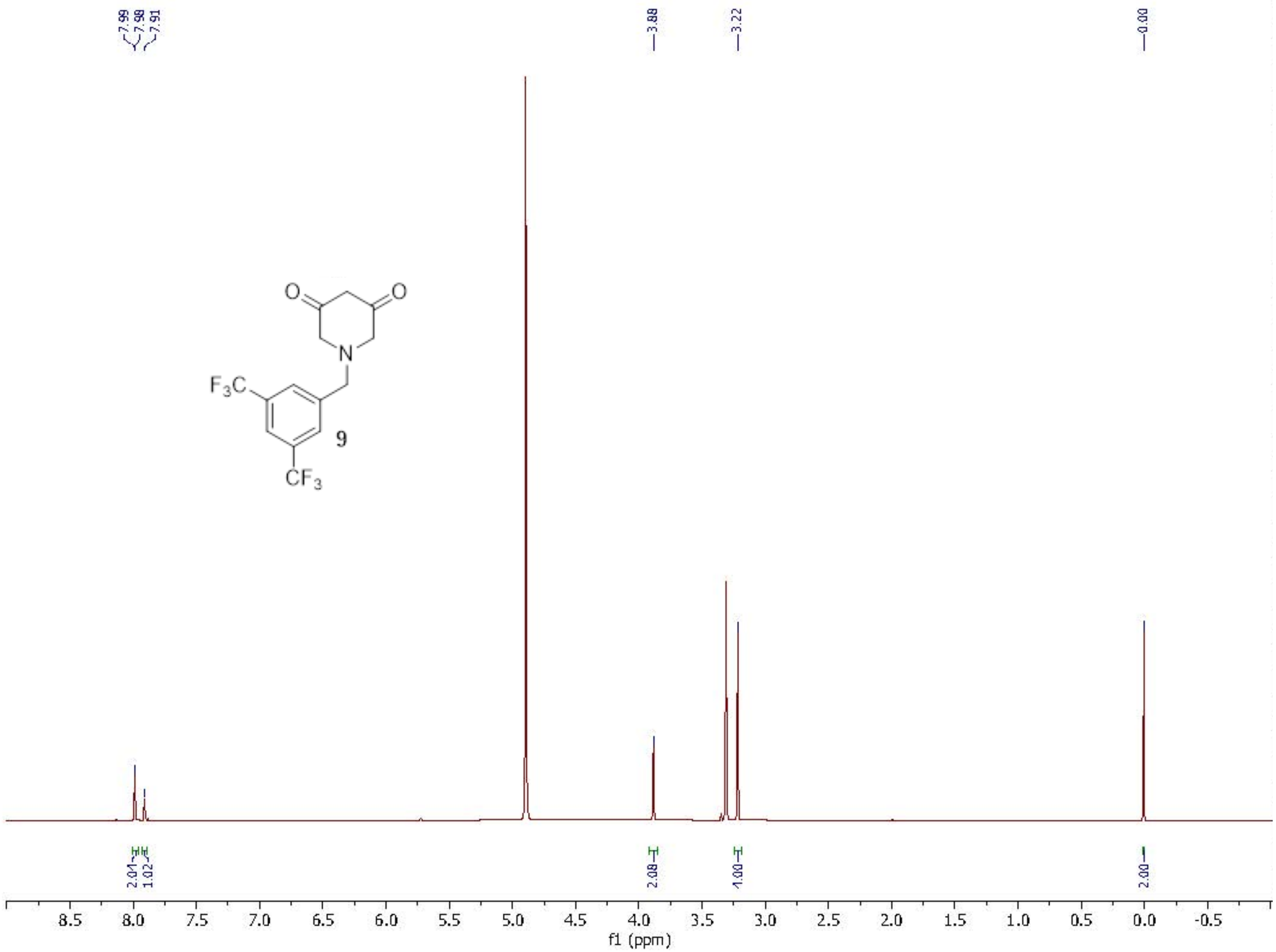

7.99
7.98
7.91
3.88
3.22
0.00
O
O
N
F_3C
CF_3
9
2.01
1.02
2.08
4.00
2.00
8.5 8.0 7.5 7.0 6.5 6.0 5.5 5.0 4.5 4.0 3.5 3.0 2.5 2.0 1.5 1.0 0.5 0.0 -0.5
f1 (ppm)

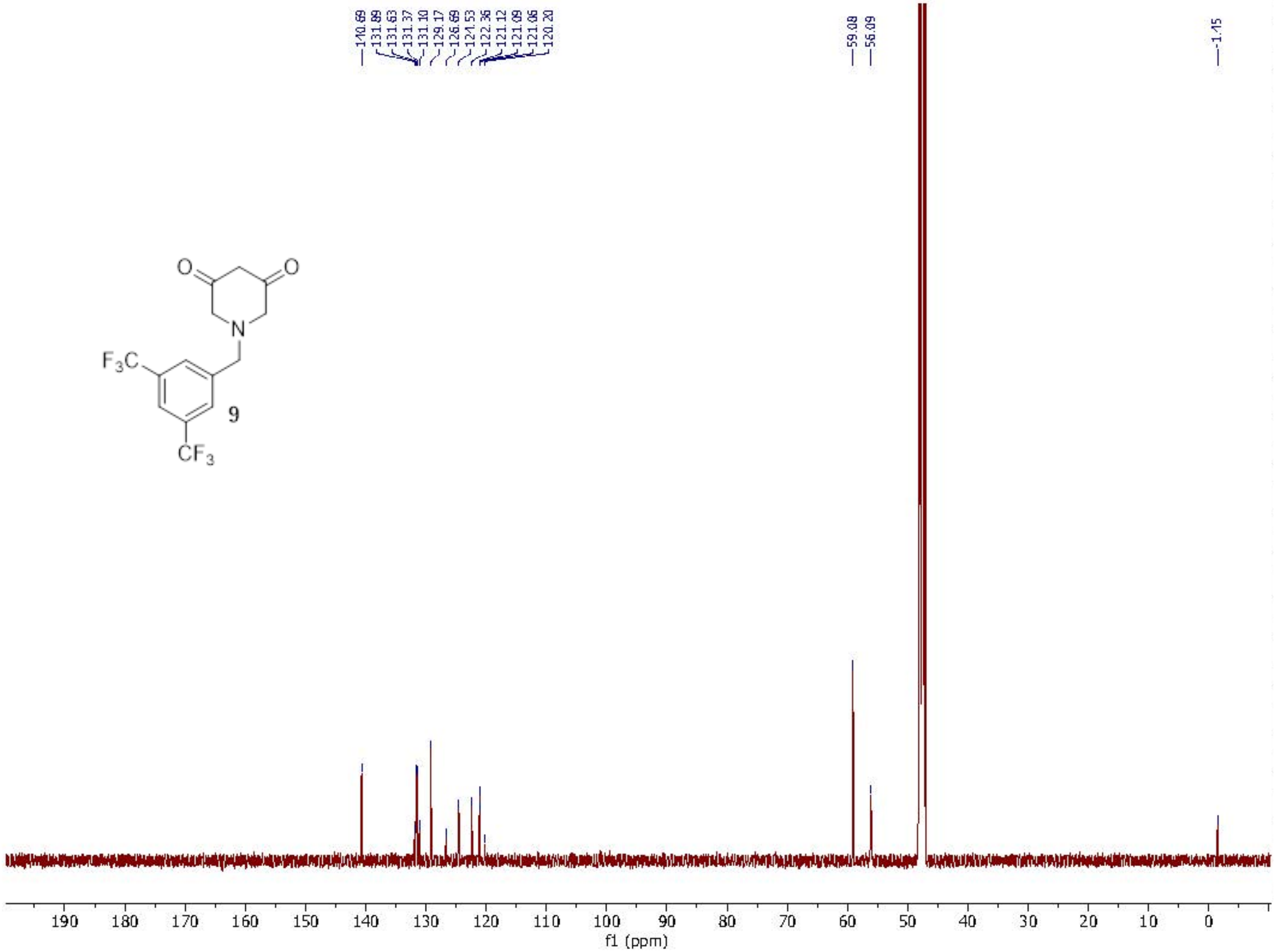
140.69
131.89
131.63
131.37
131.10
129.17
126.69
124.53
122.36
121.12
121.09
121.06
120.20
59.08
56.09
-1.15
F3C
CF3
N
O
9
190 180 170 160 150 140 130 120 110 100 90 80 70 60 50 40 30 20 10 0
f1 (ppm)

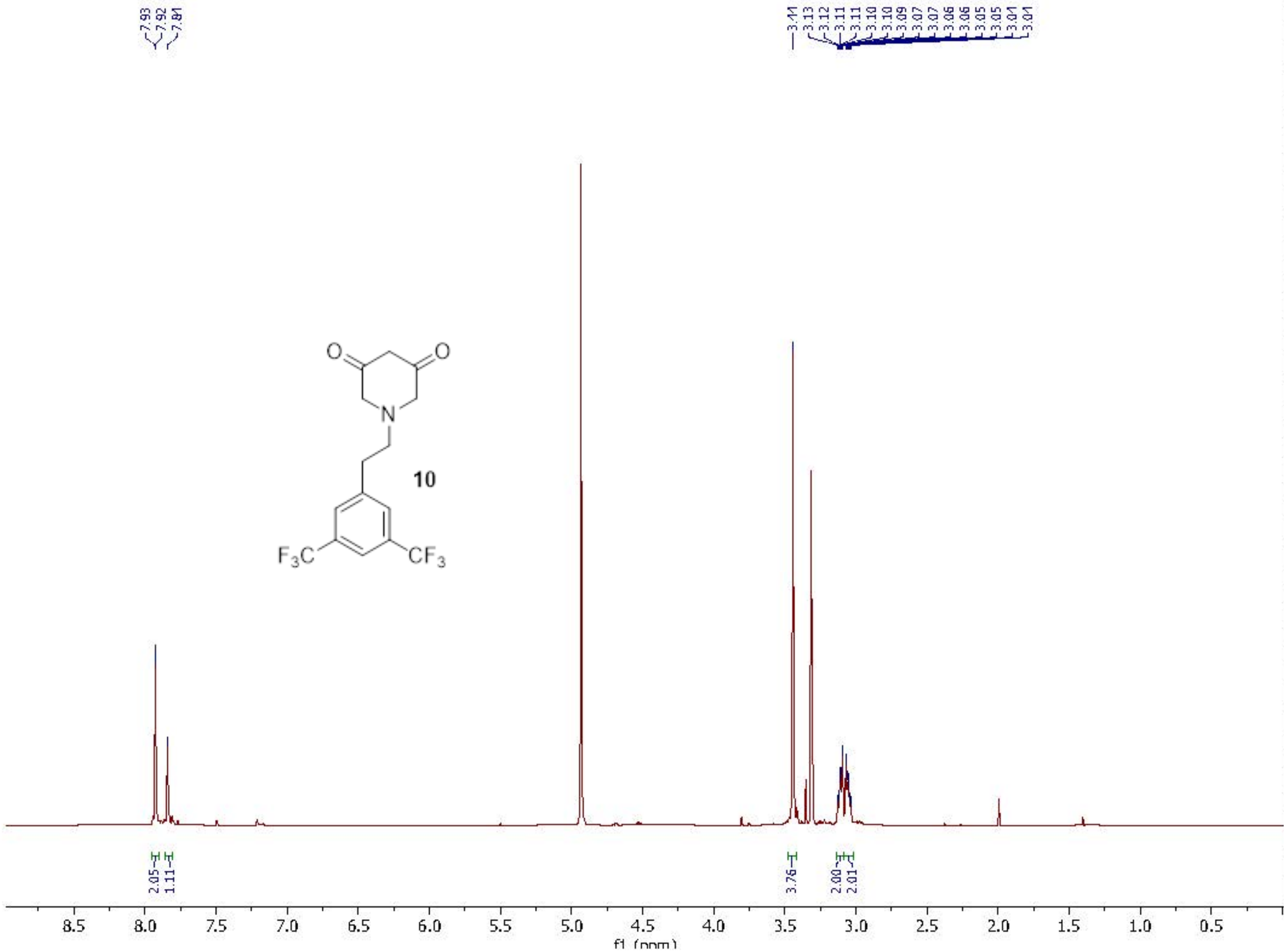
7.93
7.92
7.84
3.44
3.13
3.12
3.11
3.11
3.10
3.10
3.09
3.07
3.07
3.06
3.06
3.05
3.05
3.04
3.04
O
O
N
10
F3C
CF3
2.05
1.11
3.76
2.00
2.01
8.5
8.0
7.5
7.0
6.5
6.0
5.5
5.0
4.5
4.0
3.5
3.0
2.5
2.0
1.5
1.0
0.5
f1 (ppm)

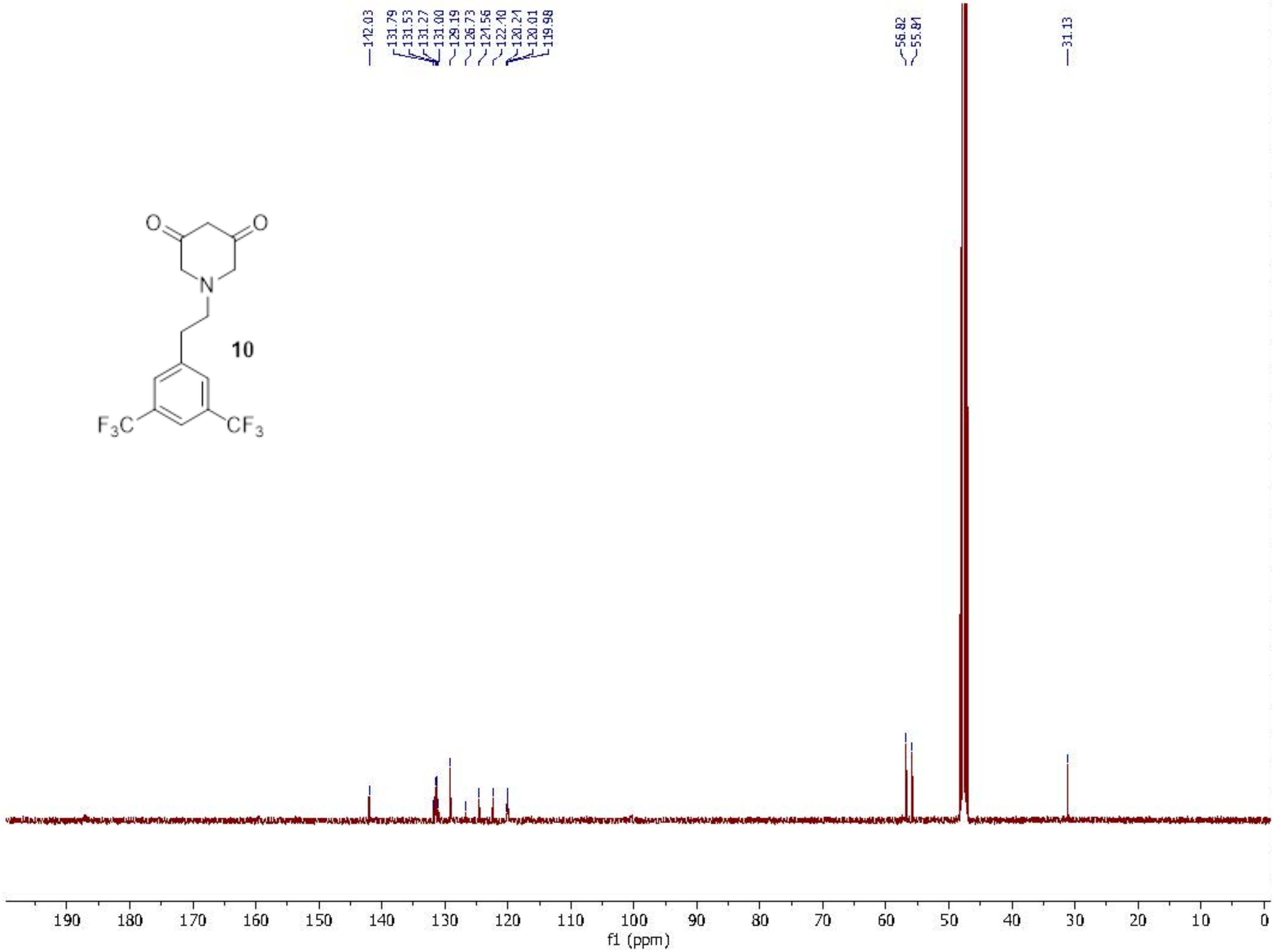
142.03
131.79
131.53
131.27
131.00
129.19
126.73
124.56
122.40
120.24
120.01
119.98
56.82
55.81
31.13
O
O
N
10
F_3C
CF_3
190 180 170 160 150 140 130 120 110 100 90 80 70 60 50 40 30 20 10 0
f1 (ppm)

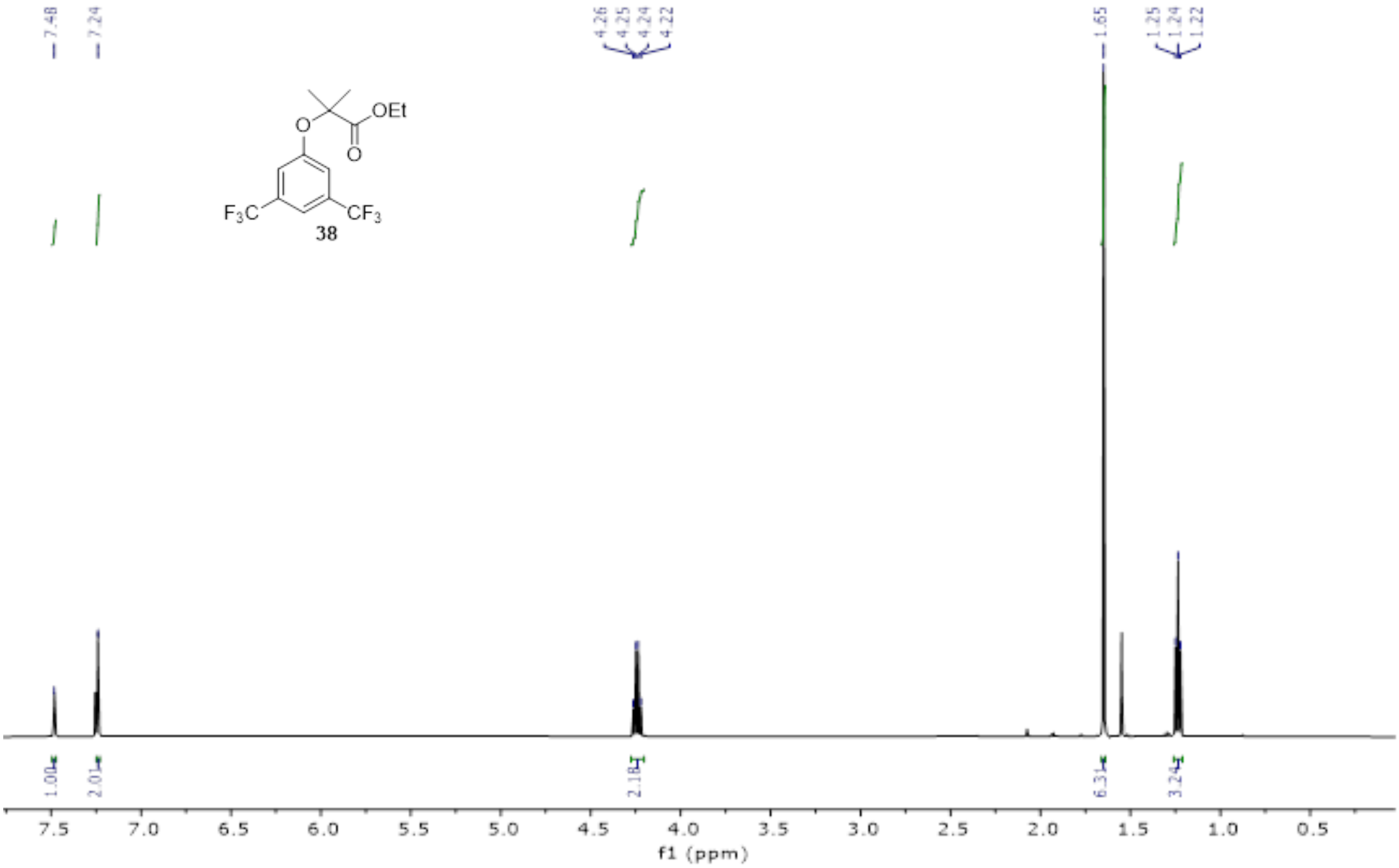
7.48
7.24
4.26
4.25
4.24
4.22
1.65
1.25
1.24
1.22
OEt
O
O
F3C
CF3
38
1.00
2.01
2.18
6.31
3.24
7.5
7.0
6.5
6.0
5.5
5.0
4.5
4.0
3.5
3.0
2.5
2.0
1.5
1.0
0.5
f1 (ppm)

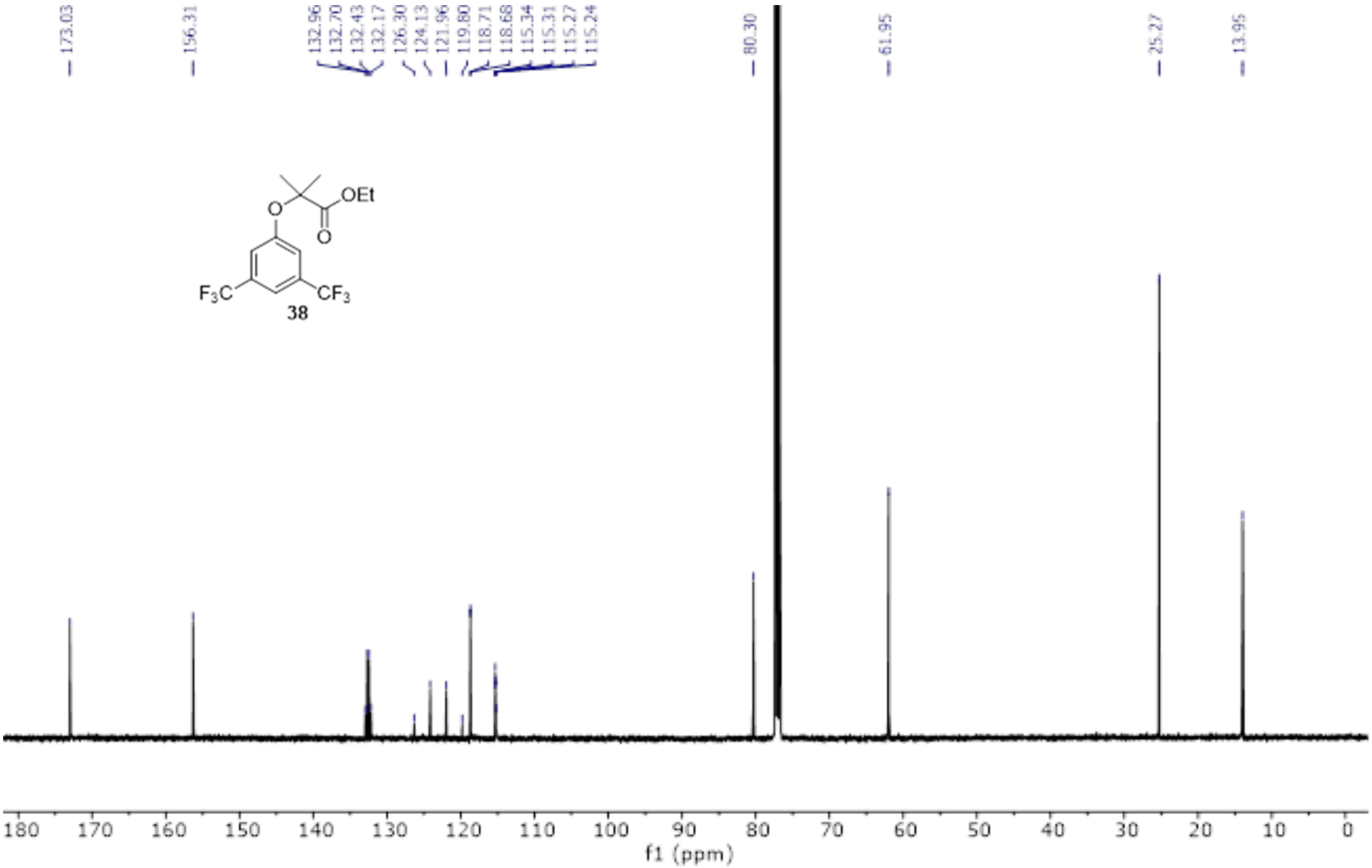
173.03
156.31
132.96
132.70
132.43
132.17
126.30
124.13
121.96
119.80
118.71
118.68
115.34
115.31
115.27
115.24
80.30
61.95
25.27
13.95
OEt
O
O
F3C
CF3
38
180
170
160
150
140
130
120
110
100
90
80
70
60
50
40
30
20
10
0
f1 (ppm)

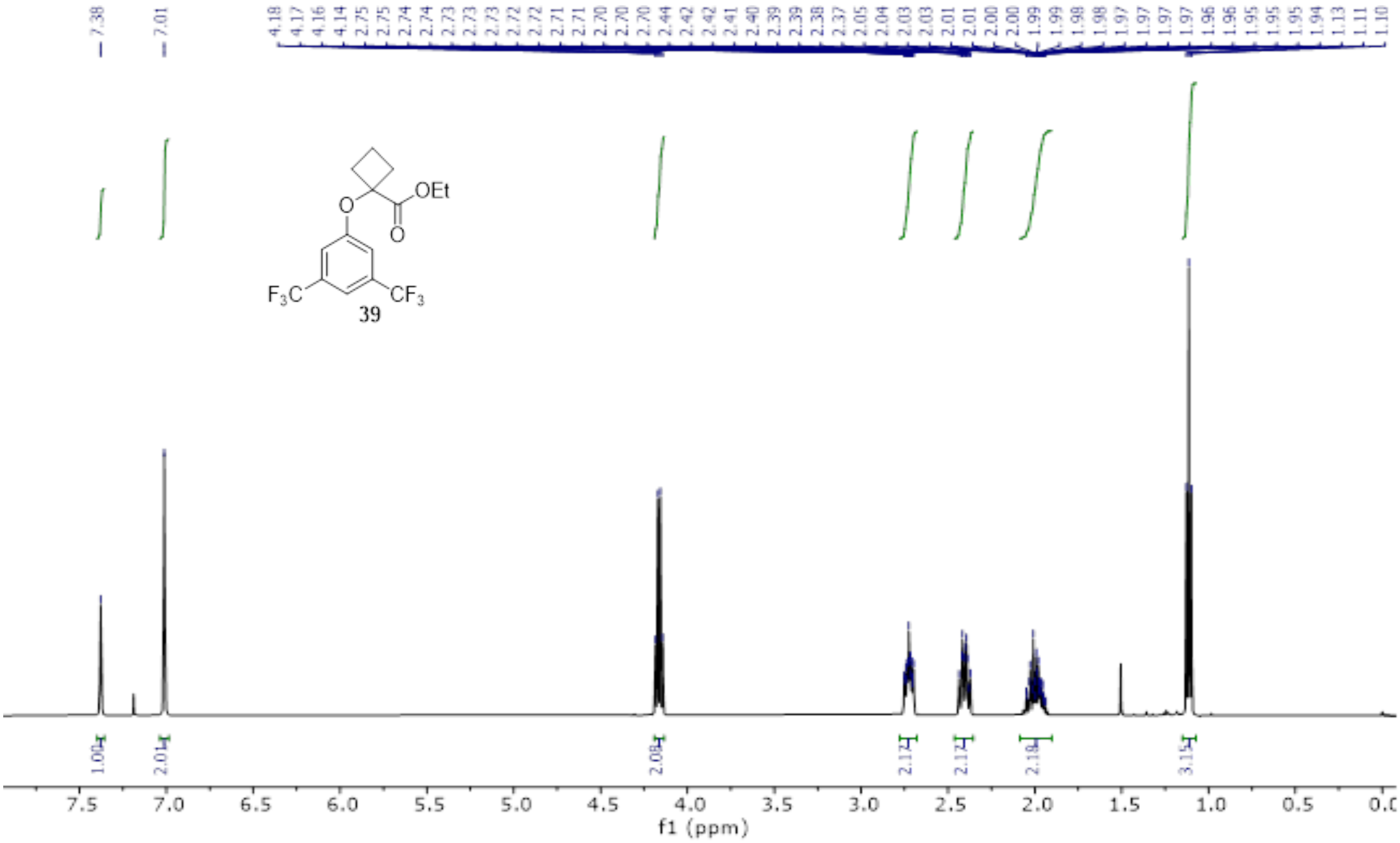
7.38
7.01
4.18
4.17
4.16
4.14
2.75
2.75
2.74
2.74
2.73
2.73
2.73
2.72
2.72
2.71
2.71
2.70
2.70
2.70
2.44
2.42
2.42
2.41
2.40
2.39
2.39
2.38
2.37
2.05
2.04
2.03
2.03
2.01
2.01
2.00
2.00
1.99
1.99
1.98
1.98
1.97
1.97
1.97
1.97
1.96
1.96
1.95
1.95
1.95
1.94
1.13
1.11
1.10
OEt
O
O
F3C
CF3
39
1.00
2.01
2.08
2.17
2.17
2.18
3.15
7.5
7.0
6.5
6.0
5.5
5.0
4.5
4.0
3.5
3.0
2.5
2.0
1.5
1.0
0.5
0.0
f1 (ppm)

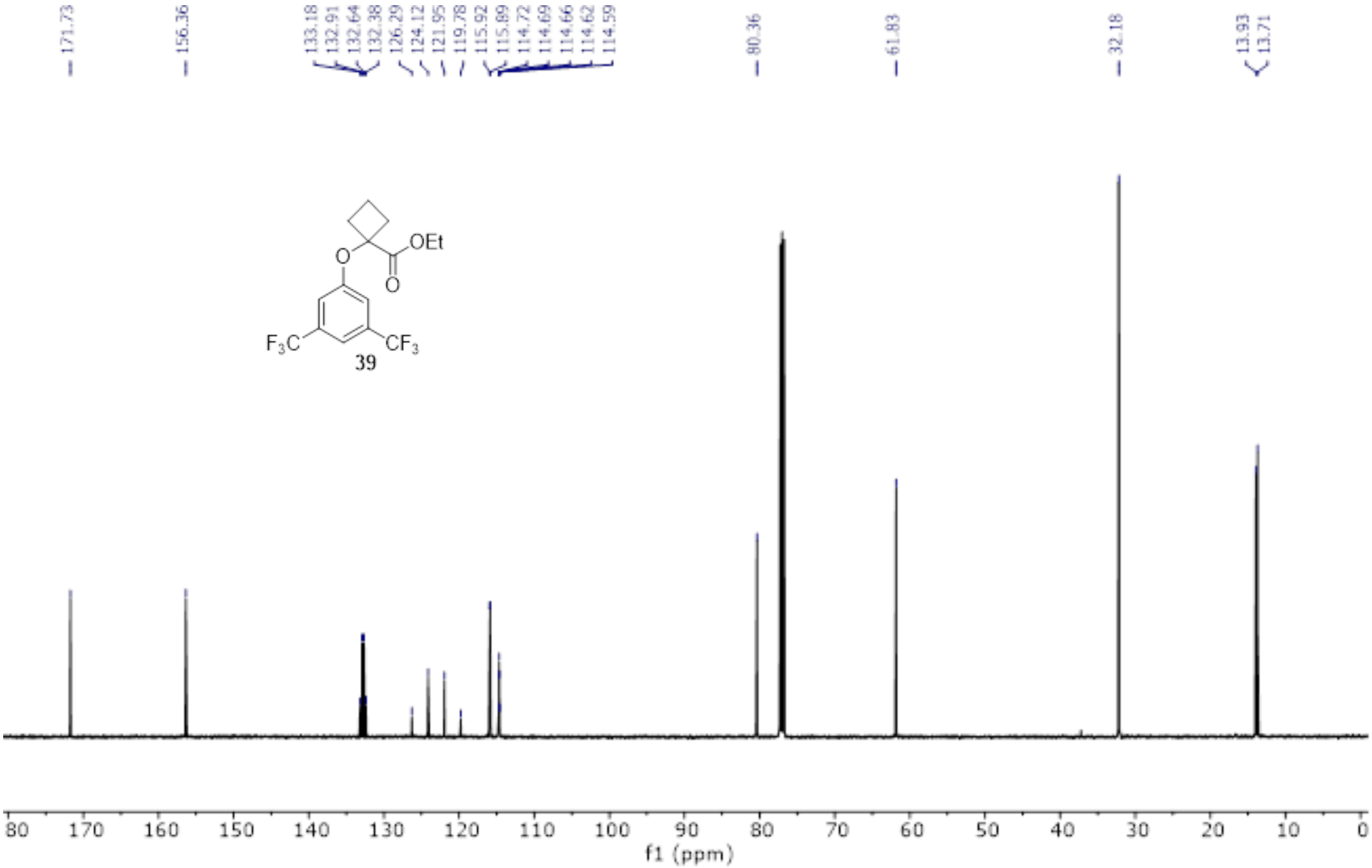
171.73
156.36
133.18
132.91
132.64
132.38
126.29
124.12
121.95
119.78
115.92
115.89
114.72
114.69
114.66
114.62
114.59
80.36
61.83
32.18
13.93
13.71
OEt
O
O
F3C
CF3
39
f1 (ppm)

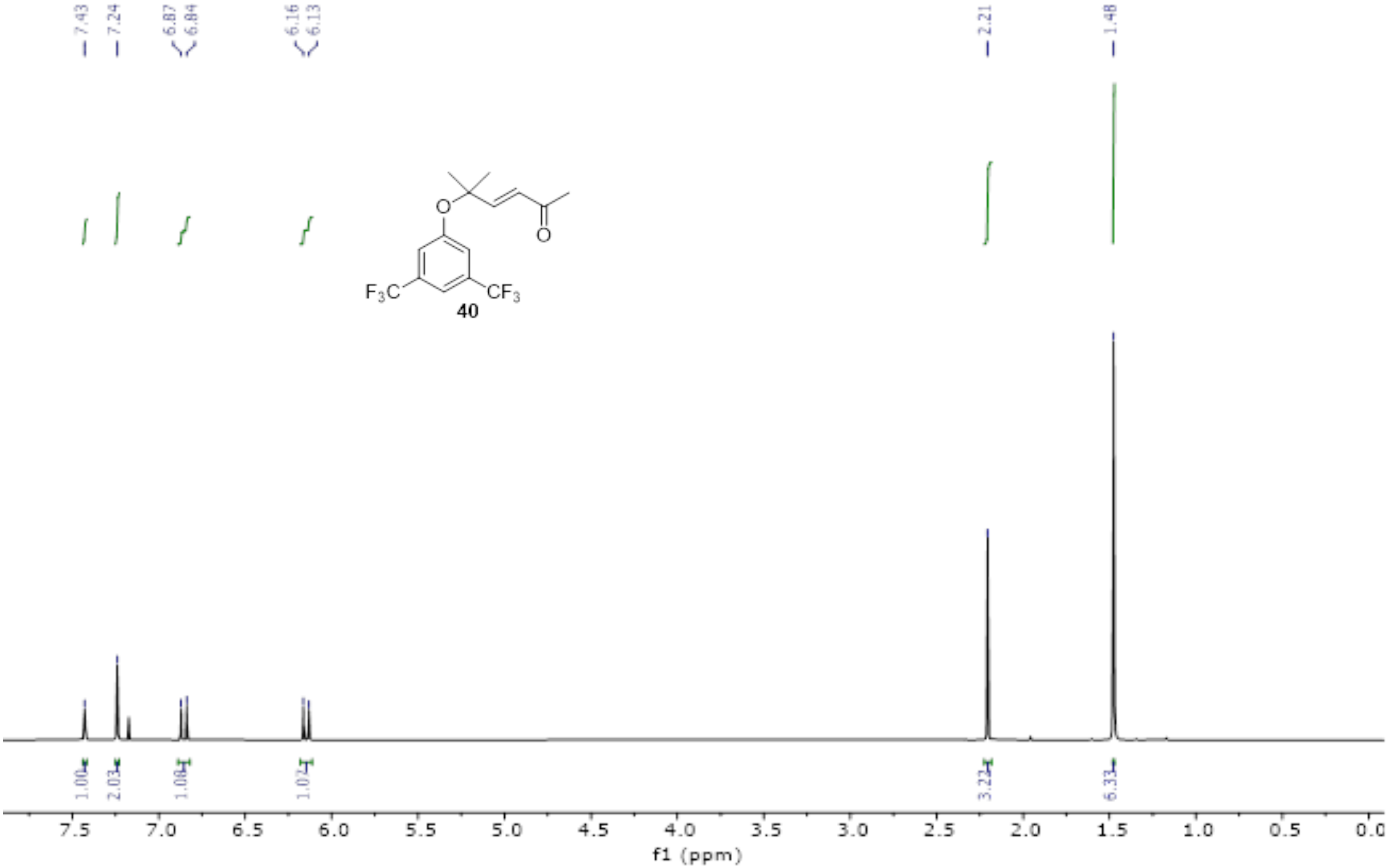
7.43
7.24
6.87
6.84
6.16
6.13
2.21
1.48
O
O
F3C
CF3
40
1.00
2.03
1.08
1.02
3.22
6.33
7.5 7.0 6.5 6.0 5.5 5.0 4.5 4.0 3.5 3.0 2.5 2.0 1.5 1.0 0.5 0.0
f1 (ppm)

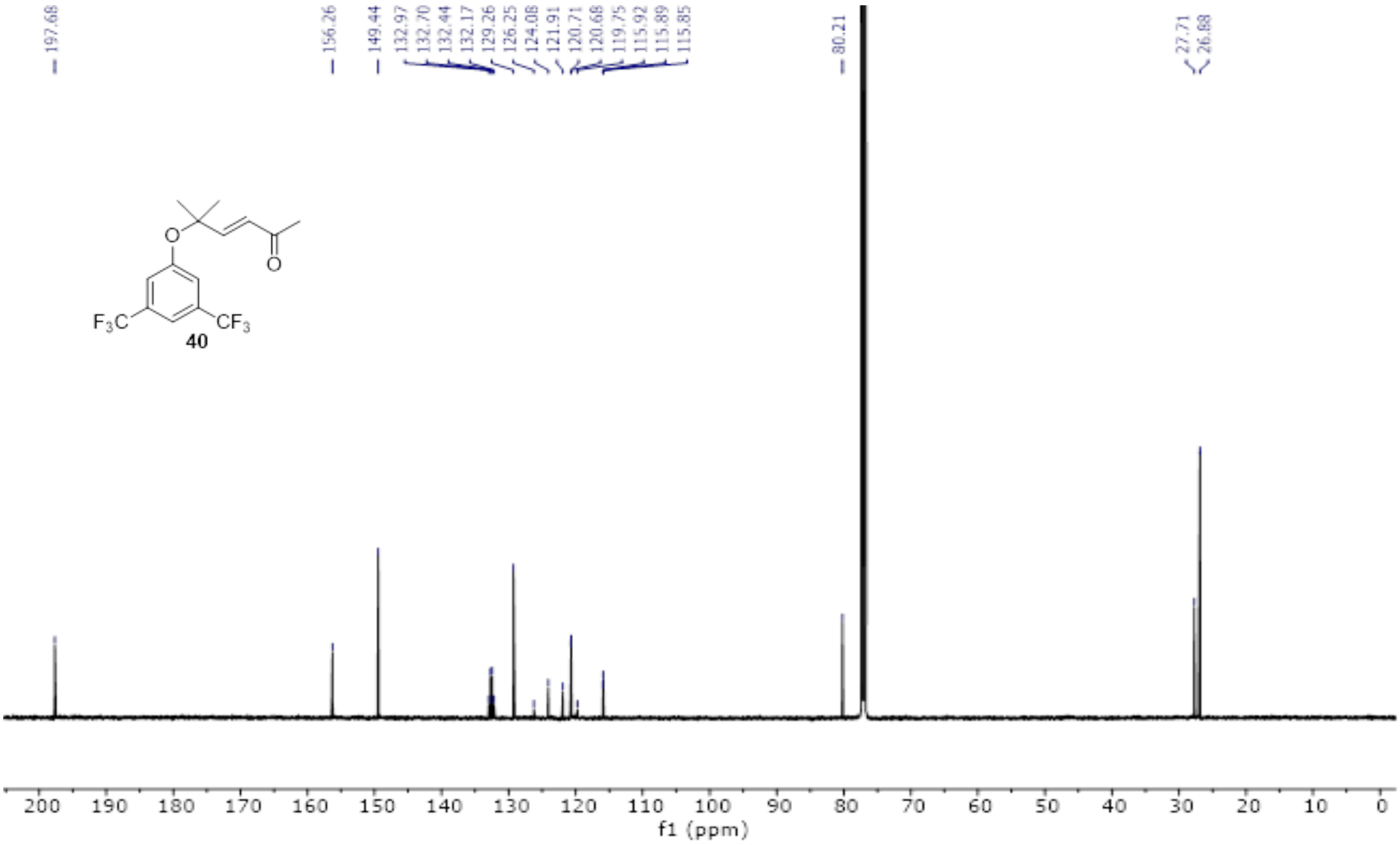

197.68
156.26
149.44
132.97
132.70
132.44
132.17
129.26
126.25
124.08
121.91
120.71
120.68
119.75
115.92
115.89
115.85
80.21
27.71
26.88
F3C
CF3
O
O
40
200 190 180 170 160 150 140 130 120 110 100 90 80 70 60 50 40 30 20 10 0
f1 (ppm)

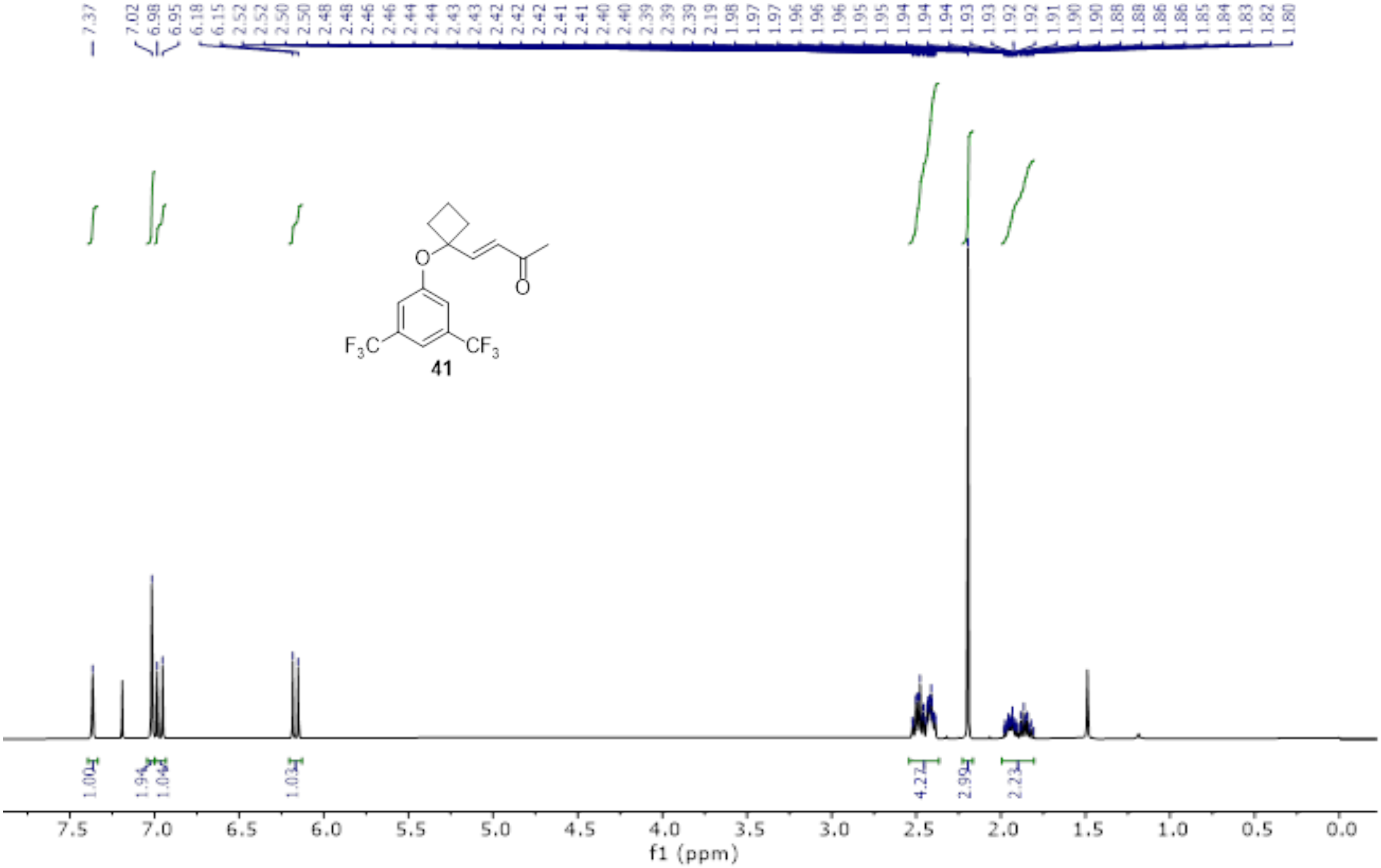
7.37
7.02
6.98
6.95
6.18
6.15
2.52
2.52
2.50
2.50
2.48
2.48
2.46
2.46
2.44
2.44
2.43
2.43
2.42
2.42
2.42
2.41
2.41
2.40
2.40
2.39
2.39
2.39
2.19
1.98
1.97
1.97
1.96
1.96
1.96
1.95
1.95
1.94
1.94
1.94
1.93
1.93
1.92
1.92
1.91
1.90
1.90
1.88
1.88
1.86
1.86
1.85
1.84
1.83
1.82
1.80
O
O
F_3C
CF_3
41
1.00
1.94
1.04
1.03
4.27
2.99
2.23
7.5
7.0
6.5
6.0
5.5
5.0
4.5
4.0
3.5
3.0
2.5
2.0
1.5
1.0
0.5
0.0
f1 (ppm)

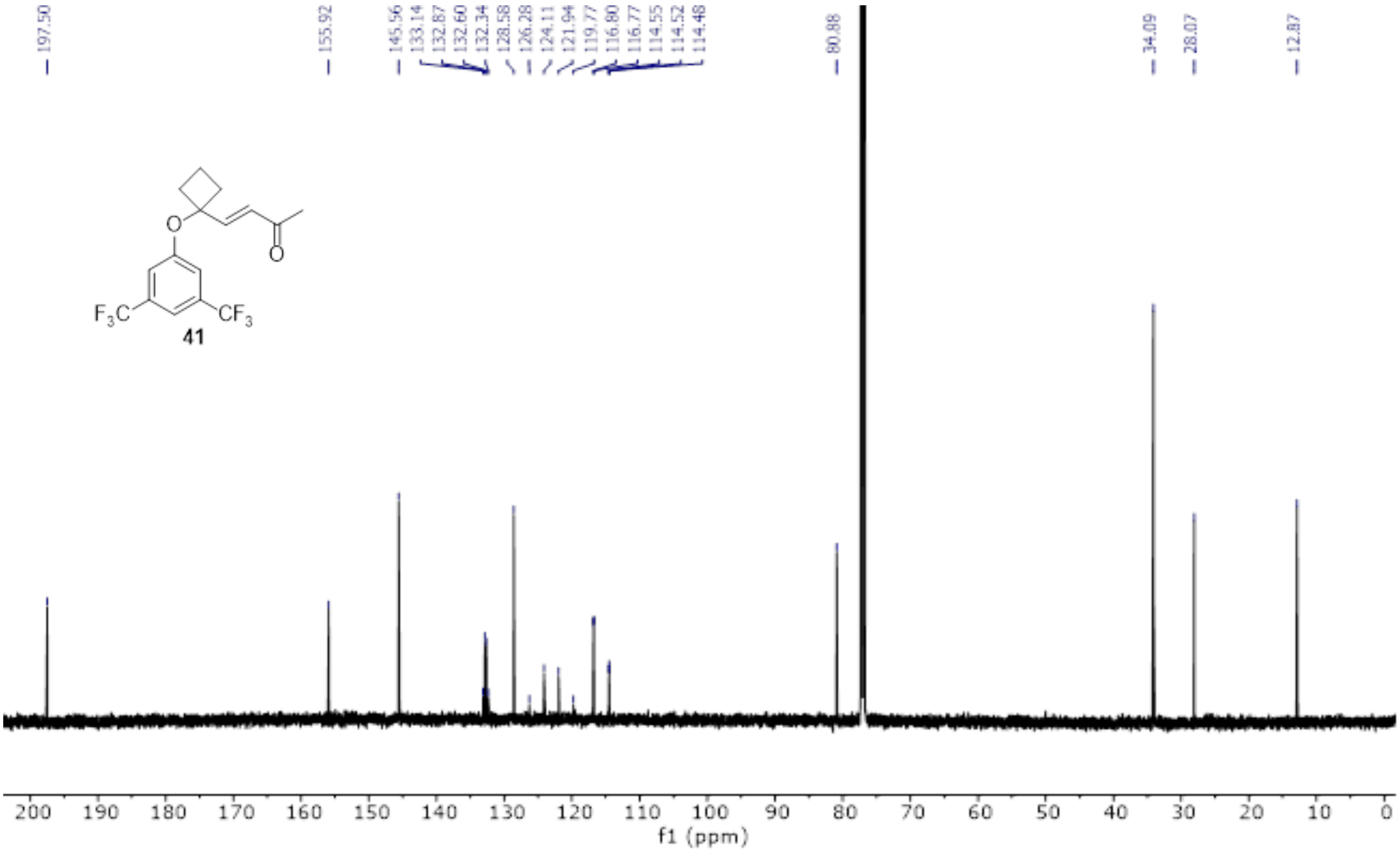
197.50
155.92
145.56
133.14
132.87
132.60
132.34
128.58
126.28
124.11
121.94
119.77
116.80
116.77
114.55
114.52
114.48
80.88
34.09
28.07
12.87
O
O
F3C
CF3
41
200
190
180
170
160
150
140
130
120
110
100
90
80
70
60
50
40
30
20
10
0
f1 (ppm)

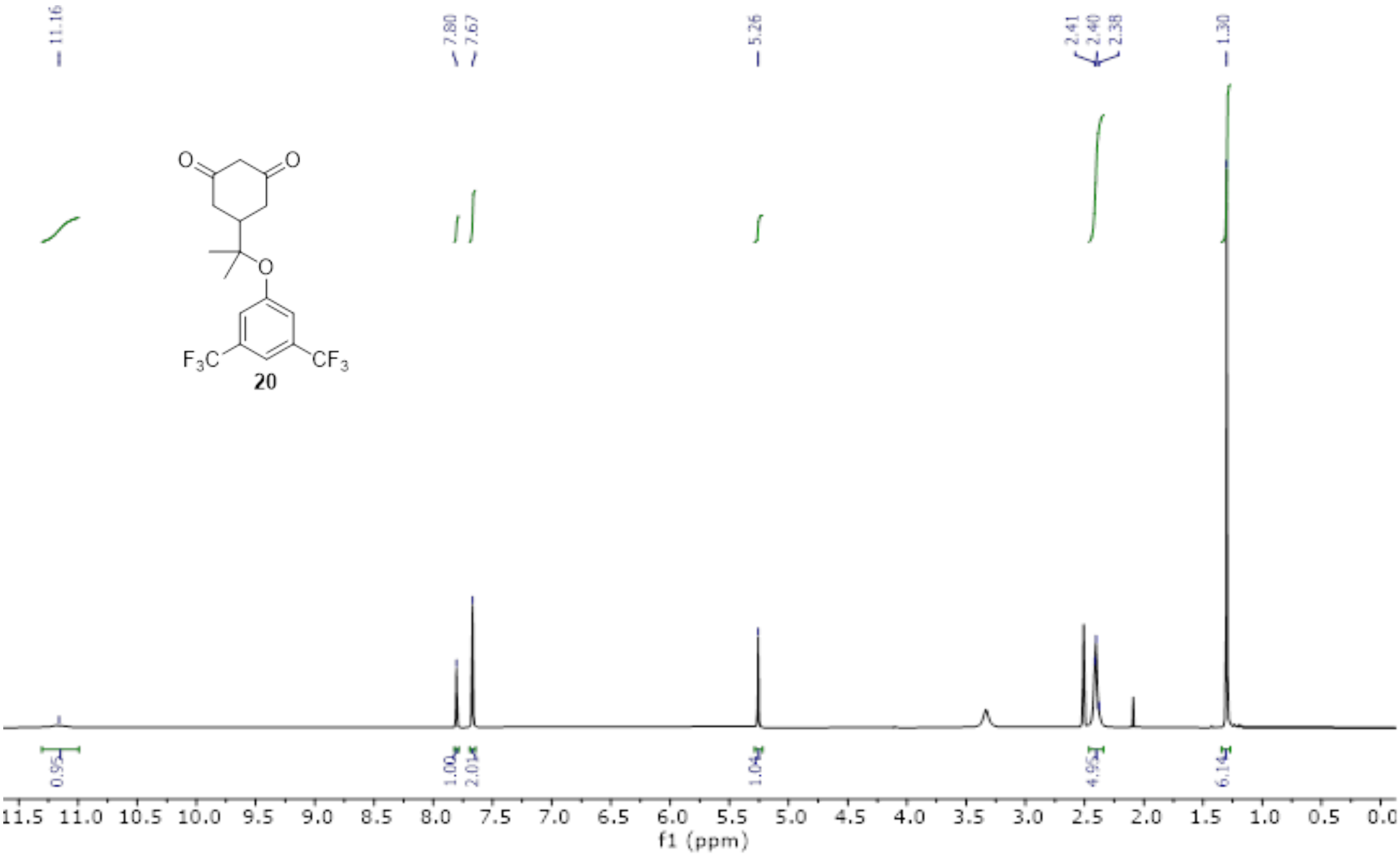

11.16
7.80
7.67
5.26
2.41
2.40
2.38
1.30
O
O
O
F3C
CF3
20
0.95
1.00
2.01
1.04
4.95
6.14
11.5 11.0 10.5 10.0 9.5 9.0 8.5 8.0 7.5 7.0 6.5 6.0 5.5 5.0 4.5 4.0 3.5 3.0 2.5 2.0 1.5 1.0 0.5 0.0
f1 (ppm)

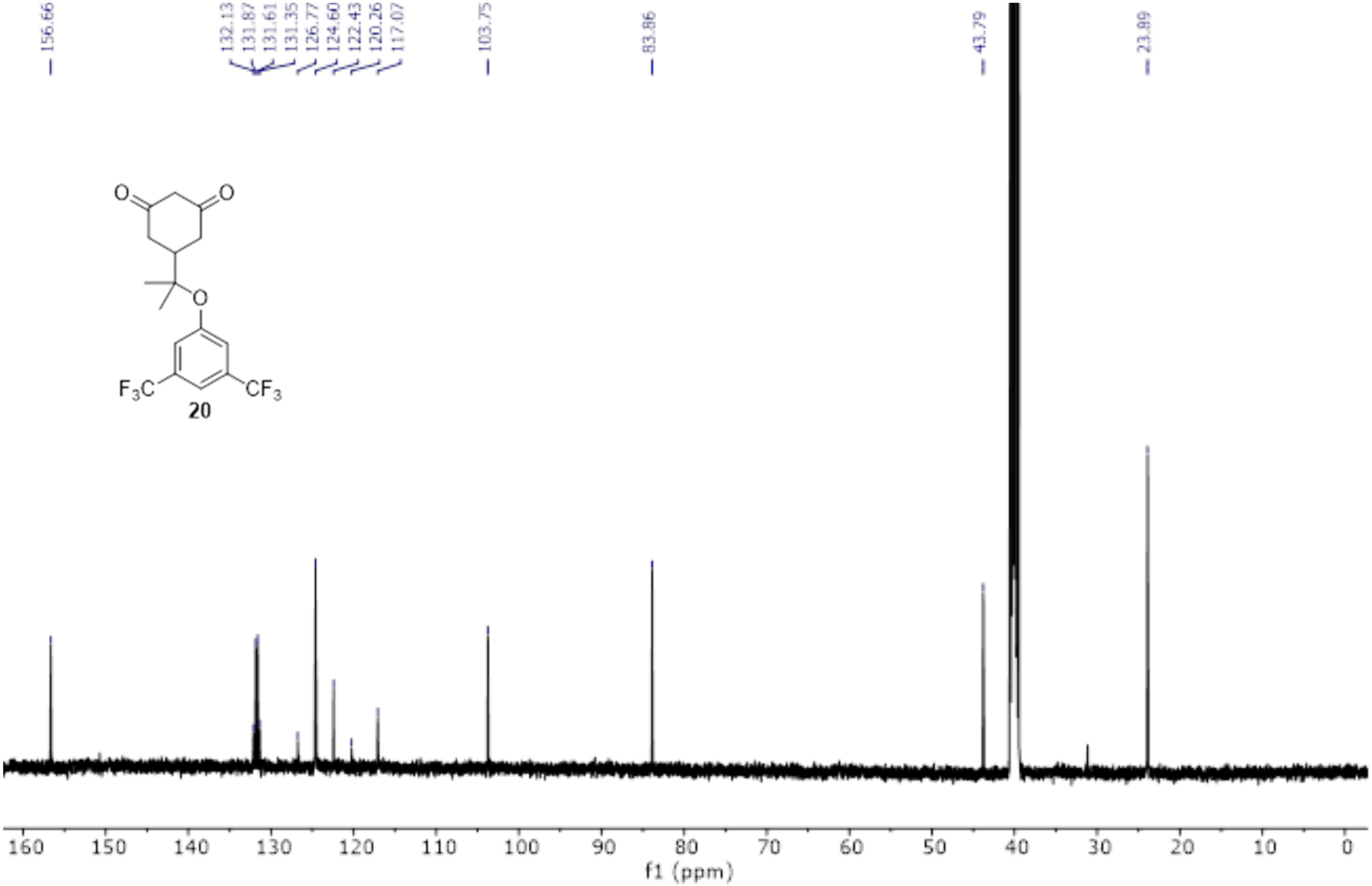
156.66
132.13
131.87
131.61
131.35
126.77
124.60
122.43
120.26
117.07
103.75
83.86
43.79
23.89
F3C
CF3
20
160 150 140 130 120 110 100 90 80 70 60 50 40 30 20 10 0
f1 (ppm)

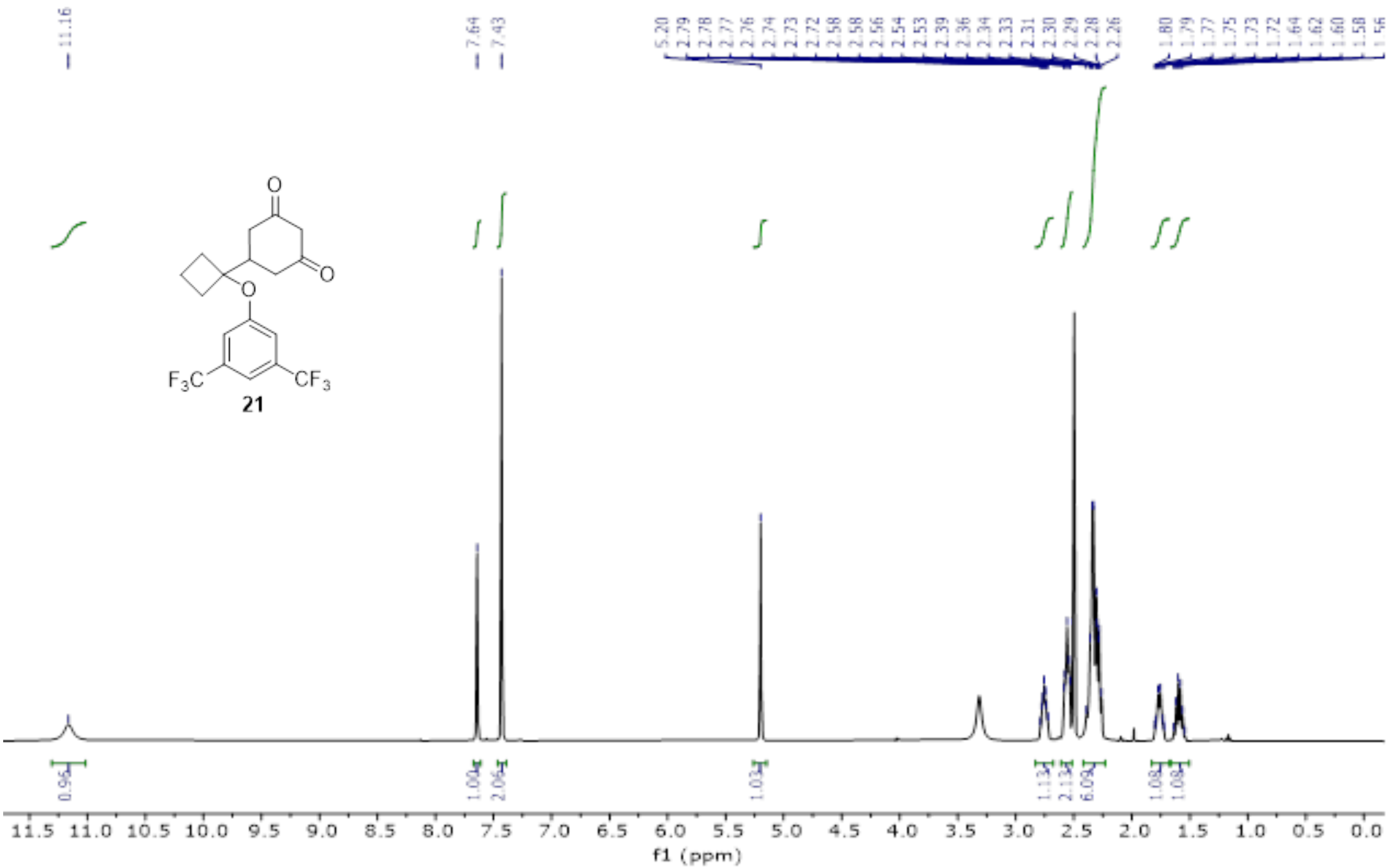

11.16
7.64
7.43
5.20
2.79
2.78
2.77
2.76
2.74
2.73
2.72
2.58
2.58
2.56
2.54
2.53
2.39
2.36
2.34
2.33
2.31
2.30
2.29
2.28
2.26
1.80
1.79
1.77
1.75
1.73
1.72
1.64
1.62
1.60
1.58
1.56
O
O
O
F3C
CF3
21
0.96
1.00
2.06
1.03
1.13
2.13
6.09
1.08
1.08
11.5 11.0 10.5 10.0 9.5 9.0 8.5 8.0 7.5 7.0 6.5 6.0 5.5 5.0 4.5 4.0 3.5 3.0 2.5 2.0 1.5 1.0 0.5 0.0
f1 (ppm)

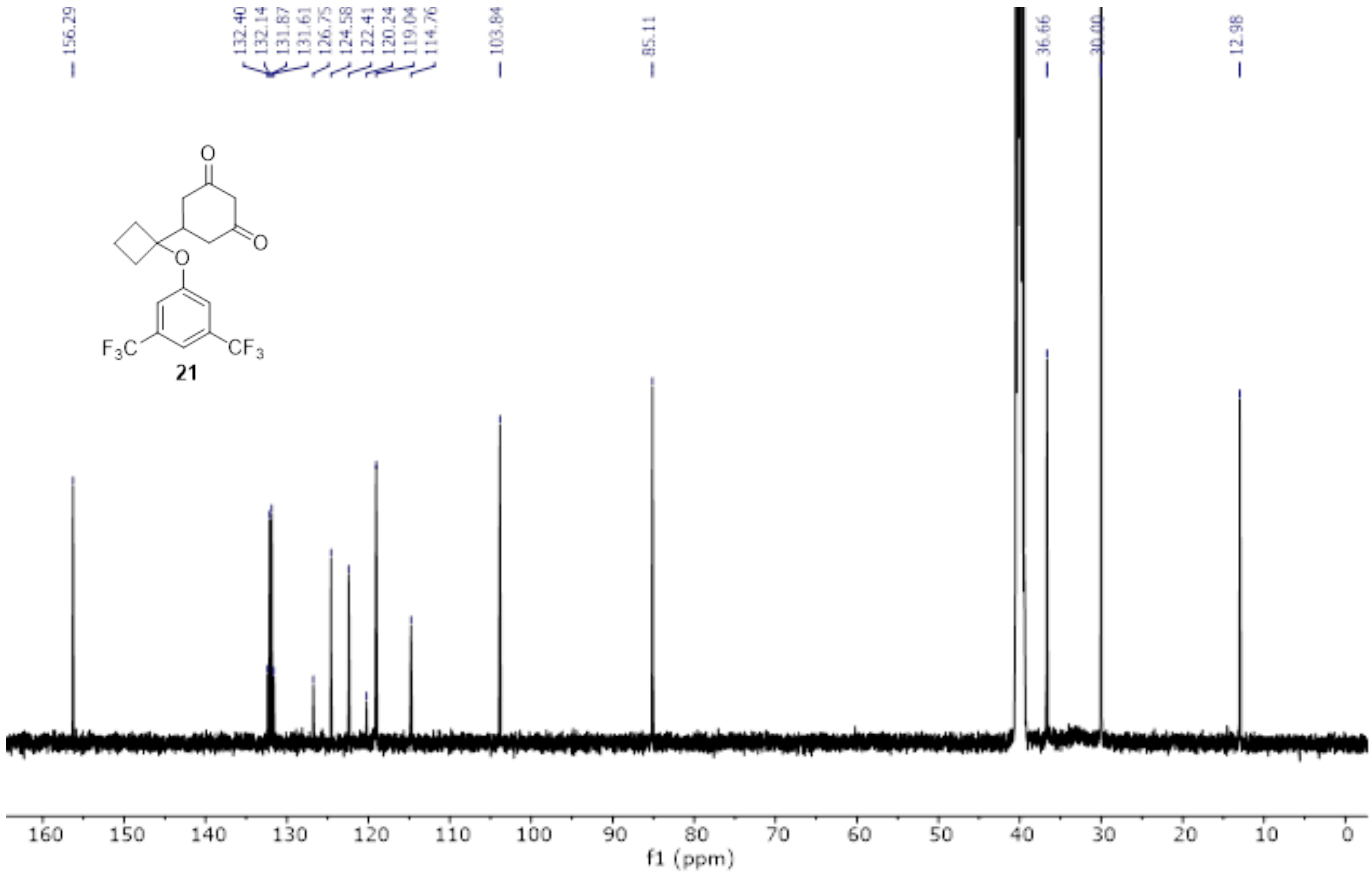
156.29
132.40
132.14
131.87
131.61
126.75
124.58
122.41
120.24
119.04
114.76
103.84
85.11
36.66
30.00
12.98
O
O
O
F_3C
CF_3
21
160 150 140 130 120 110 100 90 80 70 60 50 40 30 20 10 0
f1 (ppm)

2. HRMS spectra

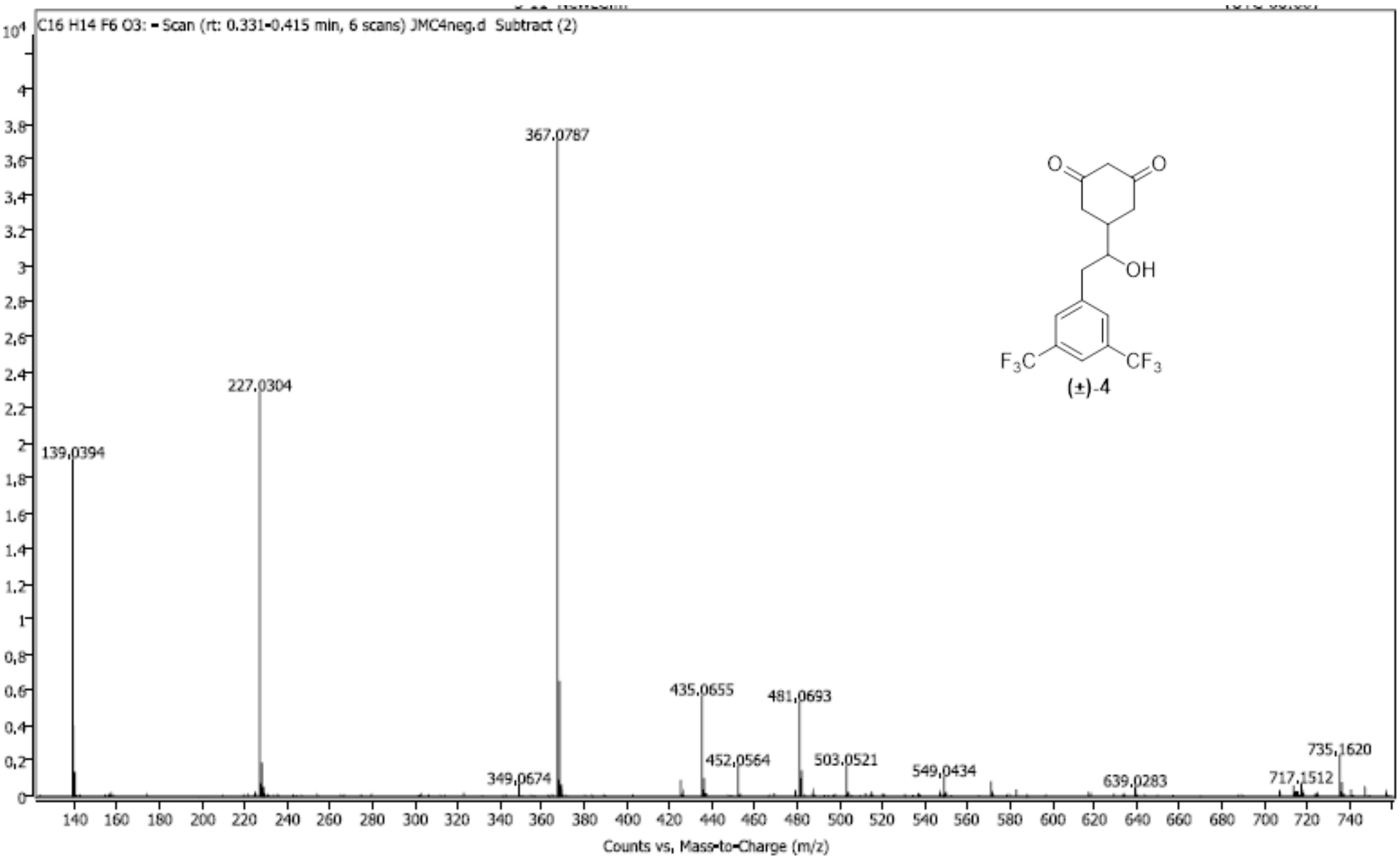

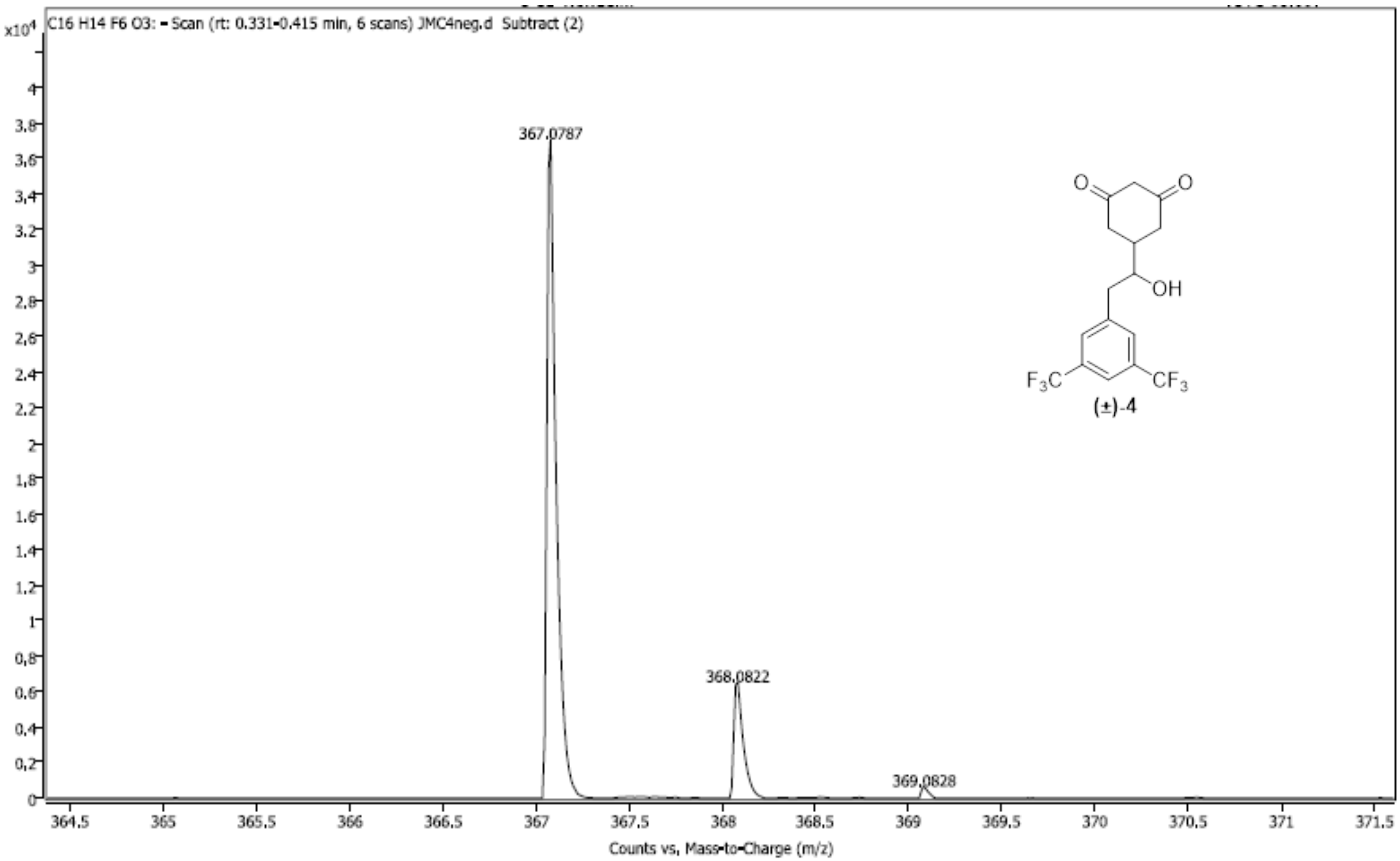

x10^4
C16 H14 F6 O3: - Scan (rt: 0.331-0.415 min, 6 scans) JMC4neg.d Subtract (2)
367.0787
368.0822
369.0828
F3C
CF3
OH
O
(±)-4
Counts vs. Mass-to-Charge (m/z)

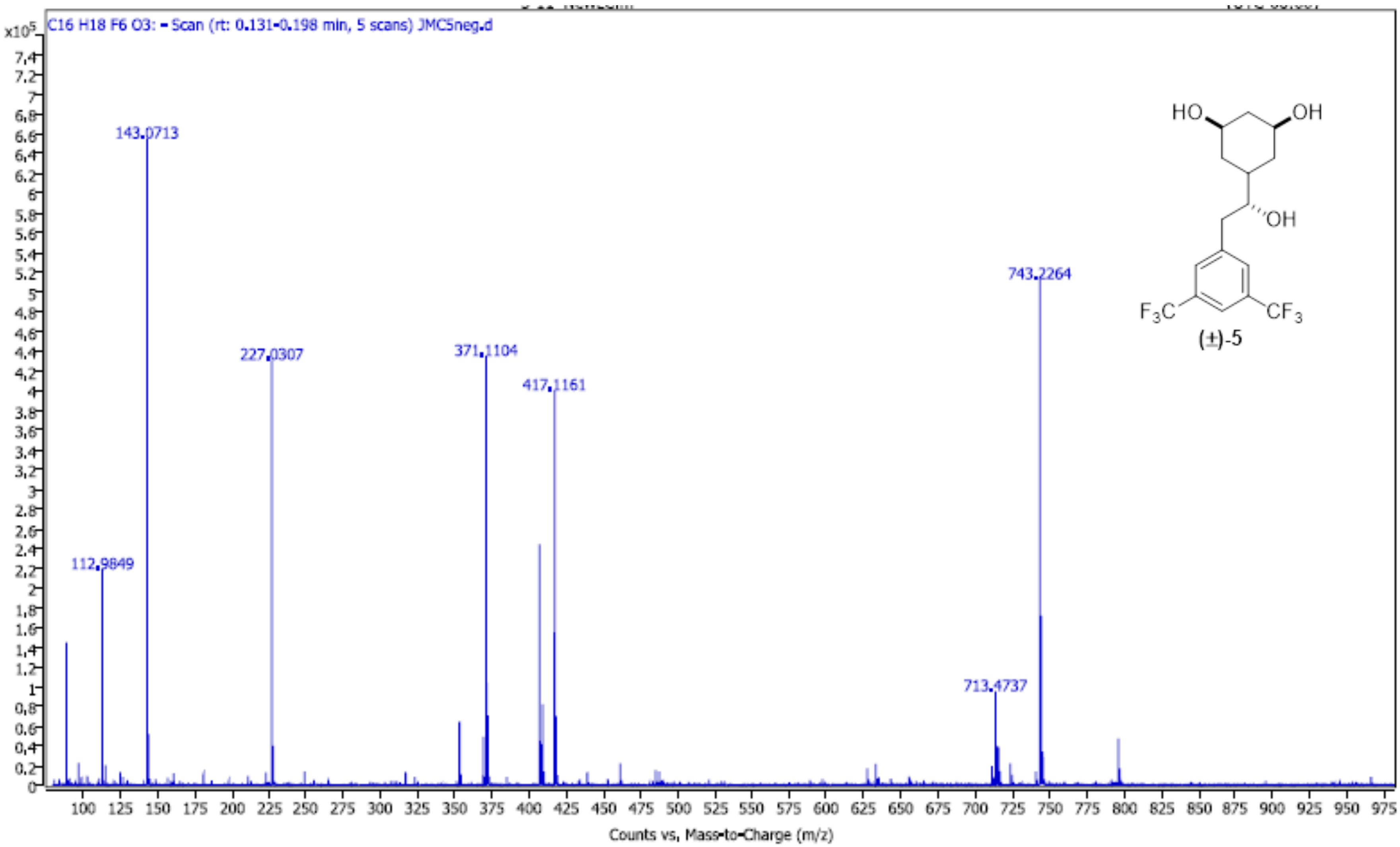

C16 H18 F6 O3: - Scan (rt: 0.131-0.198 min, 5 scans) JMC5neg.d
x10^5
143.0713
743.2264
227.0307
371.1104
417.1161
112.9849
713.4737
Counts vs. Mass-to-Charge (m/z)
HO
OH
OH
F3C
CF3
(±)-5

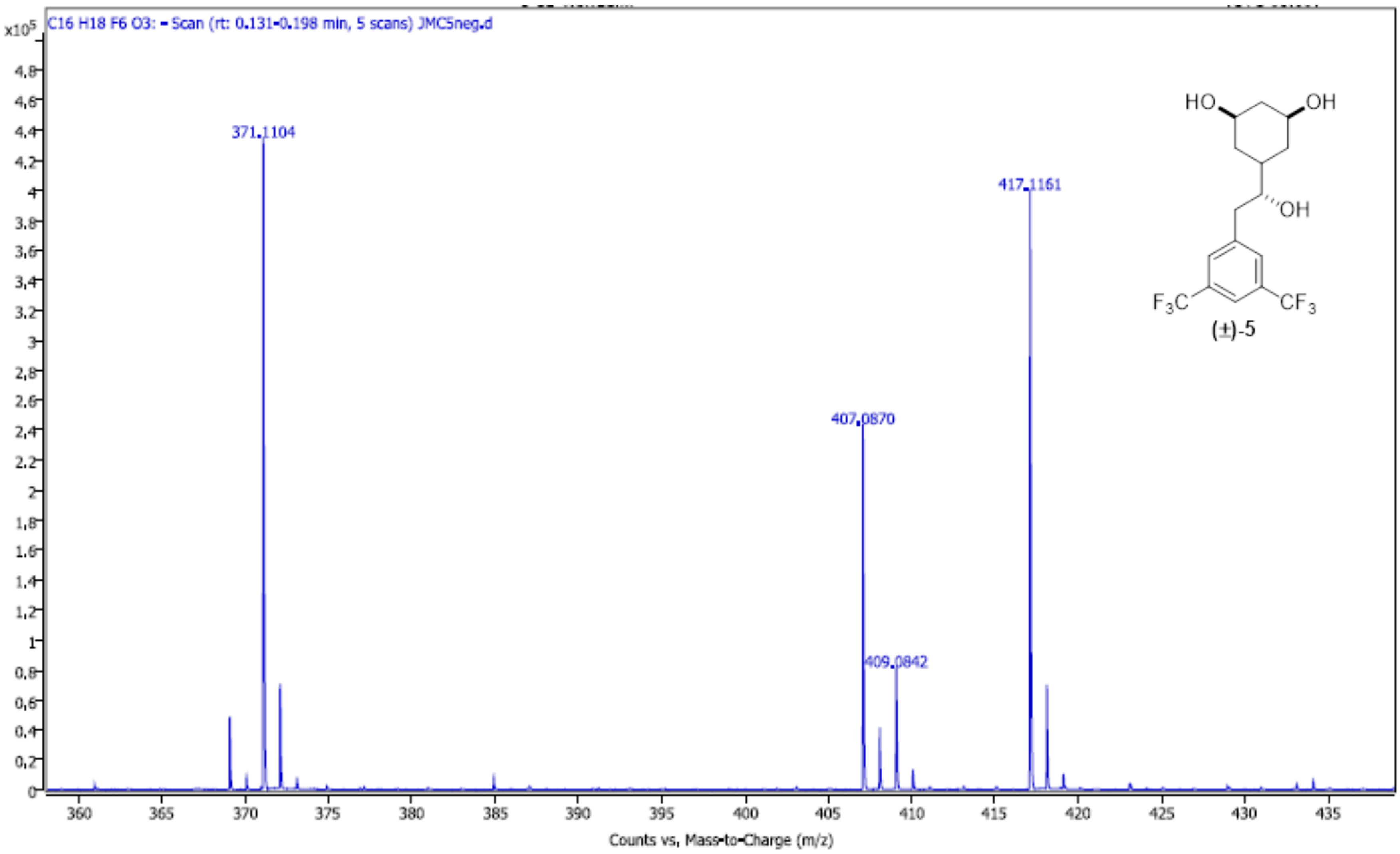
C16 H18 F6 O3: - Scan (rt: 0.131-0.198 min, 5 scans) JMC5neg.d
x10^5
371.1104
417.1161
407.0870
409.0842
Counts vs. Mass-to-Charge (m/z)
HO
OH
OH
F3C
CF3
(±)-5

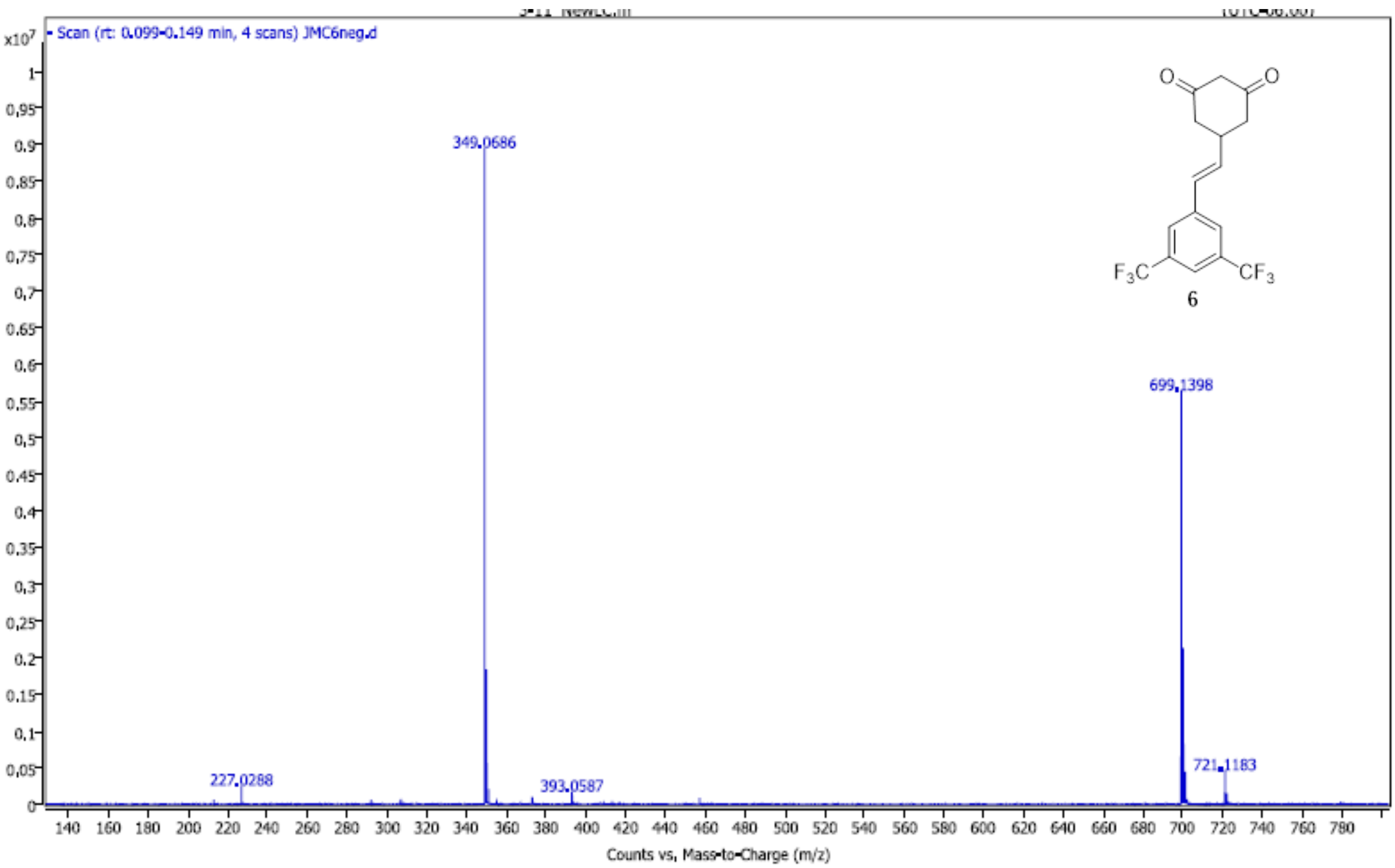

Scan (rt: 0.099-0.149 min, 4 scans) JMC6neg.d
x10^7
349.0686
699.1398
227.0288
393.0587
721.1183
O
O
F3C
CF3
6
Counts vs. Mass-to-Charge (m/z)

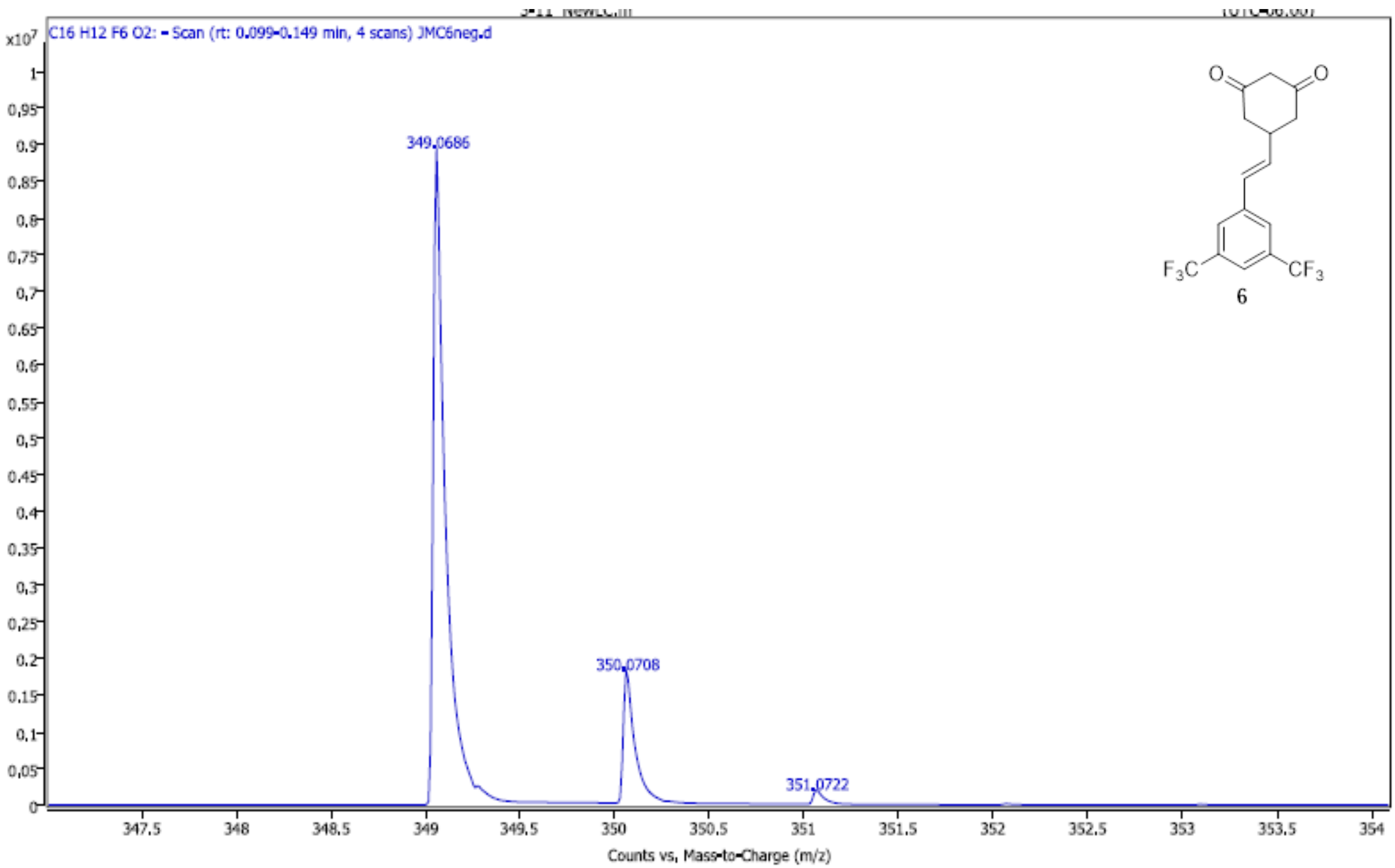

C16 H12 F6 O2: - Scan (rt: 0.099-0.149 min, 4 scans) JMC6neg.d
x10^7
349.0686
350.0708
351.0722
Counts vs, Mass-to-Charge (m/z)
O
O
F3C
CF3
6

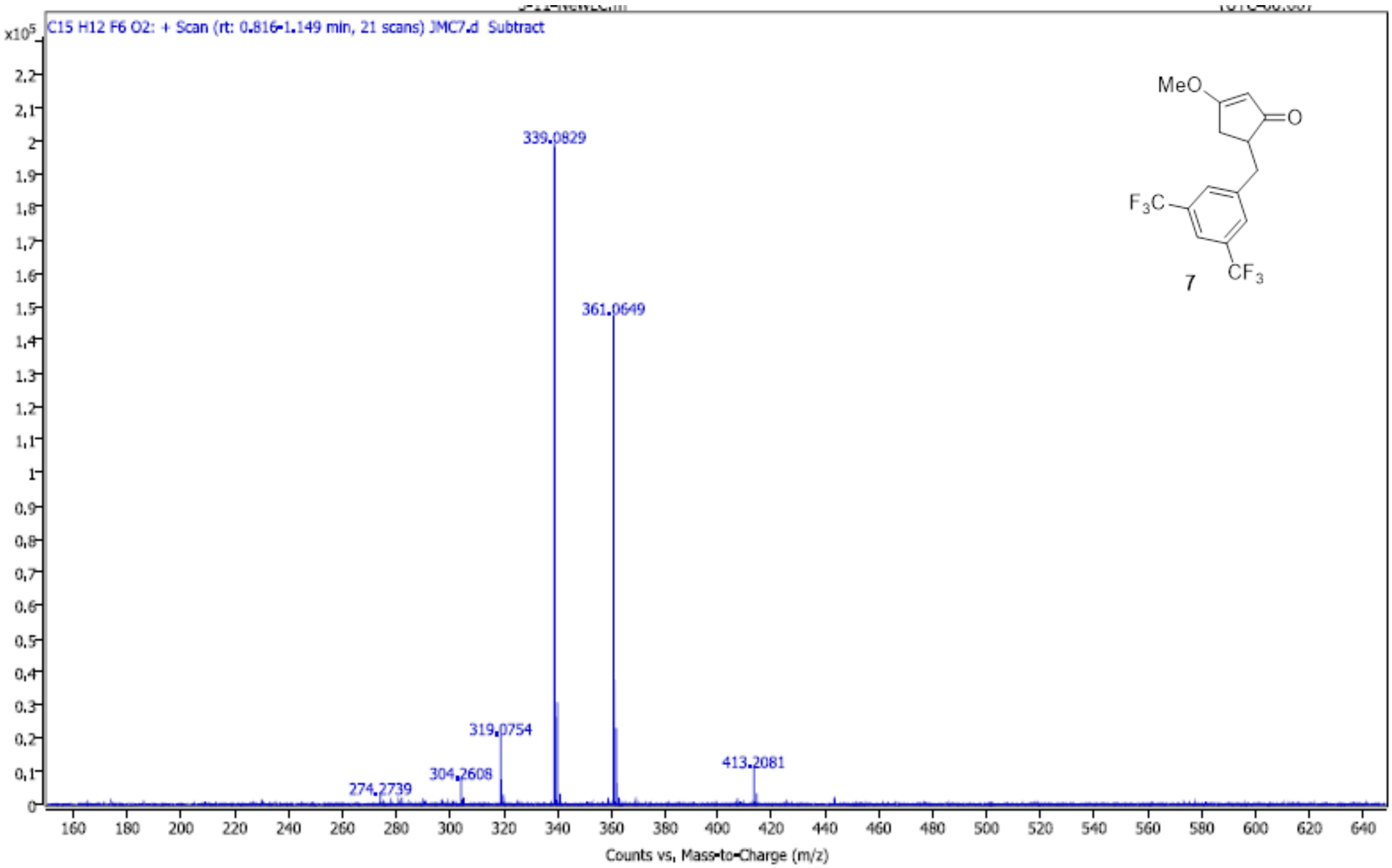
C15 H12 F6 O2: + Scan (rt: 0.816-1.149 min, 21 scans) JMC7.d Subtract
x10^5
339.0829
361.0649
319.0754
304.2608
274.2739
413.2081
Counts vs. Mass-to-Charge (m/z)
MeO
O
F3C
CF3
7

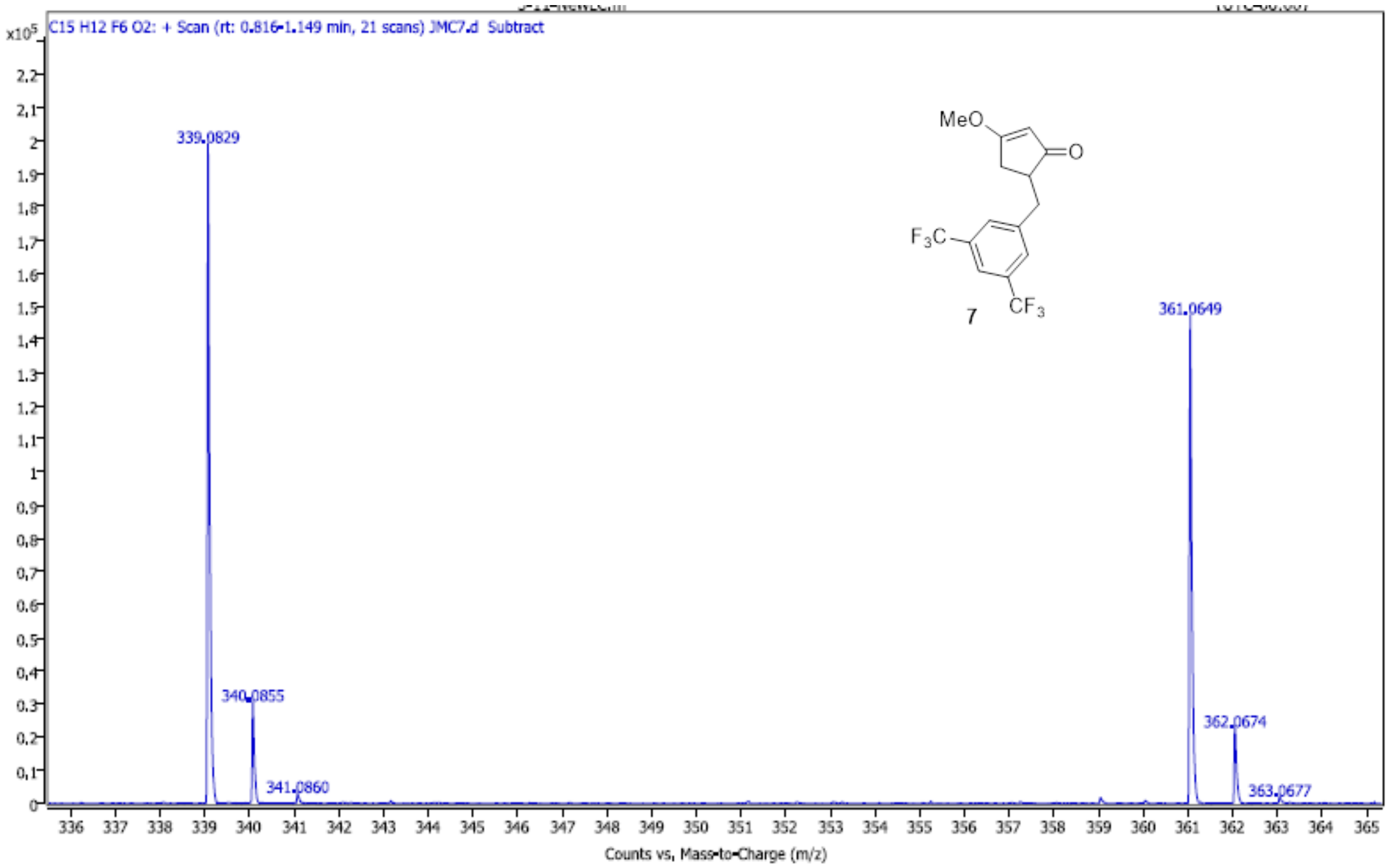
C15 H12 F6 O2: + Scan (rt: 0.816-1.149 min, 21 scans) JMC7.d Subtract
x10^5
339.0829
340.0855
341.0860
361.0649
362.0674
363.0677
MeO
O
F3C
CF3
7
Counts vs. Mass-to-Charge (m/z)

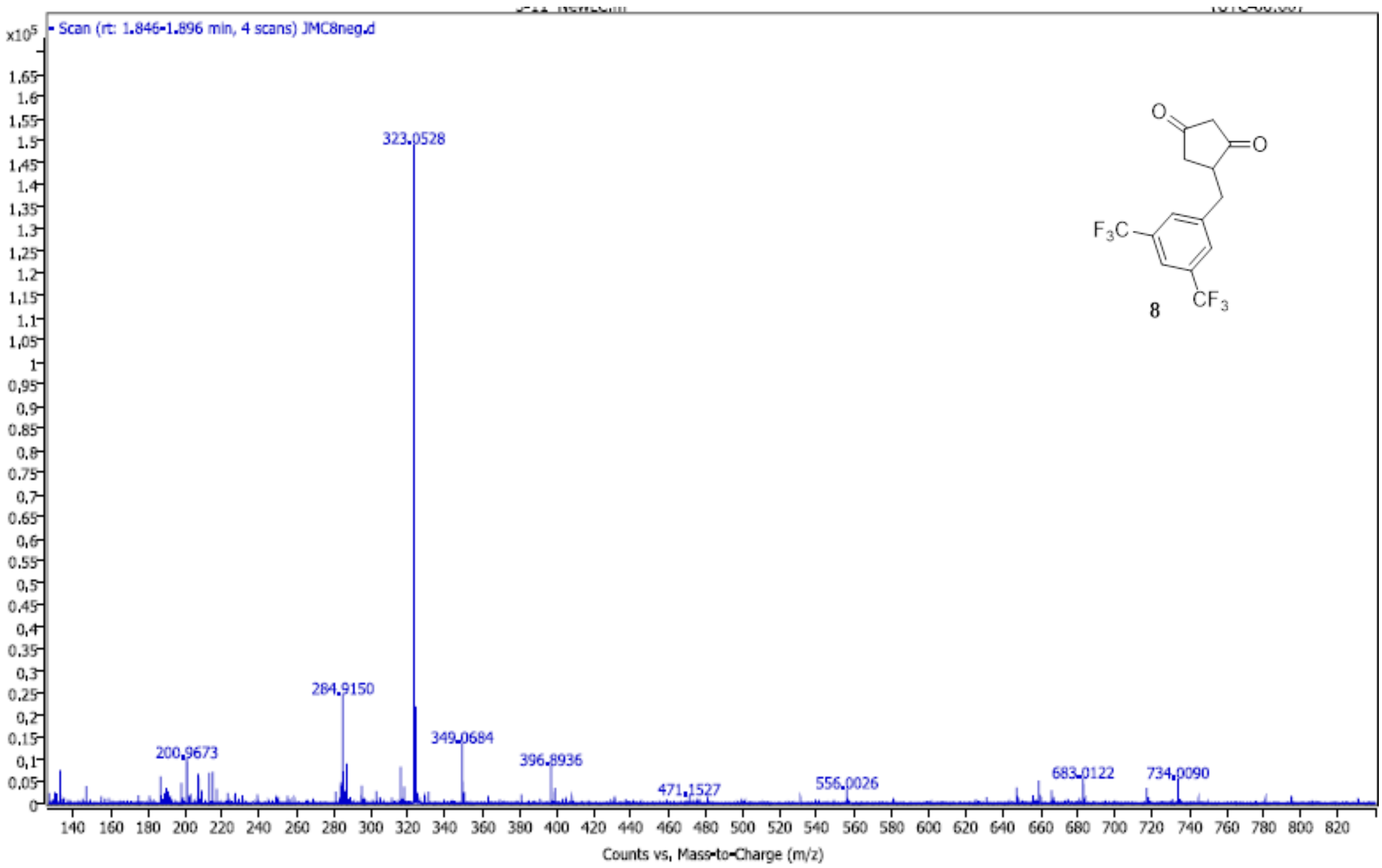
x10^5
Scan (rt: 1.846-1.896 min, 4 scans) JMC8neg.d
323.0528
284.9150
349.0684
200.9673
396.8936
471.1527
556.0026
683.0122
734.0090
F3C
CF3
8
Counts vs. Mass-to-Charge (m/z)

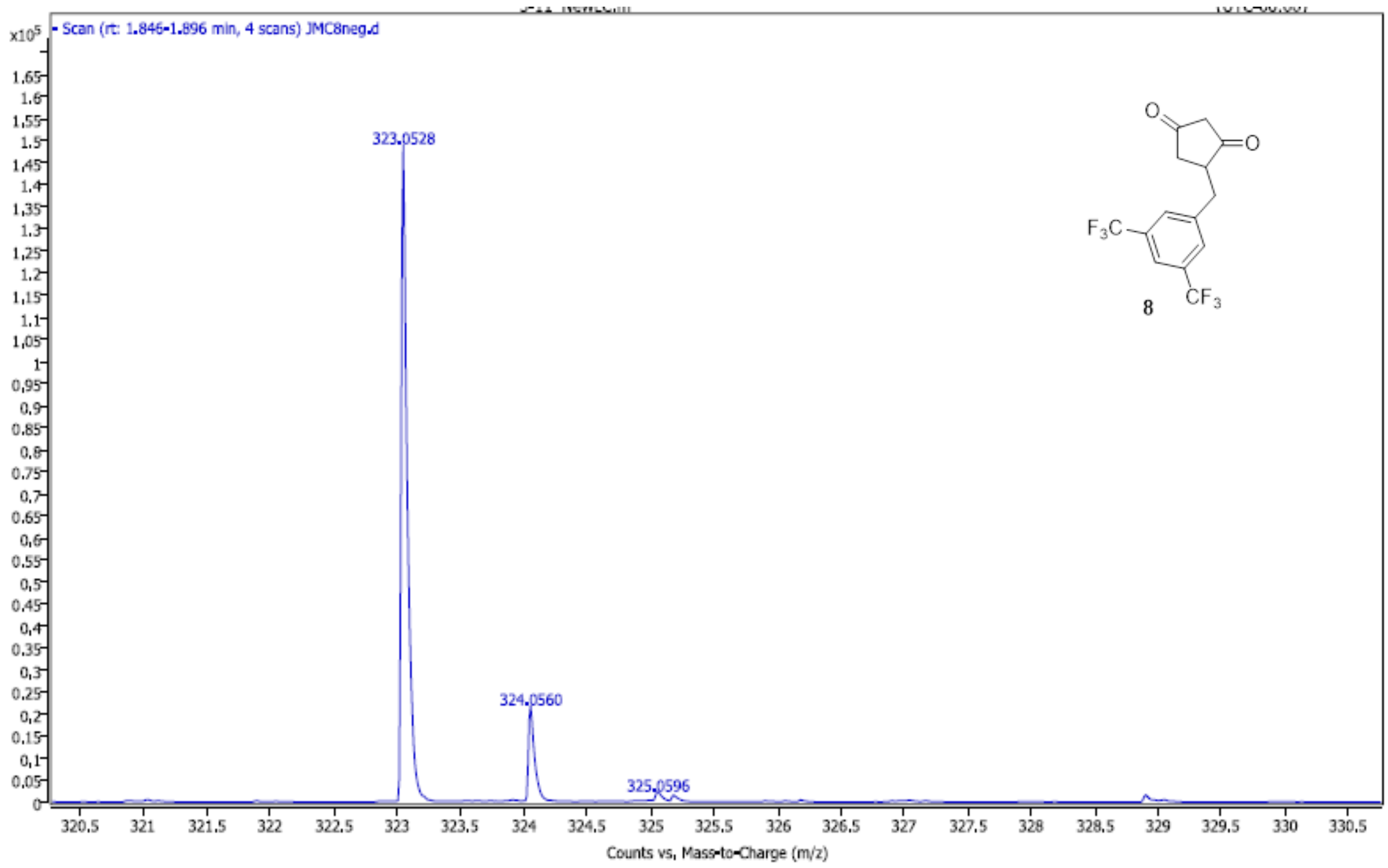

Scan (rt: 1.846-1.896 min, 4 scans) JMC8neg.d
x10^5
323.0528
324.0560
325.0596
Counts vs. Mass-to-Charge (m/z)
F3C
CF3
8

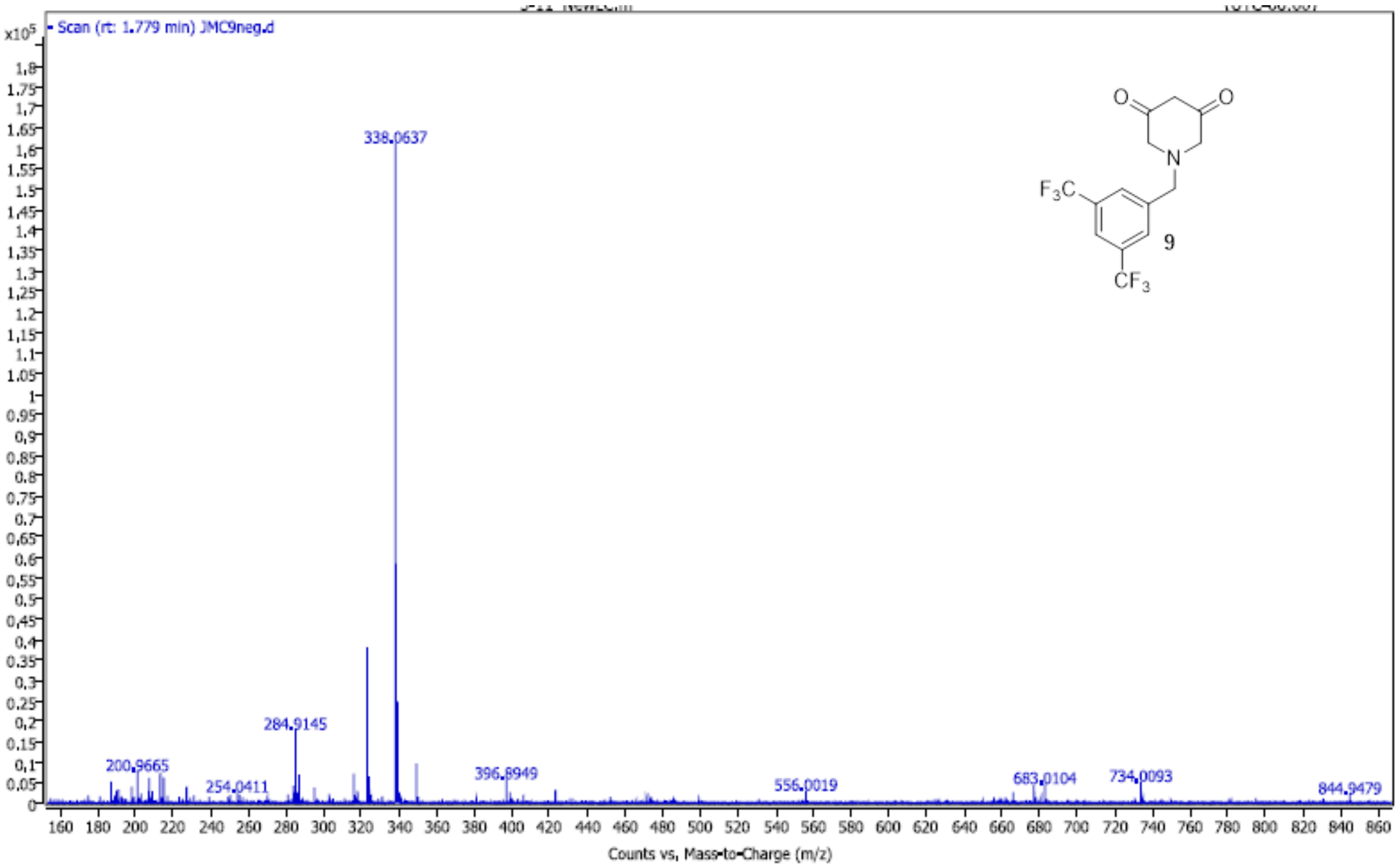

Scan (rt: 1.779 min) JMC9neg.d
x10^5
338.0637
284.9145
200.9665
254.0411
396.8949
556.0019
683.0104
734.0093
844.9479
Counts vs. Mass-to-Charge (m/z)
9

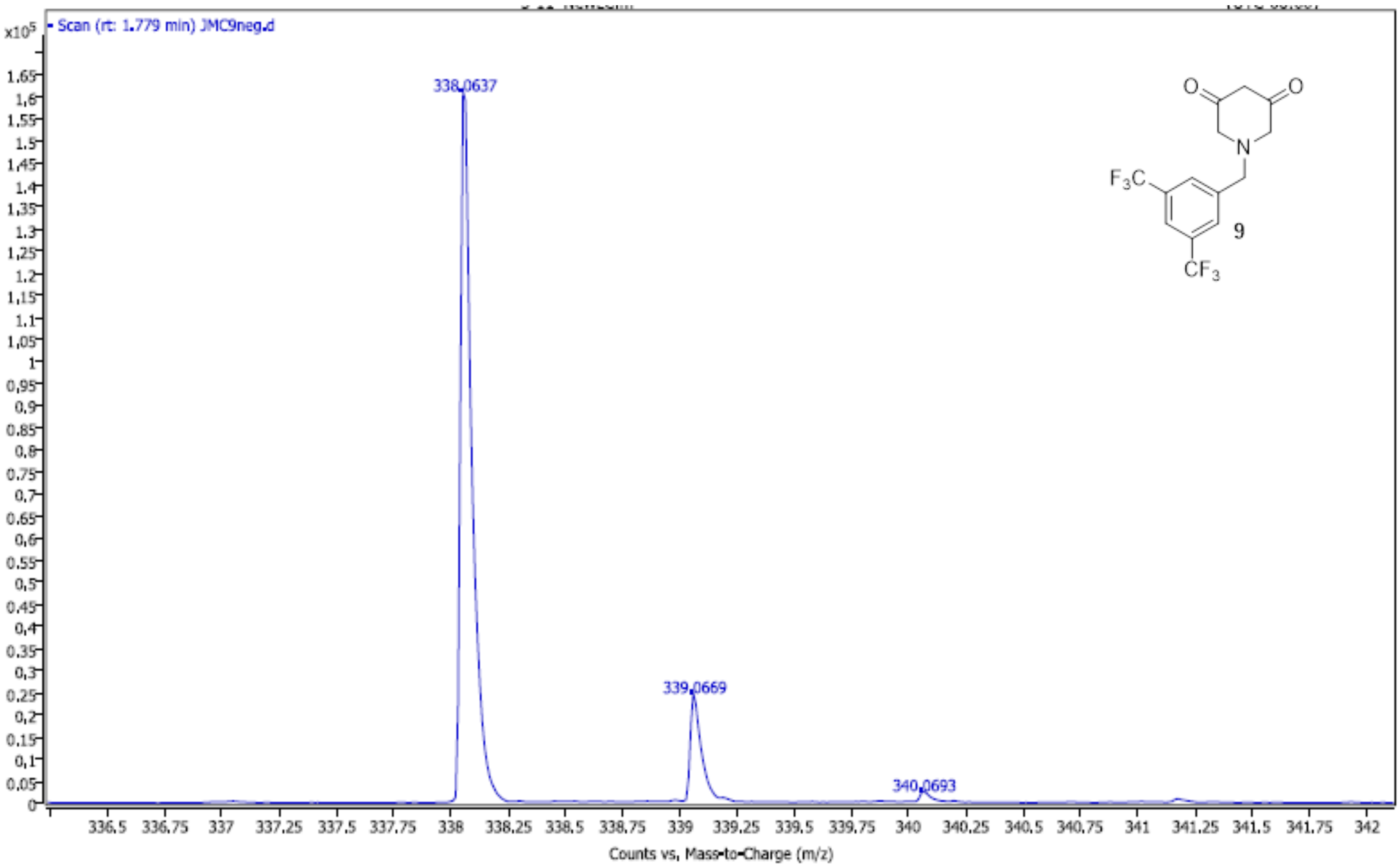
x10^5
Scan (rt: 1.779 min) JMC9neg.d
338.0637
339.0669
340.0693
O
O
N
F3C
CF3
9
Counts vs. Mass-to-Charge (m/z)

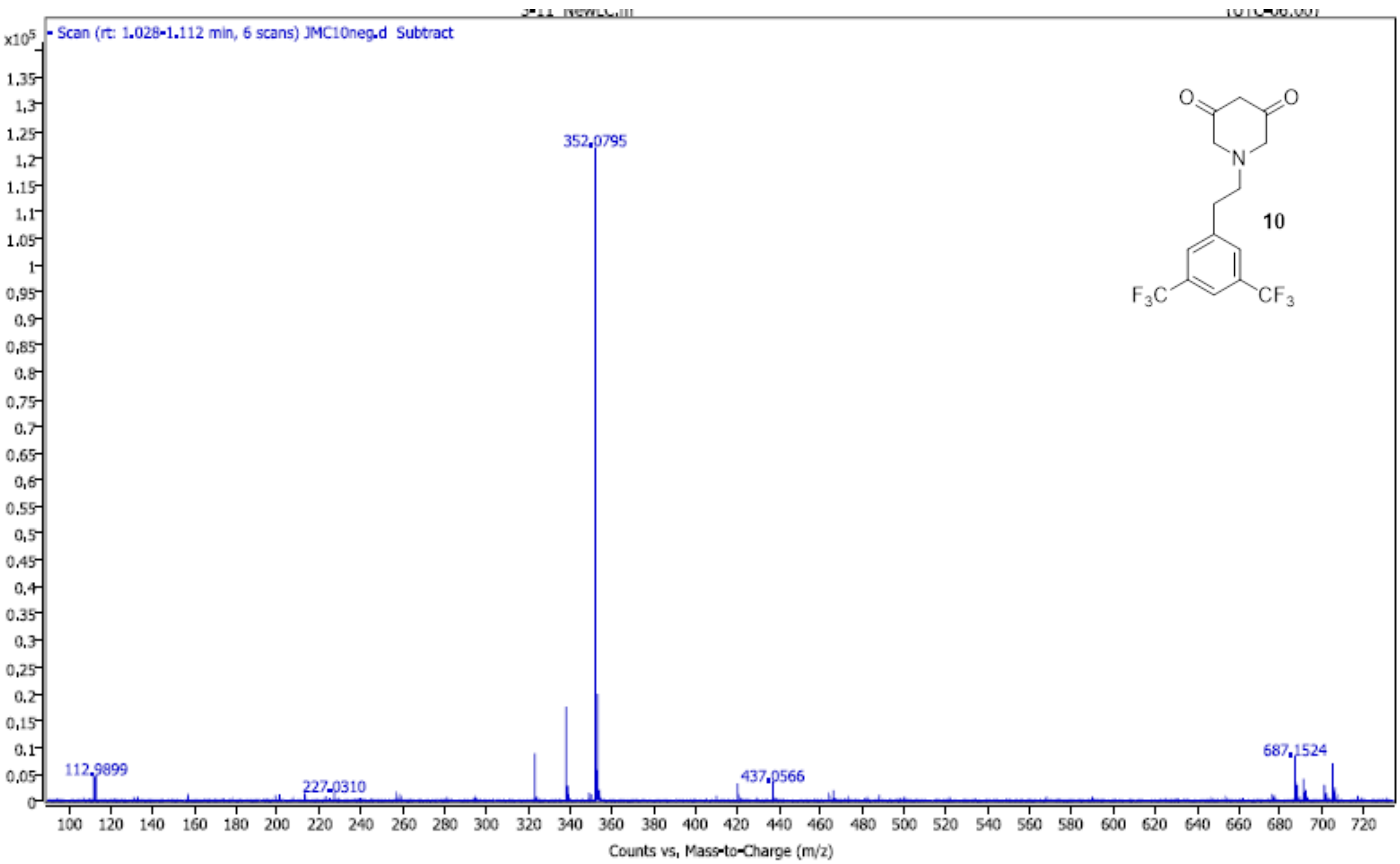

Scan (rt: 1.028-1.112 min, 6 scans) JMC10neg.d Subtract
x10^5
352.0795
112.9899
227.0310
437.0566
687.1524
10
O
N
F3C
CF3
Counts vs. Mass-to-Charge (m/z)

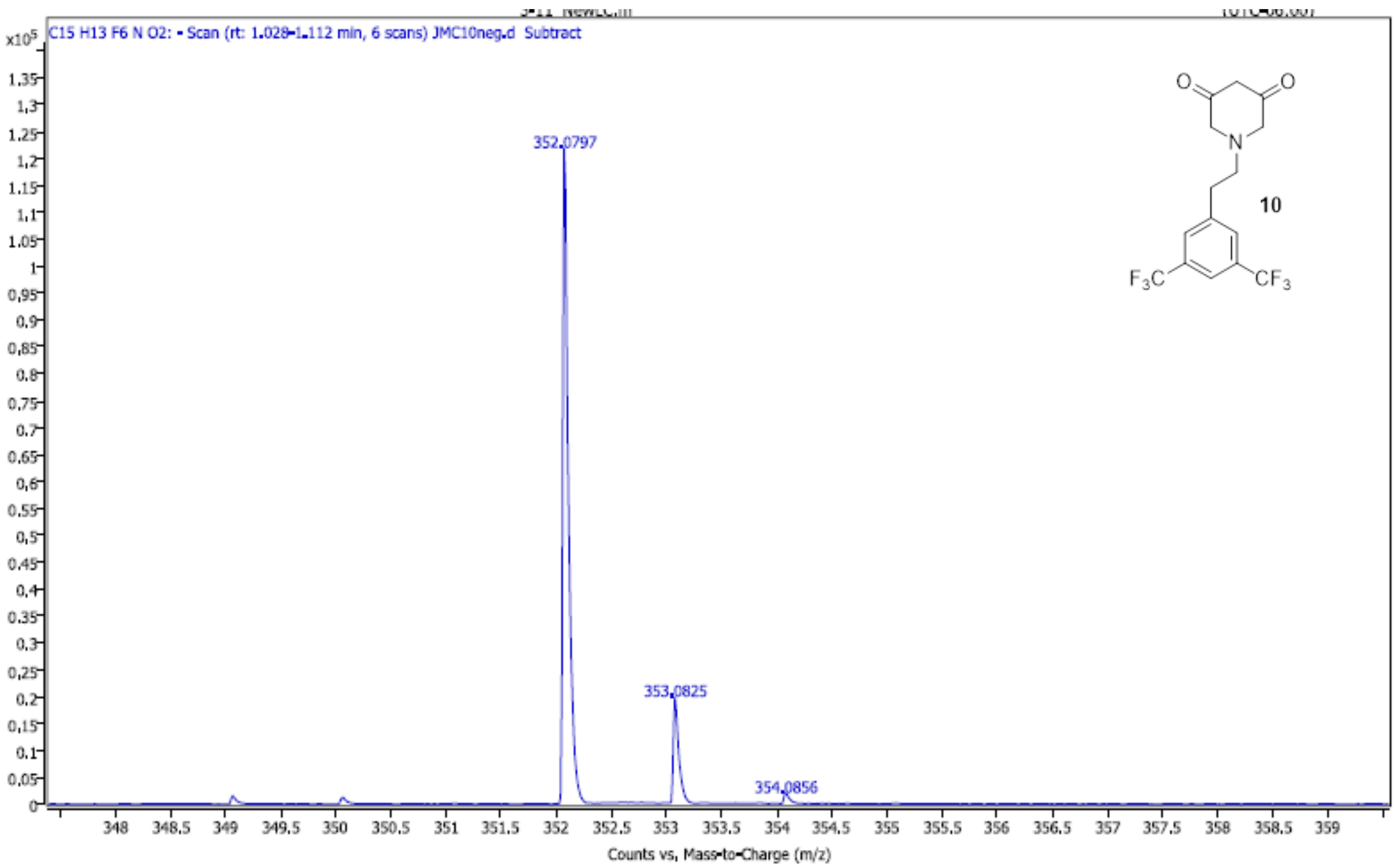
x10^5
C15 H13 F6 N O2: - Scan (rt: 1.028-1.112 min, 6 scans) JMC10neg.d Subtract
352.0797
353.0825
354.0856
O
O
N
10
F3C
CF3
Counts vs. Mass-to-Charge (m/z)

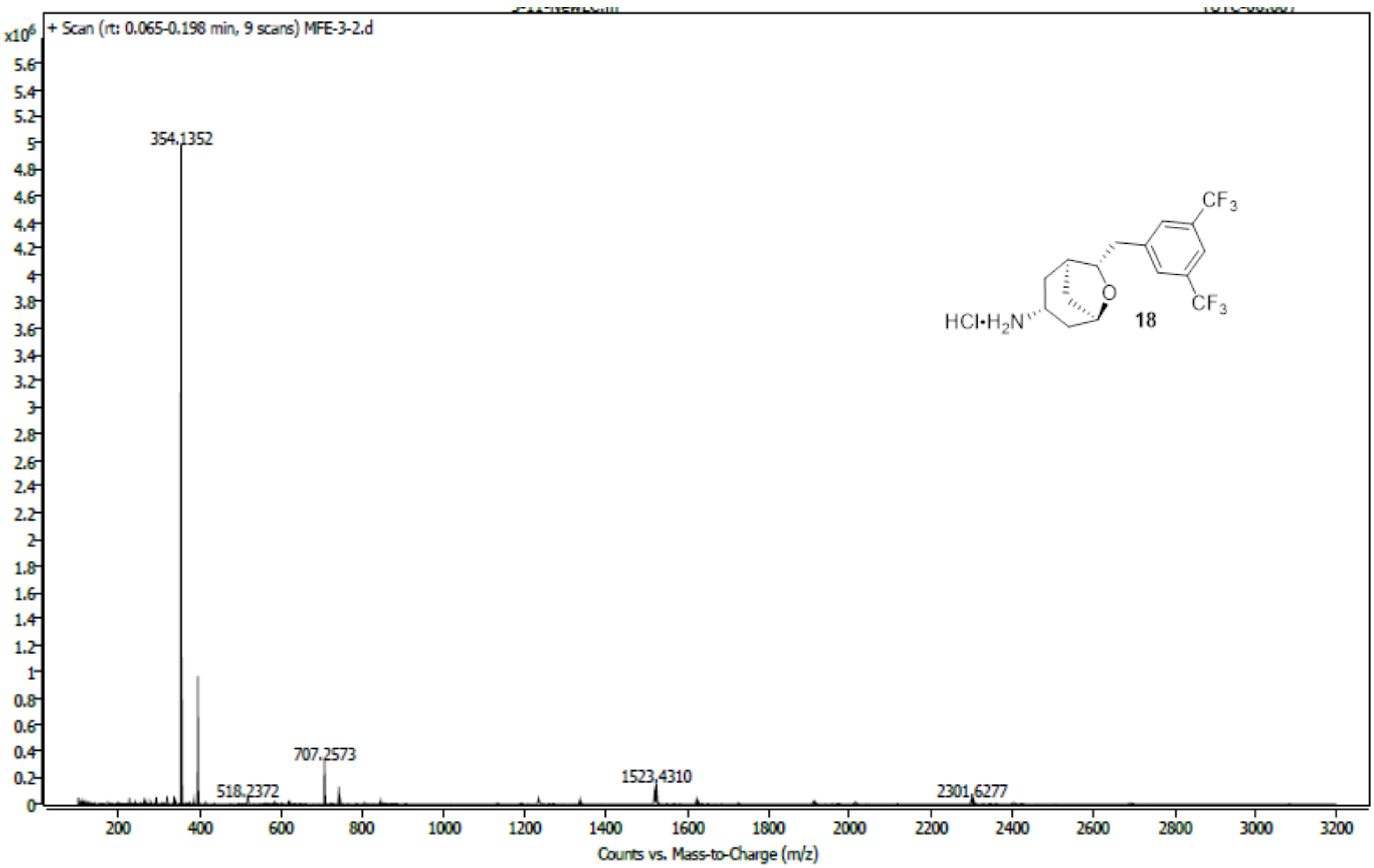

+ Scan (rt: 0.065-0.198 min, 9 scans) MFE-3-2.d
x10^6
354.1352
707.2573
518.2372
1523.4310
2301.6277
Counts vs. Mass-to-Charge (m/z)
CF3
CF3
O
HCl•H2N
18

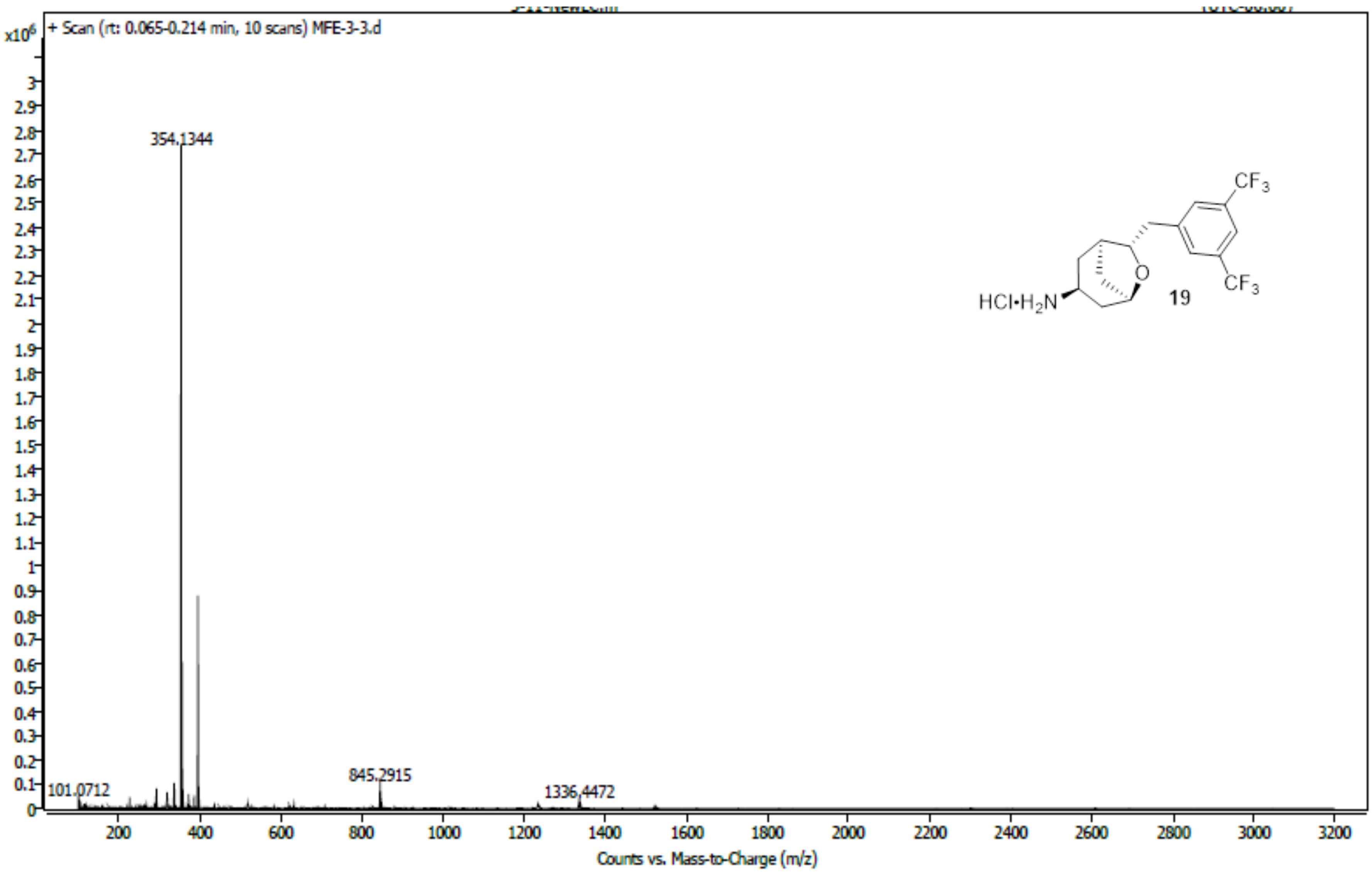

x10^6
+ Scan (rt: 0.065-0.214 min, 10 scans) MFE-3-3.d
354.1344
845.2915
1336.4472
101.0712
Counts vs. Mass-to-Charge (m/z)
CF3
CF3
HCl•H2N
O
19

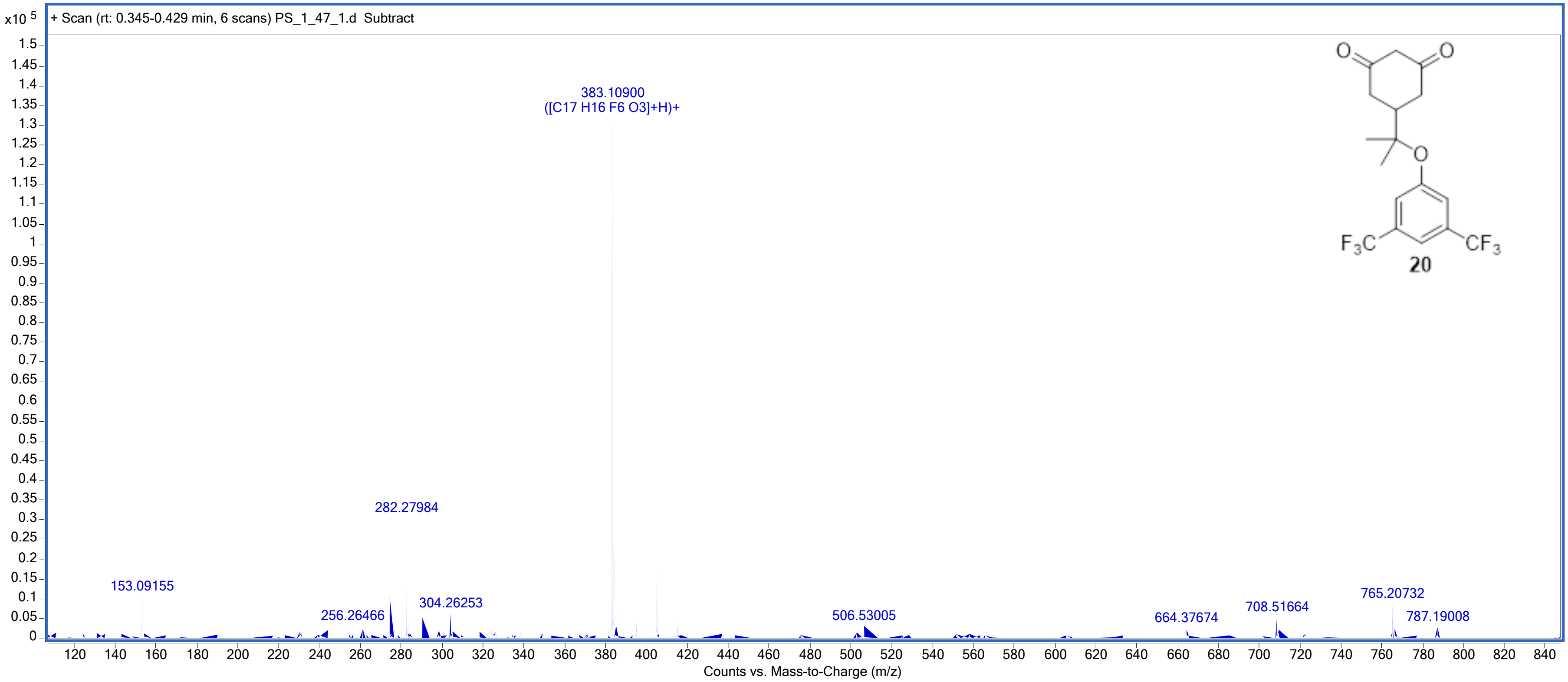

x10 5
+ Scan (rt: 0.345-0.429 min, 6 scans) PS_1_47_1.d Subtract
383.10900
([C17 H16 F6 O3]+H)+
282.27984
153.09155
256.26466
304.26253
506.53005
664.37674
708.51664
765.20732
787.19008
20
Counts vs. Mass-to-Charge (m/z)

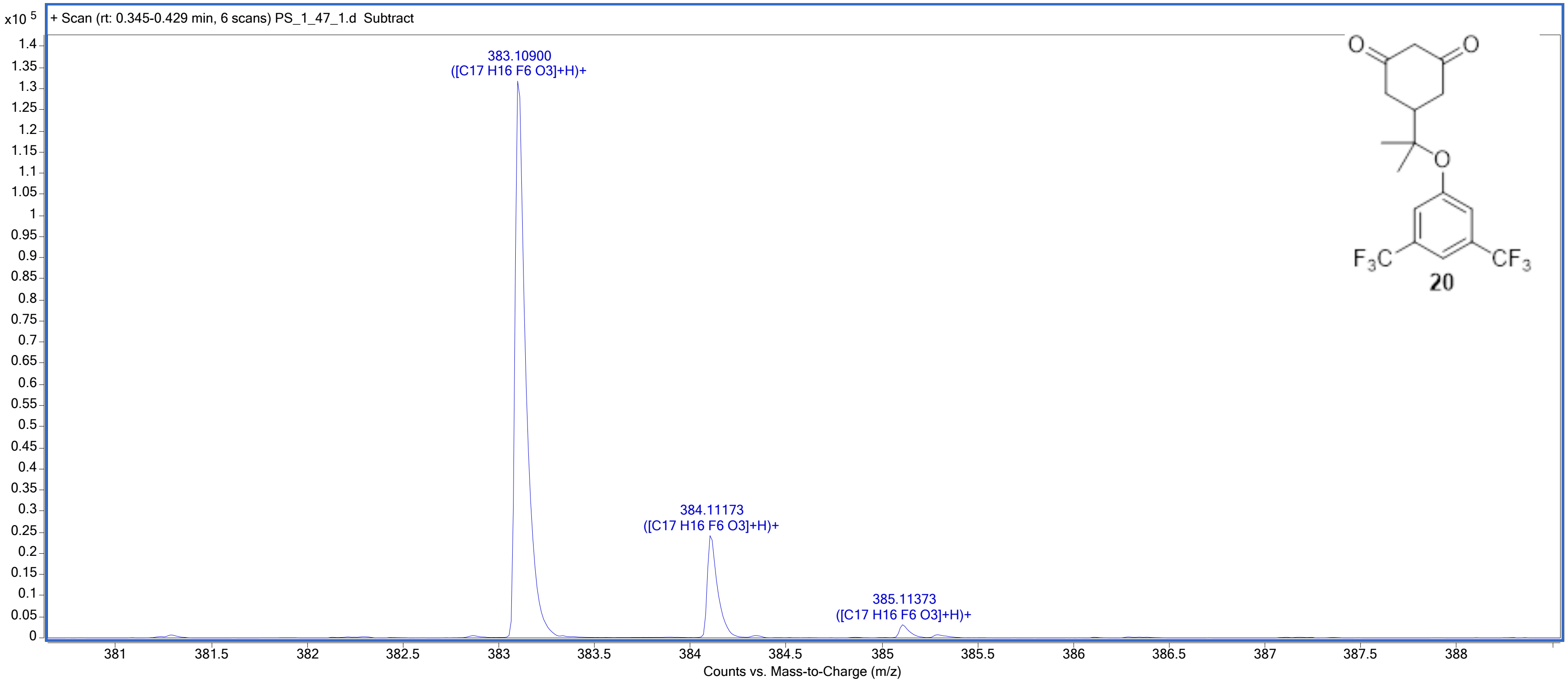
x10 5
+ Scan (rt: 0.345-0.429 min, 6 scans) PS_1_47_1.d Subtract
383.10900
([C17 H16 F6 O3]+H)+
384.11173
([C17 H16 F6 O3]+H)+
385.11373
([C17 H16 F6 O3]+H)+
Counts vs. Mass-to-Charge (m/z)
O
O
O
F3C
CF3
20

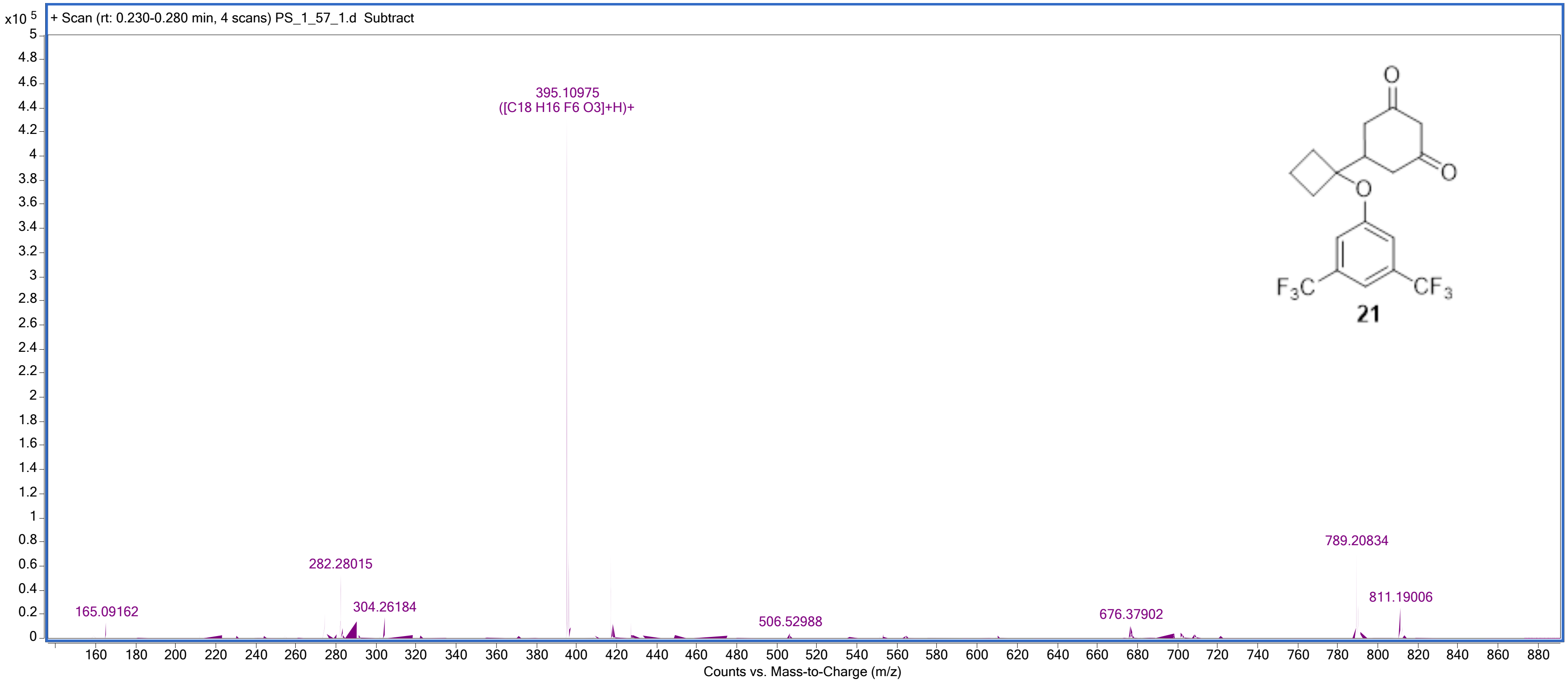
x10 5
+ Scan (rt: 0.230-0.280 min, 4 scans) PS_1_57_1.d Subtract
395.10975
([C18 H16 F6 O3]+H)+
282.28015
304.26184
165.09162
506.52988
676.37902
789.20834
811.19006
Counts vs. Mass-to-Charge (m/z)
O
O
O
F3C
CF3
21

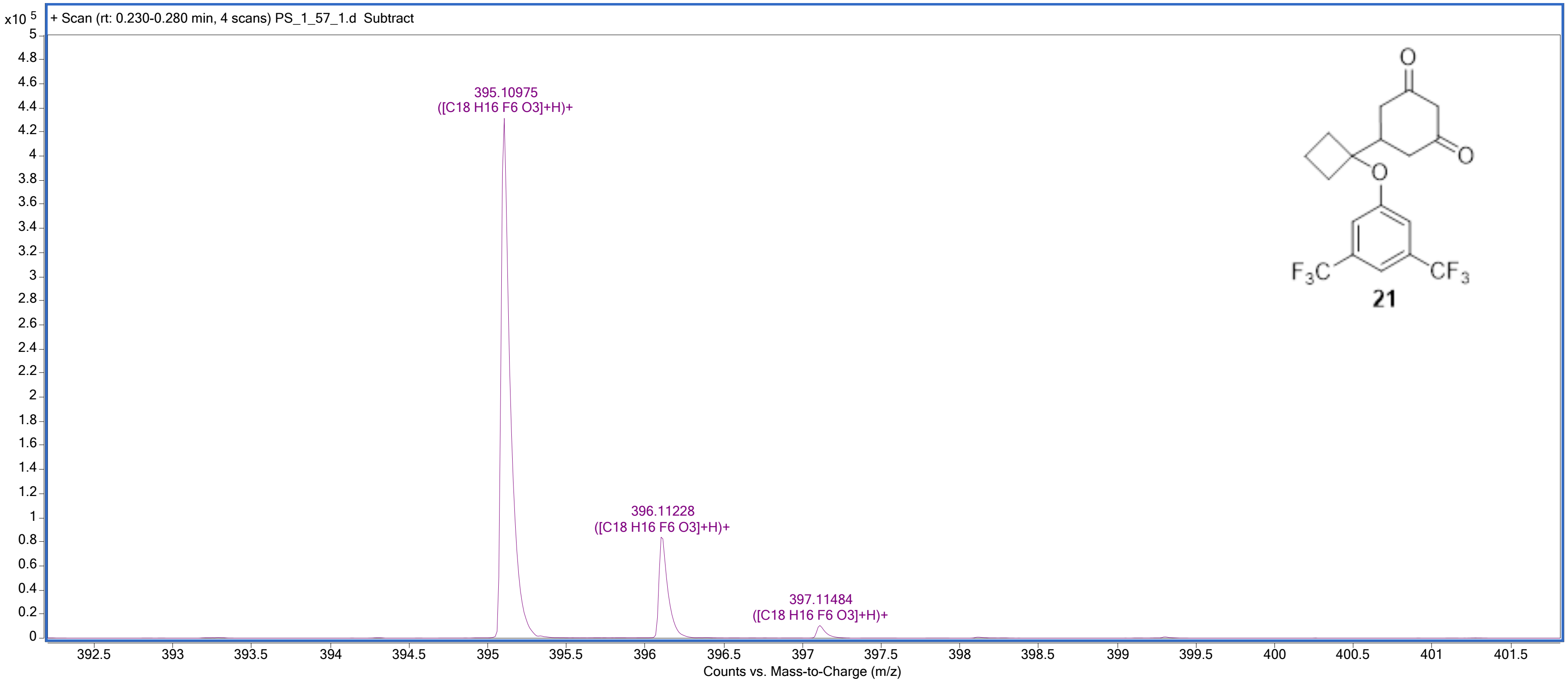
x10 5
+ Scan (rt: 0.230-0.280 min, 4 scans) PS_1_57_1.d Subtract
5
4.8
4.6
4.4
4.2
4
3.8
3.6
3.4
3.2
3
2.8
2.6
2.4
2.2
2
1.8
1.6
1.4
1.2
1
0.8
0.6
0.4
0.2
0
395.10975
([C18 H16 F6 O3]+H)+
396.11228
([C18 H16 F6 O3]+H)+
397.11484
([C18 H16 F6 O3]+H)+
O
O
O
F3C
CF3
21
392.5
393
393.5
394
394.5
395
395.5
396
396.5
397
397.5
398
398.5
399
399.5
400
400.5
401
401.5
Counts vs. Mass-to-Charge (m/z)

3. HPLC Chromatograms

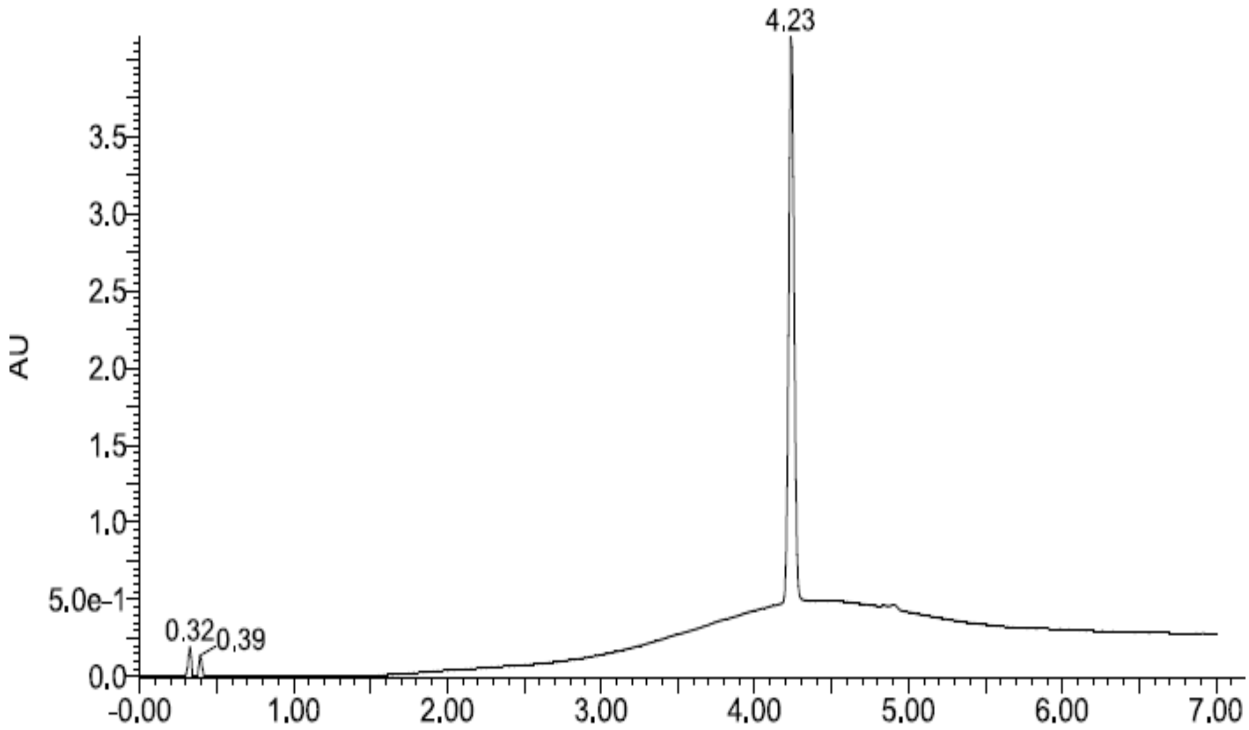

HPLC chromatogram of NU-9 (3)

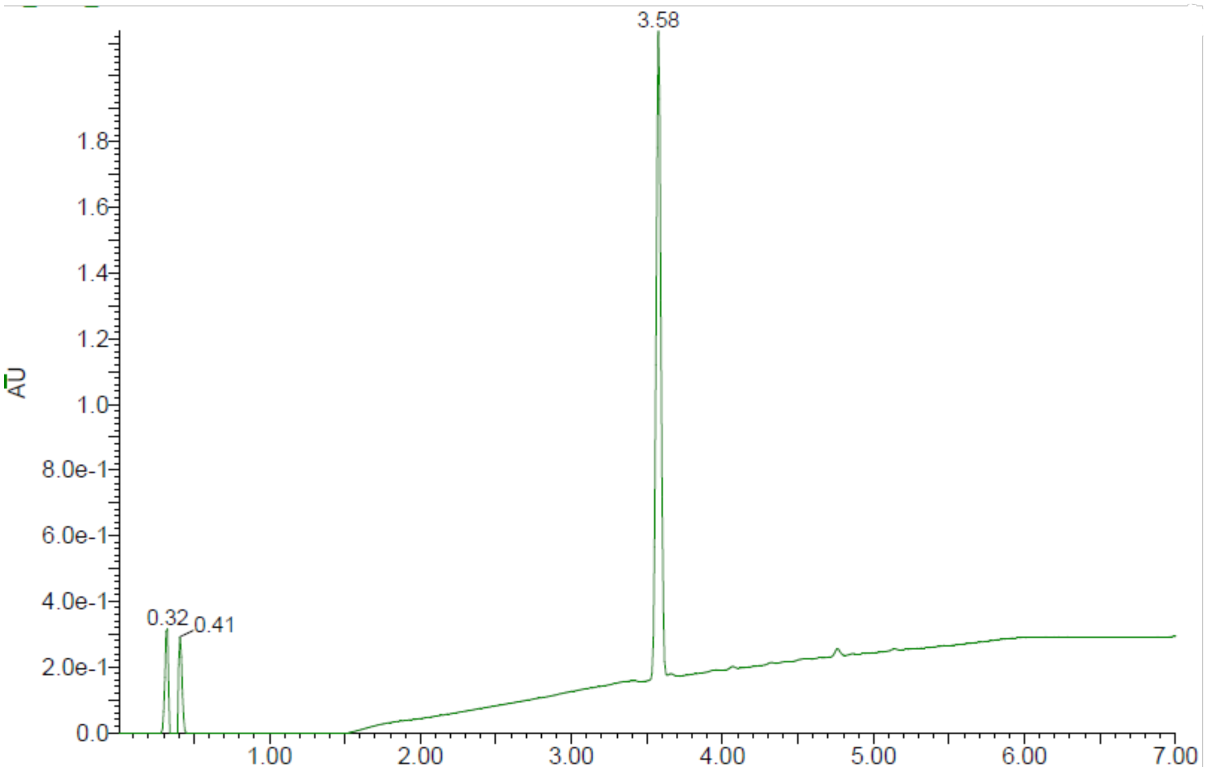

HPLC chromatogram of compound 4

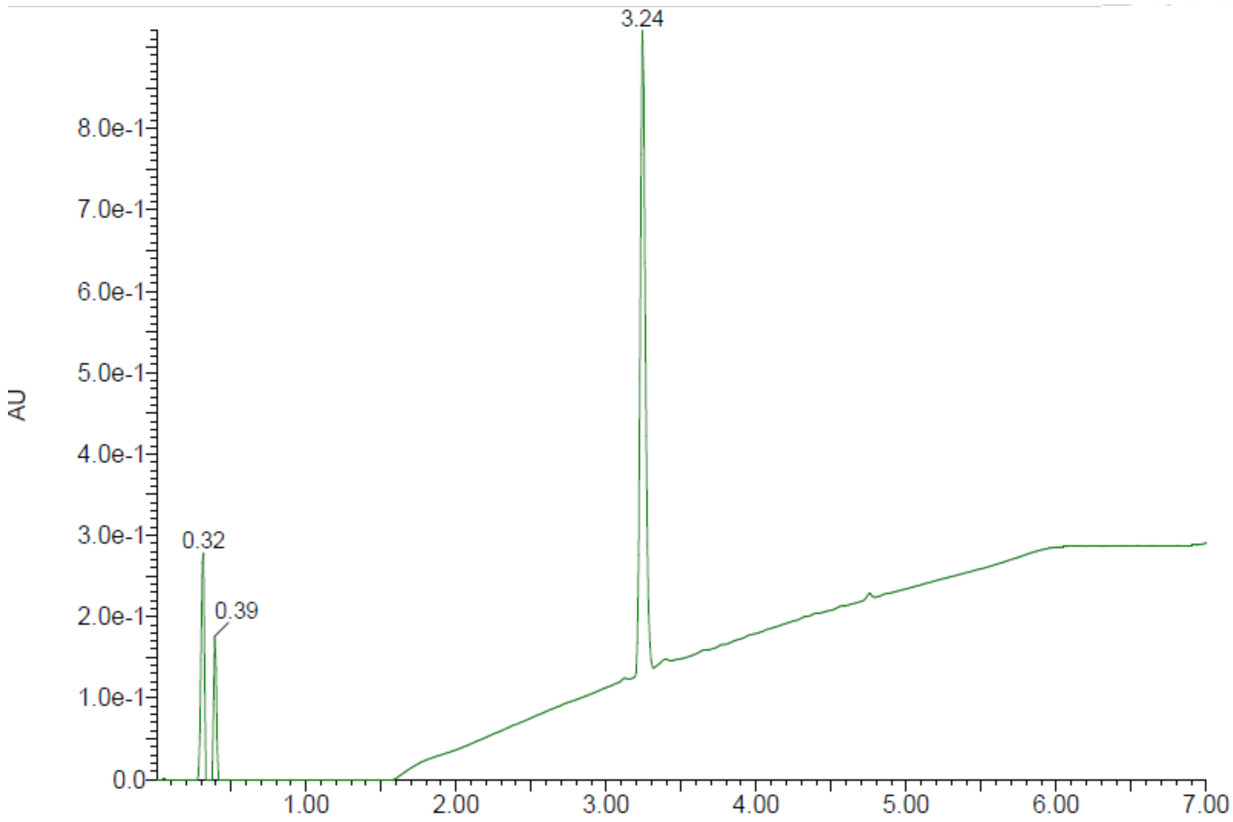

HPLC chromatogram of compound 5

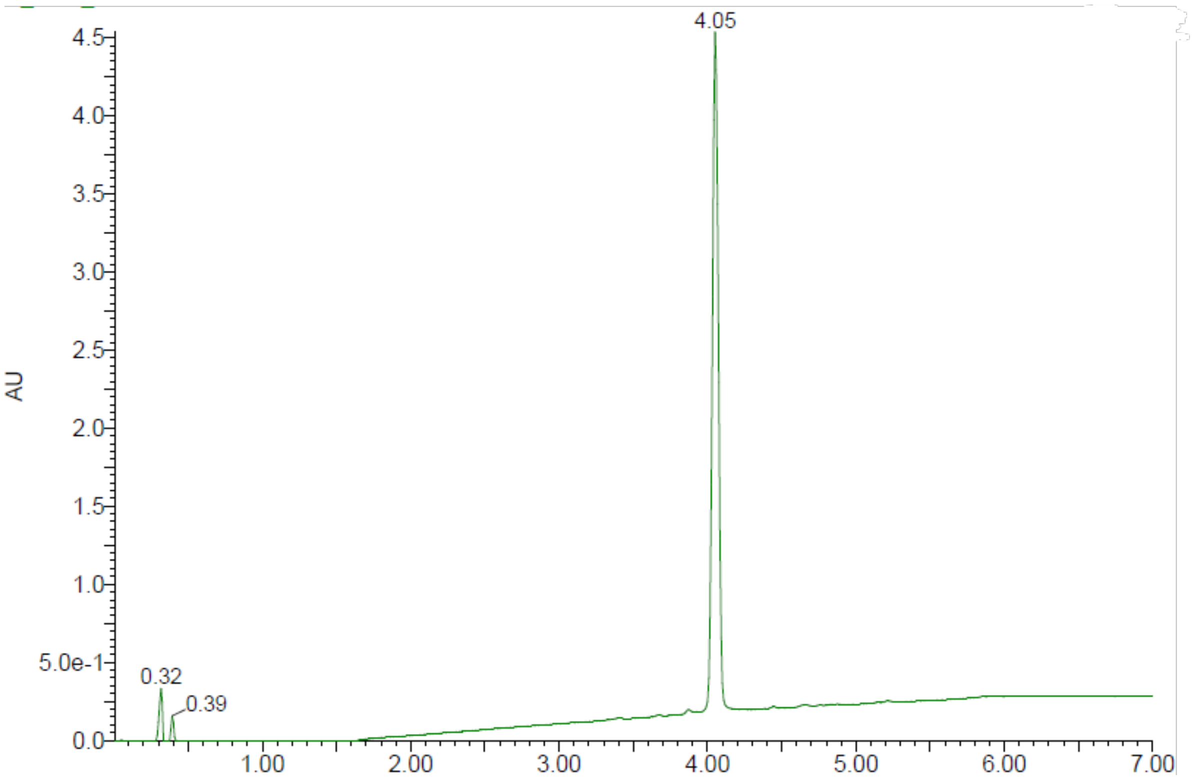

HPLC chromatogram of compound 6

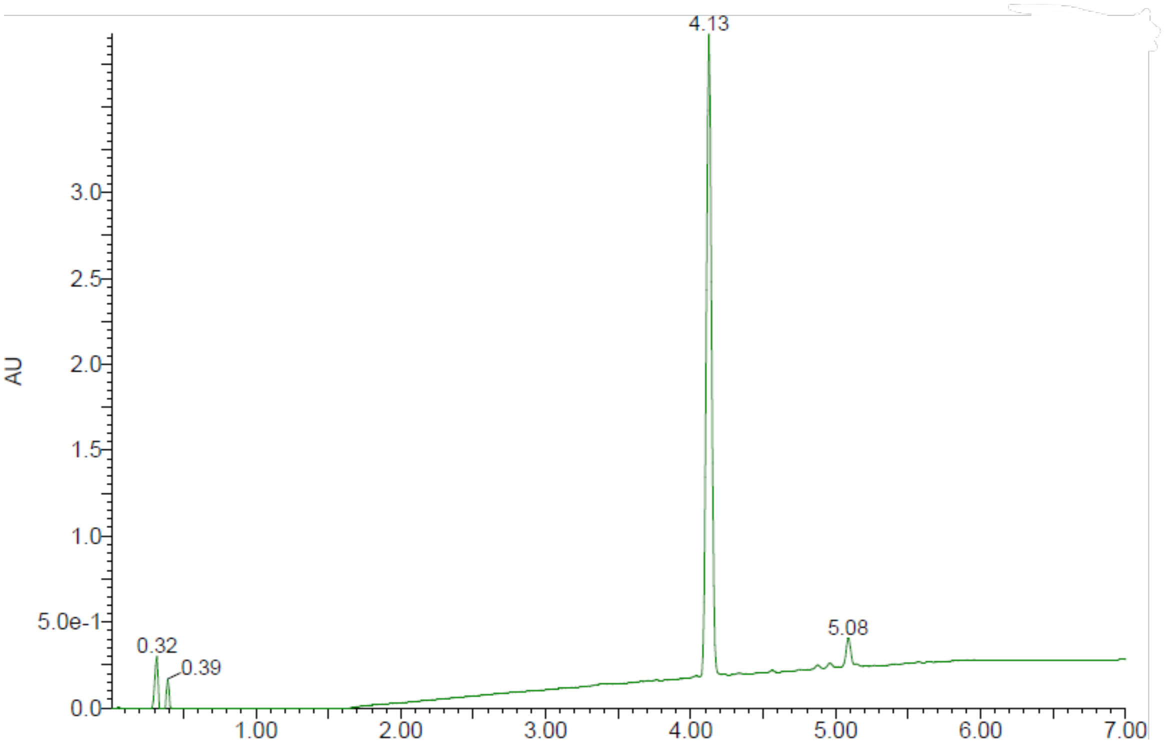

HPLC chromatogram of compound 7

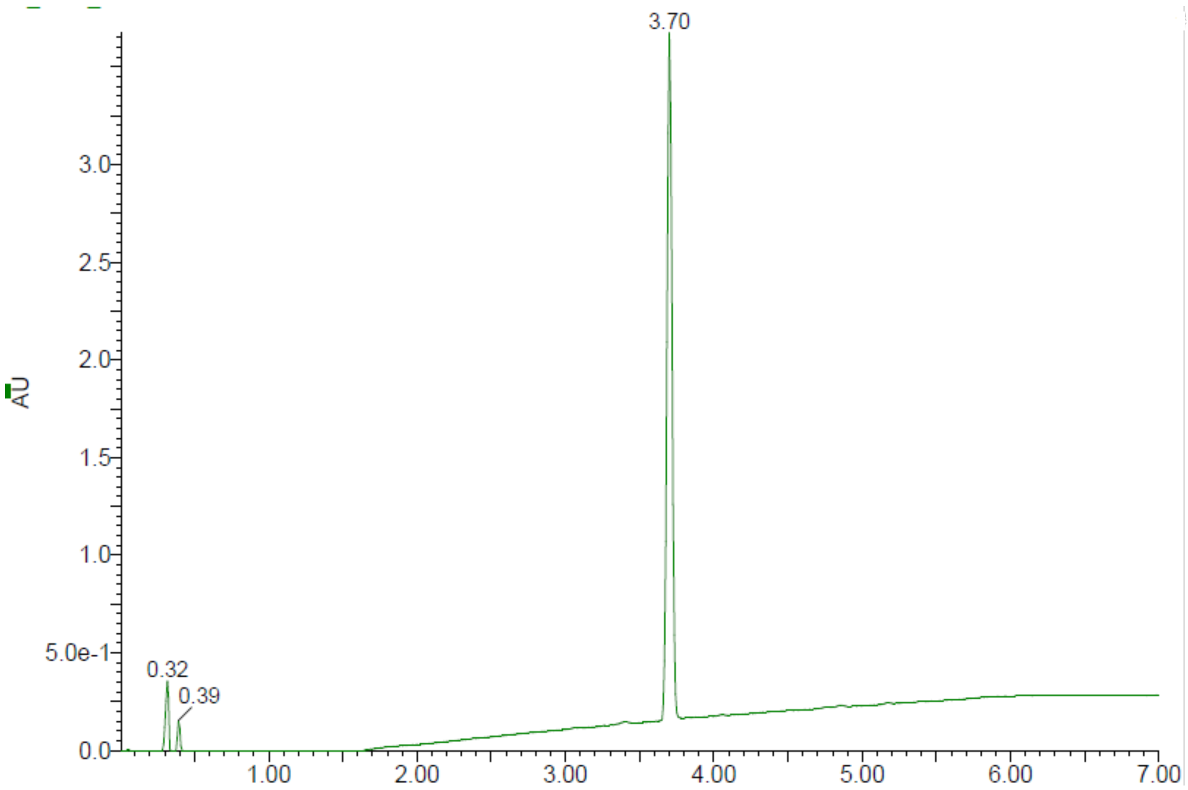

HPLC chromatogram of compound 8

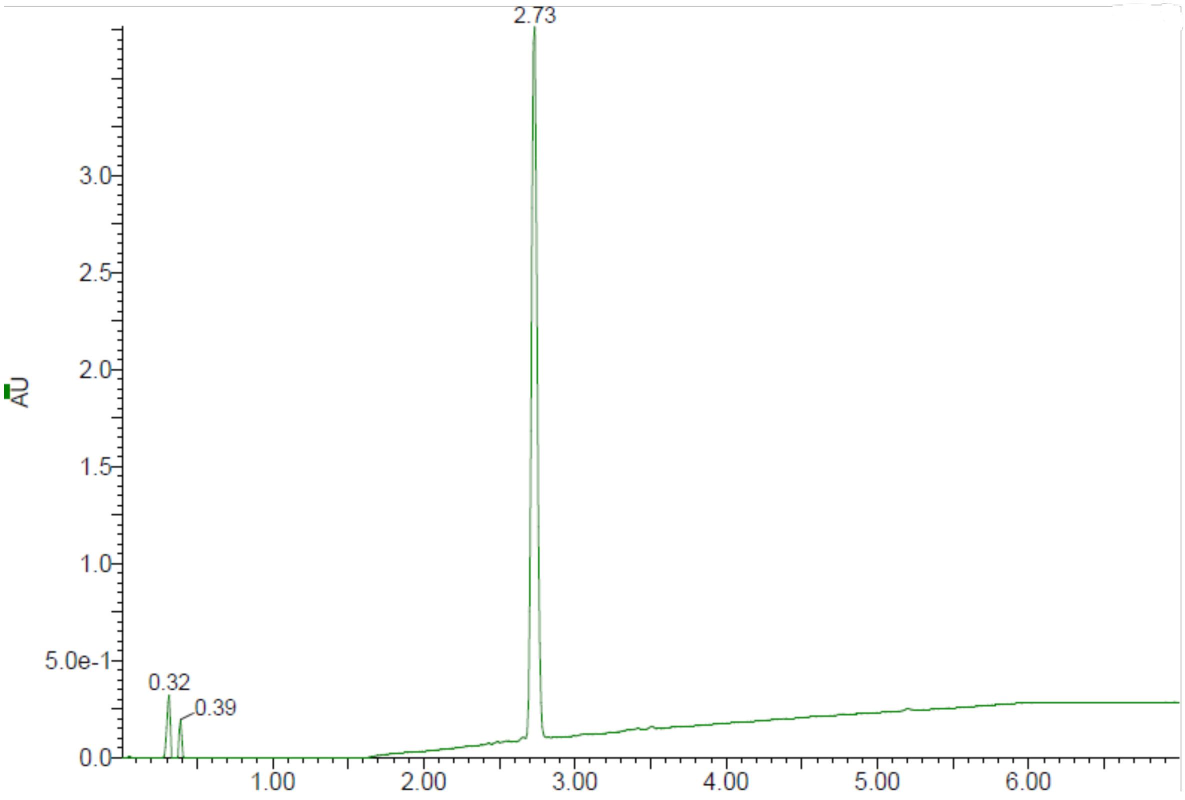

HPLC chromatogram of compound 9

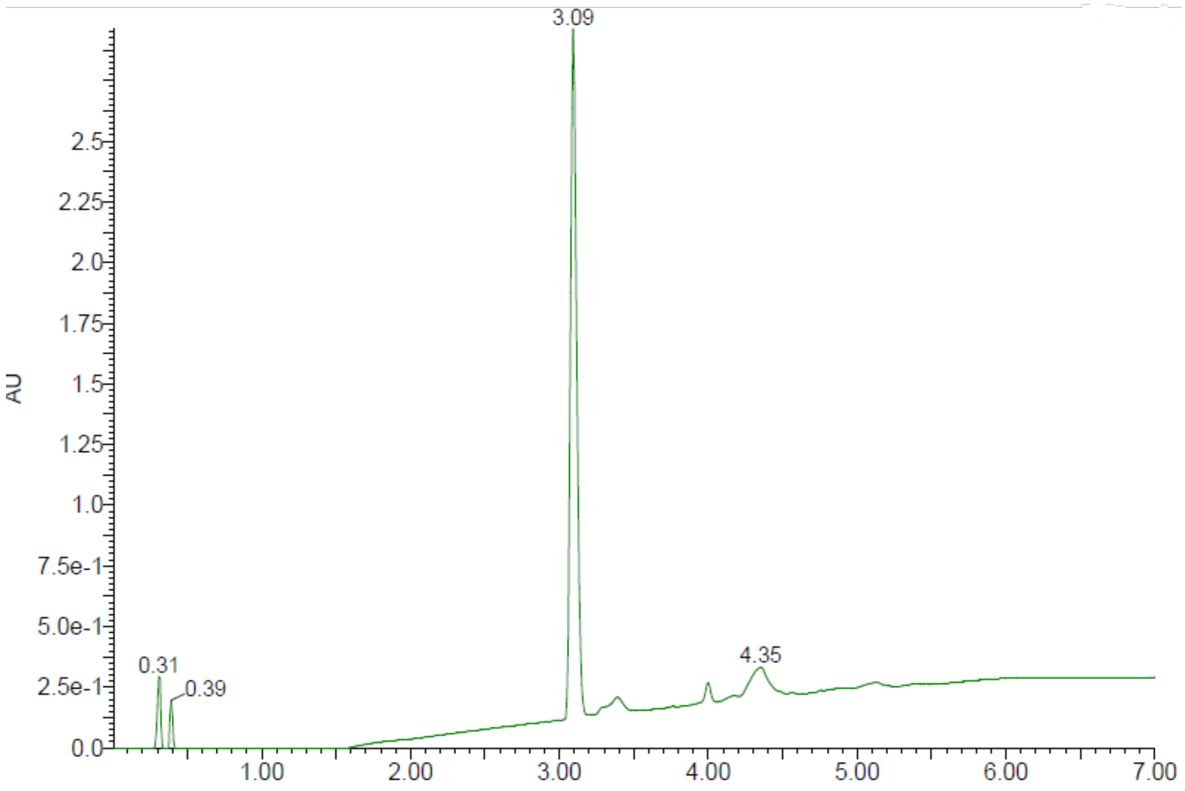

HPLC chromatogram of compound 10

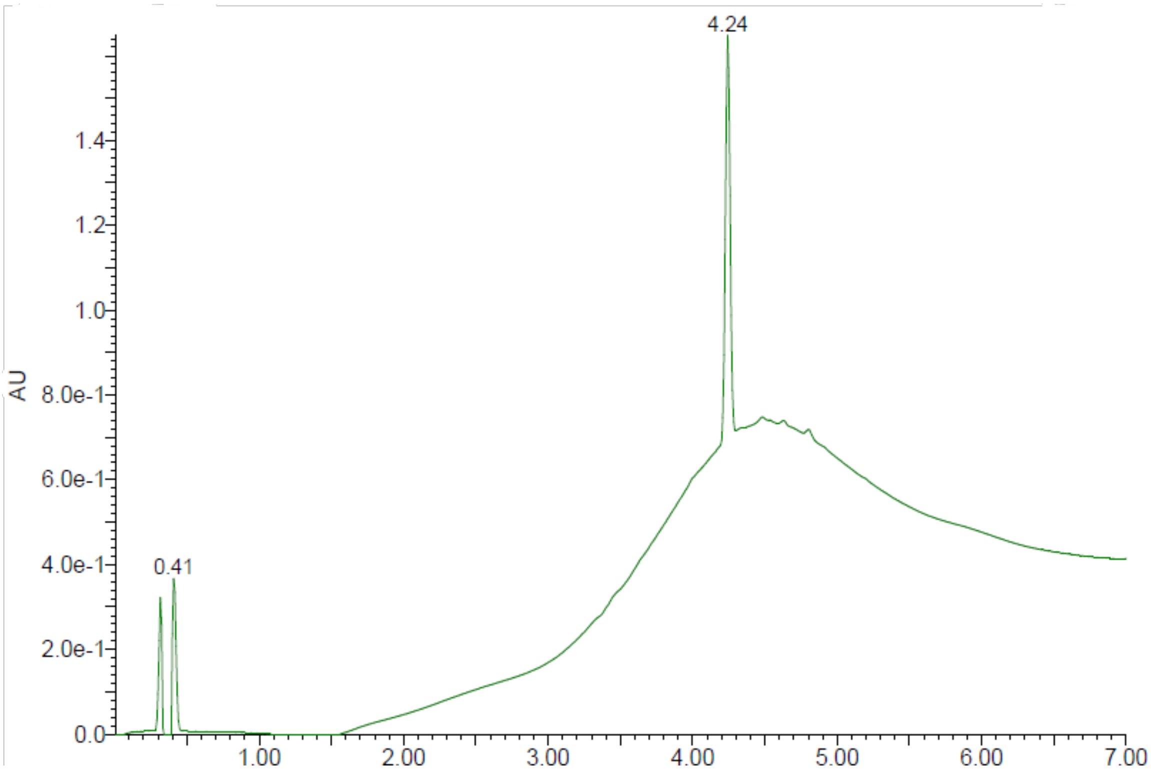

HPLC chromatogram of compound 11

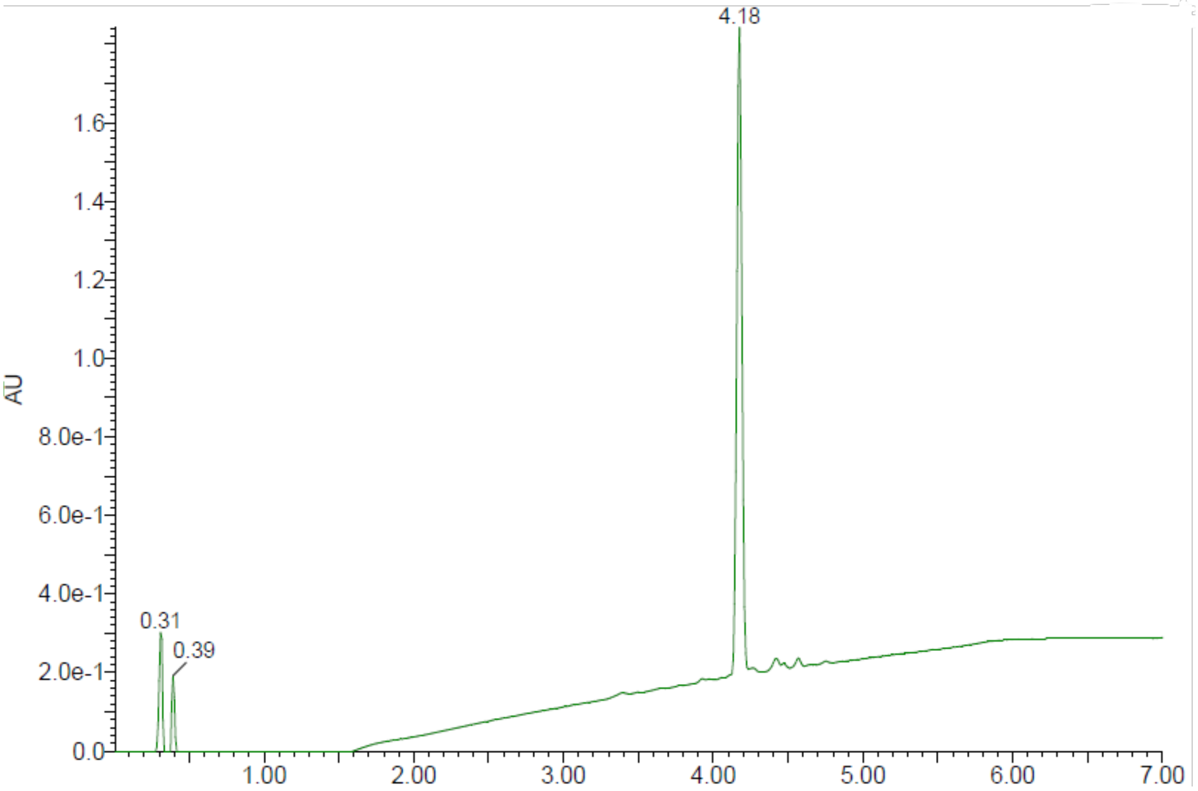

HPLC chromatogram of compound 12

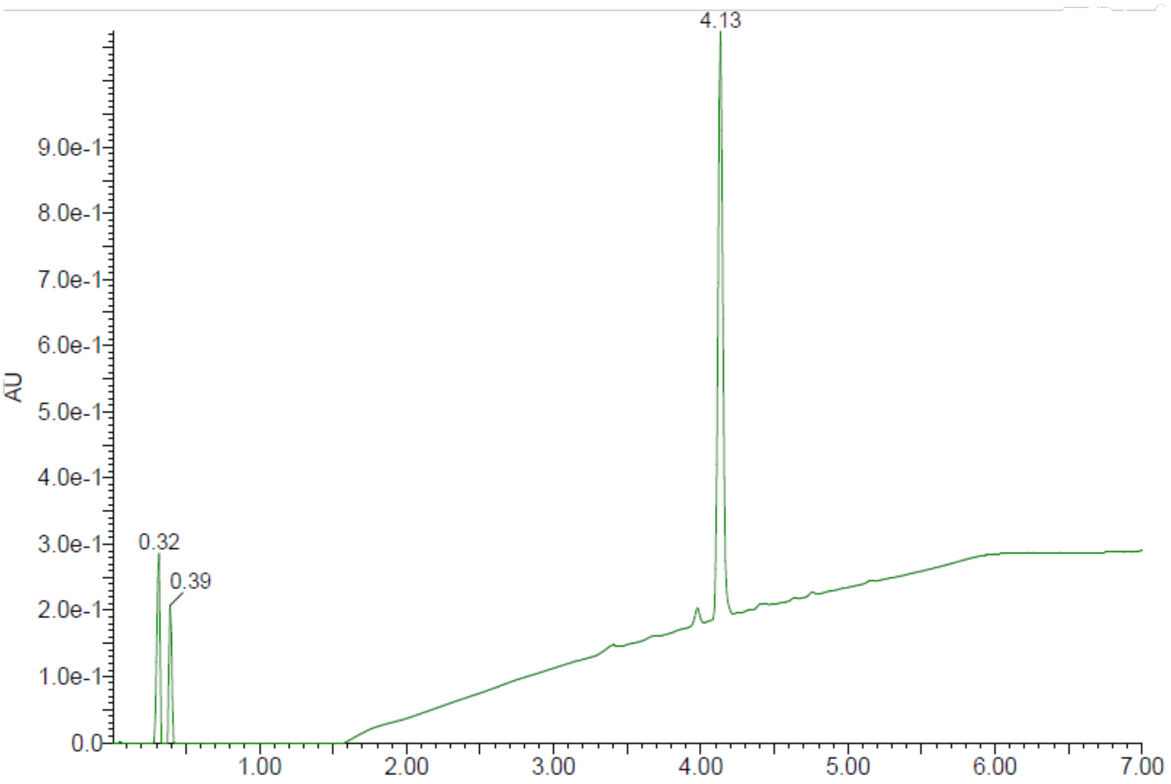

HPLC chromatogram of compound 13

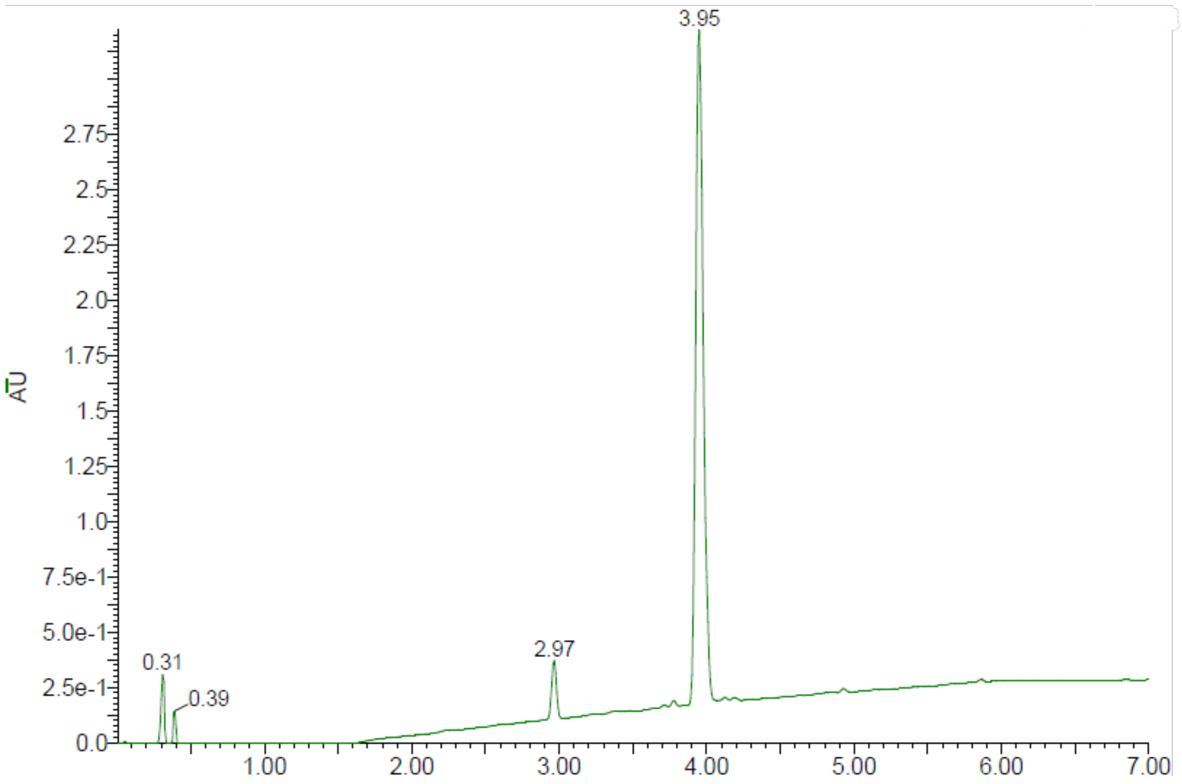

HPLC chromatogram of compound 14

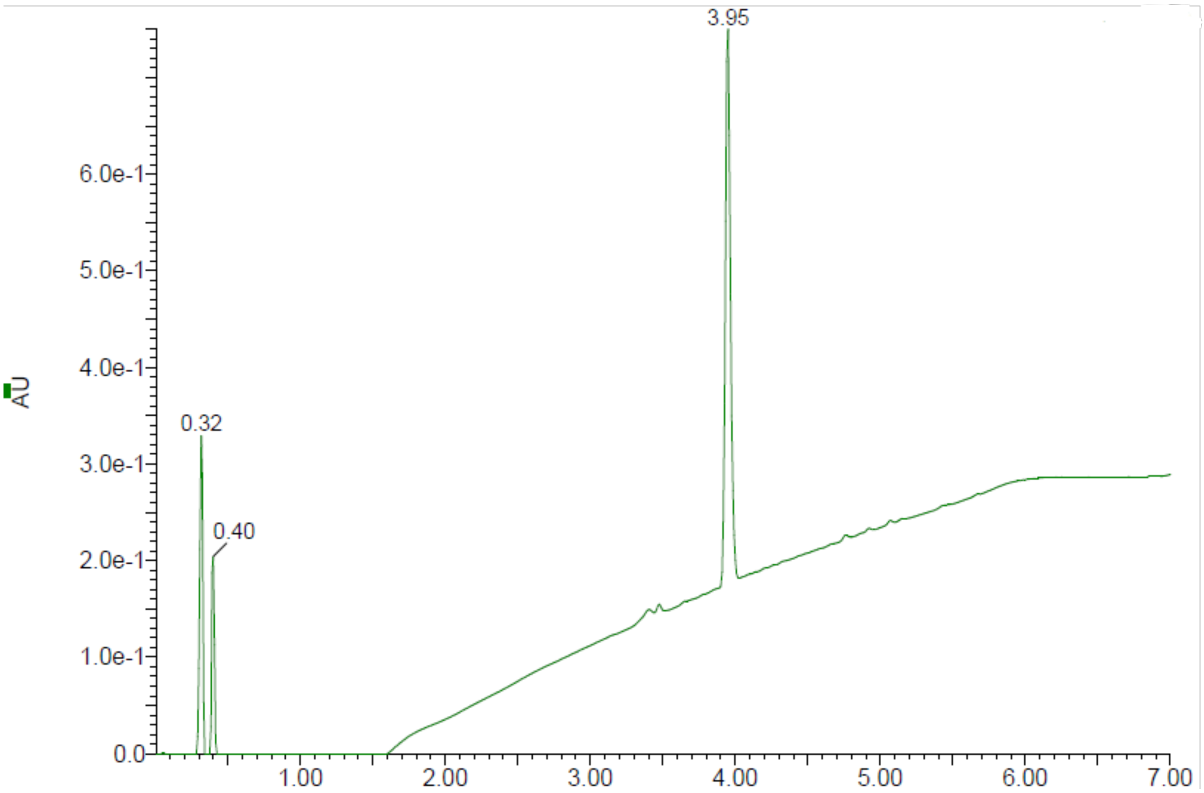

HPLC chromatogram of compound 15

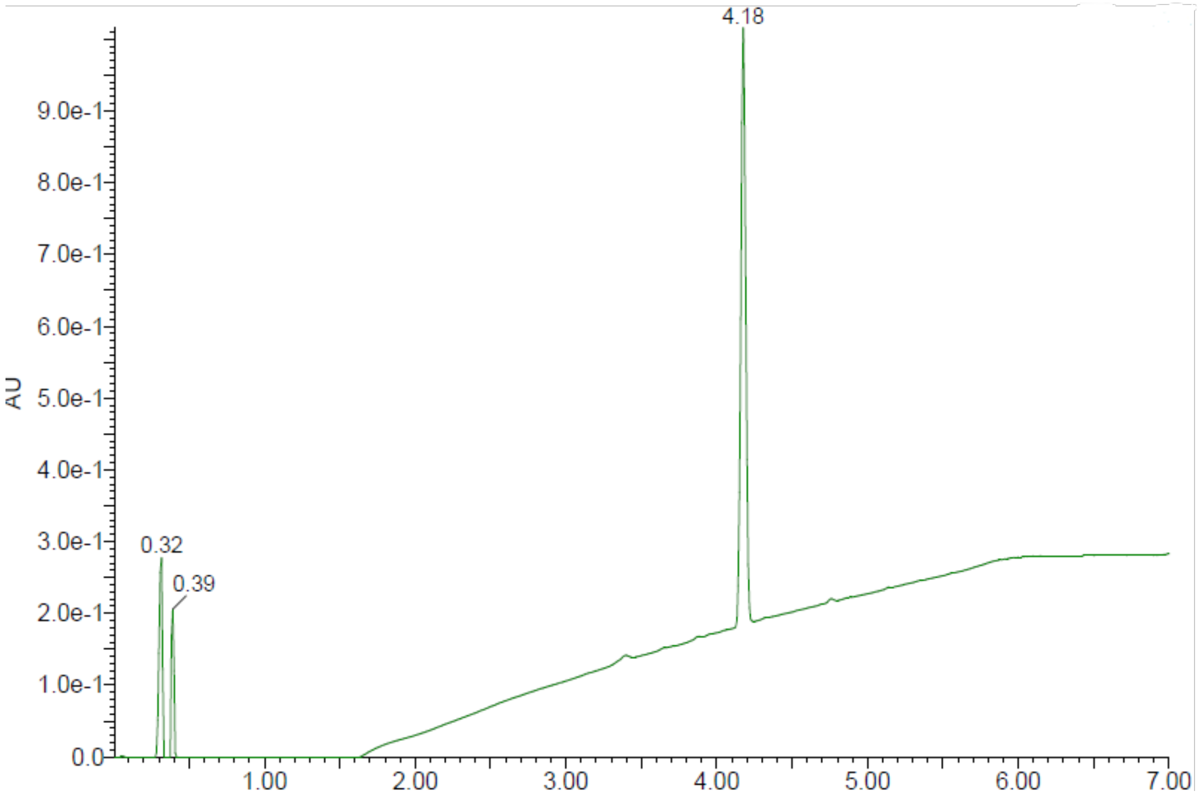

HPLC chromatogram of compound 16

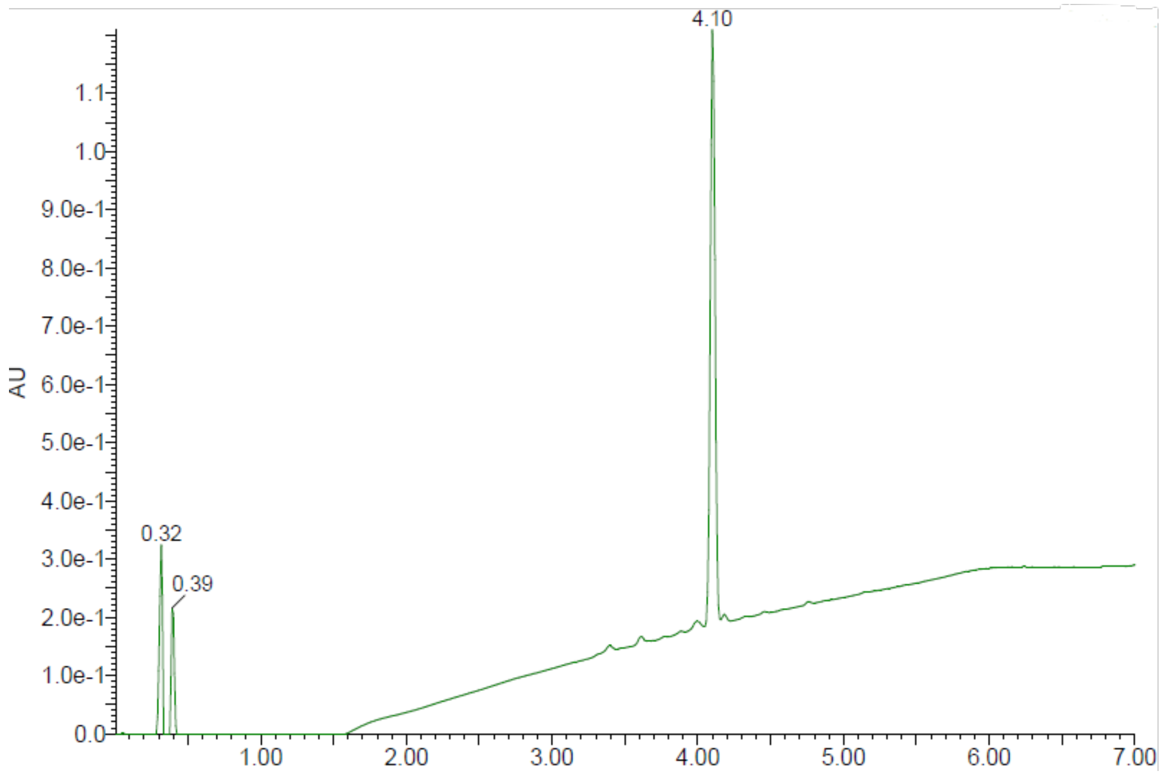

HPLC chromatogram of compound 17

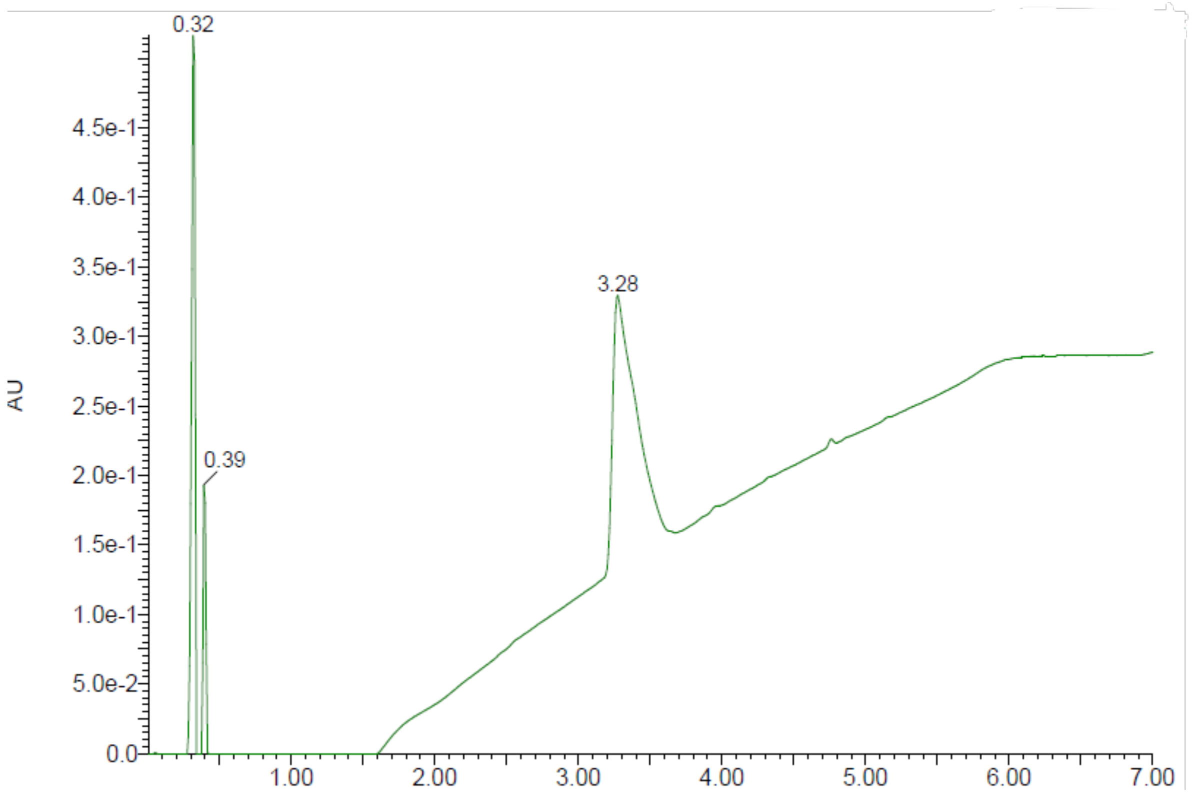

HPLC chromatogram of compound 18

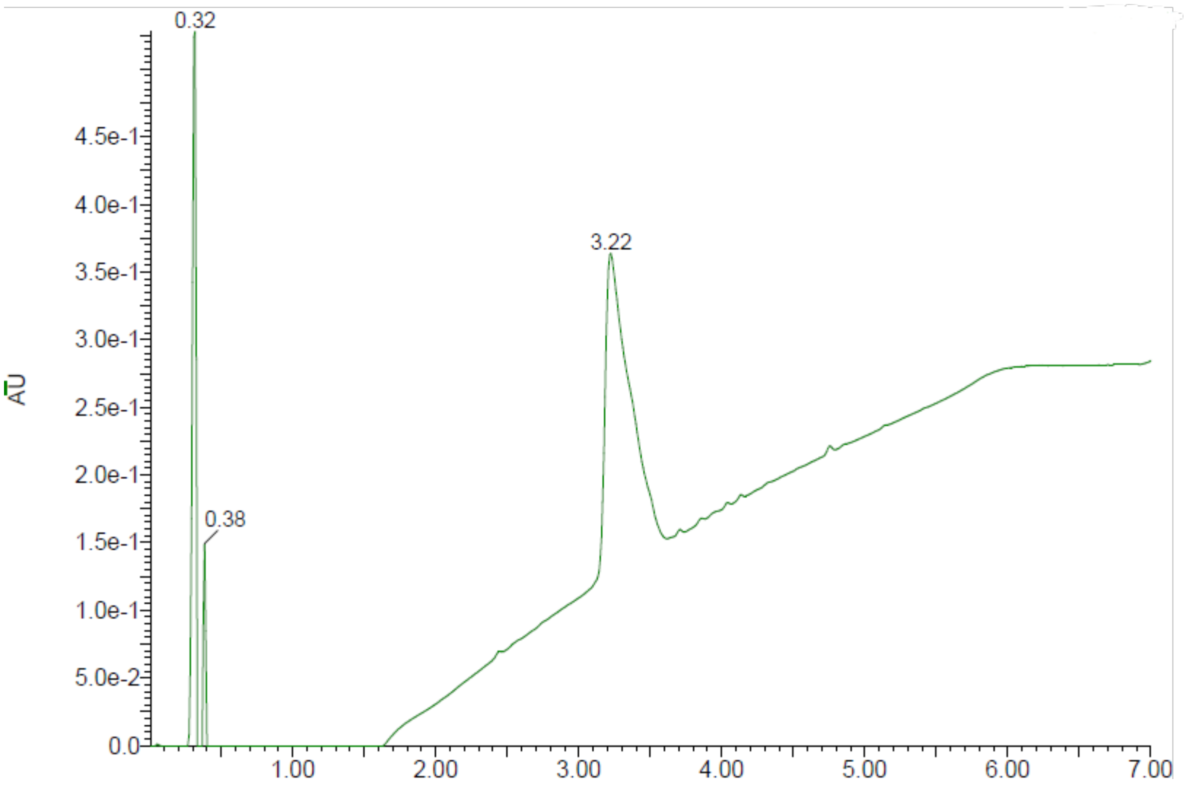

HPLC chromatogram of compound 19

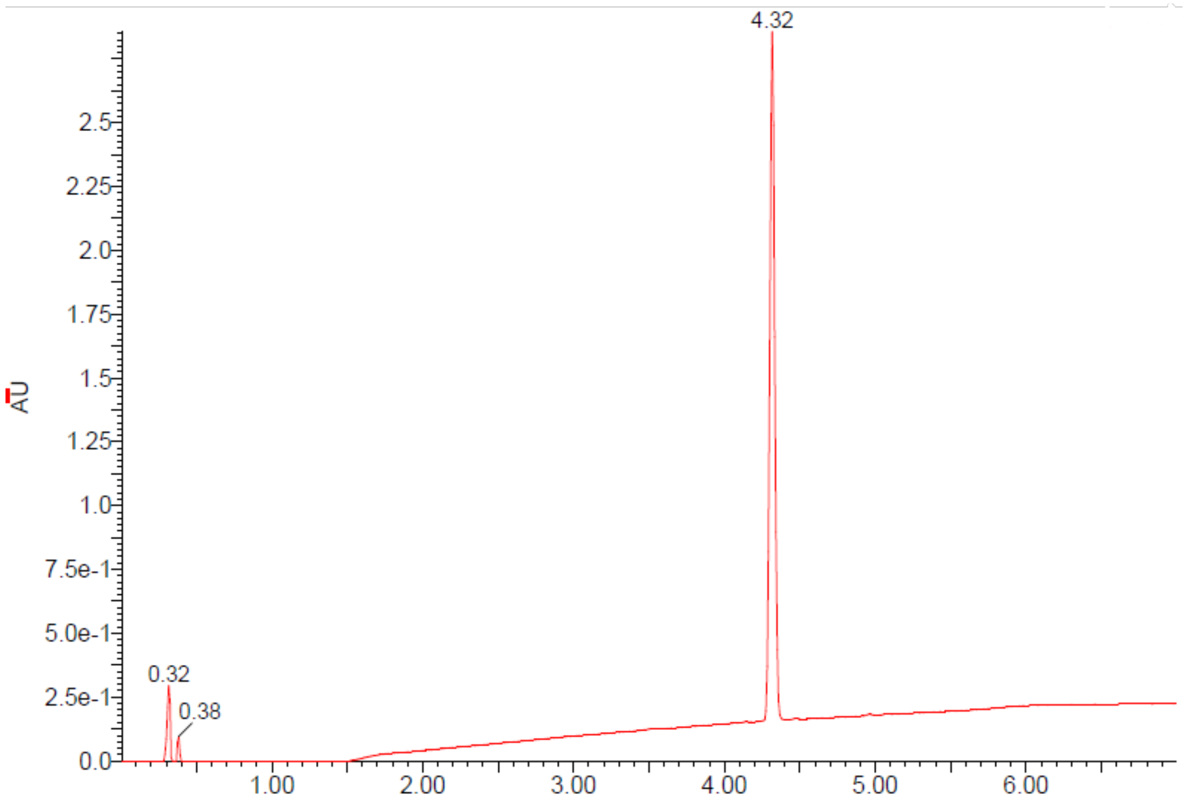

HPLC chromatogram of compound 20

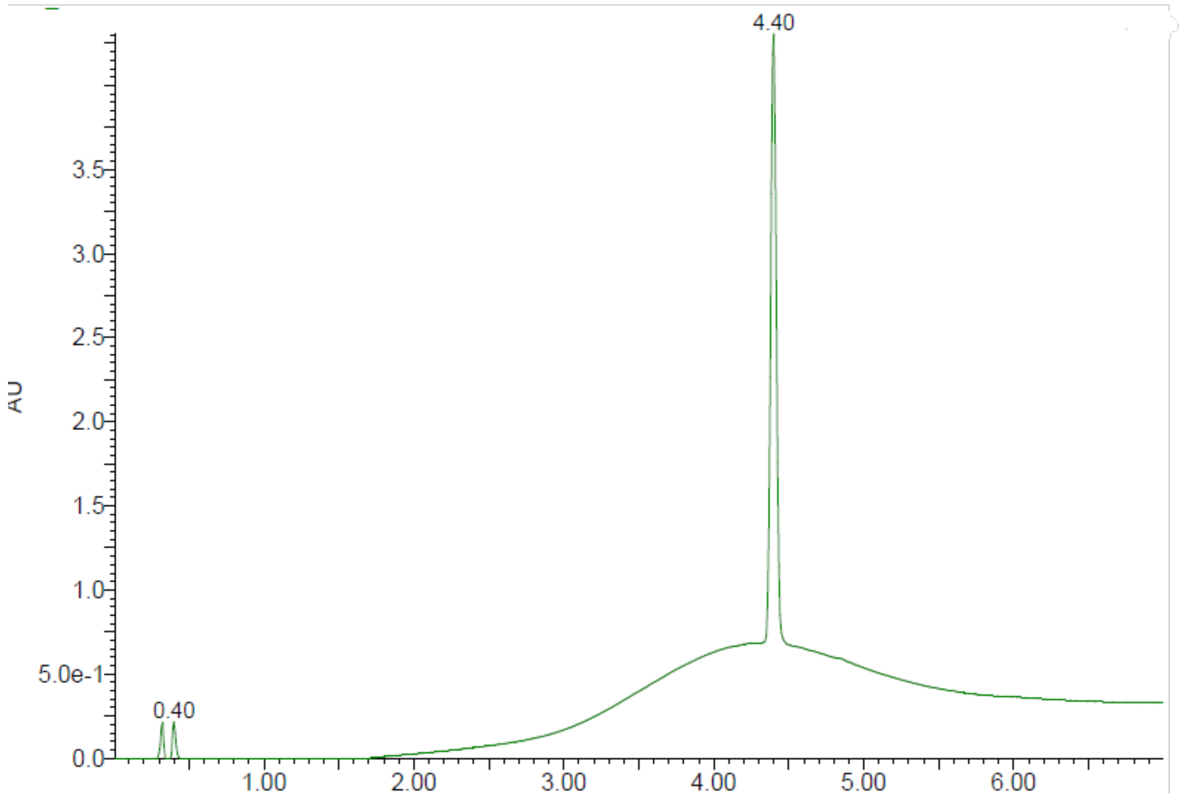

HPLC chromatogram of compound 21

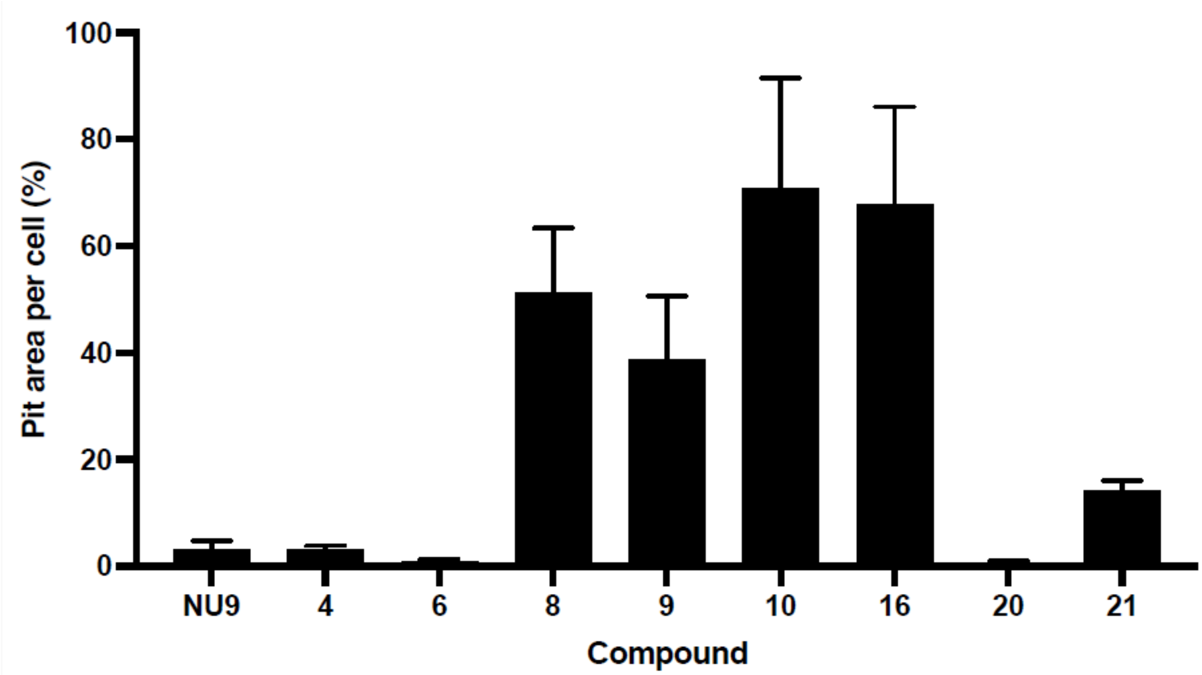

Supplemental Figure 1. Anti-aggregation screening results of **NU-9** compounds and compounds **4**, **6**, **7**, **9**, **10**, **16**, **20** and **21** (10 μM) in PC12-Tet-Off SOD1^{G85R} YFP cells after 72 h incubation. The remaining compounds were tested and did not induce a decrease on MG132 induced SOD-1^{G85R} aggregate formation at 10 μM treatment (inactive compounds).

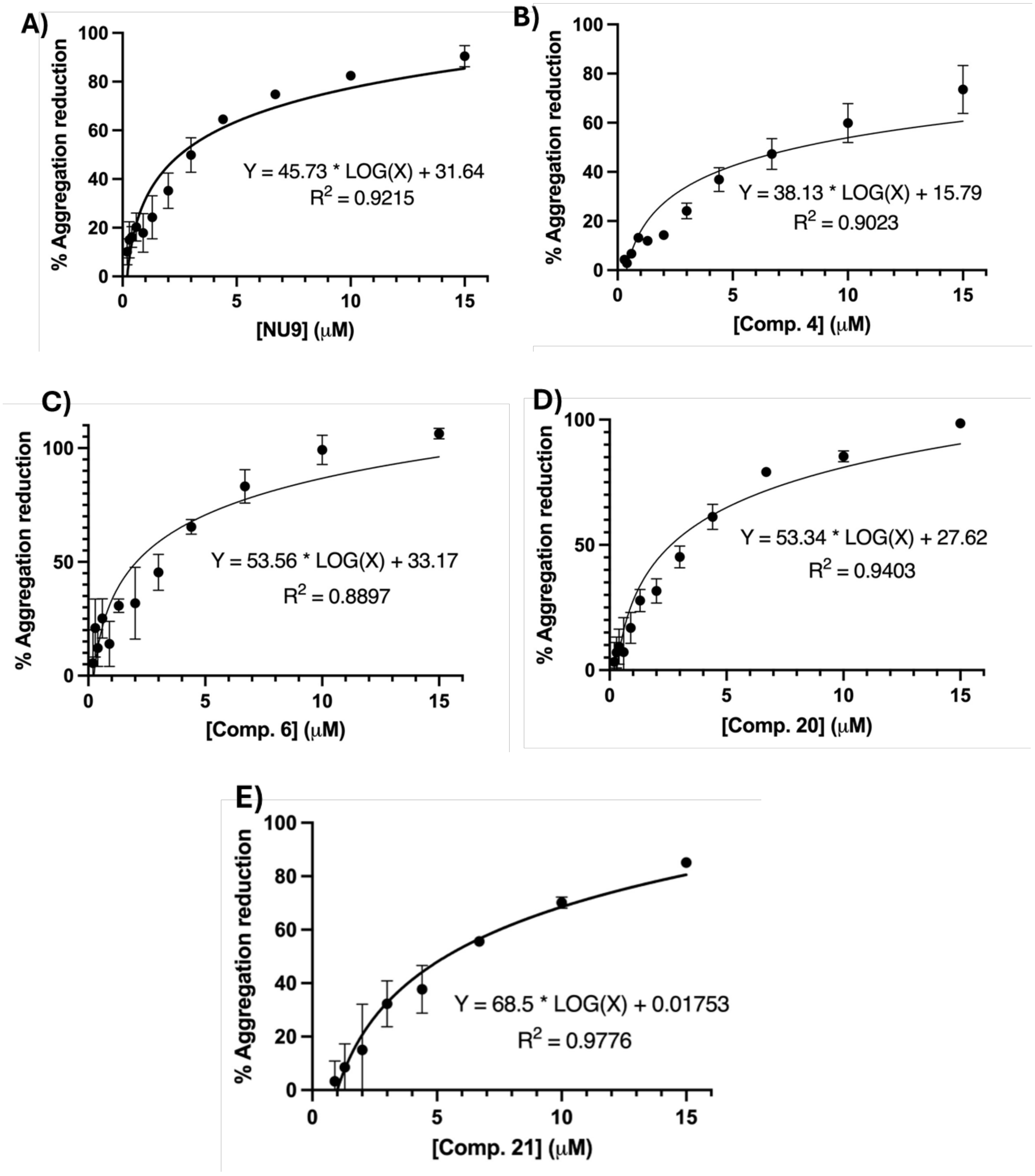

Supplemental Figure 2. Dose-dependent response of **NU-9 (A)** and compounds **4 (B), 6 (C), 20 (D),** and **21** (**E**) in PC12 Tet-Off SOD1^{G85R} YFP.

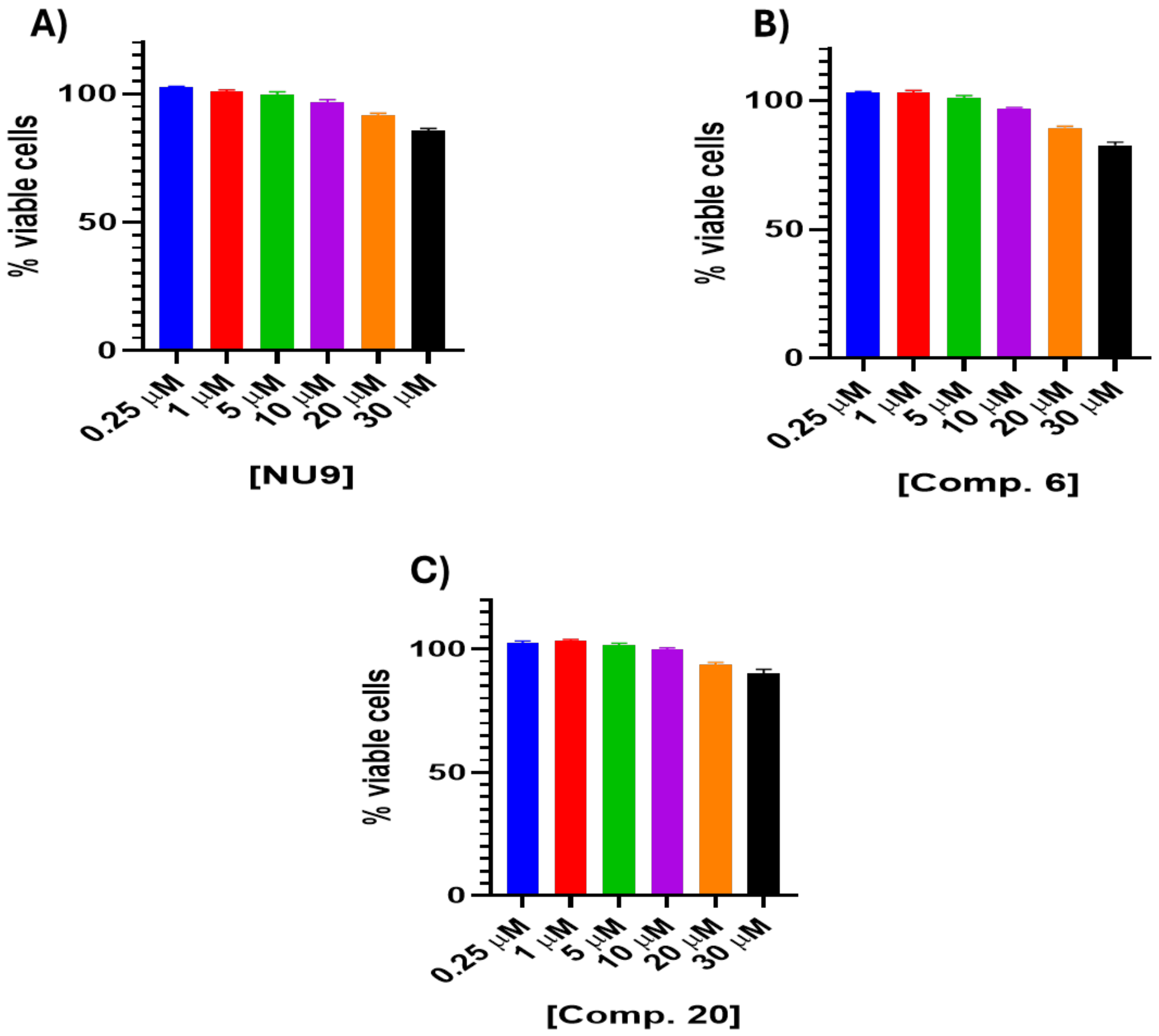

Supplemental Figure 3. Dose-dependent curves of **NU-9 (A), 6 (B)** and **20 (C)** on the cellular viability of HEK293 cells after 24 hours treatment.

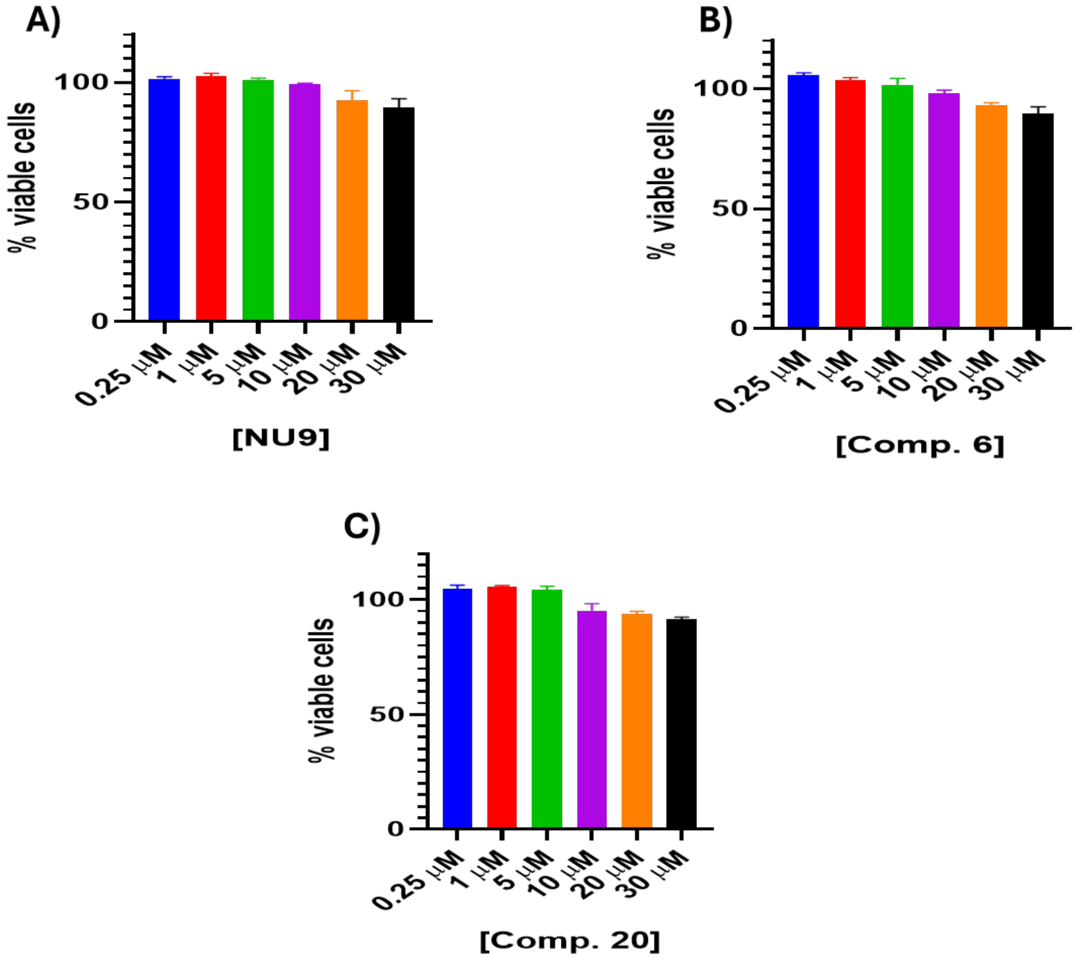

Supplemental Figure 4. Dose-dependent curves of **NU-9 (A), 6 (B)** and **20 (C)** on the cellular viability of HEK293 cells after 48 hours treatment.

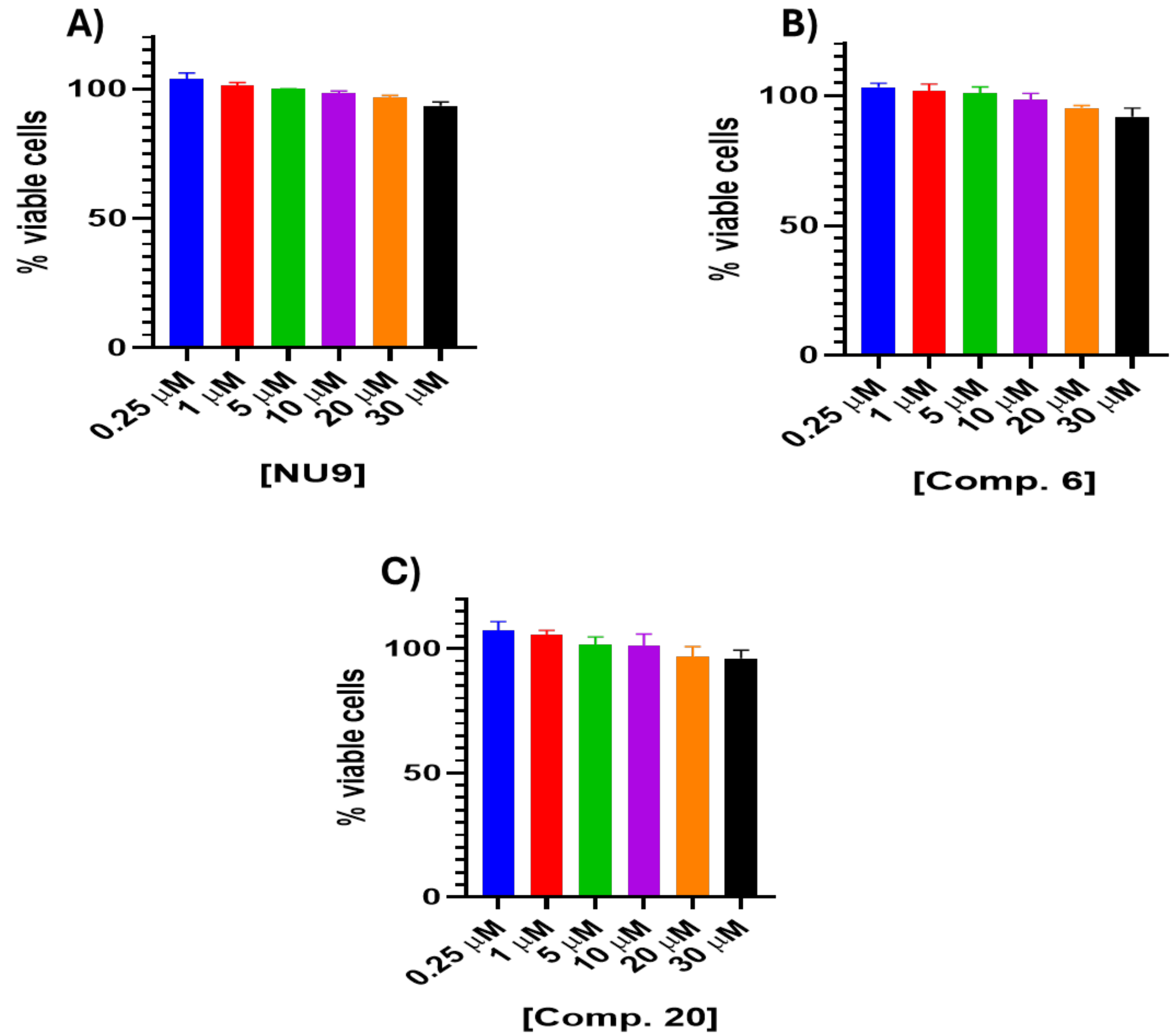

Supplemental Figure 5. Dose-dependent curves of **NU-9 (A), 6** (**B**) and **20 (C)** on the cellular viability of HepG2 cells after 24 hours treatment.

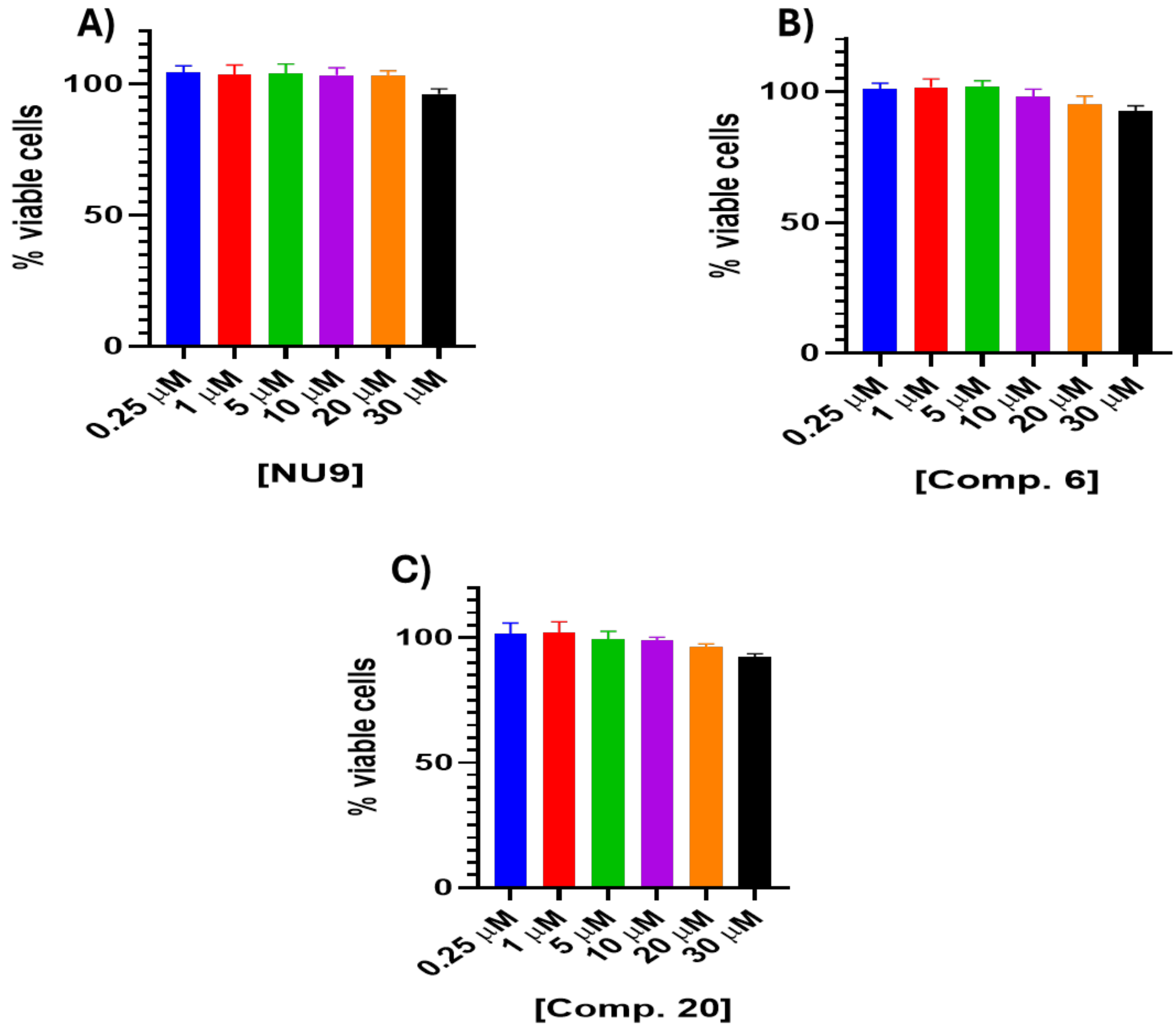

Supplemental Figure 6. Dose-dependent curves of **NU-9 (A), 6 (B)** and **20 (C)** on the cellular viability of HepG2 cells after 48 hours treatment.

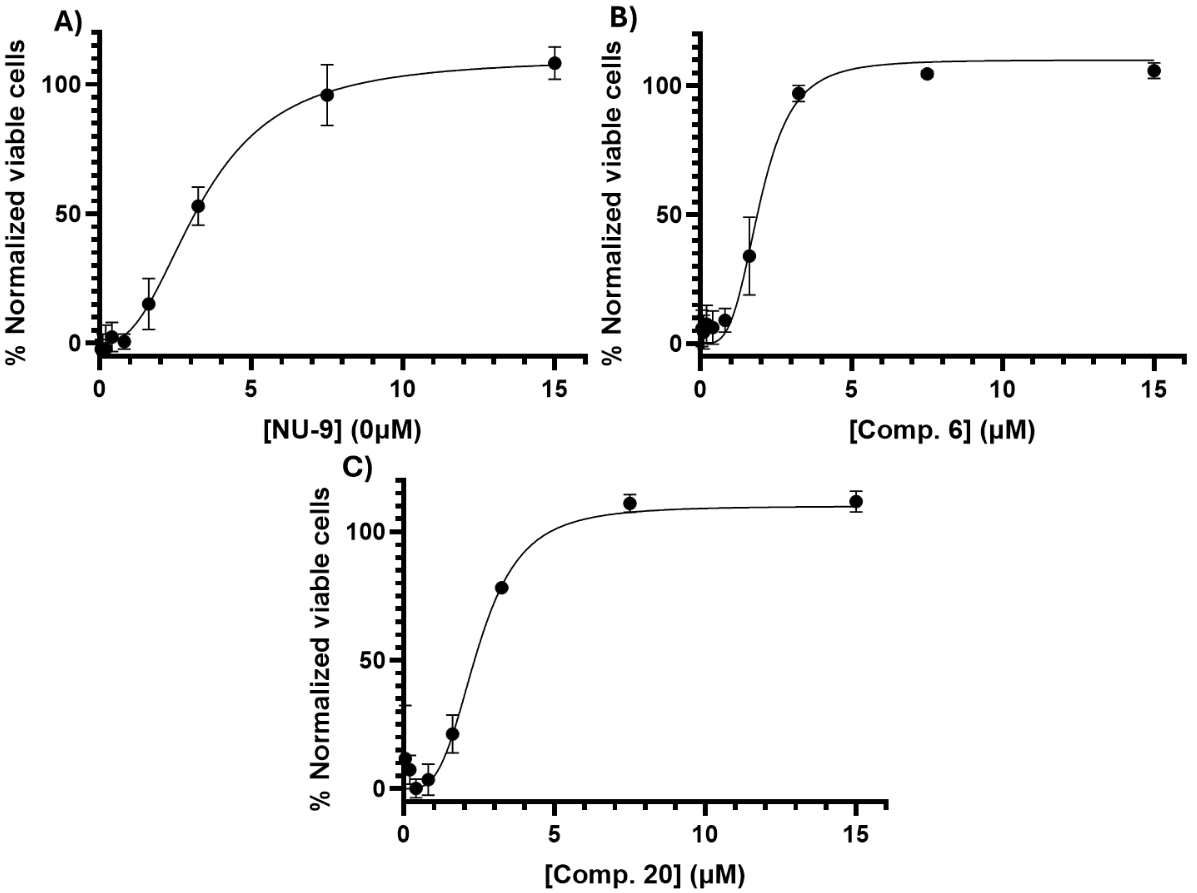

Supplemental Figure 7. Dose-dependent response of **NU-9 (A)** and compounds **6 (B)** and **20 (C),** in PC12 Tet-Off SOD1^{G85R} YFP for the viability studies against MG132-induced toxicity.
